# Clinical and Laboratory Predictors of Poor Neurological Outcomes Following Infectious Encephalitis: Systematic Review and Meta‐Analysis

**DOI:** 10.1111/ene.70445

**Published:** 2025-11-26

**Authors:** Thomas Johnson, Mia Venables, Babak Soleimani, Laurissa Havins, Annapoorna Kannan, Gregory Holt, Jonathan Cleaver, Adam E. Handel, Ava Easton, Defne Saatci, Lahiru Handunnetthi

**Affiliations:** ^1^ Oxford Laboratory for Neuroimmunology and Immunopsychiatry, Centre for Human Genetics University of Oxford Oxford UK; ^2^ Nuffield Department of Clinical Neurosciences University of Oxford Oxford UK; ^3^ Department of Psychiatry University of Oxford Oxford UK; ^4^ Infection Neuroscience Laboratory, Institute of Infection, Veterinary and Ecological Science University of Liverpool Neston UK; ^5^ Encephalitis International Malton UK; ^6^ Nuffield Department of Primary Care Health Sciences University of Oxford Oxford UK

**Keywords:** encephalitis, infection, neurological, outcomes, prognostic

## Abstract

**Background and Objectives:**

Infectious encephalitis is a serious global health problem linked to high rates of mortality and morbidity. However, clinical and laboratory factors that impact neurological outcomes following infectious encephalitis remain poorly understood. Accordingly, we undertook a systematic review and meta‐analysis of clinical and laboratory factors influencing neurological outcomes following infectious encephalitis.

**Methods:**

We searched MEDLINE and EMBASE from inception to 25th September 2023 for observational studies that reported on neurological outcomes at discharge or at ≥ 6 months. We assessed the prognostic value of a priori selected clinical and laboratory‐based features by estimating pooled risk ratios (RRs) through a random‐effects meta‐analysis. The *I* ^2^ statistic was used to assess heterogeneity. This study is registered with PROSPERO (CRD42023485045).

**Results:**

There were several key findings. First, immunocompromised status, status epilepticus, and Glasgow coma scale of < 8 during initial admission were significantly associated with poor neurological outcomes both at discharge and ≥ 6 months after infectious encephalitis onset. Second, CSF leucocytosis [RR: 0.83 95% CI: 0.69–0.98, *p* = 0.03, *n* = 5, *I* ^2^ = 0%] conferred better neurological outcomes while elevated CSF protein [RR: 1.25 95% CI: 1.07–1.46, *p* = 0.006, *n* = 7, *I* ^2^ = 0%] was linked to worse neurological outcomes at discharge. Third, there was no significant association between adjunct steroid therapy and neurological outcomes at discharge and ≥ 6 months.

**Discussion:**

This is the first systematic review and meta‐analysis to investigate prognostic factors linked to neurological outcomes following infectious encephalitis. The results highlight the prognostic value of a range of easily accessible clinical and laboratory parameters.

## Graphical abstract

Clinical and laboratory predictors of long‐term neurological outcomes after infectious encephalitis.

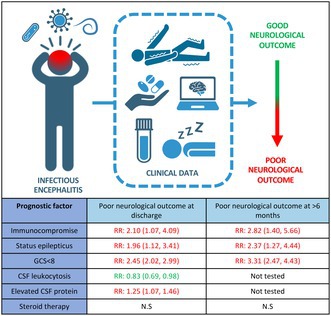

## 1 Introduction

Infectious encephalitis (IE) refers to inflammation in the brain parenchyma precipitated by an infectious agent. This condition can arise from a diverse range of pathogens, notably viruses such as herpes simplex and Japanese encephalitis, as well as parasites including *Toxoplasma gondii*. IE disproportionately affects the youngest and oldest people in the population [1, 2]. It is associated with high rates of mortality and morbidity and represents a substantial economic burden globally [3, 4, 5]. Despite this disease burden, our understanding of prognostic factors such as clinical features and laboratory markers associated with mortality and morbidity outcomes is poor. Further research in this area is needed for effective risk stratification, targeted rehabilitative follow‐up, and allocation of healthcare resources.

Our current knowledge of prognostic factors in IE is predominantly derived from small, often single‐centre, hospital‐based cohort studies that focus on either pathogen‐specific or mixed aetiologies [6, 7, 8, 9]. Whilst the underlying aetiology of the infectious agent is important, it is essential that we understand the role of other prognostic factors for several key reasons. First, the pathological mechanisms underlying brain parenchymal injury involve not only direct pathogen‐mediated damage but also the host immune responses. In particular, dysregulated or excessive immune activation can exacerbate neuronal injury [10, 11]. Second, identification of the causative organism is often a diagnostic challenge, and thus, even in well‐resourced healthcare settings, in approximately 50% of the cases, the pathogen remains unidentified [12]. Third, prognostication whilst awaiting the identification of a pathogen is a common clinical challenge. Fourth, IE is a global disease, and it is important that research reflects the demands of resource‐limited settings of low to middle‐income countries (LMICs) with a paucity of diagnostic tools such as PCR machines [13]. Therefore, there is a need to assess the prognostic value of readily identifiable and universally accessible clinical and laboratory features.

Several clinical and laboratory factors, such as advanced age and ICU admission, provide prognostic value in determining mortality and neurological disability following IE [14, 15]. However, most studies are restricted by their small sample size and report conflicting results. For example, the prognostic value of seizures in relation to poor neurological outcomes at discharge has been studied through several multi‐centre prospective cohorts [14, 16, 17]. Evidence, however, remains inconclusive, with some studies reporting a positive association between seizures and adverse outcomes [14], while others report a non‐significant relationship [16, 17]. This highlights the need for further research in the area. Accordingly, we conducted a systematic review and meta‐analysis of cohort and cross‐sectional studies to investigate the clinical and laboratory features of IE patients and their relationship to mortality and neurological disability outcomes.

## 2 Methods

### 2.1 Search Strategy

We identified potentially eligible studies by systematically searching, with no language restrictions, MEDLINE and EMBASE from inception to 25th September 2023. Our search strategy used subject headings and keywords such as ‘infection’, ‘encephalitis’, ‘viral’, ‘bacterial’, ‘parasitic’, ‘risk factor’, ‘risk’, ‘predictor’, ‘prognosis’, ‘death’, ‘mortality’, and ‘outcome’. Our full search criteria are detailed in File S1.

### 2.2 Study Selection

Eligible studies underwent title and abstract screening by two authors (D.S. and T.J.) to select for full‐text review. Any disagreements were resolved through assessment by a third reviewer (LH). Any studies published in a different language were translated and included in our study if appropriate. Authors of studies that were only available as abstracts were contacted to retrieve the full text. Unpublished conference abstracts were excluded. After study selection, bibliographies of included studies were manually screened to ensure all available studies matching our inclusion criteria had been identified.

Inclusion criteria were (i) a primary cohort study or case series of IE; (ii) involve more than 10 participants; (iii) investigate clinical and laboratory features; and (iv) report on a clinical outcome (mortality and/or neurological disability) within a 5‐year period. In studies where the proportion of non‐IE patients was quantified, we included any study where non‐infectious aetiologies comprised < 10% of the patient cohort. Articles that reported the same hospital of recruitment, period of study, and associated factors were identified as duplicates, and only one study with the largest sample size was included.

### 2.3 Data Extraction and Quality Assessment

We extracted data from included studies using a standardised form, which required information on year of study, study design, population characteristics (country, average/median age, ethnicity, socioeconomic status, aetiology, male: female ratio, ICU vs. non‐ICU cohort), measures of outcome, outcome timeframe, and major results (Table 1; Table S1). To assess the quality and risk of the included studies, the Newcastle‐Ottawa scale and quality in prognostic factor studies (QUIPS) tool were used (Table 1; File S2).

**TABLE 1:** Summary of included studies.

Author, date	Country	Diagnosis	Sample size	NOS	Summary of prognostic factors identified by study
*Mortality at Discharge*					
Dutt et al., 1982	England	HSV‐E	11	6	*No formal statistical analysis undertaken*
Burke et al., 1985	Thailand	JEV‐E	49	8	Serum JEV IgM, serum JEV IgG, CSF JEV IgM, serum JEV IgM, normal consciousness, CSF‐based isolation of virus
Ravi et al., 1997	India	JEV‐E	33	7	Headache, CSF JEV IgM, serum TNF levels
Goh et al., 2000	Malaysia	Nipah virus encephalitis	94	6	Age, vomiting, GCS score, neurological deficits, seizures, serum liver function tests, platelet count
Chong et al., 2002	Malaysia	Nipah virus encephalitis	103	7	Age, tachycardia, fever, hypertension, hyperglycaemia, sweating, inotropic support, requiring intubation and ventilation, antiepileptic agent, myoclonus, thrombocytopaenia, serum liver function tests, lymphopaenia, diabetes mellitus
Baruah et al., 2002	India	JEV‐E	39	6	*No formal statistical analysis undertaken*
Potula et al., 2003	India	JEV‐E	145	7	CSF JEV Ag, CSF JEV IgM, CSF JEV Nt antibody
Murray et al., 2008	North America	WNV‐E	113	7	Renal insufficiency, requiring intubation and ventilation, myoconlus or tremors, CSF pleocytosis
Rao et al., 2008	India	Chandipura virus encephalitis	104	9	**High grade fever, absent oculocephalic reflex, GCS** < **7 (*multi‐variate analysis*)**
Mailles et al., 2009	France	Mixed aetiology—all‐cause encephalitis	249	9	**Age, cancer, immunosuppression, duration of mechanical ventilation, coma on day 5, sepsis at day 5, HSV aetiology, VZV aetiology, *Mycobacterium tuberculosis* aetiology, *Listeria monocytogenes aetiology* (*multi‐variate analysis*)**
VanTan et al., 2010	Vietnam	Mixed aetiology—viral encephalitis	194	7	**Age, GCS score (*on multi‐variate analysis*)**
Sarkari et al., 2011	India	JEV‐E	1199	7	Coma, abnormal breathing, upper gastrointestinal haemorrhage, decerebrate rigidity, pulmonary oedema, peripheral vascular failure, convulsions
Kakoti et al., 2013	India	JEV‐E	61	7	GCS < 8
Patgiri et al., 2014	India	JEV‐E	194	9	Age, unconsciousness, paresis, CSF protein > 50 mg/dL (*multi‐variate analysis*)
Cui et al., 2015	China	SFTS bunyavirus encephalitis	103	7	**Various laboratory parameters, including serum white cell count, plaetelet count, liver function tests, and LDH levels (*on multi‐variate analysis*)**
Modi et al., 2017	North America	HSV‐E	4871	8	Seizures, status epilepticus, acute respiratory failure, ischaemic stroke, intracerebral haemorrhage
Kalita et al., 2017	India	Mixed aetiology—infectious encephalitis	164	9	**SOFA score, treatable aetiology (*multi‐variate analysis*)**
Kakoti et al., 2020	India	JEV‐E	130	7	GCS < 8, meningeal signs
Hu et al., 2020	Taiwan	Mixed aetiology—all‐cause encephalitis	158	9	GCS < 5 or GCS = 5, acute necrotising encephalitis, leukopaenia, serum liver function tests, serum glucose level, co‐infection with influenza A
*Poor Neurological Outcomes at Discharge*					
Takada et al., 1989	Japan	JEV‐E	17	7	Reduced consciousness, JEV vaccination
Klein et al., 1994	North America	Mixed aetiology—viral encephalitis	75	9	Abnormal neuroimaging, ICU admission, altered consciousness
Marton et al., 1996	Israel	HSV‐E	30	6	Duration of illness, reduced consciousness
Bhutto et al., 1999	Pakistan	Mixed aetiology—viral encephalitis	147	7	> 3 days with discrete seizure activity, GCS < 10
Libraty et al., 2002	Thailand	JEV‐E	85	9	Reduced consciousness, fever, serum JEV IgM, serum JEV IgG, CSF JEV IgG, primary flavivirus infection
Solomon et al., 2002	Vietnam	JEV‐E	144	9	**Coma, > 1 witnessed convulsion, herniation syndrome, > 7 days duration of illness (*multi‐variate analysis*)**
Mong et al., 2008*	Malaysia	JEV‐E	118	8	**> 2 witnessed convulsions, hyponatraemia (*multi‐variate analysis*)**
Rayamajhi et al., 2011	Nepal	Mixed aetiology—viral encephalitis	145	8	**Longer duration of fever pre‐admission, reduced consciousness (*multi‐variate analysis*)**
Silverman et al., 2013	North America	EEV‐E	15	7	Short prodrome
Riancho et al., 2013	Spain	HSV‐E	22	6	Disorientation, hyponatraemia, early CT abnormalities
Singh et al., 2014	North America	Mixed aetiology—viral encephalitis	95	9	**Age, immunosuppression, GCS < 8, ICU admission, CSF polymorphonuclear cells (*multi‐variate analysis*)**
Singh et al., 2015	India	JEV‐E/EV‐E	114	9	**Malnutrition, requirement for intubation and ventilation (*multi‐variate analysis*)**
Zhao et al., 2015*	China	Mixed aetiology—infectious encephalitis	1107	9	**Mechanical ventilation, GCS < 8, prodromal infection, abnormal behaviour, seizure activity (*multi‐variate analysis*)**
Singh et al., 2016*	North America	HSV‐E	45	9	**Age, coma, restricted diffusion on MRI, delay in the administration of acyclovir (*multi‐variate analysis*)**
Peng et al., 2017	China	Mixed aetiology—infectious encephalitis	62	9	**Increased CSF S100B levels (*multivariate analysis*)**
Lo et al., 2019	Taiwan	JEV‐E	68	8	**Flaccidity, rigidity, and CSF protein level (*multi‐variate analysis*)**
Hansen et al., 2020	North America	Mixed aetiology—all‐cause encephalitis	340	9	**Age, fever, GCS < 13, seizure activity (*multi‐variate analysis*)**
Hatachi et al., 2021	Japan (national cohort study)	Mixed aetiology—infectious encephalitis	2014	9	**Age, congenital anomalies, epilepsy, reduced consciousness, HSV aetiology, influenza virus, antiepileptic drugs, osmotherapy, intubation and ventilation, vasoactive agents, steroid therapy, immunoglobulin (*multi‐variate analysis*)**
Herlin et al., 2021	Denmark	VZV‐E	92	9	**Age, cerebral vasculitis, GCS < 15 (*multi‐variate analysis*)**
Pata et al., 2021	Italy	Mixed aetiology—infectious encephalitis	53	7	MRI abnormality
Pommier et al., 2022	Cambodia, Vietnam, Laos, and Myanmar	Mixed aetiology—all‐cause encephalitis	664	9	** *Mycobacterium tuberculosis* co‐infection, coma, supplemental oxygen, 1‐week duration between symptoms onset and hospital admission (*multi‐variate analysis*)**
Fillatre et al., 2023	France	Mixed aetiology—infectious encephalitis	198	9	**Immunosuppression, supratentorial focal signs, CSF leukopaenia, abnormal brain imaging, and time from symptoms onset to acyclovir start** > **2 days (*multi‐variate analysis*)**
*Poor neurological outcomes at > 6 months—5 years*					
Kao et al., 1994	China	Mixed aetiology—infectious encephalitis	18	7	SPECT abnormality
Raschilas et al., 2002	France	HSV‐E	85	9	**SOFAII ≥ 27 at admission, delay of 12 days between admission to the hospital and initiation of acyclovir therapy (*multi‐variate analysis*)**
Mong et al., 2008*	Malaysia	JEV‐E	96	9	**Poor perfusion, GCS < 8, ≥ 2 witnessed seizures (*multi‐variate analysis*)**
Riera‐Mestre et al., 2009	Spain	JEV‐E	35	9	**Days of fever after administration of acyclovir, serum hypoalbuminaemia (*multi‐variate analysis*)**
Ma and Jiang, 2013	China	JEV‐E	87	8	**≥ 2 witnessed convulsions, abnormal breathing pattern (multi‐variate analysis)**
Sili et al., 2014	Turkey	HSV‐E	106	9	**Duration of disease prior to hospital admission, MRI abnormalities (*multi‐variate analysis*)**
Zhao et al., 2015*	China	Mixed aetiology—infectious encephalitis	1055	9	**Age, requirement for intubation and ventilation, GCS score, mental and behavioural disorders, seizures (*multi‐variate analysis*)**
Kim et al., 2016	South Korea	HSV‐E	29	9	**EEG abnormalities (*multi‐variate analysis*)**
Zhang et al., 2016	China	HSV‐E	36	6	Twitch, sleepiness, coma, CT/MRI abnormalities, EEG abnormalities
Singh et al., 2016*	North America	HSV‐E	41	9	**Age, coma, restricted diffusion on MRI, delay in the administration of acyclovir (*multi‐variate analysis*)**
Ogata et al., 2017	Japan	HHV‐6 encephalitis	145	9	**Male sex, type of transplanted cells, calcineuron prophylaxis (against GvHD) (*multi‐variate analysis*)**
Liu et al., 2019	China	EV‐E	46	8	Age, GCS score, multiple MRI abnormalities, dorsal medulla oblongata involvement on MRI
Mirouse et al., 2020	France	VZV‐E	55	9	**Age, requirement for intubation and ventilation (*multi‐variate analysis*)**
Wang et al., 2020	China	JEV‐E	112	9	**Elevated CSF pressure and abnormal MRI findings are associated with poor neurological disability outcomes in viral encephalitis (*multi‐variate analysis*)**
Liu et al., 2021	China	JEV‐E	764	8	**Consciousness disorder, respiratory failure, changes in respiratory rhythm, increased muscle tone, history of JE vaccination (*multi‐variate analysis*)**
Srivastava et al., 2022	India	JEV‐E	149	9	**JE unvaccinated status, GCS < 8, malnutrition, requirement for intubation and ventilation (*multi‐variate analysis*)**
He et al., 2023	China	Mixed aetiology—infectious encephalitis	81	9	**Neutrophil:lymphocyte ratio, monocyte: lymphocyte ratio (*multi‐variate analysis*)**
Fan et al., 2023	China	*Mycoplasma pneumoniae* encephalitis	87	9	**Serum LDH, elevated CSF protein level (*multi‐variate analysis*)**

*Note:* Characteristics of included studies. Author, date, country of study, aetiology, sample size, Newcastle‐Ottawa Score (NOS), and major findings are described. Prognostic factors highlighted in bold were derived from multi‐variate models. For full characteristics of included and excluded studies, see Supporting Information. Studies marked with an asterisk (*) highlight cohorts whose neurological outcomes were evaluated at two different time points and whose data were included in the analysis.

### 2.4 Prognostic Factors of Interest

To maximise the objectivity, and therefore clinical utility of the results of our meta‐analysis, we chose to focus on quantifiable prognostic factors and/or those with standardised, internationally recognised criteria. These included demographic factors (including sex, age, deprivation level, and ethnicity), geography (as defined by location of recruitment in rural suburban, or urban areas), seizure activity or diagnosis of epilepsy during admission, focal neurological signs during admission, physiological parameters (including fever and shock), blood test results, CSF markers, microbiology, neuroimaging (including CT and MRI), and medication (specifically: steroids, anti‐seizures, and osmotherapies). When studies did not provide their own definitions for continuous variables, such as CSF pleocytosis and protein count, we used internationally recognised definitions and thresholds and individual patient data to classify patients into ‘exposed’ or ‘unexposed’ groups (File S3).

### 2.5 Outcomes of Interest

There were two primary outcomes of interest: mortality alone and neurological disability defined by standardised rating scales. All studies reporting on mortality alone were analysed separately. Studies reporting composite outcomes that included both mortality and neurological disability using the modified Rankin scale (mRS), Glasgow Outcome scale (GOS), and Liverpool Outcome scale (LOS) were analysed together. Using previously published evidence, we were able to combine the mRS and GOS to generate a binary ‘good’ and ‘poor’ neurological outcome that could be subject to meta‐analysis [18]. For example, an mRS score of 0–2 and GOS score of 4–5 were categorised as ‘good neurological outcome’ while an mRS score of 3–6 and GOS score of 1–3 were categorised as ‘poor neurological outcome’. To evaluate this categorisation approach and assess comparability across these standardised rating scales, we first conducted an epistemic analysis of semantics in relation to the mRS and GOS [19]. Next, using a panel of clinicians (D.S., T.J., and L.H.), we calculated inter‐rater reliability as well as correlation across these scales of neurological disability by using different clinical scenarios (File S4). Furthermore, we carried out sensitivity analyses for the three most widely reported exposures in which studies using mRS and GOS were excluded to evaluate whether the direction of effect reported by studies using non‐standard outcome measures differed.

When follow‐up times varied within a cohort study, the median or mean follow‐up time was used to categorise the timeframe of the clinical outcome. Using these rules, we organised studies into three groups for meta‐analysis: (i) discharge mortality alone, (ii) poor neurological outcome at discharge, and (iii) poor neurological outcome at 6 months to 5 years. These timepoints were selected to delineate patient cohorts with either ‘short‐term’ or ‘long‐term’ outcomes where possible.

### 2.6 Statistical Analysis

Each prognostic factor was analysed separately to calculate pooled risk ratios (RRs) in a generic inverse variance random‐effects model due to the assumed heterogeneity of our patient cohorts.

Heterogeneity between studies was assessed using the *I* ^2^ statistic. The levels of heterogeneity were categorised as low (< 25%), low to moderate (25% to < 50%), moderate to high (50% to < 75%), or high (≥ 75%) [20]. To investigate the robustness of the pooled estimates, a sensitivity analysis was done in which studies with non‐representative patient cohorts (immunologically compromised or requiring intensive care) or extreme effect sizes were removed.

Publication bias was assessed either using Egger's test (if studies > 10) or funnel plots [21]. All analyses were carried out using Review Manager 5.4 and the R package (Version 2023.12.1+402) ‘meta’ [22, 23].

Subgroup analyses were carried out for patient factors identified in our study protocol a priori. Subgroup analyses included (1) the infectious aetiology of their patient cohort using CSF‐based evidence, (2) the infectious aetiology of their patient cohort according to individual study definitions, (3) paediatric and adult cohorts to investigate the effect of age on different exposure effect sizes, and (4) ICU patient cohorts to ensure the applicability of prognostic factors across IE patients with different severities of illness. We assigned ‘paediatric’ or ‘adult’ descriptors to each study cohort based on the study definition.

## 3 Results

**FIGURE 1:**
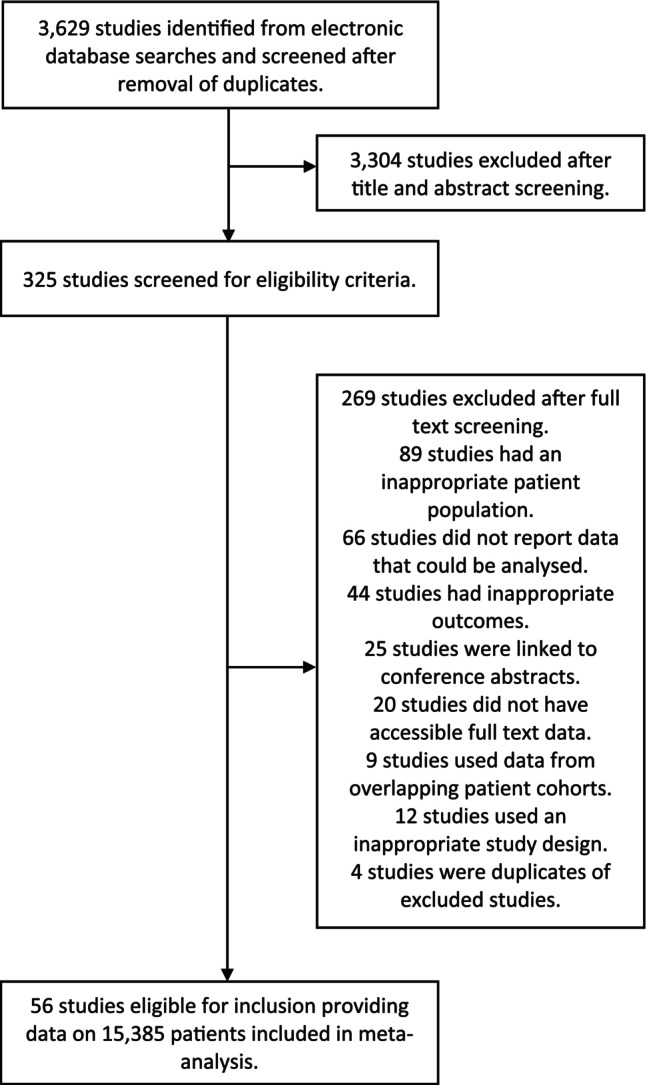
Study selection.

Out of a total of 3629 studies, 56 studies encompassing 15,385 patients were eligible for inclusion in our meta‐analysis (Figure 1 and Table 1). The age range of patient cohorts spanned across paediatric and adult populations, with 20, 26, and 9 studies reporting on adult, paediatric, and mixed (all‐ages) categories, respectively. Using the Newcastle‐Ottawa scale, we rated 90% of our included studies as ‘high quality’ (Table 1). Using the QUIPS tool, we also carried out an in‐depth evaluation of our included prognostic studies and our assessment showed that 15 studies received an assessment of low risk of bias across all 6 domains, and 33 studies had low risk of bias in the prognostic factor measurement domain (File S2).

Twenty‐eight studies provided evidence of an infectious aetiology using CSF‐ and/or serum‐based tests. Fourteen studies identified an infectious agent in the CSF. Consistent with global trends in the aetiology of IE [5], most cohort studies reported on the prognostic factors for Japanese encephalitis virus (JEV) (*n* = 19), followed by herpes simplex virus (HSV) (*n* = 10) (Table 1).

We were able to investigate the role of demographic factors (sex, location), clinical factors (immunocompromise, focal neurology, seizures, status epilepticus) and coma (GCS < 8, intubation and ventilation), investigations (thrombocytopenia, CSF leucocytosis, CSF hypoglycorrhachia, CSF protein, EEG abnormality, CT and MRI abnormalities), and medications (steroids and osmotherapy) in determining poor neurological outcomes when possible as detailed below.

### 3.1 Mortality at Discharge

#### 3.1.1 Results Summary

**FIGURE 2:**
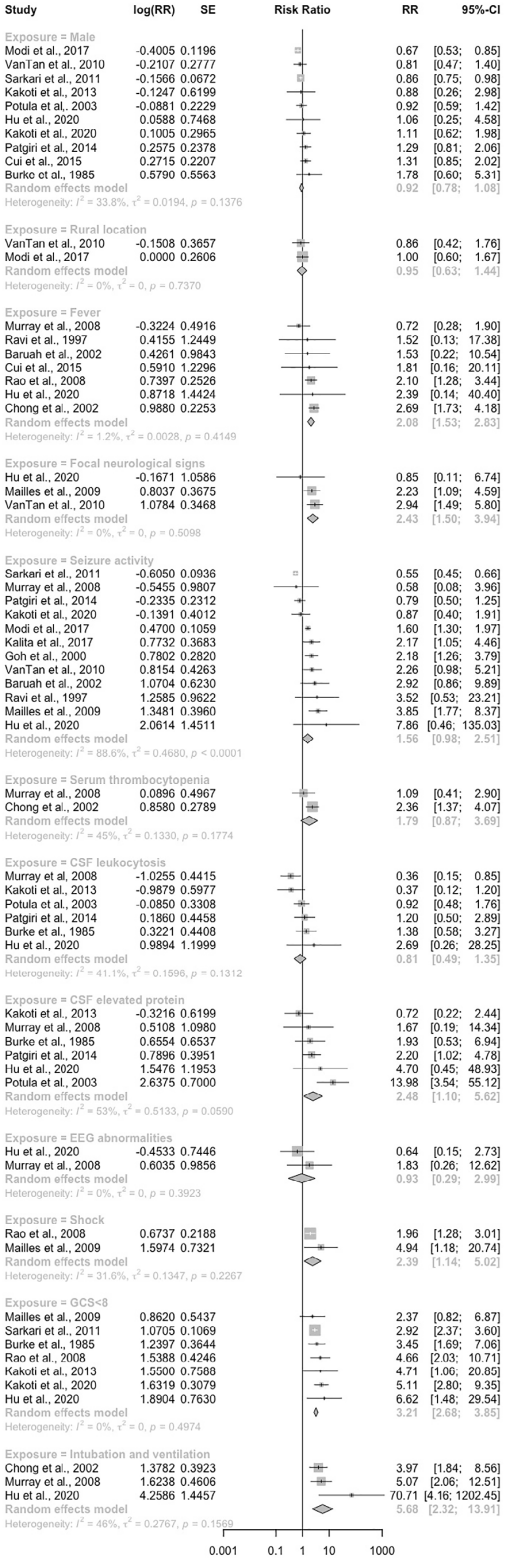
Risk ratio of the effect of clinical and laboratory exposures on mortality at discharge.

Nineteen studies reported on mortality at discharge, and six clinical and laboratory factors were associated with this outcome (Figure 2). The presence of fever (pooled RR: 2.08 [95% CI: 1.53–2.83], *p* = 0.01, *n* = 7, *I* ^2^ = 1%), focal neurological signs (pooled RR: 2.43 [95% CI: 1.50–3.94], *p* = 0.0003, *n* = 3, *I* ^2^ = 0%), elevated CSF protein level (pooled RR: 2.48 [95% CI: 1.10–5.62], *p* = 0.03, *n* = 6, *I* ^2^ = 53%), clinical shock (pooled RR: 2.39 [95% CI: 1.14–5.02], *p* = 0.02, *n* = 2, *I* ^2^ = 32%), GCS < 8 during admission (pooled RR: 3.21 [95% CI: 2.68–3.85], *p* < 0.00001, *n* = 7, *I* ^2^ = 0%), and the requirement for intubation and ventilation (pooled RR: 5.68 [95% CI: 2.32–13.91], *p* = 0.0001, *n* = 3, *I* ^2^ = 46%) were associated with a higher risk of mortality at discharge.

#### 3.1.2 Subgroup Analysis

For the outcome mortality at discharge, seven studies reported on paediatric patients (File S5). We observed an increase in the risk of mortality at discharge in paediatric populations with fever (pooled RR: 2.06 [95% CI: 1.29–3.31], *p* = 0.003, *n* = 3, *I* ^2^ = 0%), GCS < 8 (pooled RR: 3.48 [95% CI: 2.09–5.79], *p* < 0.00001, *n* = 5, *I* ^2^ = 58%) and seizure activity (pooled RR: 2.06 [95% CI: 1.22–3.48], *p* = 0.007, *n* = 4, *I* ^2^ = 0%). Focal neurological signs (pooled RR: 2.36 [95% CI: 0.93–5.99], *p* = 0.07, *n* = 2, *I* ^2^ = 20%) and CSF elevated protein level (pooled RR: 1.42 [95% CI: 0.56–3.57], *p* = 0.46, *n* = 3, *I* ^2^ = 16%) were not linked to discharge mortality in paediatric populations.

Seven studies reported on JEV‐only patient cohorts. We observed an increase in the risk of mortality at discharge in JEV‐only cohorts with CSF elevated protein level (pooled RR: 3.60 [95% CI: 1.18–11.00, *p* = 0.02, *n* = 3, *I* ^2^ = 66%]). JEV‐only cohorts with GCS < 8 were at a lower risk of discharge mortality (pooled RR: 2.98 [95% CI: 2.44–3.64, *p* < 0.00001, *n* = 3, *I* ^2^ = 0%]) (File S5).

#### 3.1.3 Heterogeneity Assessment

Heterogeneity was reported as low or low to moderate for 5 out of the 6 clinical and laboratory features significantly associated with risk of mortality at discharge. The moderate‐to‐high heterogeneity associated with CSF elevated protein was reduced to 37% in our sensitivity analysis (pooled RR: 3.27 [95% CI: 1.49–7.19], *p* = 0.003, *n* = 5, *I* ^2^ = 37%) when we removed the study suggesting that CSF elevated protein level was associated with a lower risk of mortality at discharge, opposing the direction of effect reported by the other five studies [8]. For this outcome, the removal of other individual studies based on extreme effect sizes did not change the direction of effect for any exposure. Similarly, the removal of a single study reporting on an ICU‐only cohort did not influence heterogeneity or the direction of effect of any exposure (File S5) [24].

#### 3.1.4 Publication Bias

Neither funnel plot analysis nor Egger's test revealed any evidence of publication bias (File S6).

### 3.2 Poor Neurological Outcome at Discharge

#### 3.2.1 Results Summary

**FIGURE 3:**
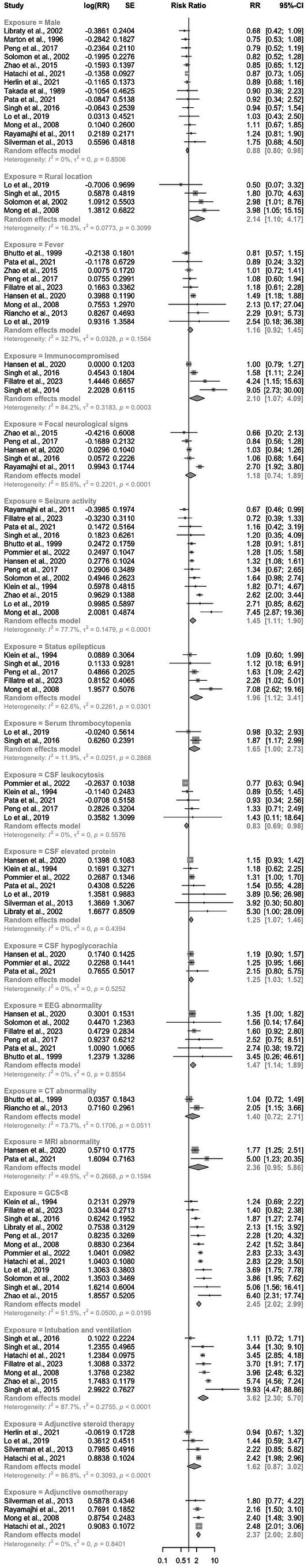
Risk ratio of the effect of clinical and laboratory exposures on poor neurological outcome at discharge.

Twenty‐two studies reported on neurological outcome at discharge, and 12 clinical and laboratory factors were associated with this outcome (Figure 3). Regarding demographic exposures, male sex reduced the risk of poor neurological outcomes at discharge (pooled RR: 0.88 [95% CI: 0.80–0.98], *p* = 0.01, *n* = 14, *I* ^2^ = 0%), whilst living in a ‘rural’ (versus suburban or urban) region increased the risk of poor neurological outcomes (pooled RR: 2.14 [95% CI: 1.10–4.17], *p* = 0.02, *n* = 4, *I* ^2^ = 16%).

##### 3.2.1.1 Clinical Features

Immunocompromised (pooled RR: 2.10 [95% CI: 1.07–4.09], *p* = 0.03, *n* = 4, *I* ^2^ = 84%), seizure activity (pooled RR: 1.45 [95% CI: 1.11–1.90], *p* = 0.007, *n* = 13, *I* ^2^ = 78%) and status epilepticus (pooled RR: 1.96 [95% CI: 1.12–3.41], *p* = 0.02, *n* = 5, *I* ^2^ = 63%), were associated with poor neurological outcomes at discharge.

##### 3.2.1.2 Laboratory Features and Investigations

CSF elevated protein level (pooled RR: 1.25 [95% CI: 1.07–1.46], *p* = 0.006, *n* = 7, *I* ^2^ = 0%), CSF hypoglycorrhachia (pooled RR: 1.25 [95% CI: 1.03–1.52], *p* = 0.03, *n* = 3, *I* ^2^ = 0%), and electroencephalography (EEG) abnormalities (pooled RR: 1.47 [95% CI: 1.14–1.89], *p* = 0.003, *n* = 6, *I* ^2^ = 0%) significantly increased the risk of poor neurological outcomes at discharge. CSF leucocytosis reduced the risk of poor neurological outcomes at discharge (pooled RR: 0.83 [95% CI: 0.69–0.98], *p* = 0.03, *n* = 5, *I* ^2^ = 0%). The association between serum thrombocytopaenia and poor neurological outcome (pooled RR: 1.65 [95% CI: 1.00–2.73], *p* = 0.05, *n* = 2, *I* ^2^ = 12%) at discharge trended towards significance.

##### 3.2.1.3 Level of Consciousness and Critical Care Factors

GCS < 8 on or during admission (pooled RR: 2.45 [95% CI: 2.02–2.99], *p* < 0.00001, *n* = 12, *I* ^2^ = 52%), intubation and ventilation (pooled RR: 3.62 [95% CI: 2.30–5.70], *p* < 0.00001, *n* = 7, *I* ^2^ = 88%), and osmotherapy (pooled RR: 2.37 [95% CI: 2.00–2.80], *p* < 0.00001, *n* = 4, *I* ^2^ = 0%) significantly increased the risk of poor neurological outcomes at discharge. Adjunctive steroid therapy was not associated with the risk of poor neurological outcome at discharge (pooled RR: 1.62 [95% CI: 0.87–3.02], *p* = 0.13, *n* = 4, *I* ^2^ = 87%).

### 3.3 Subgroup Analysis

For the neurological outcome at discharge, 12 studies reported on paediatric patients (File S5). We found that the requirement for intubation and ventilation increased the risk of poor neurological outcomes at discharge in paediatric cohorts (pooled RR: 4.24 [95% CI: 2.62–6.84], *p* < 0.00001, *n* = 3, *I* ^2^ = 63%) as well as in adult and mixed cohorts (pooled RR: 2.99 [95% CI: 1.19–7.50], *p* = 0.02, *n* = 4, *I* ^2^ = 93%).

Five studies reported on JEV‐only cohorts. We found that seizure activity increased the risk of poor neurological outcomes at discharge in JEV‐only cohorts (pooled RR: 3.04 [95% CI: 1.16–7.95], *p* = 0.02, *n* = 3, *I* ^2^ = 73%).

#### 3.3.1 Heterogeneity Assessment

Heterogeneity was reported as low or low to moderate for 10 out of the 16 factors significantly associated with poor neurological outcomes at discharge. The removal of individual studies based on extreme effect sizes did not change the direction of effect or heterogeneity reported as moderate to high for GCS < 8 and status epilepticus or high for immunosuppression, seizure activity, intubation and ventilation. Similarly, the removal of a single study reporting on an ICU‐only cohort [17] did not influence heterogeneity or the direction of effect of any exposure (File S5).

#### 3.3.2 Publication Bias

Neither funnel plot analysis nor Egger's test revealed any evidence of publication bias (File S6).

### 3.4 Poor Neurological Outcomes at > 6 Months

#### 3.4.1 Results Summary

**FIGURE 4:**
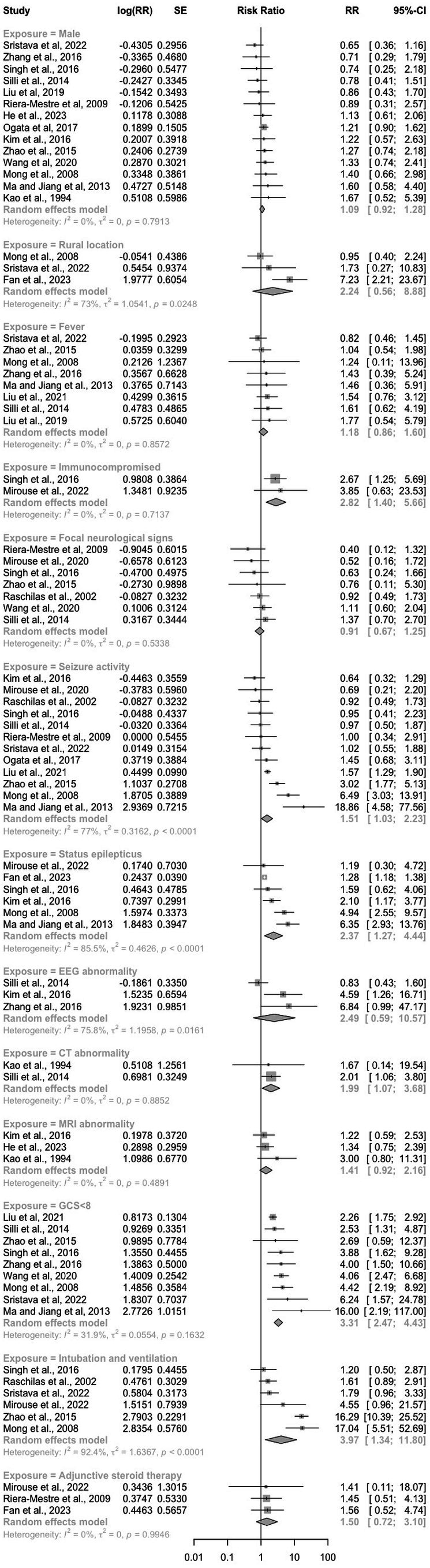
Risk ratio of the effect of clinical and laboratory exposures on poor neurological outcome at greater than 6 months.

Eighteen studies reported on poor neurological outcome at > 6 months, and 6 factors were found to be associated with this outcome (Figure 4). Clinical features: immunocompromised (pooled RR: 2.82 [95% CI: 1.40–5.66], *p* < 0.03, *n* = 2, *I* ^2^ = 0%), seizure activity (pooled RR: 1.51 [95% CI: 1.03–2.23], *p* = 0.04, *n* = 12, *I* ^2^ = 77%) and status epilepticus (pooled RR: 2.37 [95% CI: 1.27–4.44], *p* = 0.007, *n* = 6, *I* ^2^ = 85%) significantly increased the risk of poor neurological outcomes at > 6 months.

Investigations: CT abnormalities (pooled RR: 1.99 [95% CI: 1.07–3.68], *p* = 0.03, *n* = 2, *I* ^2^ = 0%) significantly increased the risk of poor neurological outcomes at > 6 months. Regarding disorders of consciousness and critical care, GCS < 8 (pooled RR: 3.31 [95% CI: 2.47–4.43], *p* < 0.00001, *I* ^2^ = 32%) and the requirement for intubation and ventilation (pooled RR: 3.97 [95% CI: 1.34–11.80], *p* = 0.01, *n* = 6, *I* ^2^ = 92%) significantly increased the risk of poor neurological outcomes at > 6 months. Adjunctive steroid therapy was not associated with the risk of poor neurological outcome at > 6 months (pooled RR: 1.50 [95% CI: 0.72–3.10], *p* = 0.28, *n* = 3, *I* ^2^ = 0%).

#### 3.4.2 Subgroup Analysis

For poor neurological outcomes at > 6 months follow‐up, 7 studies reported on paediatric patients (File S5). We found that status epilepticus increased the risk of poor neurological outcomes at > 6 months to a greater extent in paediatric cohorts (pooled RR: 3.28 [95% CI: 1.01–10.66], *p* = 0.05, *n* = 3, *I* ^2^ = 94%) than in adult and mixed cohorts (pooled RR: 1.83 [95% CI: 1.15–2.93], *p* = 0.01, *n* = 3, *I* ^2^ = 0%). We also found that GCS < 8 significantly increased the risk of poor neurological outcomes at > 6 months in paediatric cohorts (pooled RR: 4.91 [95% CI: 2.95–8.17], *p* < 0.00001, *n* = 4, *I* ^2^ = 0%) as well as adult or mixed cohorts (pooled RR: 2.77 [95% CI: 2.11–3.64], *p* < 0.00001, *n* = 5, *I* ^2^ = 20%).

For poor neurological outcomes at > 6 months follow‐up, six studies reported on JEV‐only patients and five studies reported on HSV‐only patients (File S5). Seizure activity increased the risk of poor neurological outcomes at > 6 months in JEV‐only cohorts (pooled RR: 3.10 [95% CI: 1.24–7.71], *p* < 0.00001, *n* = 4, *I* ^2^ = 89%), but not in HSV‐only cohorts (pooled RR: 0.86 [95% CI: 0.61–1.22], *p* < 0.39, *n* = 4, *I* ^2^ = 0). Status epilepticus was also found to be associated with a higher risk of poor neurological outcomes at > 6 months in JEV‐only cohorts (pooled RR: 5.49 [95% CI: 3.32–9.08], *p* < 0.00001, *n* = 2, *I* ^2^ = 0%) and HSV‐only cohorts (pooled RR: 1.94 [95% CI: 1.18–3.19], *p* = 0.009, *n* = 6, *I* ^2^ = 0%).

#### 3.4.3 Heterogeneity Assessment

Heterogeneity was reported as low or low to moderate for 4 out of the 7 factors significantly associated with poor neurological outcomes at > 6 months follow‐up. The heterogeneity associated with seizure activity and status epilepticus reduced substantially when accounting for age and aetiology. In our sensitivity analysis of studies investigating the requirement for intubation and ventilation, the removal of two studies with outlier effect sizes did not change the direction of effect (File S5) [9, 25].

#### 3.4.4 Publication Bias

Neither funnel plot analysis nor Egger's test revealed any evidence of publication bias (File S6).

## 4 Discussion

This systematic review and meta‐analysis of 56 studies encompassing 15,385 patients identified demographic, clinical, and laboratory features associated with mortality and poor neurological outcomes following IE. Several key findings emerged from this analysis. First, a number of factors were significantly associated with poor neurological outcomes both at discharge and 6 months or more after the onset of IE. These included immunocompromised status, epilepticus during admission, a GCS score of less than 8 during admission. Second, specific laboratory findings such as elevated CSF protein were linked to worse neurological outcomes at discharge, whereas CSF leucocytosis conferred better neurological prognosis at this time point. Third, the role of adjunct steroid therapy was evaluated, and our results found no significant association with neurological outcomes at discharge and 6 months after the onset of IE.

Seizure activity and status epilepticus are common manifestations of IE, particularly amongst viral encephalitides such as JEV [23], and were associated with poor short‐ and long‐term neurological outcomes in our study. In addition, our subgroup analyses showed that paediatric‐only cohorts are particularly vulnerable to the effects of seizure activity. This may reflect the vulnerability of key neurodevelopmental processes that can result in poor long‐term neurological outcomes [26, 27]. Collectively, these findings carry clinical implications. It is possible that active monitoring and controlling seizure activity could, in turn, help to achieve better neurological outcomes following IE. Furthermore, identifying vulnerable patients with protracted seizure activity could help to prioritise those for admission to critical care units who would benefit from sparse intensivist resources.

IE patients require intubation and ventilation due to the risk of airway compromise and loss of ventilatory drive [28]. GCS < 8 and the requirement for intubation and ventilation were associated with poor outcomes at all timepoints. Hypoxic insults secondary to airway compromise, thromboembolic events, microhaemorrhages, and hospital‐acquired infections may all damage the brain parenchyma in the critical care setting, in addition to metabolic and inflammatory threats posed by the infection itself [29, 30]. Focal and global neurological deficits are well‐documented complications of recovery after critical illness [31, 32]. In our subgroup analyses, we observed that JEV patients with GCS < 8 are at a lower risk of discharge mortality when compared to non‐JEV patients. This may be due to survivor bias and the predominance of paediatric patients in the subgroup [7, 8, 33].

Importantly, we found CSF leucocytosis has a protective effect and may serve as an early indicator of better neurological outcomes. This information could support clinical and escalation of care decision‐making. This is also consistent with our observation that immunocompromised patients are at high risk of poor neurological outcomes. Indeed, the protective effect of CSF pleocytosis has previously been reported in immunocompromised HSV‐E patients [34]. A robust leucocytosis in the CSF may indicate the effectiveness of the immune response in the CNS compartment. Mouse models suggest that this is likely to be immune cell and pathogen‐specific [35, 36, 37, 38]. Further work should focus on immune cell‐ and aetiology‐specific analysis of CSF composition of immunocompetent IE patients in relation to neurological outcomes.

CSF elevated protein increased the risk of poor neurological outcome at discharge. Failure of the immune system to control infection may result in direct damage to the brain parenchyma and blood–brain barrier. Therefore, a rise in intrathecal protein concentrations could reflect uncontrolled neuroinflammatory processes [39, 40, 41, 42] Further, our results showed serum thrombocytopaenia trended towards an association with poor neurological outcomes at discharge. This is consistent with previous findings that thrombocytopaenia is an independent predictor of worse outcomes in patients with sepsis [43, 44], and may increase the risk of cerebral microhaemorrhages in IE [45].

We also found that steroid therapy had no impact on neurological outcomes in patients with IE at discharge and > 6 months. This is broadly consistent with the results of several limited cohort studies, although steroid therapy appears to be beneficial in some animal models of IE [46, 47]. The large, multi‐centre, prospective randomised clinical trial ‘DexEnceph’ aims to investigate the benefit of steroids in patients with HSV‐E [48]. The results from this study will help to provide more granular insights into steroid treatment in IE.

### 4.1 Strengths and Limitations

This meta‐analysis has several strengths. First, our results reflect the global burden of IE and included cohort studies from every major continent. This is therefore the first meta‐analysis on this topic with more than 15,000 patients to date. Second, we conducted a rigorous systematic review to identify studies, and after selection, we took several steps to maximise the comparability of studies by grouping them according to available clinical data, outcomes, and outcome timeframe. Third, we systematically explored study heterogeneity through sensitivity analysis and subgroup analyses and identified high‐risk patient groups and aetiology‐specific prognostic factors. Finally, our focus on readily available clinical and laboratory factors is of particular value to clinical decision‐making in resource‐limited LMICs, many of which are disproportionately impacted by the global burden of IE.

This meta‐analysis also has several limitations. The majority of studies included in this meta‐analysis were single‐centre cohort studies which are at risk of selection and information bias. We mitigated the potential impact of this by using a random‐effects model in our meta‐analysis. Due to the intrinsic heterogeneity of IE patient cohorts, inclusion criterion varied considerably between studies. In some instances, patients were only included when an infectious aetiology was established through PCR or serological analysis and/or all non‐infectious causes of encephalitis had been excluded. In contrast, other studies were solely reliant on the international guidelines for encephalitis and/or epidemiological evidence, such as the extraction of data from patients during an epidemic. We accounted for this heterogeneity by performing subgroup analyses of studies with PCR and/or serological evidence of an infectious aetiology. These analyses consistently showed the same direction of effect when all studies were accounted for.

Whilst the identified prognostic factors might provide some insight into management when the causative organism remains unidentified, their generalisability is limited because of the over‐representation of single pathogens, such as HSV and JEV, in our analyses. To address this, we carried out subgroup analyses where possible but further meta‐analyses of larger mixed cohorts amenable to multi‐variate regression analyses will be required to identify pathogen‐independent prognostic factors.

Given the observational nature of the included studies, our findings regarding the efficacy of steroid therapy should be interpreted with caution. Although steroid use was not associated with poor neurological outcomes at discharge or beyond 6 months, treatment decisions may have been influenced by factors such as disease severity and comorbidities, introducing confounding by indication. Furthermore, the inclusion of patients at the time of admission may have biased the results in favour of those who survived long enough to receive steroid therapy, thereby introducing immortal time bias. Randomised clinical trials are necessary to evaluate the potential benefits of steroid therapy in reducing poor neurological outcomes following viral encephalitis.

The inconsistent reporting of our chosen variables by each of these cohorts also restricted the number of studies we could include in each comparison. Our decision to focus on objective clinical and laboratory features in order to maximise applicability in a clinical setting may have also led to the exclusion of subjective exposures, such as signs of meningeal irritation, which may have prognostic value. In addition, we grouped studies using different clinical assessment tools, including the mRS, GOS, and LOS, as well as other tools. To account for this heterogeneity and ensure maximum comparability between studies, we reviewed and compared the outcome definitions in sensitivity analyses. These analyses consistently showed the same direction of effect reported by both established and alternative indices of neurological disability for exposures: biological sex (male), seizure activity, and GCS < 8 (File S5).

### 4.2 Summary

We report, to our knowledge, the first systematic review to assess multiple clinical and laboratory features that can impact mortality and neurological disability following IE. Our findings identify universally accessible and clinically actionable prognostic factors to guide risk stratification, optimise resource allocation, and improve global patient outcomes, especially in resource‐limited settings.

## Author Contributions

**Thomas Johnson:** data curation, methodology, formal analysis, writing – original draft, writing – review and editing, software, visualization. **Mia Venables:** methodology, writing – review and editing. **Babak Soleimani:** methodology, writing – review and editing. **Laurissa Havins:** methodology, writing – review and editing. **Annapoorna Kannan:** methodology, writing – review and editing. **Gregory Holt:** methodology, writing – review and editing. **Jonathan Cleaver:** methodology, writing – review and editing. **Adam E. Handel:** methodology, writing – review and editing. **Ava Easton:** methodology, writing – review and editing. **Defne Saatci:** conceptualization, data curation, formal analysis, methodology, resources, software, writing – original draft, writing – review and editing. **Lahiru Handunnetthi:** conceptualization, funding acquisition, methodology, resources, writing – original draft, writing – review and editing.

## Funding

L.H. is supported by the National Institute for Health and Care Research (NIHR) Oxford Health Biomedical Research Centre, United Kingdom, and NC3Rs (APP50857). A.H. is supported by the Medical Research Council (MRC) (MR/X022013/1 and MR/V007173/1) and Oxford Health Biomedical Research Centre, United Kingdom. The funder of the study had no role in study design, data collection, data analysis, data interpretation, or writing of the report.

## Conflicts of Interest

J.C. is funded by the Guarantors of Brain through an ABN Clinical Research Training Fellowship.

## Supporting information


**File S1:** ene70445‐sup‐0001‐FileS1.docx.

**File S2:** ene70445‐sup‐0002‐FileS2.docx.

**File S3:** ene70445‐sup‐0003‐FileS3.docx.

**File S4:** ene70445‐sup‐0004‐FileS4.docx.

**File S5:** ene70445‐sup‐0005‐FileS5.docx.

**File S6:** ene70445‐sup‐0006‐FileS6.docx.

**Table S1:** ene70445‐sup‐0007‐TableS1.docx.

## Data Availability

The data that support the findings of this study are available from the corresponding author upon reasonable request.

## References

[1] P. Petitgas , P. Tattevin , A. Mailles , et al., “Infectious Encephalitis in Elderly Patients: A Prospective Multicentre Observational Study in France 2016–2019,” Infection 51, no. 4 (2023): 859–867, 10.1007/s15010-022-01927-3. 36152225

[2] J. Kalita , U. K. Misra , S. Pandey , and T. N. Dhole , “A Comparison of Clinical and Radiological Findings in Adults and Children With Japanese Encephalitis,” Archives of Neurology 60, no. 12 (2003): 1760, 10.1001/archneur.60.12.1760. 14676053

[3] C. A. Glaser and K. C. Bloch , “Encephalitis: A Global Problem Deserving of a Global Approach,” Clinical Infectious Diseases 70, no. 12 (2020): 2527–2529, 10.1093/cid/ciz690. 31549167

[4] J. Granerod , Y. Huang , N. W. S. Davies , et al., “Global Landscape of Encephalitis: Key Priorities to Reduce Future Disease Burden,” Clinical Infectious Diseases 77, no. 11 (2023): 1552–1560, 10.1093/cid/ciad417. 37436770 PMC10686956

[5] H. Wang , S. Zhao , S. Wang , et al., “Global Magnitude of Encephalitis Burden and Its Evolving Pattern Over the Past 30 Years,” Journal of Infection 84, no. 6 (2022): 777–787, 10.1016/j.jinf.2022.04.026. 35452715

[6] T. Singh , J. Fugate , S. Hocker , E. Wijdicks , A. Aksamit , and A. Rabinstein , “Predictors of Outcome in HSV Encephalitis,” Neurology 86, no. 16 suppl. 1 (2016): 277–289. 26568560 10.1007/s00415-015-7960-8

[7] G. Kakoti and B. Das , “Clinico‐Epidemiological Characteristics of Hospitalised Acute Encephalitis Syndrome Children and Their Correlation With Case Fatality Rate,” Journal of Family Medicine and Primary Care 9, no. 12 (2020): 5948, 10.4103/jfmpc.jfmpc_1645_20. PMC7928120 33681025

[8] G. Kakoti , P. Dutta , B. Ram Das , J. Borah , and J. Mahanta , “Clinical Profile and Outcome of Japanese Encephalitis in Children Admitted With Acute Encephalitis Syndrome,” BioMed Research International 2013 (2013): 1–5, 10.1155/2013/152656. PMC3891618 24490147

[9] M. H. Ooi , P. Lewthwaite , B. F. Lai , et al., “The Epidemiology, Clinical Features, and Long‐Term Prognosis of Japanese Encephalitis in Central Sarawak, Malaysia, 1997–2005,” Clinical Infectious Diseases 47, no. 4 (2008): 458–468, 10.1086/590008. 18616397

[10] K. Bohmwald , C. A. Andrade , N. M. S. Gálvez , V. P. Mora , J. T. Muñoz , and A. M. Kalergis , “The Causes and Long‐Term Consequences of Viral Encephalitis,” Frontiers in Cellular Neuroscience 15 (2021): 15, 10.3389/fncel.2021.755875. PMC8668867 34916908

[11] C. Chhatbar and M. Prinz , “The Roles of Microglia in Viral Encephalitis: From Sensome to Therapeutic Targeting,” Cellular & Molecular Immunology 18, no. 2 (2021): 250–258, 10.1038/s41423-020-00620-5. 33437050 PMC7802409

[12] A. Boucher , J. L. Herrmann , P. Morand , et al., “Epidemiology of Infectious Encephalitis Causes in 2016,” Médecine et Maladies Infectieuses 47, no. 3 (2017): 221–235, 10.1016/j.medmal.2017.02.003. 28341533

[13] H. Yadav , D. Shah , S. Sayed , S. Horton , and L. F. Schroeder , “Availability of Essential Diagnostics in Ten Low‐Income and Middle‐Income Countries: Results From National Health Facility Surveys,” Lancet Global Health 9, no. 11 (2021): e1553–e1560, 10.1016/S2214-109X(21)00442-3. 34626546 PMC8526361

[14] M. A. Hansen , M. S. Samannodi , R. L. Castelblanco , and R. Hasbun , “Clinical Epidemiology, Risk Factors, and Outcomes of Encephalitis in Older Adults,” Clinical Infectious Diseases 70, no. 11 (2020): 2377–2385, 10.1093/cid/ciz635. 31294449

[15] T. D. Singh , J. E. Fugate , S. Hocker , E. F. M. Wijdicks , A. J. Aksamit , and A. A. Rabinstein , “Predictors of Outcome in HSV Encephalitis,” Journal of Neurology 263, no. 2 (2016): 277–289, 10.1007/s00415-015-7960-8. 26568560

[16] J. D. Pommier , C. Gorman , Y. Crabol , et al., “Childhood Encephalitis in the Greater Mekong Region (the SouthEast Asia Encephalitis Project): A Multicentre Prospective Study,” Lancet Global Health 10, no. 7 (2022): e989–e1002, 10.1016/S2214-109X(22)00174-7. 35714649 PMC9210261

[17] P. Fillatre , A. Mailles , J. P. Stahl , et al., “Characteristics, Management, and Outcomes of Patients With Infectious Encephalitis Requiring Intensive Care: A Prospective Multicentre Observational Study,” Journal of Critical Care 77 (2023): 154300, 10.1016/j.jcrc.2023.154300. 37207520

[18] B. Gaastra , D. Ren , S. Alexander , et al., “Evidence‐Based Interconversion of the Glasgow Outcome and Modified Rankin Scales: Pitfalls and Best Practices,” Journal of Stroke and Cerebrovascular Diseases 31, no. 12 (2022): 106845, 10.1016/j.jstrokecerebrovasdis.2022.106845. 36309002 PMC11295112

[19] H. Van Den Tooren , A. Easton , C. Hooper , et al., “How Should We Define a ‘Good’ Outcome From Encephalitis? A Systematic Review of the Range of Outcome Measures Used in the Long‐Term Follow‐Up of Patients With Encephalitis,” Clinical Medicine 22, no. 2 (2022): 145–148, 10.7861/clinmed.2021-0505. 35197253 PMC8966817

[20] J. P. T. Higgins , “Measuring Inconsistency in Meta‐Analyses,” BMJ 327, no. 7414 (2003): 557–560, 10.1136/bmj.327.7414.557. 12958120 PMC192859

[21] L. Lin and H. Chu , “Quantifying Publication Bias in Meta‐Analysis,” Biometrics 74, no. 3 (2018): 785–794, 10.1111/biom.12817. 29141096 PMC5953768

[22] “Review Manager (RevMan),” Published Online (2020).

[23] S. Balduzzi , G. Rücker , and G. Schwarzer , “How to Perform a Meta‐Analysis With R: A Practical Tutorial,” Evidence‐Based Mental Health 22, no. 4 (2019): 153–160, 10.1136/ebmental-2019-300,117. 31563865 PMC10231495

[24] J. Kalita , V. E. Mani , S. K. Bhoi , and U. K. Misra , “Spectrum and Outcome of Acute Infectious Encephalitis/Encephalopathy in an Intensive Care Unit From India,” QJM 110 (2016): 141–148, 10.1093/qjmed/hcw132. 27512107

[25] L. Zhao , M. Zhou , B. Wang , J. Guo , N. Chen , and L. He , “Clinical Characteristics and Outcome of Clinically Diagnosed Viral Encephalitis in Southwest China,” Neurological Sciences 36, no. 12 (2015): 2191–2197, 10.1007/s10072-015-2333-8. 26205533

[26] G. L. Holmes and Y. Ben‐Ari , “Seizures in the Developing Brain,” Neuron 21, no. 6 (1998): 1231–1234, 10.1016/S0896-6273(00)80642-X. 9883716

[27] G. L. Holmes and Y. Ben‐Ari , “The Neurobiology and Consequences of Epilepsy in the Developing Brain,” Pediatric Research 49, no. 3 (2001): 320–325, 10.1203/00006450-200,103,000-00004. 11228256

[28] L. A. Diaz‐Arias , C. A. Pardo , and J. C. Probasco , “Infectious Encephalitis in the Neurocritical Care Unit,” Current Treatment Options in Neurology 22, no. 6 (2020): 18, 10.1007/s11940-020-00623-7.

[29] S. Blot , E. Ruppé , S. Harbarth , et al., “Healthcare‐Associated Infections in Adult Intensive Care Unit Patients: Changes in Epidemiology, Diagnosis, Prevention and Contributions of New Technologies,” Intensive & Critical Care Nursing 70 (2022): 103227, 10.1016/j.iccn.2022.103227. 35249794 PMC8892223

[30] F. Fan , C. Yang , X. Zhu , et al., “Association Between Infectious Burden and Cerebral Microbleeds: A Pilot Cross‐Sectional Study,” Annals of Clinical Translational Neurology 8, no. 2 (2021): 395–405, 10.1002/acn3.51285. 33410595 PMC7886034

[31] A. N. LacKamp and R. D. Stevens , “Neurologic Implications of Critical Illness and Organ Dysfunction,” in Textbook of Neurointensive Care (Springer, 2013), 409–425, 10.1007/978-1-4471-5226-2_21.

[32] P. P. Pandharipande , T. D. Girard , J. C. Jackson , et al., “Long‐Term Cognitive Impairment After Critical Illness,” New England Journal of Medicine 369, no. 14 (2013): 1306–1316, 10.1056/NEJMoa1301372. 24088092 PMC3922401

[33] W. Lorsomrudee , C. J. Leake , C. H. Hoke , et al., “Fatal Outcome in Japanese Encephalitis,” American Journal of Tropical Medicine and Hygiene 34, no. 6 (1985): 1203–1210, 10.4269/ajtmh.1985.34.1203. 3010752

[34] I. L. Tan , J. C. McArthur , A. Venkatesan , and A. Nath , “Atypical Manifestations and Poor Outcome of Herpes Simplex Encephalitis in the Immunocompromised,” Neurology 79, no. 21 (2012): 2125–2132, 10.1212/WNL.0b013e3182752ceb. 23136265 PMC3511927

[35] J. D. Brien , J. L. Uhrlaub , and J. Nikolich‐Žugich , “West Nile Virus‐Specific CD4 T Cells Exhibit Direct Antiviral Cytokine Secretion and Cytotoxicity and Are Sufficient for Antiviral Protection,” Journal of Immunology 181, no. 12 (2008): 8568–8575, 10.4049/jimmunol.181.12.8568. PMC3504655 19050276

[36] P. Kumar , P. Sulochana , G. Nirmala , R. Chandrashekar , M. Haridattatreya , and V. Satchidanandam , “Impaired T Helper 1 Function of Nonstructural Protein 3–Specific T Cells in Japanese Patients With Encephalitis With Neurological Sequelae,” Journal of Infectious Diseases 189, no. 5 (2004): 880–891, 10.1086/381768. 14976606

[37] D. Růžek , J. Salát , M. Palus , et al., “CD8+ T‐Cells Mediate Immunopathology in Tick‐Borne Encephalitis,” Virology 384, no. 1 (2009): 1–6, 10.1016/j.virol.2008.11.023. 19070884

[38] M. Palus , J. Vojtíšková , J. Salát , et al., “Mice With Different Susceptibility to Tick‐Borne Encephalitis Virus Infection Show Selective Neutralizing Antibody Response and Inflammatory Reaction in the Central Nervous System,” Journal of Neuroinflammation 10, no. 1 (2013): 847, 10.1186/1742-2094-10-77. PMC3700758 23805778

[39] C. A. Santacruz , J. L. Vincent , A. Bader , et al., “Association of Cerebrospinal Fluid Protein Biomarkers With Outcomes in Patients With Traumatic and Non‐Traumatic Acute Brain Injury: Systematic Review of the Literature,” Critical Care 25, no. 1 (2021): 278, 10.1186/s13054-021-03698-z. 34353354 PMC8340466

[40] M. Veje , V. Griška , J. Pakalnienė , et al., “Serum and Cerebrospinal Fluid Brain Damage Markers Neurofilament Light and Glial Fibrillary Acidic Protein Correlate With Tick‐Borne Encephalitis Disease Severity—A Multicentre Study on Lithuanian and Swedish Patients,” European Journal of Neurology 30, no. 10 (2023): 3182–3189, 10.1111/ene.15978. 37431060

[41] I. E. van Zeggeren , L. ter Horst , H. Heijst , C. E. Teunissen , D. van de Beek , and M. C. Brouwer , “Neurofilament Light Chain in Central Nervous System Infections: A Prospective Study of Diagnostic Accuracy,” Scientific Reports 12, no. 1 (2022): 14140, 10.1038/s41598-022-17,643-9. 35986031 PMC9391449

[42] Q. L. Peng , S. H. Tao , N. Yu , X. Z. Zhou , Y. Z. Peng , and N. Fu , “Elevated Levels of Cerebrospinal Fluid S100B Are Associated With Brain Injury and Unfavorable Outcomes in Children With Central Nervous System Infections,” International Journal of Neuroscience 127, no. 1 (2017): 1–9, 10.3109/00207454.2015.1135334. 26710878

[43] S. Esposito , G. De Simone , G. Boccia , F. De Caro , and P. Pagliano , “Sepsis and Septic Shock: New Definitions, New Diagnostic and Therapeutic Approaches,” Journal of Global Antimicrobial Resistance 10 (2017): 204–212, 10.1016/j.jgar.2017.06.013. 28743646

[44] R. Zarychanski and D. S. Houston , “Assessing Thrombocytopenia in the Intensive Care Unit: The Past, Present, and Future,” Haematology 2017, no. 1 (2017): 660–666, 10.1182/asheducation-2017.1.660. PMC6142536 29222318

[45] N. Cooper , M. A. Morrison , C. Vladescu , et al., “Identification of Occult Cerebral Microbleeds in Adults With Immune Thrombocytopenia,” Blood 136, no. 25 (2020): 2875–2880, 10.1182/blood.2020004858. 32750707 PMC7751366

[46] E. Hodzic , R. Hasbun , A. Granillo , et al., “Steroids for the Treatment of Viral Encephalitis: A Systematic Literature Review and Meta‐Analysis,” Journal of Neurology 270, no. 7 (2023): 3603–3615, 10.1007/s00415-023-11,715-0. 37060361 PMC10105360

[47] S. Gundamraj and R. Hasbun , “The Use of Adjunctive Steroids in Central Nervous Infections,” Frontiers in Cellular and Infection Microbiology 10 (2020), 10.3389/fcimb.2020.592017. PMC7719626 33330135

[48] T. Whitfield , C. Fernandez , K. Davies , et al., “Protocol for DexEnceph: A Randomised Controlled Trial of Dexamethasone Therapy in Adults With Herpes Simplex Virus Encephalitis,” BMJ Open 11, no. 7 (2021): e041808, 10.1136/bmjopen-2020-041808. PMC8728349 34301646

