# Supplementary material for: Clinical and Laboratory Predictors of Poor Neurological Outcomes Following Infectious Encephalitis: Systematic Review and Meta‐Analysis
Source: Eur J Neurol. 2025 Nov 26;32(12):e70445. doi: 10.1111/ene.70445 (PMC12649060; doi:10.1111/ene.70445)
Supplement: Supplementary file 1 — File S1: ene70445‐sup‐0001‐FileS1.docx. [file ENE-32-e70445-s006.docx]

**Supplementary File 1 - Protocol and search strategy**

Medline and EMBASE will be systematically searched, with no language restrictions, from inception to MEDLINE and EMBASE will be systematically searched, with no language restrictions, from inception to 25th September for the following search terms within the title and abstract of the publication; “infection”, “encephalitis”, “viral”, “bacterial”, “parasitic”, “risk factor”, “risk”, “predictor”, “prognosis”, “death”, “mortality, “outcome”.

Specifically:
“((infectio*) adj2 encephalit*).ab,ti
OR
((vir* OR bacteri* OR parasi*) adj4 encephalit*).ab,ti OR
encephalit*.ab,ti
AND
(risk* adj2 factor*).ab,ti
OR
risk*.ab,ti
OR
predict*.ab,ti
OR
prognos*.ab,ti
AND
dea*.ab,ti
OR
mortal*.ab,ti
OR

outcome*.ab,ti”

**Identification of Studies**

Title and abstracts will be screened by one researcher (DS) and full text reviews by two researchers. Cohort studies with at least 10 participants, exploring prognostic factors for mortality in patients with infectious encephalitis. All non-English studies will be translated.

*Inclusion Criteria*

P = Any patient population >10 with a clinical diagnosis of infectious encephalitis (with or without CSF or radiological evidence of infection)

I/ C = All clinical and laboratory parameters reported for each patient group with infectious encephalitis in each study cohort.

O = patient mortality (at discharge, 6-months, 12-months, reported as an odds ratio or hazard ratio, and morbidity (with a focus on neurological sequalae/ disability, using scoring systems such as the ‘modified Rankin Scale’)

S = All observational (prospective or retrospective) cohort studies

*Exclusion Criteria*

- Non-infectious causes of encephalitis
- Does not report on relevant prognostic factors
- Does not report on mortality e.g. studies investigating risk of morbidity at 6-months would be excluded
- Any other study design (RCT, non-RCT, case-control, case series, cross-sectional)

We will assess the quality of all selected studies using the Newcastle-Ottawa scale.

**Data Extraction and Analysis**

Data will be extracted using a standardised form. It includes information on publication details, number of participants, participant characteristics, exposures (described above), study outcomes, measures of effect, confidence intervals, and potential causes of bias reported by the authors.

We anticipate variation in the patient populations, outcomes, and prognostic factors investigated by the studies identified through our search protocol. For example, some studies may exclude patients without a definitive (laboratory- or imaging-confirmed encephalitis) diagnosis of infectious encephalitis, whilst others may include patients with persistent fever and confusion (presumed encephalitis). Studies may report on patient mortality at discharge, 6-months, or 12-months. Studies may exclusively investigate the effect of a single clinical factor (e.g. time between onset of symptoms and treatment) on mortality, whilst other studies may instead investigate the effect of multiple clinical and laboratory-based parameters.

We will extract the data as presented within individual studies and obtain effect sizes specific to each prognostic factor. Initially, we will synthesise the pooled effect size of each prognostic factor for all studies using patient populations that meet our inclusion criteria. Given the variable composition of our patient populations as highlighted above, we will then conduct further subgroup analysis, accounting for factors such as i) age, ii) infectious agent, iii) CSF/ radiological evidence of confirmed infection, iv) ICU admission, and v) geographical location, where able.

Different descriptions of prognostic risk factors which overall imply the same exposure (severe hypotension versus septic shock) will be combined for the meta-analysis.

Our studies are likely to report on both continuous (e.g. age) and binary (e.g. abnormal versus normal neuroimaging) exposure variables. For binary variables, the number of patients in each group and the total number of patients will be recorded. Any missing data will be addressed by contacting authors. For any overlapping data across studies, only the most recent and/or relevant publication will be included.

**Statistical Analysis**

Effect sizes (odd’s ratios and hazard ratios) from each study will be inputted to determine the weighted average (pooled) effect size of each prognostic variable, through random- effects meta-analysis.

For continuous data with significant skew, weighted proportions from each study will be log transformed to fit a normal distribution and untransformed to provide interpretable results.

Furthermore, as between-study variation is expected across included observational studies, the pooled proportion will be calculated using the restricted maximum likelihood (REML) random- effects model.

Heterogeneity will be measured using I2**.** This measure assesses the percentage of the total observed variance, which can be accounted for by between-study variation.

Our meta-analysis of proportions will be carried out using the “meta” package in R.
