# Supplementary material for: Clinical and Laboratory Predictors of Poor Neurological Outcomes Following Infectious Encephalitis: Systematic Review and Meta‐Analysis
Source: Eur J Neurol. 2025 Nov 26;32(12):e70445. doi: 10.1111/ene.70445 (PMC12649060; doi:10.1111/ene.70445)
Supplement: Supplementary file 2 — File S2: ene70445‐sup‐0002‐FileS2.docx. [file ENE-32-e70445-s007.docx]

**Supplementary File 3 – QUIPS analysis**

Mortality at discharge: QUIPS analysis

| Study title: | Clinical investigation of hospitalized human cases of West Nile virus infection in Houston, Texas, 2002-2004 | | |
| --- | --- | --- | --- |
| Study author/ data: | Murray et al., 2008 | | |
| Study identifier: | 10.1089/vbz.2007.0109 | | |
|  | | | |
| Biases | Issues to consider for judging overall rating of "Risk of bias" | Rating of reporting | Rating of "Risk of bias" |
| **1. Study Participation** | Goal: To judge the risk of selection bias (likelihood that relationship between PF and outcome is different for participants and eligible non-participants). |  |  |
| Source of target population | The source population or population of interest is adequately described for key characteristics (LIST). | Yes |  |
| Method used to identify population | The sampling frame and recruitment are adequately described, including methods to identify the sample sufficient to limit potential bias (number and type used, e.g., referral patterns in health care) | No |  |
| Recruitment period | Period of recruitment is adequately described | Partial |  |
| Place of recruitment | Place of recruitment (setting and geographic location) are adequately described | Partial |  |
| Inclusion and exclusion criteria | Inclusion and exclusion criteria are adequately described (e.g., including explicit diagnostic criteria or “zero time” description). | Partial |  |
| Adequate study participation | There is adequate participation in the study by eligible individuals | Yes |  |
| Baseline characteristics | The baseline study sample (i.e., individuals entering the study) is adequately described for key characteristics (LIST). | Partial |  |
| **Summary Study participation** | The study sample represents the population of interest on key characteristics, sufficient to limit potential bias of the observed relationship between PF and outcome. |  | Moderate |
|  | | | |
| **2. Study Attrition** | Goal: To judge the risk of attrition bias (likelihood that relationship between PF and outcome are different for completing and non-completing participants). |  |  |
| Proportion of baseline sample available for analysis | Response rate (i.e., proportion of study sample completing the study and providing outcome data) is adequate. | No |  |
| Attempts to collect information on participants who dropped out | Attempts to collect information on participants who dropped out of the study are described. | No |  |
| Reasons and potential impact of subjects lost to follow-up | Reasons for loss to follow-up are provided. | No |  |
| Outcome and prognostic factor information on those lost to follow-up | Participants lost to follow-up are adequately described for key characteristics (LIST)./ There are no important differences between key characteristics (LIST) and outcomes in participants who completed the study and those who did not. | No |  |
| **Study Attrition Summary** | Loss to follow-up (from baseline sample to study population analyzed) is not associated with key characteristics (i.e., the study data adequately represent the sample) sufficient to limit potential bias to the observed relationship between PF and outcome. |  | Low |
|  | | | |
| **3. Prognostic Factor Measurement** | Goal: To judge the risk of measurement bias related to how PF was measured (differential measurement of PF related to the level of outcome). |  |  |
| Definition of the PF | A clear definition or description of 'PF' is provided (e.g., including dose, level, duration of exposure, and clear specification of the method of measurement). | No |  |
| Valid and Reliable Measurement of PF | Method of PF measurement is adequately valid and reliable to limit misclassification bias (e.g., may include relevant outside sources of information on measurement properties, also characteristics, such as blind measurement and limited reliance on recall)./ Continuous variables are reported or appropriate cut-points (i.e., not data-dependent) are used. | Partial |  |
| Method and Setting of PF Measurement | The method and setting of measurement of PF is the same for all study participants. | Yes |  |
| Proportion of data on PF available for analysis | Adequate proportion of the study sample has complete data for PF variable. | Yes |  |
| Method used for missing data | Appropriate methods of imputation are used for missing 'PF' data. | Yes |  |
| **PF Measurement Summary** | PF is adequately measured in study participants to sufficiently limit potential bias. |  | Low |
|  | | | |
| 4. Outcome Measurement | Goal: To judge the risk of bias related to the measurement of outcome (differential measurement of outcome related to the baseline level of PF). |  |  |
| Definition of the Outcome | A clear definition of outcome is provided, including duration of follow-up and level and extent of the outcome construct. | Yes |  |
| Valid and Reliable Measurement of Outcome | The method of outcome measurement used is adequately valid and reliable to limit misclassification bias (e.g., may include relevant outside sources of information on measurement properties, also characteristics, such as blind measurement and confirmation of outcome with valid and reliable test). | Yes |  |
| Method and Setting of Outcome Measurement | The method and setting of outcome measurement is the same for all study participants. | Yes |  |
| **Outcome Measurement Summary** | Outcome of interest is adequately measured in study participants to sufficiently limit potential bias. |  | Low |
|  | | | |
| **5. Study Confounding** | Goal: To judge the risk of bias due to confounding (i.e. the effect of PF is distorted by another factor that is related to PF and outcome). |  |  |
| Important Confounders Measured | All important confounders, including treatments (key variables in conceptual model: LIST), are measured. | Partial |  |
| Definition of the confounding factor | Clear definitions of the important confounders measured are provided (e.g., including dose, level, and duration of exposures). | No |  |
| Valid and Reliable Measurement of Confounders | Measurement of all important confounders is adequately valid and reliable (e.g., may include relevant outside sources of information on measurement properties, also characteristics, such as blind measurement and limited reliance on recall). | No |  |
| Method and Setting of Confounding Measurement | The method and setting of confounding measurement are the same for all study participants. | Yes |  |
| Method used for missing data | Appropriate methods are used if imputation is used for missing confounder data. | No |  |
| Appropriate Accounting for Confounding | Important potential confounders are accounted for in the study design (e.g., matching for key variables, stratification, or initial assembly of comparable groups). /Important potential confounders are accounted for in the analysis (i.e., appropriate adjustment). | No |  |
| **Study Confounding Summary** | Important potential confounders are appropriately accounted for, limiting potential bias with respect to the relationship between PF and outcome . |  | High |
|  | | | |
| **6. Statistical Analysis and Reporting** | Goal: To judge the risk of bias related to the statistical analysis and presentation of results. |  |  |
| Presentation of analytical strategy | There is sufficient presentation of data to assess the adequacy of the analysis. | Yes |  |
| Model development strategy | The strategy for model building (i.e., inclusion of variables in the statistical model) is appropriate and is based on a conceptual framework or model./ The selected statistical model is adequate for the design of the study. | No |  |
| Reporting of results | There is no selective reporting of results. | Yes |  |
| **Statistical Analysis and Presentation Summary** | The statistical analysis is appropriate for the design of the study, limiting potential for presentation of invalid or spurious results. |  | Moderate |
|  | | | |
| Study title: | An appraisal of clinicopathological parameters in Japanese encephalitis and changing epidemiological trends in upper Assam, India | | |
| Study author/ data: | Patgiri et al., 2014 | | |
| Study identifier: | 10.4103/0377-4929.138732 | | |
|  | | | |
| Biases | Issues to consider for judging overall rating of "Risk of bias" | Rating of reporting | Rating of "Risk of bias" |
| **1. Study Participation** | Goal: To judge the risk of selection bias (likelihood that relationship between PF and outcome is different for participants and eligible non-participants). |  |  |
| Source of target population | The source population or population of interest is adequately described for key characteristics (LIST). | Yes |  |
| Method used to identify population | The sampling frame and recruitment are adequately described, including methods to identify the sample sufficient to limit potential bias (number and type used, e.g., referral patterns in health care) | No |  |
| Recruitment period | Period of recruitment is adequately described | Yes |  |
| Place of recruitment | Place of recruitment (setting and geographic location) are adequately described | Yes |  |
| Inclusion and exclusion criteria | Inclusion and exclusion criteria are adequately described (e.g., including explicit diagnostic criteria or “zero time” description). | Partial |  |
| Adequate study participation | There is adequate participation in the study by eligible individuals | Yes |  |
| Baseline characteristics | The baseline study sample (i.e., individuals entering the study) is adequately described for key characteristics (LIST). | Yes |  |
| **Summary Study participation** | The study sample represents the population of interest on key characteristics, sufficient to limit potential bias of the observed relationship between PF and outcome. |  | Low |
|  | | | |
| **2. Study Attrition** | Goal: To judge the risk of attrition bias (likelihood that relationship between PF and outcome are different for completing and non-completing participants). |  |  |
| Proportion of baseline sample available for analysis | Response rate (i.e., proportion of study sample completing the study and providing outcome data) is adequate. | Yes |  |
| Attempts to collect information on participants who dropped out | Attempts to collect information on participants who dropped out of the study are described. | No |  |
| Reasons and potential impact of subjects lost to follow-up | Reasons for loss to follow-up are provided. | No |  |
| Outcome and prognostic factor information on those lost to follow-up | Participants lost to follow-up are adequately described for key characteristics (LIST)./ There are no important differences between key characteristics (LIST) and outcomes in participants who completed the study and those who did not. | No |  |
| **Study Attrition Summary** | Loss to follow-up (from baseline sample to study population analyzed) is not associated with key characteristics (i.e., the study data adequately represent the sample) sufficient to limit potential bias to the observed relationship between PF and outcome. |  | Low |
|  | | | |
| **3. Prognostic Factor Measurement** | Goal: To judge the risk of measurement bias related to how PF was measured (differential measurement of PF related to the level of outcome). |  |  |
| Definition of the PF | A clear definition or description of 'PF' is provided (e.g., including dose, level, duration of exposure, and clear specification of the method of measurement). | No |  |
| Valid and Reliable Measurement of PF | Method of PF measurement is adequately valid and reliable to limit misclassification bias (e.g., may include relevant outside sources of information on measurement properties, also characteristics, such as blind measurement and limited reliance on recall)./ Continuous variables are reported or appropriate cut-points (i.e., not data-dependent) are used. | Yes |  |
| Method and Setting of PF Measurement | The method and setting of measurement of PF is the same for all study participants. | Yes |  |
| Proportion of data on PF available for analysis | Adequate proportion of the study sample has complete data for PF variable. | Yes |  |
| Method used for missing data | Appropriate methods of imputation are used for missing 'PF' data. | No |  |
| **PF Measurement Summary** | PF is adequately measured in study participants to sufficiently limit potential bias. |  | Low |
|  | | | |
| 4. Outcome Measurement | Goal: To judge the risk of bias related to the measurement of outcome (differential measurement of outcome related to the baseline level of PF). |  |  |
| Definition of the Outcome | A clear definition of outcome is provided, including duration of follow-up and level and extent of the outcome construct. | Yes |  |
| Valid and Reliable Measurement of Outcome | The method of outcome measurement used is adequately valid and reliable to limit misclassification bias (e.g., may include relevant outside sources of information on measurement properties, also characteristics, such as blind measurement and confirmation of outcome with valid and reliable test). | Yes |  |
| Method and Setting of Outcome Measurement | The method and setting of outcome measurement is the same for all study participants. | Yes |  |
| **Outcome Measurement Summary** | Outcome of interest is adequately measured in study participants to sufficiently limit potential bias. |  | Low |
|  | | | |
| **5. Study Confounding** | Goal: To judge the risk of bias due to confounding (i.e. the effect of PF is distorted by another factor that is related to PF and outcome). |  |  |
| Important Confounders Measured | All important confounders, including treatments (key variables in conceptual model: LIST), are measured. | Yes |  |
| Definition of the confounding factor | Clear definitions of the important confounders measured are provided (e.g., including dose, level, and duration of exposures). | Partial |  |
| Valid and Reliable Measurement of Confounders | Measurement of all important confounders is adequately valid and reliable (e.g., may include relevant outside sources of information on measurement properties, also characteristics, such as blind measurement and limited reliance on recall). | Yes |  |
| Method and Setting of Confounding Measurement | The method and setting of confounding measurement are the same for all study participants. | Yes |  |
| Method used for missing data | Appropriate methods are used if imputation is used for missing confounder data. | No |  |
| Appropriate Accounting for Confounding | Important potential confounders are accounted for in the study design (e.g., matching for key variables, stratification, or initial assembly of comparable groups). /Important potential confounders are accounted for in the analysis (i.e., appropriate adjustment). | Partial |  |
| **Study Confounding Summary** | Important potential confounders are appropriately accounted for, limiting potential bias with respect to the relationship between PF and outcome . |  | Low |
|  | | | |
| **6. Statistical Analysis and Reporting** | Goal: To judge the risk of bias related to the statistical analysis and presentation of results. |  |  |
| Presentation of analytical strategy | There is sufficient presentation of data to assess the adequacy of the analysis. | Yes |  |
| Model development strategy | The strategy for model building (i.e., inclusion of variables in the statistical model) is appropriate and is based on a conceptual framework or model./ The selected statistical model is adequate for the design of the study. | No |  |
| Reporting of results | There is no selective reporting of results. | Yes |  |
| **Statistical Analysis and Presentation Summary** | The statistical analysis is appropriate for the design of the study, limiting potential for presentation of invalid or spurious results. |  | Low |
|  | | | |
| Study title: | Burden of herpes simplex virus encephalitis in the United States | | |
| Study author/ data: | Modi et al., 2017 | | |
| Study identifier: | 10.1007/s00415-017-8516-x | | |
|  | | | |
| Biases | Issues to consider for judging overall rating of "Risk of bias" | Rating of reporting | Rating of "Risk of bias" |
| **1. Study Participation** | Goal: To judge the risk of selection bias (likelihood that relationship between PF and outcome is different for participants and eligible non-participants). |  |  |
| Source of target population | The source population or population of interest is adequately described for key characteristics (LIST). | Yes |  |
| Method used to identify population | The sampling frame and recruitment are adequately described, including methods to identify the sample sufficient to limit potential bias (number and type used, e.g., referral patterns in health care) | Partial |  |
| Recruitment period | Period of recruitment is adequately described | Yes |  |
| Place of recruitment | Place of recruitment (setting and geographic location) are adequately described | Yes |  |
| Inclusion and exclusion criteria | Inclusion and exclusion criteria are adequately described (e.g., including explicit diagnostic criteria or “zero time” description). | Partial |  |
| Adequate study participation | There is adequate participation in the study by eligible individuals | Yes |  |
| Baseline characteristics | The baseline study sample (i.e., individuals entering the study) is adequately described for key characteristics (LIST). | Yes |  |
| **Summary Study participation** | The study sample represents the population of interest on key characteristics, sufficient to limit potential bias of the observed relationship between PF and outcome. |  | Low |
|  |  |  |  |
| **2. Study Attrition** | Goal: To judge the risk of attrition bias (likelihood that relationship between PF and outcome are different for completing and non-completing participants). |  |  |
| Proportion of baseline sample available for analysis | Response rate (i.e., proportion of study sample completing the study and providing outcome data) is adequate. | Yes |  |
| Attempts to collect information on participants who dropped out | Attempts to collect information on participants who dropped out of the study are described. | No |  |
| Reasons and potential impact of subjects lost to follow-up | Reasons for loss to follow-up are provided. | No |  |
| Outcome and prognostic factor information on those lost to follow-up | Participants lost to follow-up are adequately described for key characteristics (LIST)./ There are no important differences between key characteristics (LIST) and outcomes in participants who completed the study and those who did not. | No |  |
| **Study Attrition Summary** | Loss to follow-up (from baseline sample to study population analyzed) is not associated with key characteristics (i.e., the study data adequately represent the sample) sufficient to limit potential bias to the observed relationship between PF and outcome. |  | Low |
|  | | | |
| **3. Prognostic Factor Measurement** | Goal: To judge the risk of measurement bias related to how PF was measured (differential measurement of PF related to the level of outcome). |  |  |
| Definition of the PF | A clear definition or description of 'PF' is provided (e.g., including dose, level, duration of exposure, and clear specification of the method of measurement). | No |  |
| Valid and Reliable Measurement of PF | Method of PF measurement is adequately valid and reliable to limit misclassification bias (e.g., may include relevant outside sources of information on measurement properties, also characteristics, such as blind measurement and limited reliance on recall)./ Continuous variables are reported or appropriate cut-points (i.e., not data-dependent) are used. | Unsure |  |
| Method and Setting of PF Measurement | The method and setting of measurement of PF is the same for all study participants. | Yes |  |
| Proportion of data on PF available for analysis | Adequate proportion of the study sample has complete data for PF variable. | Unsure |  |
| Method used for missing data | Appropriate methods of imputation are used for missing 'PF' data. | No |  |
| **PF Measurement Summary** | PF is adequately measured in study participants to sufficiently limit potential bias. |  | Moderate |
|  | | | |
| 4. Outcome Measurement | Goal: To judge the risk of bias related to the measurement of outcome (differential measurement of outcome related to the baseline level of PF). |  |  |
| Definition of the Outcome | A clear definition of outcome is provided, including duration of follow-up and level and extent of the outcome construct. | Partial |  |
| Valid and Reliable Measurement of Outcome | The method of outcome measurement used is adequately valid and reliable to limit misclassification bias (e.g., may include relevant outside sources of information on measurement properties, also characteristics, such as blind measurement and confirmation of outcome with valid and reliable test). | Partial |  |
| Method and Setting of Outcome Measurement | The method and setting of outcome measurement is the same for all study participants. | No |  |
| **Outcome Measurement Summary** | Outcome of interest is adequately measured in study participants to sufficiently limit potential bias. |  | Moderate |
|  | | | |
| **5. Study Confounding** | Goal: To judge the risk of bias due to confounding (i.e. the effect of PF is distorted by another factor that is related to PF and outcome). |  |  |
| Important Confounders Measured | All important confounders, including treatments (key variables in conceptual model: LIST), are measured. | Partial |  |
| Definition of the confounding factor | Clear definitions of the important confounders measured are provided (e.g., including dose, level, and duration of exposures). | Partial |  |
| Valid and Reliable Measurement of Confounders | Measurement of all important confounders is adequately valid and reliable (e.g., may include relevant outside sources of information on measurement properties, also characteristics, such as blind measurement and limited reliance on recall). | Partial |  |
| Method and Setting of Confounding Measurement | The method and setting of confounding measurement are the same for all study participants. | Unsure |  |
| Method used for missing data | Appropriate methods are used if imputation is used for missing confounder data. | No |  |
| Appropriate Accounting for Confounding | Important potential confounders are accounted for in the study design (e.g., matching for key variables, stratification, or initial assembly of comparable groups). /Important potential confounders are accounted for in the analysis (i.e., appropriate adjustment). | Yes |  |
| **Study Confounding Summary** | Important potential confounders are appropriately accounted for, limiting potential bias with respect to the relationship between PF and outcome . |  | Low |
|  | | | |
| **6. Statistical Analysis and Reporting** | Goal: To judge the risk of bias related to the statistical analysis and presentation of results. |  |  |
| Presentation of analytical strategy | There is sufficient presentation of data to assess the adequacy of the analysis. | Yes |  |
| Model development strategy | The strategy for model building (i.e., inclusion of variables in the statistical model) is appropriate and is based on a conceptual framework or model./ The selected statistical model is adequate for the design of the study. | No |  |
| Reporting of results | There is no selective reporting of results. | No |  |
| **Statistical Analysis and Presentation Summary** | The statistical analysis is appropriate for the design of the study, limiting potential for presentation of invalid or spurious results. |  | Low |
|  | | | |
| Study title: | Clinico‐epidemiological characteristics of hospitalized acute encephalitis syndrome children and their correlation with case fatality rate | | |
| Study author/ data: | Kakoti et al., 2020 | | |
| Study identifier: | 10.4103/jfmpc.jfmpc_1645_20 | | |
|  | | | |
| Biases | Issues to consider for judging overall rating of "Risk of bias" | Rating of reporting | Rating of "Risk of bias" |
| **1. Study Participation** | Goal: To judge the risk of selection bias (likelihood that relationship between PF and outcome is different for participants and eligible non-participants). |  |  |
| Source of target population | The source population or population of interest is adequately described for key characteristics (LIST). | Partial |  |
| Method used to identify population | The sampling frame and recruitment are adequately described, including methods to identify the sample sufficient to limit potential bias (number and type used, e.g., referral patterns in health care) | Partial |  |
| Recruitment period | Period of recruitment is adequately described | Yes |  |
| Place of recruitment | Place of recruitment (setting and geographic location) are adequately described | Yes |  |
| Inclusion and exclusion criteria | Inclusion and exclusion criteria are adequately described (e.g., including explicit diagnostic criteria or “zero time” description). | Partial |  |
| Adequate study participation | There is adequate participation in the study by eligible individuals | Partial |  |
| Baseline characteristics | The baseline study sample (i.e., individuals entering the study) is adequately described for key characteristics (LIST). | Partial |  |
| **Summary Study participation** | The study sample represents the population of interest on key characteristics, sufficient to limit potential bias of the observed relationship between PF and outcome. |  | Moderate |
|  | | | |
| **2. Study Attrition** | Goal: To judge the risk of attrition bias (likelihood that relationship between PF and outcome are different for completing and non-completing participants). |  |  |
| Proportion of baseline sample available for analysis | Response rate (i.e., proportion of study sample completing the study and providing outcome data) is adequate. | Yes |  |
| Attempts to collect information on participants who dropped out | Attempts to collect information on participants who dropped out of the study are described. | No |  |
| Reasons and potential impact of subjects lost to follow-up | Reasons for loss to follow-up are provided. | Yes |  |
| Outcome and prognostic factor information on those lost to follow-up | Participants lost to follow-up are adequately described for key characteristics (LIST)./ There are no important differences between key characteristics (LIST) and outcomes in participants who completed the study and those who did not. | No |  |
| **Study Attrition Summary** | Loss to follow-up (from baseline sample to study population analyzed) is not associated with key characteristics (i.e., the study data adequately represent the sample) sufficient to limit potential bias to the observed relationship between PF and outcome. |  | Moderate |
|  | | | |
| **3. Prognostic Factor Measurement** | Goal: To judge the risk of measurement bias related to how PF was measured (differential measurement of PF related to the level of outcome). |  |  |
| Definition of the PF | A clear definition or description of 'PF' is provided (e.g., including dose, level, duration of exposure, and clear specification of the method of measurement). | No |  |
| Valid and Reliable Measurement of PF | Method of PF measurement is adequately valid and reliable to limit misclassification bias (e.g., may include relevant outside sources of information on measurement properties, also characteristics, such as blind measurement and limited reliance on recall)./ Continuous variables are reported or appropriate cut-points (i.e., not data-dependent) are used. | Partial |  |
| Method and Setting of PF Measurement | The method and setting of measurement of PF is the same for all study participants. | Yes |  |
| Proportion of data on PF available for analysis | Adequate proportion of the study sample has complete data for PF variable. | Yes |  |
| Method used for missing data | Appropriate methods of imputation are used for missing 'PF' data. | No |  |
| **PF Measurement Summary** | PF is adequately measured in study participants to sufficiently limit potential bias. |  | Low |
|  | | | |
| 4. Outcome Measurement | Goal: To judge the risk of bias related to the measurement of outcome (differential measurement of outcome related to the baseline level of PF). |  |  |
| Definition of the Outcome | A clear definition of outcome is provided, including duration of follow-up and level and extent of the outcome construct. | Yes |  |
| Valid and Reliable Measurement of Outcome | The method of outcome measurement used is adequately valid and reliable to limit misclassification bias (e.g., may include relevant outside sources of information on measurement properties, also characteristics, such as blind measurement and confirmation of outcome with valid and reliable test). | Yes |  |
| Method and Setting of Outcome Measurement | The method and setting of outcome measurement is the same for all study participants. | Yes |  |
| **Outcome Measurement Summary** | Outcome of interest is adequately measured in study participants to sufficiently limit potential bias. |  | Low |
|  | | | |
| **5. Study Confounding** | Goal: To judge the risk of bias due to confounding (i.e. the effect of PF is distorted by another factor that is related to PF and outcome). |  |  |
| Important Confounders Measured | All important confounders, including treatments (key variables in conceptual model: LIST), are measured. | No |  |
| Definition of the confounding factor | Clear definitions of the important confounders measured are provided (e.g., including dose, level, and duration of exposures). | No |  |
| Valid and Reliable Measurement of Confounders | Measurement of all important confounders is adequately valid and reliable (e.g., may include relevant outside sources of information on measurement properties, also characteristics, such as blind measurement and limited reliance on recall). | No |  |
| Method and Setting of Confounding Measurement | The method and setting of confounding measurement are the same for all study participants. | No |  |
| Method used for missing data | Appropriate methods are used if imputation is used for missing confounder data. | No |  |
| Appropriate Accounting for Confounding | Important potential confounders are accounted for in the study design (e.g., matching for key variables, stratification, or initial assembly of comparable groups). /Important potential confounders are accounted for in the analysis (i.e., appropriate adjustment). | No |  |
| **Study Confounding Summary** | Important potential confounders are appropriately accounted for, limiting potential bias with respect to the relationship between PF and outcome . |  | High |
|  | | | |
| **6. Statistical Analysis and Reporting** | Goal: To judge the risk of bias related to the statistical analysis and presentation of results. |  |  |
| Presentation of analytical strategy | There is sufficient presentation of data to assess the adequacy of the analysis. | Yes |  |
| Model development strategy | The strategy for model building (i.e., inclusion of variables in the statistical model) is appropriate and is based on a conceptual framework or model./ The selected statistical model is adequate for the design of the study. | No |  |
| Reporting of results | There is no selective reporting of results. | Unsure |  |
| **Statistical Analysis and Presentation Summary** | The statistical analysis is appropriate for the design of the study, limiting potential for presentation of invalid or spurious results. |  | Moderate |
|  | | | |
| Study title: | Correlation of Tumor Necrosis Factor Levels in the Serum and Cerebrospinal Fluid With Clinical Outcome in Japanese Encephalitis Patients | | |
| Study author/ data: | Ravi et al., 1997 | | |
| Study identifier: | 10.1002/%28SICI%291096-9071%28199702%2951:2%3C132::AID-JMV8%3E3.0.CO;2-8 | | |
|  | | | |
| Biases | Issues to consider for judging overall rating of "Risk of bias" | Rating of reporting | Rating of "Risk of bias" |
| **1. Study Participation** | Goal: To judge the risk of selection bias (likelihood that relationship between PF and outcome is different for participants and eligible non-participants). |  |  |
| Source of target population | The source population or population of interest is adequately described for key characteristics (LIST). | Partial |  |
| Method used to identify population | The sampling frame and recruitment are adequately described, including methods to identify the sample sufficient to limit potential bias (number and type used, e.g., referral patterns in health care) | Yes |  |
| Recruitment period | Period of recruitment is adequately described | Partial |  |
| Place of recruitment | Place of recruitment (setting and geographic location) are adequately described | Yes |  |
| Inclusion and exclusion criteria | Inclusion and exclusion criteria are adequately described (e.g., including explicit diagnostic criteria or “zero time” description). | No |  |
| Adequate study participation | There is adequate participation in the study by eligible individuals | Partial |  |
| Baseline characteristics | The baseline study sample (i.e., individuals entering the study) is adequately described for key characteristics (LIST). | Partial |  |
| **Summary Study participation** | The study sample represents the population of interest on key characteristics, sufficient to limit potential bias of the observed relationship between PF and outcome. |  | Moderate |
|  | | | |
| **2. Study Attrition** | Goal: To judge the risk of attrition bias (likelihood that relationship between PF and outcome are different for completing and non-completing participants). |  |  |
| Proportion of baseline sample available for analysis | Response rate (i.e., proportion of study sample completing the study and providing outcome data) is adequate. | No |  |
| Attempts to collect information on participants who dropped out | Attempts to collect information on participants who dropped out of the study are described. | No |  |
| Reasons and potential impact of subjects lost to follow-up | Reasons for loss to follow-up are provided. | No |  |
| Outcome and prognostic factor information on those lost to follow-up | Participants lost to follow-up are adequately described for key characteristics (LIST)./ There are no important differences between key characteristics (LIST) and outcomes in participants who completed the study and those who did not. | No |  |
| **Study Attrition Summary** | Loss to follow-up (from baseline sample to study population analyzed) is not associated with key characteristics (i.e., the study data adequately represent the sample) sufficient to limit potential bias to the observed relationship between PF and outcome. |  | High |
|  | | | |
| **3. Prognostic Factor Measurement** | Goal: To judge the risk of measurement bias related to how PF was measured (differential measurement of PF related to the level of outcome). |  |  |
| Definition of the PF | A clear definition or description of 'PF' is provided (e.g., including dose, level, duration of exposure, and clear specification of the method of measurement). | No |  |
| Valid and Reliable Measurement of PF | Method of PF measurement is adequately valid and reliable to limit misclassification bias (e.g., may include relevant outside sources of information on measurement properties, also characteristics, such as blind measurement and limited reliance on recall)./ Continuous variables are reported or appropriate cut-points (i.e., not data-dependent) are used. | Partial |  |
| Method and Setting of PF Measurement | The method and setting of measurement of PF is the same for all study participants. | Yes |  |
| Proportion of data on PF available for analysis | Adequate proportion of the study sample has complete data for PF variable. | Yes |  |
| Method used for missing data | Appropriate methods of imputation are used for missing 'PF' data. | No |  |
| **PF Measurement Summary** | PF is adequately measured in study participants to sufficiently limit potential bias. |  | Moderate |
|  | | | |
| 4. Outcome Measurement | Goal: To judge the risk of bias related to the measurement of outcome (differential measurement of outcome related to the baseline level of PF). |  |  |
| Definition of the Outcome | A clear definition of outcome is provided, including duration of follow-up and level and extent of the outcome construct. | Yes |  |
| Valid and Reliable Measurement of Outcome | The method of outcome measurement used is adequately valid and reliable to limit misclassification bias (e.g., may include relevant outside sources of information on measurement properties, also characteristics, such as blind measurement and confirmation of outcome with valid and reliable test). | Yes |  |
| Method and Setting of Outcome Measurement | The method and setting of outcome measurement is the same for all study participants. | Yes |  |
| **Outcome Measurement Summary** | Outcome of interest is adequately measured in study participants to sufficiently limit potential bias. |  | Low |
|  | | | |
| **5. Study Confounding** | Goal: To judge the risk of bias due to confounding (i.e. the effect of PF is distorted by another factor that is related to PF and outcome). |  |  |
| Important Confounders Measured | All important confounders, including treatments (key variables in conceptual model: LIST), are measured. | No |  |
| Definition of the confounding factor | Clear definitions of the important confounders measured are provided (e.g., including dose, level, and duration of exposures). | No |  |
| Valid and Reliable Measurement of Confounders | Measurement of all important confounders is adequately valid and reliable (e.g., may include relevant outside sources of information on measurement properties, also characteristics, such as blind measurement and limited reliance on recall). | No |  |
| Method and Setting of Confounding Measurement | The method and setting of confounding measurement are the same for all study participants. | Unsure |  |
| Method used for missing data | Appropriate methods are used if imputation is used for missing confounder data. | No |  |
| Appropriate Accounting for Confounding | Important potential confounders are accounted for in the study design (e.g., matching for key variables, stratification, or initial assembly of comparable groups). /Important potential confounders are accounted for in the analysis (i.e., appropriate adjustment). | No |  |
| **Study Confounding Summary** | Important potential confounders are appropriately accounted for, limiting potential bias with respect to the relationship between PF and outcome . |  | High |
|  | | | |
| **6. Statistical Analysis and Reporting** | Goal: To judge the risk of bias related to the statistical analysis and presentation of results. |  |  |
| Presentation of analytical strategy | There is sufficient presentation of data to assess the adequacy of the analysis. | Yes |  |
| Model development strategy | The strategy for model building (i.e., inclusion of variables in the statistical model) is appropriate and is based on a conceptual framework or model./ The selected statistical model is adequate for the design of the study. | No |  |
| Reporting of results | There is no selective reporting of results. | Unsure |  |
| **Statistical Analysis and Presentation Summary** | The statistical analysis is appropriate for the design of the study, limiting potential for presentation of invalid or spurious results. |  | Moderate |
|  | | | |
| Study title: | BrainStem Encephalitis Associated with Chandipura in Andhra Pradesh Outbreak | | |
| Study author/ data: | Rao et al., 2008 | | |
| Study identifier: | 10.1093/tropej/fmm078 | | |
|  | | | |
| Biases | Issues to consider for judging overall rating of "Risk of bias" | Rating of reporting | Rating of "Risk of bias" |
| **1. Study Participation** | Goal: To judge the risk of selection bias (likelihood that relationship between PF and outcome is different for participants and eligible non-participants). |  |  |
| Source of target population | The source population or population of interest is adequately described for key characteristics (LIST). | No |  |
| Method used to identify population | The sampling frame and recruitment are adequately described, including methods to identify the sample sufficient to limit potential bias (number and type used, e.g., referral patterns in health care) | No |  |
| Recruitment period | Period of recruitment is adequately described | Partial |  |
| Place of recruitment | Place of recruitment (setting and geographic location) are adequately described | Yes |  |
| Inclusion and exclusion criteria | Inclusion and exclusion criteria are adequately described (e.g., including explicit diagnostic criteria or “zero time” description). | Partial |  |
| Adequate study participation | There is adequate participation in the study by eligible individuals | Yes |  |
| Baseline characteristics | The baseline study sample (i.e., individuals entering the study) is adequately described for key characteristics (LIST). | No |  |
| **Summary Study participation** | The study sample represents the population of interest on key characteristics, sufficient to limit potential bias of the observed relationship between PF and outcome. |  | Moderate |
|  | | | |
| **2. Study Attrition** | Goal: To judge the risk of attrition bias (likelihood that relationship between PF and outcome are different for completing and non-completing participants). |  |  |
| Proportion of baseline sample available for analysis | Response rate (i.e., proportion of study sample completing the study and providing outcome data) is adequate. | Yes |  |
| Attempts to collect information on participants who dropped out | Attempts to collect information on participants who dropped out of the study are described. | No |  |
| Reasons and potential impact of subjects lost to follow-up | Reasons for loss to follow-up are provided. | No |  |
| Outcome and prognostic factor information on those lost to follow-up | Participants lost to follow-up are adequately described for key characteristics (LIST)./ There are no important differences between key characteristics (LIST) and outcomes in participants who completed the study and those who did not. | No |  |
| **Study Attrition Summary** | Loss to follow-up (from baseline sample to study population analyzed) is not associated with key characteristics (i.e., the study data adequately represent the sample) sufficient to limit potential bias to the observed relationship between PF and outcome. |  | Low |
|  | | | |
| **3. Prognostic Factor Measurement** | Goal: To judge the risk of measurement bias related to how PF was measured (differential measurement of PF related to the level of outcome). |  |  |
| Definition of the PF | A clear definition or description of 'PF' is provided (e.g., including dose, level, duration of exposure, and clear specification of the method of measurement). | Partial |  |
| Valid and Reliable Measurement of PF | Method of PF measurement is adequately valid and reliable to limit misclassification bias (e.g., may include relevant outside sources of information on measurement properties, also characteristics, such as blind measurement and limited reliance on recall)./ Continuous variables are reported or appropriate cut-points (i.e., not data-dependent) are used. | Partial |  |
| Method and Setting of PF Measurement | The method and setting of measurement of PF is the same for all study participants. | Yes |  |
| Proportion of data on PF available for analysis | Adequate proportion of the study sample has complete data for PF variable. | Partial |  |
| Method used for missing data | Appropriate methods of imputation are used for missing 'PF' data. | No |  |
| **PF Measurement Summary** | PF is adequately measured in study participants to sufficiently limit potential bias. |  | Moderate |
|  | | | |
| 4. Outcome Measurement | Goal: To judge the risk of bias related to the measurement of outcome (differential measurement of outcome related to the baseline level of PF). |  |  |
| Definition of the Outcome | A clear definition of outcome is provided, including duration of follow-up and level and extent of the outcome construct. | Yes |  |
| Valid and Reliable Measurement of Outcome | The method of outcome measurement used is adequately valid and reliable to limit misclassification bias (e.g., may include relevant outside sources of information on measurement properties, also characteristics, such as blind measurement and confirmation of outcome with valid and reliable test). | Yes |  |
| Method and Setting of Outcome Measurement | The method and setting of outcome measurement is the same for all study participants. | Yes |  |
| **Outcome Measurement Summary** | Outcome of interest is adequately measured in study participants to sufficiently limit potential bias. |  | Low |
|  | | | |
| **5. Study Confounding** | Goal: To judge the risk of bias due to confounding (i.e. the effect of PF is distorted by another factor that is related to PF and outcome). |  |  |
| Important Confounders Measured | All important confounders, including treatments (key variables in conceptual model: LIST), are measured. | No |  |
| Definition of the confounding factor | Clear definitions of the important confounders measured are provided (e.g., including dose, level, and duration of exposures). | No |  |
| Valid and Reliable Measurement of Confounders | Measurement of all important confounders is adequately valid and reliable (e.g., may include relevant outside sources of information on measurement properties, also characteristics, such as blind measurement and limited reliance on recall). | No |  |
| Method and Setting of Confounding Measurement | The method and setting of confounding measurement are the same for all study participants. | No |  |
| Method used for missing data | Appropriate methods are used if imputation is used for missing confounder data. | No |  |
| Appropriate Accounting for Confounding | Important potential confounders are accounted for in the study design (e.g., matching for key variables, stratification, or initial assembly of comparable groups). /Important potential confounders are accounted for in the analysis (i.e., appropriate adjustment). | No |  |
| **Study Confounding Summary** | Important potential confounders are appropriately accounted for, limiting potential bias with respect to the relationship between PF and outcome . |  | High |
|  | | | |
| **6. Statistical Analysis and Reporting** | Goal: To judge the risk of bias related to the statistical analysis and presentation of results. |  |  |
| Presentation of analytical strategy | There is sufficient presentation of data to assess the adequacy of the analysis. | Yes |  |
| Model development strategy | The strategy for model building (i.e., inclusion of variables in the statistical model) is appropriate and is based on a conceptual framework or model./ The selected statistical model is adequate for the design of the study. | No |  |
| Reporting of results | There is no selective reporting of results. | No |  |
| **Statistical Analysis and Presentation Summary** | The statistical analysis is appropriate for the design of the study, limiting potential for presentation of invalid or spurious results. |  | Moderate |
|  | | | |
| Study title: | Viral Etiology of Encephalitis in Children in Southern Vietnam: Results of a One-Year Prospective Descriptive Study | | |
| Study author/ data: | VanTan et al., 2010 | | |
| Study identifier: | 10.1371/journal.pntd.0000854 | | |
|  | | | |
| Biases | Issues to consider for judging overall rating of "Risk of bias" | Rating of reporting | Rating of "Risk of bias" |
| **1. Study Participation** | Goal: To judge the risk of selection bias (likelihood that relationship between PF and outcome is different for participants and eligible non-participants). |  |  |
| Source of target population | The source population or population of interest is adequately described for key characteristics (LIST). | Yes |  |
| Method used to identify population | The sampling frame and recruitment are adequately described, including methods to identify the sample sufficient to limit potential bias (number and type used, e.g., referral patterns in health care) | Partial |  |
| Recruitment period | Period of recruitment is adequately described | Yes |  |
| Place of recruitment | Place of recruitment (setting and geographic location) are adequately described | Yes |  |
| Inclusion and exclusion criteria | Inclusion and exclusion criteria are adequately described (e.g., including explicit diagnostic criteria or “zero time” description). | Partial |  |
| Adequate study participation | There is adequate participation in the study by eligible individuals | Yes |  |
| Baseline characteristics | The baseline study sample (i.e., individuals entering the study) is adequately described for key characteristics (LIST). | Yes |  |
| **Summary Study participation** | The study sample represents the population of interest on key characteristics, sufficient to limit potential bias of the observed relationship between PF and outcome. |  | Low |
|  | | | |
| **2. Study Attrition** | Goal: To judge the risk of attrition bias (likelihood that relationship between PF and outcome are different for completing and non-completing participants). |  |  |
| Proportion of baseline sample available for analysis | Response rate (i.e., proportion of study sample completing the study and providing outcome data) is adequate. | Yes |  |
| Attempts to collect information on participants who dropped out | Attempts to collect information on participants who dropped out of the study are described. | No |  |
| Reasons and potential impact of subjects lost to follow-up | Reasons for loss to follow-up are provided. | No |  |
| Outcome and prognostic factor information on those lost to follow-up | Participants lost to follow-up are adequately described for key characteristics (LIST)./ There are no important differences between key characteristics (LIST) and outcomes in participants who completed the study and those who did not. | No |  |
| **Study Attrition Summary** | Loss to follow-up (from baseline sample to study population analyzed) is not associated with key characteristics (i.e., the study data adequately represent the sample) sufficient to limit potential bias to the observed relationship between PF and outcome. |  | Low |
|  | | | |
| **3. Prognostic Factor Measurement** | Goal: To judge the risk of measurement bias related to how PF was measured (differential measurement of PF related to the level of outcome). |  |  |
| Definition of the PF | A clear definition or description of 'PF' is provided (e.g., including dose, level, duration of exposure, and clear specification of the method of measurement). | No |  |
| Valid and Reliable Measurement of PF | Method of PF measurement is adequately valid and reliable to limit misclassification bias (e.g., may include relevant outside sources of information on measurement properties, also characteristics, such as blind measurement and limited reliance on recall)./ Continuous variables are reported or appropriate cut-points (i.e., not data-dependent) are used. | Partial |  |
| Method and Setting of PF Measurement | The method and setting of measurement of PF is the same for all study participants. | Yes |  |
| Proportion of data on PF available for analysis | Adequate proportion of the study sample has complete data for PF variable. | Yes |  |
| Method used for missing data | Appropriate methods of imputation are used for missing 'PF' data. | No |  |
| **PF Measurement Summary** | PF is adequately measured in study participants to sufficiently limit potential bias. |  | Low |
|  | | | |
| 4. Outcome Measurement | Goal: To judge the risk of bias related to the measurement of outcome (differential measurement of outcome related to the baseline level of PF). |  |  |
| Definition of the Outcome | A clear definition of outcome is provided, including duration of follow-up and level and extent of the outcome construct. | Yes |  |
| Valid and Reliable Measurement of Outcome | The method of outcome measurement used is adequately valid and reliable to limit misclassification bias (e.g., may include relevant outside sources of information on measurement properties, also characteristics, such as blind measurement and confirmation of outcome with valid and reliable test). | Yes |  |
| Method and Setting of Outcome Measurement | The method and setting of outcome measurement is the same for all study participants. | Yes |  |
| **Outcome Measurement Summary** | Outcome of interest is adequately measured in study participants to sufficiently limit potential bias. |  | Low |
|  | | | |
| **5. Study Confounding** | Goal: To judge the risk of bias due to confounding (i.e. the effect of PF is distorted by another factor that is related to PF and outcome). |  |  |
| Important Confounders Measured | All important confounders, including treatments (key variables in conceptual model: LIST), are measured. | Yes |  |
| Definition of the confounding factor | Clear definitions of the important confounders measured are provided (e.g., including dose, level, and duration of exposures). | Partial |  |
| Valid and Reliable Measurement of Confounders | Measurement of all important confounders is adequately valid and reliable (e.g., may include relevant outside sources of information on measurement properties, also characteristics, such as blind measurement and limited reliance on recall). | Yes |  |
| Method and Setting of Confounding Measurement | The method and setting of confounding measurement are the same for all study participants. | Yes |  |
| Method used for missing data | Appropriate methods are used if imputation is used for missing confounder data. | No |  |
| Appropriate Accounting for Confounding | Important potential confounders are accounted for in the study design (e.g., matching for key variables, stratification, or initial assembly of comparable groups). /Important potential confounders are accounted for in the analysis (i.e., appropriate adjustment). | Partial |  |
| **Study Confounding Summary** | Important potential confounders are appropriately accounted for, limiting potential bias with respect to the relationship between PF and outcome . |  | Low |
|  | | | |
| **6. Statistical Analysis and Reporting** | Goal: To judge the risk of bias related to the statistical analysis and presentation of results. |  |  |
| Presentation of analytical strategy | There is sufficient presentation of data to assess the adequacy of the analysis. | Yes |  |
| Model development strategy | The strategy for model building (i.e., inclusion of variables in the statistical model) is appropriate and is based on a conceptual framework or model./ The selected statistical model is adequate for the design of the study. | No |  |
| Reporting of results | There is no selective reporting of results. | Unsure |  |
| **Statistical Analysis and Presentation Summary** | The statistical analysis is appropriate for the design of the study, limiting potential for presentation of invalid or spurious results. |  | Low |
|  | | | |
| Study title: | Spectrum and outcome of acute infectious encephalitis/encephalopathy in an intensive care unit from India | | |
| Study author/ data: | Kalita et al., 2017 | | |
| Study identifier: | 10.1093/qjmed/hcw132 | | |
|  | | | |
| Biases | Issues to consider for judging overall rating of "Risk of bias" | Rating of reporting | Rating of "Risk of bias" |
| **1. Study Participation** | Goal: To judge the risk of selection bias (likelihood that relationship between PF and outcome is different for participants and eligible non-participants). |  |  |
| Source of target population | The source population or population of interest is adequately described for key characteristics (LIST). | Yes |  |
| Method used to identify population | The sampling frame and recruitment are adequately described, including methods to identify the sample sufficient to limit potential bias (number and type used, e.g., referral patterns in health care) | Partial |  |
| Recruitment period | Period of recruitment is adequately described | Yes |  |
| Place of recruitment | Place of recruitment (setting and geographic location) are adequately described | Yes |  |
| Inclusion and exclusion criteria | Inclusion and exclusion criteria are adequately described (e.g., including explicit diagnostic criteria or “zero time” description). | Yes |  |
| Adequate study participation | There is adequate participation in the study by eligible individuals | Yes |  |
| Baseline characteristics | The baseline study sample (i.e., individuals entering the study) is adequately described for key characteristics (LIST). | Yes |  |
| **Summary Study participation** | The study sample represents the population of interest on key characteristics, sufficient to limit potential bias of the observed relationship between PF and outcome. |  | Low |
|  | | | |
| **2. Study Attrition** | Goal: To judge the risk of attrition bias (likelihood that relationship between PF and outcome are different for completing and non-completing participants). |  |  |
| Proportion of baseline sample available for analysis | Response rate (i.e., proportion of study sample completing the study and providing outcome data) is adequate. | Yes |  |
| Attempts to collect information on participants who dropped out | Attempts to collect information on participants who dropped out of the study are described. | No |  |
| Reasons and potential impact of subjects lost to follow-up | Reasons for loss to follow-up are provided. | No |  |
| Outcome and prognostic factor information on those lost to follow-up | Participants lost to follow-up are adequately described for key characteristics (LIST)./ There are no important differences between key characteristics (LIST) and outcomes in participants who completed the study and those who did not. | No |  |
| **Study Attrition Summary** | Loss to follow-up (from baseline sample to study population analyzed) is not associated with key characteristics (i.e., the study data adequately represent the sample) sufficient to limit potential bias to the observed relationship between PF and outcome. |  | Low |
|  | | | |
| **3. Prognostic Factor Measurement** | Goal: To judge the risk of measurement bias related to how PF was measured (differential measurement of PF related to the level of outcome). |  |  |
| Definition of the PF | A clear definition or description of 'PF' is provided (e.g., including dose, level, duration of exposure, and clear specification of the method of measurement). | Partial |  |
| Valid and Reliable Measurement of PF | Method of PF measurement is adequately valid and reliable to limit misclassification bias (e.g., may include relevant outside sources of information on measurement properties, also characteristics, such as blind measurement and limited reliance on recall)./ Continuous variables are reported or appropriate cut-points (i.e., not data-dependent) are used. | Yes |  |
| Method and Setting of PF Measurement | The method and setting of measurement of PF is the same for all study participants. | Yes |  |
| Proportion of data on PF available for analysis | Adequate proportion of the study sample has complete data for PF variable. | Yes |  |
| Method used for missing data | Appropriate methods of imputation are used for missing 'PF' data. | No |  |
| **PF Measurement Summary** | PF is adequately measured in study participants to sufficiently limit potential bias. |  | Low |
|  | | | |
| 4. Outcome Measurement | Goal: To judge the risk of bias related to the measurement of outcome (differential measurement of outcome related to the baseline level of PF). |  |  |
| Definition of the Outcome | A clear definition of outcome is provided, including duration of follow-up and level and extent of the outcome construct. | Yes |  |
| Valid and Reliable Measurement of Outcome | The method of outcome measurement used is adequately valid and reliable to limit misclassification bias (e.g., may include relevant outside sources of information on measurement properties, also characteristics, such as blind measurement and confirmation of outcome with valid and reliable test). | Yes |  |
| Method and Setting of Outcome Measurement | The method and setting of outcome measurement is the same for all study participants. | Yes |  |
| **Outcome Measurement Summary** | Outcome of interest is adequately measured in study participants to sufficiently limit potential bias. |  | Low |
|  | | | |
| **5. Study Confounding** | Goal: To judge the risk of bias due to confounding (i.e. the effect of PF is distorted by another factor that is related to PF and outcome). |  |  |
| Important Confounders Measured | All important confounders, including treatments (key variables in conceptual model: LIST), are measured. | Partial |  |
| Definition of the confounding factor | Clear definitions of the important confounders measured are provided (e.g., including dose, level, and duration of exposures). | No |  |
| Valid and Reliable Measurement of Confounders | Measurement of all important confounders is adequately valid and reliable (e.g., may include relevant outside sources of information on measurement properties, also characteristics, such as blind measurement and limited reliance on recall). | Yes |  |
| Method and Setting of Confounding Measurement | The method and setting of confounding measurement are the same for all study participants. | Yes |  |
| Method used for missing data | Appropriate methods are used if imputation is used for missing confounder data. | No |  |
| Appropriate Accounting for Confounding | Important potential confounders are accounted for in the study design (e.g., matching for key variables, stratification, or initial assembly of comparable groups). /Important potential confounders are accounted for in the analysis (i.e., appropriate adjustment). | Yes |  |
| **Study Confounding Summary** | Important potential confounders are appropriately accounted for, limiting potential bias with respect to the relationship between PF and outcome . |  | Low |
|  | | | |
| **6. Statistical Analysis and Reporting** | Goal: To judge the risk of bias related to the statistical analysis and presentation of results. |  |  |
| Presentation of analytical strategy | There is sufficient presentation of data to assess the adequacy of the analysis. | Yes |  |
| Model development strategy | The strategy for model building (i.e., inclusion of variables in the statistical model) is appropriate and is based on a conceptual framework or model./ The selected statistical model is adequate for the design of the study. | Partial |  |
| Reporting of results | There is no selective reporting of results. | Yes |  |
| **Statistical Analysis and Presentation Summary** | The statistical analysis is appropriate for the design of the study, limiting potential for presentation of invalid or spurious results. |  | Low |
|  | | | |
| Study title: | Japanese encephalitis (JE). Part I: clinical profile of 1,282 adult acute cases of four epidemics | | |
| Study author/ data: | Sarkari et al., 2011 | | |
| Study identifier: | 10.1007/s00415-011-6118-6 | | |
|  | | | |
| Biases | Issues to consider for judging overall rating of "Risk of bias" | Rating of reporting | Rating of "Risk of bias" |
| **1. Study Participation** | Goal: To judge the risk of selection bias (likelihood that relationship between PF and outcome is different for participants and eligible non-participants). |  |  |
| Source of target population | The source population or population of interest is adequately described for key characteristics (LIST). | Partial |  |
| Method used to identify population | The sampling frame and recruitment are adequately described, including methods to identify the sample sufficient to limit potential bias (number and type used, e.g., referral patterns in health care) | Partial |  |
| Recruitment period | Period of recruitment is adequately described | Partial |  |
| Place of recruitment | Place of recruitment (setting and geographic location) are adequately described | Yes |  |
| Inclusion and exclusion criteria | Inclusion and exclusion criteria are adequately described (e.g., including explicit diagnostic criteria or “zero time” description). | Partial |  |
| Adequate study participation | There is adequate participation in the study by eligible individuals | Yes |  |
| Baseline characteristics | The baseline study sample (i.e., individuals entering the study) is adequately described for key characteristics (LIST). | Partial |  |
| **Summary Study participation** | The study sample represents the population of interest on key characteristics, sufficient to limit potential bias of the observed relationship between PF and outcome. |  | Moderate |
|  | | | |
| **2. Study Attrition** | Goal: To judge the risk of attrition bias (likelihood that relationship between PF and outcome are different for completing and non-completing participants). |  |  |
| Proportion of baseline sample available for analysis | Response rate (i.e., proportion of study sample completing the study and providing outcome data) is adequate. | Yes |  |
| Attempts to collect information on participants who dropped out | Attempts to collect information on participants who dropped out of the study are described. | No |  |
| Reasons and potential impact of subjects lost to follow-up | Reasons for loss to follow-up are provided. | Yes |  |
| Outcome and prognostic factor information on those lost to follow-up | Participants lost to follow-up are adequately described for key characteristics (LIST)./ There are no important differences between key characteristics (LIST) and outcomes in participants who completed the study and those who did not. | No |  |
| **Study Attrition Summary** | Loss to follow-up (from baseline sample to study population analyzed) is not associated with key characteristics (i.e., the study data adequately represent the sample) sufficient to limit potential bias to the observed relationship between PF and outcome. |  | Moderate |
|  | | | |
| **3. Prognostic Factor Measurement** | Goal: To judge the risk of measurement bias related to how PF was measured (differential measurement of PF related to the level of outcome). |  |  |
| Definition of the PF | A clear definition or description of 'PF' is provided (e.g., including dose, level, duration of exposure, and clear specification of the method of measurement). | No |  |
| Valid and Reliable Measurement of PF | Method of PF measurement is adequately valid and reliable to limit misclassification bias (e.g., may include relevant outside sources of information on measurement properties, also characteristics, such as blind measurement and limited reliance on recall)./ Continuous variables are reported or appropriate cut-points (i.e., not data-dependent) are used. | No |  |
| Method and Setting of PF Measurement | The method and setting of measurement of PF is the same for all study participants. | No |  |
| Proportion of data on PF available for analysis | Adequate proportion of the study sample has complete data for PF variable. | Partial |  |
| Method used for missing data | Appropriate methods of imputation are used for missing 'PF' data. | No |  |
| **PF Measurement Summary** | PF is adequately measured in study participants to sufficiently limit potential bias. |  | High |
|  | | | |
| 4. Outcome Measurement | Goal: To judge the risk of bias related to the measurement of outcome (differential measurement of outcome related to the baseline level of PF). |  |  |
| Definition of the Outcome | A clear definition of outcome is provided, including duration of follow-up and level and extent of the outcome construct. | Partial |  |
| Valid and Reliable Measurement of Outcome | The method of outcome measurement used is adequately valid and reliable to limit misclassification bias (e.g., may include relevant outside sources of information on measurement properties, also characteristics, such as blind measurement and confirmation of outcome with valid and reliable test). | Partial |  |
| Method and Setting of Outcome Measurement | The method and setting of outcome measurement is the same for all study participants. | Unsure |  |
| **Outcome Measurement Summary** | Outcome of interest is adequately measured in study participants to sufficiently limit potential bias. |  | High |
|  | | | |
| **5. Study Confounding** | Goal: To judge the risk of bias due to confounding (i.e. the effect of PF is distorted by another factor that is related to PF and outcome). |  |  |
| Important Confounders Measured | All important confounders, including treatments (key variables in conceptual model: LIST), are measured. | Partial |  |
| Definition of the confounding factor | Clear definitions of the important confounders measured are provided (e.g., including dose, level, and duration of exposures). | No |  |
| Valid and Reliable Measurement of Confounders | Measurement of all important confounders is adequately valid and reliable (e.g., may include relevant outside sources of information on measurement properties, also characteristics, such as blind measurement and limited reliance on recall). | Partial |  |
| Method and Setting of Confounding Measurement | The method and setting of confounding measurement are the same for all study participants. | No |  |
| Method used for missing data | Appropriate methods are used if imputation is used for missing confounder data. | No |  |
| Appropriate Accounting for Confounding | Important potential confounders are accounted for in the study design (e.g., matching for key variables, stratification, or initial assembly of comparable groups). /Important potential confounders are accounted for in the analysis (i.e., appropriate adjustment). | Partial |  |
| **Study Confounding Summary** | Important potential confounders are appropriately accounted for, limiting potential bias with respect to the relationship between PF and outcome . |  | High |
|  | | | |
| **6. Statistical Analysis and Reporting** | Goal: To judge the risk of bias related to the statistical analysis and presentation of results. |  |  |
| Presentation of analytical strategy | There is sufficient presentation of data to assess the adequacy of the analysis. | Unsure |  |
| Model development strategy | The strategy for model building (i.e., inclusion of variables in the statistical model) is appropriate and is based on a conceptual framework or model./ The selected statistical model is adequate for the design of the study. | Partial |  |
| Reporting of results | There is no selective reporting of results. | No |  |
| **Statistical Analysis and Presentation Summary** | The statistical analysis is appropriate for the design of the study, limiting potential for presentation of invalid or spurious results. |  | Moderate |
|  | | | |
| Study title: | Severe fever with thrombocytopenia syndrome bunyavirus-related human encephalitis | | |
| Study author/ data: | Cui et al., 2015 | | |
| Study identifier: | 10.1016/j.jinf.2014.08.001 | | |
|  | | | |
| Biases | Issues to consider for judging overall rating of "Risk of bias" | Rating of reporting | Rating of "Risk of bias" |
| **1. Study Participation** | Goal: To judge the risk of selection bias (likelihood that relationship between PF and outcome is different for participants and eligible non-participants). |  |  |
| Source of target population | The source population or population of interest is adequately described for key characteristics (LIST). | Partial |  |
| Method used to identify population | The sampling frame and recruitment are adequately described, including methods to identify the sample sufficient to limit potential bias (number and type used, e.g., referral patterns in health care) | Partial |  |
| Recruitment period | Period of recruitment is adequately described | Yes |  |
| Place of recruitment | Place of recruitment (setting and geographic location) are adequately described | Yes |  |
| Inclusion and exclusion criteria | Inclusion and exclusion criteria are adequately described (e.g., including explicit diagnostic criteria or “zero time” description). | Partial |  |
| Adequate study participation | There is adequate participation in the study by eligible individuals | Yes |  |
| Baseline characteristics | The baseline study sample (i.e., individuals entering the study) is adequately described for key characteristics (LIST). | Partial |  |
| **Summary Study participation** | The study sample represents the population of interest on key characteristics, sufficient to limit potential bias of the observed relationship between PF and outcome. |  | Moderate |
|  | | | |
| **2. Study Attrition** | Goal: To judge the risk of attrition bias (likelihood that relationship between PF and outcome are different for completing and non-completing participants). |  |  |
| Proportion of baseline sample available for analysis | Response rate (i.e., proportion of study sample completing the study and providing outcome data) is adequate. | Yes |  |
| Attempts to collect information on participants who dropped out | Attempts to collect information on participants who dropped out of the study are described. | No |  |
| Reasons and potential impact of subjects lost to follow-up | Reasons for loss to follow-up are provided. | No |  |
| Outcome and prognostic factor information on those lost to follow-up | Participants lost to follow-up are adequately described for key characteristics (LIST)./ There are no important differences between key characteristics (LIST) and outcomes in participants who completed the study and those who did not. | No |  |
| **Study Attrition Summary** | Loss to follow-up (from baseline sample to study population analyzed) is not associated with key characteristics (i.e., the study data adequately represent the sample) sufficient to limit potential bias to the observed relationship between PF and outcome. |  | Low |
|  | | | |
| **3. Prognostic Factor Measurement** | Goal: To judge the risk of measurement bias related to how PF was measured (differential measurement of PF related to the level of outcome). |  |  |
| Definition of the PF | A clear definition or description of 'PF' is provided (e.g., including dose, level, duration of exposure, and clear specification of the method of measurement). | No |  |
| Valid and Reliable Measurement of PF | Method of PF measurement is adequately valid and reliable to limit misclassification bias (e.g., may include relevant outside sources of information on measurement properties, also characteristics, such as blind measurement and limited reliance on recall)./ Continuous variables are reported or appropriate cut-points (i.e., not data-dependent) are used. | Partial |  |
| Method and Setting of PF Measurement | The method and setting of measurement of PF is the same for all study participants. | Yes |  |
| Proportion of data on PF available for analysis | Adequate proportion of the study sample has complete data for PF variable. | Yes |  |
| Method used for missing data | Appropriate methods of imputation are used for missing 'PF' data. | No |  |
| **PF Measurement Summary** | PF is adequately measured in study participants to sufficiently limit potential bias. |  | Low |
|  | | | |
| 4. Outcome Measurement | Goal: To judge the risk of bias related to the measurement of outcome (differential measurement of outcome related to the baseline level of PF). |  |  |
| Definition of the Outcome | A clear definition of outcome is provided, including duration of follow-up and level and extent of the outcome construct. | Yes |  |
| Valid and Reliable Measurement of Outcome | The method of outcome measurement used is adequately valid and reliable to limit misclassification bias (e.g., may include relevant outside sources of information on measurement properties, also characteristics, such as blind measurement and confirmation of outcome with valid and reliable test). | Yes |  |
| Method and Setting of Outcome Measurement | The method and setting of outcome measurement is the same for all study participants. | Yes |  |
| **Outcome Measurement Summary** | Outcome of interest is adequately measured in study participants to sufficiently limit potential bias. |  | Low |
|  | | | |
| **5. Study Confounding** | Goal: To judge the risk of bias due to confounding (i.e. the effect of PF is distorted by another factor that is related to PF and outcome). |  |  |
| Important Confounders Measured | All important confounders, including treatments (key variables in conceptual model: LIST), are measured. | Yes |  |
| Definition of the confounding factor | Clear definitions of the important confounders measured are provided (e.g., including dose, level, and duration of exposures). | Partial |  |
| Valid and Reliable Measurement of Confounders | Measurement of all important confounders is adequately valid and reliable (e.g., may include relevant outside sources of information on measurement properties, also characteristics, such as blind measurement and limited reliance on recall). | Yes |  |
| Method and Setting of Confounding Measurement | The method and setting of confounding measurement are the same for all study participants. | Yes |  |
| Method used for missing data | Appropriate methods are used if imputation is used for missing confounder data. | No |  |
| Appropriate Accounting for Confounding | Important potential confounders are accounted for in the study design (e.g., matching for key variables, stratification, or initial assembly of comparable groups). /Important potential confounders are accounted for in the analysis (i.e., appropriate adjustment). | Yes |  |
| **Study Confounding Summary** | Important potential confounders are appropriately accounted for, limiting potential bias with respect to the relationship between PF and outcome . |  | Low |
|  | | | |
| **6. Statistical Analysis and Reporting** | Goal: To judge the risk of bias related to the statistical analysis and presentation of results. |  |  |
| Presentation of analytical strategy | There is sufficient presentation of data to assess the adequacy of the analysis. | Yes |  |
| Model development strategy | The strategy for model building (i.e., inclusion of variables in the statistical model) is appropriate and is based on a conceptual framework or model./ The selected statistical model is adequate for the design of the study. | No |  |
| Reporting of results | There is no selective reporting of results. | Yes |  |
| **Statistical Analysis and Presentation Summary** | The statistical analysis is appropriate for the design of the study, limiting potential for presentation of invalid or spurious results. |  | Low |
|  | | | |
| Study title: | Fatal outcome in Japanese Encephalitis | | |
| Study author/ data: | Burke et al., 2015 | | |
| Study identifier: | 10.4269/ajtmh.1985.34.1203 | | |
|  | | | |
| Biases | Issues to consider for judging overall rating of "Risk of bias" | Rating of reporting | Rating of "Risk of bias" |
| **1. Study Participation** | Goal: To judge the risk of selection bias (likelihood that relationship between PF and outcome is different for participants and eligible non-participants). |  |  |
| Source of target population | The source population or population of interest is adequately described for key characteristics (LIST). | Partial |  |
| Method used to identify population | The sampling frame and recruitment are adequately described, including methods to identify the sample sufficient to limit potential bias (number and type used, e.g., referral patterns in health care) | Partial |  |
| Recruitment period | Period of recruitment is adequately described | Yes |  |
| Place of recruitment | Place of recruitment (setting and geographic location) are adequately described | Yes |  |
| Inclusion and exclusion criteria | Inclusion and exclusion criteria are adequately described (e.g., including explicit diagnostic criteria or “zero time” description). | No |  |
| Adequate study participation | There is adequate participation in the study by eligible individuals | Yes |  |
| Baseline characteristics | The baseline study sample (i.e., individuals entering the study) is adequately described for key characteristics (LIST). | Partial |  |
| **Summary Study participation** | The study sample represents the population of interest on key characteristics, sufficient to limit potential bias of the observed relationship between PF and outcome. |  | Moderate |
|  | | | |
| **2. Study Attrition** | Goal: To judge the risk of attrition bias (likelihood that relationship between PF and outcome are different for completing and non-completing participants). |  |  |
| Proportion of baseline sample available for analysis | Response rate (i.e., proportion of study sample completing the study and providing outcome data) is adequate. | Yes |  |
| Attempts to collect information on participants who dropped out | Attempts to collect information on participants who dropped out of the study are described. | No |  |
| Reasons and potential impact of subjects lost to follow-up | Reasons for loss to follow-up are provided. | No |  |
| Outcome and prognostic factor information on those lost to follow-up | Participants lost to follow-up are adequately described for key characteristics (LIST)./ There are no important differences between key characteristics (LIST) and outcomes in participants who completed the study and those who did not. | No |  |
| **Study Attrition Summary** | Loss to follow-up (from baseline sample to study population analyzed) is not associated with key characteristics (i.e., the study data adequately represent the sample) sufficient to limit potential bias to the observed relationship between PF and outcome. |  | Low |
|  | | | |
| **3. Prognostic Factor Measurement** | Goal: To judge the risk of measurement bias related to how PF was measured (differential measurement of PF related to the level of outcome). |  |  |
| Definition of the PF | A clear definition or description of 'PF' is provided (e.g., including dose, level, duration of exposure, and clear specification of the method of measurement). | Partial |  |
| Valid and Reliable Measurement of PF | Method of PF measurement is adequately valid and reliable to limit misclassification bias (e.g., may include relevant outside sources of information on measurement properties, also characteristics, such as blind measurement and limited reliance on recall)./ Continuous variables are reported or appropriate cut-points (i.e., not data-dependent) are used. | Partial |  |
| Method and Setting of PF Measurement | The method and setting of measurement of PF is the same for all study participants. | Yes |  |
| Proportion of data on PF available for analysis | Adequate proportion of the study sample has complete data for PF variable. | Yes |  |
| Method used for missing data | Appropriate methods of imputation are used for missing 'PF' data. | No |  |
| **PF Measurement Summary** | PF is adequately measured in study participants to sufficiently limit potential bias. |  | Low |
|  | | | |
| 4. Outcome Measurement | Goal: To judge the risk of bias related to the measurement of outcome (differential measurement of outcome related to the baseline level of PF). |  |  |
| Definition of the Outcome | A clear definition of outcome is provided, including duration of follow-up and level and extent of the outcome construct. | Yes |  |
| Valid and Reliable Measurement of Outcome | The method of outcome measurement used is adequately valid and reliable to limit misclassification bias (e.g., may include relevant outside sources of information on measurement properties, also characteristics, such as blind measurement and confirmation of outcome with valid and reliable test). | Yes |  |
| Method and Setting of Outcome Measurement | The method and setting of outcome measurement is the same for all study participants. | Yes |  |
| **Outcome Measurement Summary** | Outcome of interest is adequately measured in study participants to sufficiently limit potential bias. |  | Low |
|  | | | |
| **5. Study Confounding** | Goal: To judge the risk of bias due to confounding (i.e. the effect of PF is distorted by another factor that is related to PF and outcome). |  |  |
| Important Confounders Measured | All important confounders, including treatments (key variables in conceptual model: LIST), are measured. | Partial |  |
| Definition of the confounding factor | Clear definitions of the important confounders measured are provided (e.g., including dose, level, and duration of exposures). | Partial |  |
| Valid and Reliable Measurement of Confounders | Measurement of all important confounders is adequately valid and reliable (e.g., may include relevant outside sources of information on measurement properties, also characteristics, such as blind measurement and limited reliance on recall). | Yes |  |
| Method and Setting of Confounding Measurement | The method and setting of confounding measurement are the same for all study participants. | Yes |  |
| Method used for missing data | Appropriate methods are used if imputation is used for missing confounder data. | No |  |
| Appropriate Accounting for Confounding | Important potential confounders are accounted for in the study design (e.g., matching for key variables, stratification, or initial assembly of comparable groups). /Important potential confounders are accounted for in the analysis (i.e., appropriate adjustment). | Partial |  |
| **Study Confounding Summary** | Important potential confounders are appropriately accounted for, limiting potential bias with respect to the relationship between PF and outcome . |  | Moderate |
|  | | | |
| **6. Statistical Analysis and Reporting** | Goal: To judge the risk of bias related to the statistical analysis and presentation of results. |  |  |
| Presentation of analytical strategy | There is sufficient presentation of data to assess the adequacy of the analysis. | Partial |  |
| Model development strategy | The strategy for model building (i.e., inclusion of variables in the statistical model) is appropriate and is based on a conceptual framework or model./ The selected statistical model is adequate for the design of the study. | No |  |
| Reporting of results | There is no selective reporting of results. | Yes |  |
| **Statistical Analysis and Presentation Summary** | The statistical analysis is appropriate for the design of the study, limiting potential for presentation of invalid or spurious results. |  | Low |
|  | | | |
| Study title: | Clinical Features and Risk Factors for Mortality in Children With Acute Encephalitis Who Present to the Emergency Department | | |
| Study author/ data: | Hu et al., 2020 | | |
| Study identifier: | 10.1177/0883073820930557 | | |
|  | | | |
| Biases | Issues to consider for judging overall rating of "Risk of bias" | Rating of reporting | Rating of "Risk of bias" |
| **1. Study Participation** | Goal: To judge the risk of selection bias (likelihood that relationship between PF and outcome is different for participants and eligible non-participants). |  |  |
| Source of target population | The source population or population of interest is adequately described for key characteristics (LIST). | Yes |  |
| Method used to identify population | The sampling frame and recruitment are adequately described, including methods to identify the sample sufficient to limit potential bias (number and type used, e.g., referral patterns in health care) | Partial |  |
| Recruitment period | Period of recruitment is adequately described | Yes |  |
| Place of recruitment | Place of recruitment (setting and geographic location) are adequately described | Yes |  |
| Inclusion and exclusion criteria | Inclusion and exclusion criteria are adequately described (e.g., including explicit diagnostic criteria or “zero time” description). | Yes |  |
| Adequate study participation | There is adequate participation in the study by eligible individuals | Yes |  |
| Baseline characteristics | The baseline study sample (i.e., individuals entering the study) is adequately described for key characteristics (LIST). | Yes |  |
| **Summary Study participation** | The study sample represents the population of interest on key characteristics, sufficient to limit potential bias of the observed relationship between PF and outcome. |  | Low |
|  | | | |
| **2. Study Attrition** | Goal: To judge the risk of attrition bias (likelihood that relationship between PF and outcome are different for completing and non-completing participants). |  |  |
| Proportion of baseline sample available for analysis | Response rate (i.e., proportion of study sample completing the study and providing outcome data) is adequate. | Yes |  |
| Attempts to collect information on participants who dropped out | Attempts to collect information on participants who dropped out of the study are described. | No |  |
| Reasons and potential impact of subjects lost to follow-up | Reasons for loss to follow-up are provided. | No |  |
| Outcome and prognostic factor information on those lost to follow-up | Participants lost to follow-up are adequately described for key characteristics (LIST)./ There are no important differences between key characteristics (LIST) and outcomes in participants who completed the study and those who did not. | No |  |
| **Study Attrition Summary** | Loss to follow-up (from baseline sample to study population analyzed) is not associated with key characteristics (i.e., the study data adequately represent the sample) sufficient to limit potential bias to the observed relationship between PF and outcome. |  | Low |
|  | | | |
| **3. Prognostic Factor Measurement** | Goal: To judge the risk of measurement bias related to how PF was measured (differential measurement of PF related to the level of outcome). |  |  |
| Definition of the PF | A clear definition or description of 'PF' is provided (e.g., including dose, level, duration of exposure, and clear specification of the method of measurement). | Partial |  |
| Valid and Reliable Measurement of PF | Method of PF measurement is adequately valid and reliable to limit misclassification bias (e.g., may include relevant outside sources of information on measurement properties, also characteristics, such as blind measurement and limited reliance on recall)./ Continuous variables are reported or appropriate cut-points (i.e., not data-dependent) are used. | Yes |  |
| Method and Setting of PF Measurement | The method and setting of measurement of PF is the same for all study participants. | Yes |  |
| Proportion of data on PF available for analysis | Adequate proportion of the study sample has complete data for PF variable. | Yes |  |
| Method used for missing data | Appropriate methods of imputation are used for missing 'PF' data. | No |  |
| **PF Measurement Summary** | PF is adequately measured in study participants to sufficiently limit potential bias. |  | Low |
|  | | | |
| 4. Outcome Measurement | Goal: To judge the risk of bias related to the measurement of outcome (differential measurement of outcome related to the baseline level of PF). |  |  |
| Definition of the Outcome | A clear definition of outcome is provided, including duration of follow-up and level and extent of the outcome construct. | Yes |  |
| Valid and Reliable Measurement of Outcome | The method of outcome measurement used is adequately valid and reliable to limit misclassification bias (e.g., may include relevant outside sources of information on measurement properties, also characteristics, such as blind measurement and confirmation of outcome with valid and reliable test). | Yes |  |
| Method and Setting of Outcome Measurement | The method and setting of outcome measurement is the same for all study participants. | Yes |  |
| **Outcome Measurement Summary** | Outcome of interest is adequately measured in study participants to sufficiently limit potential bias. |  | Low |
|  | | | |
| **5. Study Confounding** | Goal: To judge the risk of bias due to confounding (i.e. the effect of PF is distorted by another factor that is related to PF and outcome). |  |  |
| Important Confounders Measured | All important confounders, including treatments (key variables in conceptual model: LIST), are measured. | Partial |  |
| Definition of the confounding factor | Clear definitions of the important confounders measured are provided (e.g., including dose, level, and duration of exposures). | Yes |  |
| Valid and Reliable Measurement of Confounders | Measurement of all important confounders is adequately valid and reliable (e.g., may include relevant outside sources of information on measurement properties, also characteristics, such as blind measurement and limited reliance on recall). | Yes |  |
| Method and Setting of Confounding Measurement | The method and setting of confounding measurement are the same for all study participants. | Yes |  |
| Method used for missing data | Appropriate methods are used if imputation is used for missing confounder data. | No |  |
| Appropriate Accounting for Confounding | Important potential confounders are accounted for in the study design (e.g., matching for key variables, stratification, or initial assembly of comparable groups). /Important potential confounders are accounted for in the analysis (i.e., appropriate adjustment). | Yes |  |
| **Study Confounding Summary** | Important potential confounders are appropriately accounted for, limiting potential bias with respect to the relationship between PF and outcome . |  | Low |
|  | | | |
| **6. Statistical Analysis and Reporting** | Goal: To judge the risk of bias related to the statistical analysis and presentation of results. |  |  |
| Presentation of analytical strategy | There is sufficient presentation of data to assess the adequacy of the analysis. | Yes |  |
| Model development strategy | The strategy for model building (i.e., inclusion of variables in the statistical model) is appropriate and is based on a conceptual framework or model./ The selected statistical model is adequate for the design of the study. | Yes |  |
| Reporting of results | There is no selective reporting of results. | No |  |
| **Statistical Analysis and Presentation Summary** | The statistical analysis is appropriate for the design of the study, limiting potential for presentation of invalid or spurious results. |  | Low |
|  | | | |
| Study title: | Clinical Profile and Outcome of Japanese Encephalitis in Children Admitted with Acute Encephalitis Syndrome | | |
| Study author/ data: | Kakoti et al., 2013 | | |
| Study identifier: | 10.1155/2013/152656 | | |
|  | | | |
| Biases | Issues to consider for judging overall rating of "Risk of bias" | Rating of reporting | Rating of "Risk of bias" |
| **1. Study Participation** | Goal: To judge the risk of selection bias (likelihood that relationship between PF and outcome is different for participants and eligible non-participants). |  |  |
| Source of target population | The source population or population of interest is adequately described for key characteristics (LIST). | Yes |  |
| Method used to identify population | The sampling frame and recruitment are adequately described, including methods to identify the sample sufficient to limit potential bias (number and type used, e.g., referral patterns in health care) | Partial |  |
| Recruitment period | Period of recruitment is adequately described | Yes |  |
| Place of recruitment | Place of recruitment (setting and geographic location) are adequately described | Yes |  |
| Inclusion and exclusion criteria | Inclusion and exclusion criteria are adequately described (e.g., including explicit diagnostic criteria or “zero time” description). | Partial |  |
| Adequate study participation | There is adequate participation in the study by eligible individuals | Yes |  |
| Baseline characteristics | The baseline study sample (i.e., individuals entering the study) is adequately described for key characteristics (LIST). | Partial |  |
| **Summary Study participation** | The study sample represents the population of interest on key characteristics, sufficient to limit potential bias of the observed relationship between PF and outcome. |  | Low |
|  | | | |
| **2. Study Attrition** | Goal: To judge the risk of attrition bias (likelihood that relationship between PF and outcome are different for completing and non-completing participants). |  |  |
| Proportion of baseline sample available for analysis | Response rate (i.e., proportion of study sample completing the study and providing outcome data) is adequate. | Yes |  |
| Attempts to collect information on participants who dropped out | Attempts to collect information on participants who dropped out of the study are described. | No |  |
| Reasons and potential impact of subjects lost to follow-up | Reasons for loss to follow-up are provided. | Yes |  |
| Outcome and prognostic factor information on those lost to follow-up | Participants lost to follow-up are adequately described for key characteristics (LIST)./ There are no important differences between key characteristics (LIST) and outcomes in participants who completed the study and those who did not. | No |  |
| **Study Attrition Summary** | Loss to follow-up (from baseline sample to study population analyzed) is not associated with key characteristics (i.e., the study data adequately represent the sample) sufficient to limit potential bias to the observed relationship between PF and outcome. |  | Low |
|  | | | |
| **3. Prognostic Factor Measurement** | Goal: To judge the risk of measurement bias related to how PF was measured (differential measurement of PF related to the level of outcome). |  |  |
| Definition of the PF | A clear definition or description of 'PF' is provided (e.g., including dose, level, duration of exposure, and clear specification of the method of measurement). | Partial |  |
| Valid and Reliable Measurement of PF | Method of PF measurement is adequately valid and reliable to limit misclassification bias (e.g., may include relevant outside sources of information on measurement properties, also characteristics, such as blind measurement and limited reliance on recall)./ Continuous variables are reported or appropriate cut-points (i.e., not data-dependent) are used. | No |  |
| Method and Setting of PF Measurement | The method and setting of measurement of PF is the same for all study participants. | Yes |  |
| Proportion of data on PF available for analysis | Adequate proportion of the study sample has complete data for PF variable. | Yes |  |
| Method used for missing data | Appropriate methods of imputation are used for missing 'PF' data. | No |  |
| **PF Measurement Summary** | PF is adequately measured in study participants to sufficiently limit potential bias. |  | Moderate |
|  | | | |
| 4. Outcome Measurement | Goal: To judge the risk of bias related to the measurement of outcome (differential measurement of outcome related to the baseline level of PF). |  |  |
| Definition of the Outcome | A clear definition of outcome is provided, including duration of follow-up and level and extent of the outcome construct. | Yes |  |
| Valid and Reliable Measurement of Outcome | The method of outcome measurement used is adequately valid and reliable to limit misclassification bias (e.g., may include relevant outside sources of information on measurement properties, also characteristics, such as blind measurement and confirmation of outcome with valid and reliable test). | Yes |  |
| Method and Setting of Outcome Measurement | The method and setting of outcome measurement is the same for all study participants. | Yes |  |
| **Outcome Measurement Summary** | Outcome of interest is adequately measured in study participants to sufficiently limit potential bias. |  | Low |
|  | | | |
| **5. Study Confounding** | Goal: To judge the risk of bias due to confounding (i.e. the effect of PF is distorted by another factor that is related to PF and outcome). |  |  |
| Important Confounders Measured | All important confounders, including treatments (key variables in conceptual model: LIST), are measured. | No |  |
| Definition of the confounding factor | Clear definitions of the important confounders measured are provided (e.g., including dose, level, and duration of exposures). | No |  |
| Valid and Reliable Measurement of Confounders | Measurement of all important confounders is adequately valid and reliable (e.g., may include relevant outside sources of information on measurement properties, also characteristics, such as blind measurement and limited reliance on recall). | No |  |
| Method and Setting of Confounding Measurement | The method and setting of confounding measurement are the same for all study participants. | Yes |  |
| Method used for missing data | Appropriate methods are used if imputation is used for missing confounder data. | No |  |
| Appropriate Accounting for Confounding | Important potential confounders are accounted for in the study design (e.g., matching for key variables, stratification, or initial assembly of comparable groups). /Important potential confounders are accounted for in the analysis (i.e., appropriate adjustment). | No |  |
| **Study Confounding Summary** | Important potential confounders are appropriately accounted for, limiting potential bias with respect to the relationship between PF and outcome . |  | High |
|  | | | |
| **6. Statistical Analysis and Reporting** | Goal: To judge the risk of bias related to the statistical analysis and presentation of results. |  |  |
| Presentation of analytical strategy | There is sufficient presentation of data to assess the adequacy of the analysis. | Yes |  |
| Model development strategy | The strategy for model building (i.e., inclusion of variables in the statistical model) is appropriate and is based on a conceptual framework or model./ The selected statistical model is adequate for the design of the study. | No |  |
| Reporting of results | There is no selective reporting of results. | Unsure |  |
| **Statistical Analysis and Presentation Summary** | The statistical analysis is appropriate for the design of the study, limiting potential for presentation of invalid or spurious results. |  | Low |
|  | | | |
| Study title: | Nipah encephalitis outbreak in Malaysia, clinical features in patients from Seremban | | |
| Study author/ data: | Chong et al., 2002 | | |
| Study identifier: | 10.1017/S0317167100001785 | | |
|  | | | |
| Biases | Issues to consider for judging overall rating of "Risk of bias" | Rating of reporting | Rating of "Risk of bias" |
| **1. Study Participation** | Goal: To judge the risk of selection bias (likelihood that relationship between PF and outcome is different for participants and eligible non-participants). |  |  |
| Source of target population | The source population or population of interest is adequately described for key characteristics (LIST). | Yes |  |
| Method used to identify population | The sampling frame and recruitment are adequately described, including methods to identify the sample sufficient to limit potential bias (number and type used, e.g., referral patterns in health care) | Partial |  |
| Recruitment period | Period of recruitment is adequately described | Yes |  |
| Place of recruitment | Place of recruitment (setting and geographic location) are adequately described | Yes |  |
| Inclusion and exclusion criteria | Inclusion and exclusion criteria are adequately described (e.g., including explicit diagnostic criteria or “zero time” description). | No |  |
| Adequate study participation | There is adequate participation in the study by eligible individuals | Yes |  |
| Baseline characteristics | The baseline study sample (i.e., individuals entering the study) is adequately described for key characteristics (LIST). | Yes |  |
| **Summary Study participation** | The study sample represents the population of interest on key characteristics, sufficient to limit potential bias of the observed relationship between PF and outcome. |  | Low |
|  | | | |
| **2. Study Attrition** | Goal: To judge the risk of attrition bias (likelihood that relationship between PF and outcome are different for completing and non-completing participants). |  |  |
| Proportion of baseline sample available for analysis | Response rate (i.e., proportion of study sample completing the study and providing outcome data) is adequate. | Yes |  |
| Attempts to collect information on participants who dropped out | Attempts to collect information on participants who dropped out of the study are described. | No |  |
| Reasons and potential impact of subjects lost to follow-up | Reasons for loss to follow-up are provided. | Yes |  |
| Outcome and prognostic factor information on those lost to follow-up | Participants lost to follow-up are adequately described for key characteristics (LIST)./ There are no important differences between key characteristics (LIST) and outcomes in participants who completed the study and those who did not. | No |  |
| **Study Attrition Summary** | Loss to follow-up (from baseline sample to study population analyzed) is not associated with key characteristics (i.e., the study data adequately represent the sample) sufficient to limit potential bias to the observed relationship between PF and outcome. |  | Low |
|  | | | |
| **3. Prognostic Factor Measurement** | Goal: To judge the risk of measurement bias related to how PF was measured (differential measurement of PF related to the level of outcome). |  |  |
| Definition of the PF | A clear definition or description of 'PF' is provided (e.g., including dose, level, duration of exposure, and clear specification of the method of measurement). | Partial |  |
| Valid and Reliable Measurement of PF | Method of PF measurement is adequately valid and reliable to limit misclassification bias (e.g., may include relevant outside sources of information on measurement properties, also characteristics, such as blind measurement and limited reliance on recall)./ Continuous variables are reported or appropriate cut-points (i.e., not data-dependent) are used. | Yes |  |
| Method and Setting of PF Measurement | The method and setting of measurement of PF is the same for all study participants. | Yes |  |
| Proportion of data on PF available for analysis | Adequate proportion of the study sample has complete data for PF variable. | Partial |  |
| Method used for missing data | Appropriate methods of imputation are used for missing 'PF' data. | No |  |
| **PF Measurement Summary** | PF is adequately measured in study participants to sufficiently limit potential bias. |  | Moderate |
|  | | | |
| 4. Outcome Measurement | Goal: To judge the risk of bias related to the measurement of outcome (differential measurement of outcome related to the baseline level of PF). |  |  |
| Definition of the Outcome | A clear definition of outcome is provided, including duration of follow-up and level and extent of the outcome construct. | Yes |  |
| Valid and Reliable Measurement of Outcome | The method of outcome measurement used is adequately valid and reliable to limit misclassification bias (e.g., may include relevant outside sources of information on measurement properties, also characteristics, such as blind measurement and confirmation of outcome with valid and reliable test). | Yes |  |
| Method and Setting of Outcome Measurement | The method and setting of outcome measurement is the same for all study participants. | Yes |  |
| **Outcome Measurement Summary** | Outcome of interest is adequately measured in study participants to sufficiently limit potential bias. |  | Low |
|  | | | |
| **5. Study Confounding** | Goal: To judge the risk of bias due to confounding (i.e. the effect of PF is distorted by another factor that is related to PF and outcome). |  |  |
| Important Confounders Measured | All important confounders, including treatments (key variables in conceptual model: LIST), are measured. | Yes |  |
| Definition of the confounding factor | Clear definitions of the important confounders measured are provided (e.g., including dose, level, and duration of exposures). | No |  |
| Valid and Reliable Measurement of Confounders | Measurement of all important confounders is adequately valid and reliable (e.g., may include relevant outside sources of information on measurement properties, also characteristics, such as blind measurement and limited reliance on recall). | Yes |  |
| Method and Setting of Confounding Measurement | The method and setting of confounding measurement are the same for all study participants. | YES |  |
| Method used for missing data | Appropriate methods are used if imputation is used for missing confounder data. | No |  |
| Appropriate Accounting for Confounding | Important potential confounders are accounted for in the study design (e.g., matching for key variables, stratification, or initial assembly of comparable groups). /Important potential confounders are accounted for in the analysis (i.e., appropriate adjustment). | Yes |  |
| **Study Confounding Summary** | Important potential confounders are appropriately accounted for, limiting potential bias with respect to the relationship between PF and outcome . |  | Low |
|  | | | |
| **6. Statistical Analysis and Reporting** | Goal: To judge the risk of bias related to the statistical analysis and presentation of results. |  |  |
| Presentation of analytical strategy | There is sufficient presentation of data to assess the adequacy of the analysis. | Yes |  |
| Model development strategy | The strategy for model building (i.e., inclusion of variables in the statistical model) is appropriate and is based on a conceptual framework or model./ The selected statistical model is adequate for the design of the study. | Partial |  |
| Reporting of results | There is no selective reporting of results. | No |  |
| **Statistical Analysis and Presentation Summary** | The statistical analysis is appropriate for the design of the study, limiting potential for presentation of invalid or spurious results. |  | Low |
|  | | | |
| Study title: | Clinical Outcome and Neurological Sequelae in Serologically Confirmed Cases of Japanese Encephalitis Patients in Assam, India | | |
| Study author/ data: | Baruah et al,. 2002 | | |
| Study identifier: | PMID: 12522277 | | |
|  | | | |
| Biases | Issues to consider for judging overall rating of "Risk of bias" | Rating of reporting | Rating of "Risk of bias" |
| **1. Study Participation** | Goal: To judge the risk of selection bias (likelihood that relationship between PF and outcome is different for participants and eligible non-participants). |  |  |
| Source of target population | The source population or population of interest is adequately described for key characteristics (LIST). | No |  |
| Method used to identify population | The sampling frame and recruitment are adequately described, including methods to identify the sample sufficient to limit potential bias (number and type used, e.g., referral patterns in health care) | Partial |  |
| Recruitment period | Period of recruitment is adequately described | Yes |  |
| Place of recruitment | Place of recruitment (setting and geographic location) are adequately described | Yes |  |
| Inclusion and exclusion criteria | Inclusion and exclusion criteria are adequately described (e.g., including explicit diagnostic criteria or “zero time” description). | Partial |  |
| Adequate study participation | There is adequate participation in the study by eligible individuals | Yes |  |
| Baseline characteristics | The baseline study sample (i.e., individuals entering the study) is adequately described for key characteristics (LIST). | No |  |
| **Summary Study participation** | The study sample represents the population of interest on key characteristics, sufficient to limit potential bias of the observed relationship between PF and outcome. |  | Moderate |
|  | | | |
| **2. Study Attrition** | Goal: To judge the risk of attrition bias (likelihood that relationship between PF and outcome are different for completing and non-completing participants). |  |  |
| Proportion of baseline sample available for analysis | Response rate (i.e., proportion of study sample completing the study and providing outcome data) is adequate. | No |  |
| Attempts to collect information on participants who dropped out | Attempts to collect information on participants who dropped out of the study are described. | No |  |
| Reasons and potential impact of subjects lost to follow-up | Reasons for loss to follow-up are provided. | Yes |  |
| Outcome and prognostic factor information on those lost to follow-up | Participants lost to follow-up are adequately described for key characteristics (LIST)./ There are no important differences between key characteristics (LIST) and outcomes in participants who completed the study and those who did not. | No |  |
| **Study Attrition Summary** | Loss to follow-up (from baseline sample to study population analyzed) is not associated with key characteristics (i.e., the study data adequately represent the sample) sufficient to limit potential bias to the observed relationship between PF and outcome. |  | Low |
|  | | | |
| **3. Prognostic Factor Measurement** | Goal: To judge the risk of measurement bias related to how PF was measured (differential measurement of PF related to the level of outcome). |  |  |
| Definition of the PF | A clear definition or description of 'PF' is provided (e.g., including dose, level, duration of exposure, and clear specification of the method of measurement). | No |  |
| Valid and Reliable Measurement of PF | Method of PF measurement is adequately valid and reliable to limit misclassification bias (e.g., may include relevant outside sources of information on measurement properties, also characteristics, such as blind measurement and limited reliance on recall)./ Continuous variables are reported or appropriate cut-points (i.e., not data-dependent) are used. | Unsure |  |
| Method and Setting of PF Measurement | The method and setting of measurement of PF is the same for all study participants. | Yes |  |
| Proportion of data on PF available for analysis | Adequate proportion of the study sample has complete data for PF variable. | Yes |  |
| Method used for missing data | Appropriate methods of imputation are used for missing 'PF' data. | No |  |
| **PF Measurement Summary** | PF is adequately measured in study participants to sufficiently limit potential bias. |  | Low |
|  | | | |
| 4. Outcome Measurement | Goal: To judge the risk of bias related to the measurement of outcome (differential measurement of outcome related to the baseline level of PF). |  |  |
| Definition of the Outcome | A clear definition of outcome is provided, including duration of follow-up and level and extent of the outcome construct. | Yes |  |
| Valid and Reliable Measurement of Outcome | The method of outcome measurement used is adequately valid and reliable to limit misclassification bias (e.g., may include relevant outside sources of information on measurement properties, also characteristics, such as blind measurement and confirmation of outcome with valid and reliable test). | No |  |
| Method and Setting of Outcome Measurement | The method and setting of outcome measurement is the same for all study participants. | Yes |  |
| **Outcome Measurement Summary** | Outcome of interest is adequately measured in study participants to sufficiently limit potential bias. |  | Low |
|  | | | |
| **5. Study Confounding** | Goal: To judge the risk of bias due to confounding (i.e. the effect of PF is distorted by another factor that is related to PF and outcome). |  |  |
| Important Confounders Measured | All important confounders, including treatments (key variables in conceptual model: LIST), are measured. | No |  |
| Definition of the confounding factor | Clear definitions of the important confounders measured are provided (e.g., including dose, level, and duration of exposures). | No |  |
| Valid and Reliable Measurement of Confounders | Measurement of all important confounders is adequately valid and reliable (e.g., may include relevant outside sources of information on measurement properties, also characteristics, such as blind measurement and limited reliance on recall). | Yes |  |
| Method and Setting of Confounding Measurement | The method and setting of confounding measurement are the same for all study participants. | No |  |
| Method used for missing data | Appropriate methods are used if imputation is used for missing confounder data. | No |  |
| Appropriate Accounting for Confounding | Important potential confounders are accounted for in the study design (e.g., matching for key variables, stratification, or initial assembly of comparable groups). /Important potential confounders are accounted for in the analysis (i.e., appropriate adjustment). | No |  |
| **Study Confounding Summary** | Important potential confounders are appropriately accounted for, limiting potential bias with respect to the relationship between PF and outcome . |  | Moderate |
|  | | | |
| **6. Statistical Analysis and Reporting** | Goal: To judge the risk of bias related to the statistical analysis and presentation of results. |  |  |
| Presentation of analytical strategy | There is sufficient presentation of data to assess the adequacy of the analysis. | No |  |
| Model development strategy | The strategy for model building (i.e., inclusion of variables in the statistical model) is appropriate and is based on a conceptual framework or model./ The selected statistical model is adequate for the design of the study. | No |  |
| Reporting of results | There is no selective reporting of results. | Yes |  |
| **Statistical Analysis and Presentation Summary** | The statistical analysis is appropriate for the design of the study, limiting potential for presentation of invalid or spurious results. |  | No |
|  | | | |
| Study title: | Infectious Encephalitis in France in 2007: A National Prospective Study | | |
| Study author/ data: | Mailles et al., 2009 | | |
| Study identifier: | 10.1086/648419 | | |
|  | | | |
| Biases | Issues to consider for judging overall rating of "Risk of bias" | Rating of reporting | Rating of "Risk of bias" |
| **1. Study Participation** | Goal: To judge the risk of selection bias (likelihood that relationship between PF and outcome is different for participants and eligible non-participants). |  |  |
| Source of target population | The source population or population of interest is adequately described for key characteristics (LIST). | Yes |  |
| Method used to identify population | The sampling frame and recruitment are adequately described, including methods to identify the sample sufficient to limit potential bias (number and type used, e.g., referral patterns in health care) | Yes |  |
| Recruitment period | Period of recruitment is adequately described | Yes |  |
| Place of recruitment | Place of recruitment (setting and geographic location) are adequately described | Yes |  |
| Inclusion and exclusion criteria | Inclusion and exclusion criteria are adequately described (e.g., including explicit diagnostic criteria or “zero time” description). | Yes |  |
| Adequate study participation | There is adequate participation in the study by eligible individuals | Yes |  |
| Baseline characteristics | The baseline study sample (i.e., individuals entering the study) is adequately described for key characteristics (LIST). | Yes |  |
| **Summary Study participation** | The study sample represents the population of interest on key characteristics, sufficient to limit potential bias of the observed relationship between PF and outcome. |  | Low |
|  | | | |
| **2. Study Attrition** | Goal: To judge the risk of attrition bias (likelihood that relationship between PF and outcome are different for completing and non-completing participants). |  |  |
| Proportion of baseline sample available for analysis | Response rate (i.e., proportion of study sample completing the study and providing outcome data) is adequate. | Yes |  |
| Attempts to collect information on participants who dropped out | Attempts to collect information on participants who dropped out of the study are described. | Yes |  |
| Reasons and potential impact of subjects lost to follow-up | Reasons for loss to follow-up are provided. | Yes |  |
| Outcome and prognostic factor information on those lost to follow-up | Participants lost to follow-up are adequately described for key characteristics (LIST)./ There are no important differences between key characteristics (LIST) and outcomes in participants who completed the study and those who did not. | Partial |  |
| **Study Attrition Summary** | Loss to follow-up (from baseline sample to study population analyzed) is not associated with key characteristics (i.e., the study data adequately represent the sample) sufficient to limit potential bias to the observed relationship between PF and outcome. |  | Low |
|  | | | |
| **3. Prognostic Factor Measurement** | Goal: To judge the risk of measurement bias related to how PF was measured (differential measurement of PF related to the level of outcome). |  |  |
| Definition of the PF | A clear definition or description of 'PF' is provided (e.g., including dose, level, duration of exposure, and clear specification of the method of measurement). | Unsure |  |
| Valid and Reliable Measurement of PF | Method of PF measurement is adequately valid and reliable to limit misclassification bias (e.g., may include relevant outside sources of information on measurement properties, also characteristics, such as blind measurement and limited reliance on recall)./ Continuous variables are reported or appropriate cut-points (i.e., not data-dependent) are used. | Yes |  |
| Method and Setting of PF Measurement | The method and setting of measurement of PF is the same for all study participants. | Yes |  |
| Proportion of data on PF available for analysis | Adequate proportion of the study sample has complete data for PF variable. | Yes |  |
| Method used for missing data | Appropriate methods of imputation are used for missing 'PF' data. | Unsure |  |
| **PF Measurement Summary** | PF is adequately measured in study participants to sufficiently limit potential bias. |  | Low |
|  | | | |
| 4. Outcome Measurement | Goal: To judge the risk of bias related to the measurement of outcome (differential measurement of outcome related to the baseline level of PF). |  |  |
| Definition of the Outcome | A clear definition of outcome is provided, including duration of follow-up and level and extent of the outcome construct. | Yes |  |
| Valid and Reliable Measurement of Outcome | The method of outcome measurement used is adequately valid and reliable to limit misclassification bias (e.g., may include relevant outside sources of information on measurement properties, also characteristics, such as blind measurement and confirmation of outcome with valid and reliable test). | Yes |  |
| Method and Setting of Outcome Measurement | The method and setting of outcome measurement is the same for all study participants. | Yes |  |
| **Outcome Measurement Summary** | Outcome of interest is adequately measured in study participants to sufficiently limit potential bias. |  | Low |
|  | | | |
| **5. Study Confounding** | Goal: To judge the risk of bias due to confounding (i.e. the effect of PF is distorted by another factor that is related to PF and outcome). |  |  |
| Important Confounders Measured | All important confounders, including treatments (key variables in conceptual model: LIST), are measured. | Partial |  |
| Definition of the confounding factor | Clear definitions of the important confounders measured are provided (e.g., including dose, level, and duration of exposures). | Partial |  |
| Valid and Reliable Measurement of Confounders | Measurement of all important confounders is adequately valid and reliable (e.g., may include relevant outside sources of information on measurement properties, also characteristics, such as blind measurement and limited reliance on recall). | Partial |  |
| Method and Setting of Confounding Measurement | The method and setting of confounding measurement are the same for all study participants. | Yes |  |
| Method used for missing data | Appropriate methods are used if imputation is used for missing confounder data. | No |  |
| Appropriate Accounting for Confounding | Important potential confounders are accounted for in the study design (e.g., matching for key variables, stratification, or initial assembly of comparable groups). /Important potential confounders are accounted for in the analysis (i.e., appropriate adjustment). | Yes |  |
| **Study Confounding Summary** | Important potential confounders are appropriately accounted for, limiting potential bias with respect to the relationship between PF and outcome . |  | Low |
|  | | | |
| **6. Statistical Analysis and Reporting** | Goal: To judge the risk of bias related to the statistical analysis and presentation of results. |  |  |
| Presentation of analytical strategy | There is sufficient presentation of data to assess the adequacy of the analysis. | Yes |  |
| Model development strategy | The strategy for model building (i.e., inclusion of variables in the statistical model) is appropriate and is based on a conceptual framework or model./ The selected statistical model is adequate for the design of the study. | Partial |  |
| Reporting of results | There is no selective reporting of results. | No |  |
| **Statistical Analysis and Presentation Summary** | The statistical analysis is appropriate for the design of the study, limiting potential for presentation of invalid or spurious results. |  | Low |
|  | | | |
| Study title: | Computed tomography and EEG in herpes simplex encephalitis. Their value in diagnosis and prognosis. | | |
| Study author/ data: | Dutt et al., 1982 | | |
| Study identifier: | 10.1001/archneur.1982.00510140033008 | | |
|  | | | |
| Biases | Issues to consider for judging overall rating of "Risk of bias" | Rating of reporting | Rating of "Risk of bias" |
| **1. Study Participation** | Goal: To judge the risk of selection bias (likelihood that relationship between PF and outcome is different for participants and eligible non-participants). |  |  |
| Source of target population | The source population or population of interest is adequately described for key characteristics (LIST). | No |  |
| Method used to identify population | The sampling frame and recruitment are adequately described, including methods to identify the sample sufficient to limit potential bias (number and type used, e.g., referral patterns in health care) | No |  |
| Recruitment period | Period of recruitment is adequately described | Partial |  |
| Place of recruitment | Place of recruitment (setting and geographic location) are adequately described | No |  |
| Inclusion and exclusion criteria | Inclusion and exclusion criteria are adequately described (e.g., including explicit diagnostic criteria or “zero time” description). | No |  |
| Adequate study participation | There is adequate participation in the study by eligible individuals | Yes |  |
| Baseline characteristics | The baseline study sample (i.e., individuals entering the study) is adequately described for key characteristics (LIST). | No |  |
| **Summary Study participation** | The study sample represents the population of interest on key characteristics, sufficient to limit potential bias of the observed relationship between PF and outcome. |  | High |
|  | | | |
| **2. Study Attrition** | Goal: To judge the risk of attrition bias (likelihood that relationship between PF and outcome are different for completing and non-completing participants). |  |  |
| Proportion of baseline sample available for analysis | Response rate (i.e., proportion of study sample completing the study and providing outcome data) is adequate. | Yes |  |
| Attempts to collect information on participants who dropped out | Attempts to collect information on participants who dropped out of the study are described. | No |  |
| Reasons and potential impact of subjects lost to follow-up | Reasons for loss to follow-up are provided. | No |  |
| Outcome and prognostic factor information on those lost to follow-up | Participants lost to follow-up are adequately described for key characteristics (LIST)./ There are no important differences between key characteristics (LIST) and outcomes in participants who completed the study and those who did not. | No |  |
| **Study Attrition Summary** | Loss to follow-up (from baseline sample to study population analyzed) is not associated with key characteristics (i.e., the study data adequately represent the sample) sufficient to limit potential bias to the observed relationship between PF and outcome. |  | Low |
|  | | | |
| **3. Prognostic Factor Measurement** | Goal: To judge the risk of measurement bias related to how PF was measured (differential measurement of PF related to the level of outcome). |  |  |
| Definition of the PF | A clear definition or description of 'PF' is provided (e.g., including dose, level, duration of exposure, and clear specification of the method of measurement). | No |  |
| Valid and Reliable Measurement of PF | Method of PF measurement is adequately valid and reliable to limit misclassification bias (e.g., may include relevant outside sources of information on measurement properties, also characteristics, such as blind measurement and limited reliance on recall)./ Continuous variables are reported or appropriate cut-points (i.e., not data-dependent) are used. | No |  |
| Method and Setting of PF Measurement | The method and setting of measurement of PF is the same for all study participants. | No |  |
| Proportion of data on PF available for analysis | Adequate proportion of the study sample has complete data for PF variable. | No |  |
| Method used for missing data | Appropriate methods of imputation are used for missing 'PF' data. | No | High |
| **PF Measurement Summary** | PF is adequately measured in study participants to sufficiently limit potential bias. |  |  |
|  | | | |
| 4. Outcome Measurement | Goal: To judge the risk of bias related to the measurement of outcome (differential measurement of outcome related to the baseline level of PF). |  |  |
| Definition of the Outcome | A clear definition of outcome is provided, including duration of follow-up and level and extent of the outcome construct. | Partial |  |
| Valid and Reliable Measurement of Outcome | The method of outcome measurement used is adequately valid and reliable to limit misclassification bias (e.g., may include relevant outside sources of information on measurement properties, also characteristics, such as blind measurement and confirmation of outcome with valid and reliable test). | Partial |  |
| Method and Setting of Outcome Measurement | The method and setting of outcome measurement is the same for all study participants. | Yes |  |
| **Outcome Measurement Summary** | Outcome of interest is adequately measured in study participants to sufficiently limit potential bias. |  | Moderate |
|  | | | |
| **5. Study Confounding** | Goal: To judge the risk of bias due to confounding (i.e. the effect of PF is distorted by another factor that is related to PF and outcome). |  |  |
| Important Confounders Measured | All important confounders, including treatments (key variables in conceptual model: LIST), are measured. | No |  |
| Definition of the confounding factor | Clear definitions of the important confounders measured are provided (e.g., including dose, level, and duration of exposures). | No |  |
| Valid and Reliable Measurement of Confounders | Measurement of all important confounders is adequately valid and reliable (e.g., may include relevant outside sources of information on measurement properties, also characteristics, such as blind measurement and limited reliance on recall). | No |  |
| Method and Setting of Confounding Measurement | The method and setting of confounding measurement are the same for all study participants. | Unsure |  |
| Method used for missing data | Appropriate methods are used if imputation is used for missing confounder data. | No |  |
| Appropriate Accounting for Confounding | Important potential confounders are accounted for in the study design (e.g., matching for key variables, stratification, or initial assembly of comparable groups). /Important potential confounders are accounted for in the analysis (i.e., appropriate adjustment). | No |  |
| **Study Confounding Summary** | Important potential confounders are appropriately accounted for, limiting potential bias with respect to the relationship between PF and outcome . |  | High |
|  | | | |
| **6. Statistical Analysis and Reporting** | Goal: To judge the risk of bias related to the statistical analysis and presentation of results. |  |  |
| Presentation of analytical strategy | There is sufficient presentation of data to assess the adequacy of the analysis. | Yes |  |
| Model development strategy | The strategy for model building (i.e., inclusion of variables in the statistical model) is appropriate and is based on a conceptual framework or model./ The selected statistical model is adequate for the design of the study. | No |  |
| Reporting of results | There is no selective reporting of results. | Unsure |  |
| **Statistical Analysis and Presentation Summary** | The statistical analysis is appropriate for the design of the study, limiting potential for presentation of invalid or spurious results. |  | Moderate |
|  | | | |
| Study title: | Clinical features of Nipah virus encephalitis among pig farmers in Malaysia | | |
| Study author/ data: | Goh et al., 2000 | | |
| Study identifier: | 10.1056/NEJM20000427342170 | | |
|  | | | |
| Biases | Issues to consider for judging overall rating of "Risk of bias" | Rating of reporting | Rating of "Risk of bias" |
| **1. Study Participation** | Goal: To judge the risk of selection bias (likelihood that relationship between PF and outcome is different for participants and eligible non-participants). |  |  |
| Source of target population | The source population or population of interest is adequately described for key characteristics (LIST). | Partial |  |
| Method used to identify population | The sampling frame and recruitment are adequately described, including methods to identify the sample sufficient to limit potential bias (number and type used, e.g., referral patterns in health care) | Partial |  |
| Recruitment period | Period of recruitment is adequately described | Yes |  |
| Place of recruitment | Place of recruitment (setting and geographic location) are adequately described | Yes |  |
| Inclusion and exclusion criteria | Inclusion and exclusion criteria are adequately described (e.g., including explicit diagnostic criteria or “zero time” description). | Partial |  |
| Adequate study participation | There is adequate participation in the study by eligible individuals | Yes |  |
| Baseline characteristics | The baseline study sample (i.e., individuals entering the study) is adequately described for key characteristics (LIST). | Partial |  |
| **Summary Study participation** | The study sample represents the population of interest on key characteristics, sufficient to limit potential bias of the observed relationship between PF and outcome. |  | Low |
|  | | | |
| **2. Study Attrition** | Goal: To judge the risk of attrition bias (likelihood that relationship between PF and outcome are different for completing and non-completing participants). |  |  |
| Proportion of baseline sample available for analysis | Response rate (i.e., proportion of study sample completing the study and providing outcome data) is adequate. | Yes |  |
| Attempts to collect information on participants who dropped out | Attempts to collect information on participants who dropped out of the study are described. | No |  |
| Reasons and potential impact of subjects lost to follow-up | Reasons for loss to follow-up are provided. | No |  |
| Outcome and prognostic factor information on those lost to follow-up | Participants lost to follow-up are adequately described for key characteristics (LIST)./ There are no important differences between key characteristics (LIST) and outcomes in participants who completed the study and those who did not. | No |  |
| **Study Attrition Summary** | Loss to follow-up (from baseline sample to study population analyzed) is not associated with key characteristics (i.e., the study data adequately represent the sample) sufficient to limit potential bias to the observed relationship between PF and outcome. |  | Moderate |
|  | | | |
| **3. Prognostic Factor Measurement** | Goal: To judge the risk of measurement bias related to how PF was measured (differential measurement of PF related to the level of outcome). |  |  |
| Definition of the PF | A clear definition or description of 'PF' is provided (e.g., including dose, level, duration of exposure, and clear specification of the method of measurement). | No |  |
| Valid and Reliable Measurement of PF | Method of PF measurement is adequately valid and reliable to limit misclassification bias (e.g., may include relevant outside sources of information on measurement properties, also characteristics, such as blind measurement and limited reliance on recall)./ Continuous variables are reported or appropriate cut-points (i.e., not data-dependent) are used. | Yes |  |
| Method and Setting of PF Measurement | The method and setting of measurement of PF is the same for all study participants. | Yes |  |
| Proportion of data on PF available for analysis | Adequate proportion of the study sample has complete data for PF variable. | Partial |  |
| Method used for missing data | Appropriate methods of imputation are used for missing 'PF' data. | No |  |
| **PF Measurement Summary** | PF is adequately measured in study participants to sufficiently limit potential bias. |  | Low |
|  | | | |
| 4. Outcome Measurement | Goal: To judge the risk of bias related to the measurement of outcome (differential measurement of outcome related to the baseline level of PF). |  |  |
| Definition of the Outcome | A clear definition of outcome is provided, including duration of follow-up and level and extent of the outcome construct. | Yes |  |
| Valid and Reliable Measurement of Outcome | The method of outcome measurement used is adequately valid and reliable to limit misclassification bias (e.g., may include relevant outside sources of information on measurement properties, also characteristics, such as blind measurement and confirmation of outcome with valid and reliable test). | Yes |  |
| Method and Setting of Outcome Measurement | The method and setting of outcome measurement is the same for all study participants. | Yes |  |
| **Outcome Measurement Summary** | Outcome of interest is adequately measured in study participants to sufficiently limit potential bias. |  | Low |
|  | | | |
| **5. Study Confounding** | Goal: To judge the risk of bias due to confounding (i.e. the effect of PF is distorted by another factor that is related to PF and outcome). |  |  |
| Important Confounders Measured | All important confounders, including treatments (key variables in conceptual model: LIST), are measured. | No |  |
| Definition of the confounding factor | Clear definitions of the important confounders measured are provided (e.g., including dose, level, and duration of exposures). | No |  |
| Valid and Reliable Measurement of Confounders | Measurement of all important confounders is adequately valid and reliable (e.g., may include relevant outside sources of information on measurement properties, also characteristics, such as blind measurement and limited reliance on recall). | Yes |  |
| Method and Setting of Confounding Measurement | The method and setting of confounding measurement are the same for all study participants. | Yes |  |
| Method used for missing data | Appropriate methods are used if imputation is used for missing confounder data. | No |  |
| Appropriate Accounting for Confounding | Important potential confounders are accounted for in the study design (e.g., matching for key variables, stratification, or initial assembly of comparable groups). /Important potential confounders are accounted for in the analysis (i.e., appropriate adjustment). | No |  |
| **Study Confounding Summary** | Important potential confounders are appropriately accounted for, limiting potential bias with respect to the relationship between PF and outcome . |  | High |
|  | | | |
| **6. Statistical Analysis and Reporting** | Goal: To judge the risk of bias related to the statistical analysis and presentation of results. |  |  |
| Presentation of analytical strategy | There is sufficient presentation of data to assess the adequacy of the analysis. | Unsure |  |
| Model development strategy | The strategy for model building (i.e., inclusion of variables in the statistical model) is appropriate and is based on a conceptual framework or model./ The selected statistical model is adequate for the design of the study. | No |  |
| Reporting of results | There is no selective reporting of results. | Unsure |  |
| **Statistical Analysis and Presentation Summary** | The statistical analysis is appropriate for the design of the study, limiting potential for presentation of invalid or spurious results. |  | Moderate |
|  | | | |
| Study title: | Japanese Encephalitis in and around Pondicherry, South India: a Clinical Appraisal and Prognostic Indicators for the Outcome | | |
| Study author/ data: | Potula et al., 2014 | | |
| Study identifier: | 10.1093/tropej/49.1.48 | | |
|  | | | |
| Biases | Issues to consider for judging overall rating of "Risk of bias" | Rating of reporting | Rating of "Risk of bias" |
| **1. Study Participation** | Goal: To judge the risk of selection bias (likelihood that relationship between PF and outcome is different for participants and eligible non-participants). |  |  |
| Source of target population | The source population or population of interest is adequately described for key characteristics (LIST). | Partial |  |
| Method used to identify population | The sampling frame and recruitment are adequately described, including methods to identify the sample sufficient to limit potential bias (number and type used, e.g., referral patterns in health care) | Partial |  |
| Recruitment period | Period of recruitment is adequately described | No |  |
| Place of recruitment | Place of recruitment (setting and geographic location) are adequately described | Yes |  |
| Inclusion and exclusion criteria | Inclusion and exclusion criteria are adequately described (e.g., including explicit diagnostic criteria or “zero time” description). | Partial |  |
| Adequate study participation | There is adequate participation in the study by eligible individuals | Yes |  |
| Baseline characteristics | The baseline study sample (i.e., individuals entering the study) is adequately described for key characteristics (LIST). | Partial |  |
| **Summary Study participation** | The study sample represents the population of interest on key characteristics, sufficient to limit potential bias of the observed relationship between PF and outcome. |  | Low |
|  | | | |
| **2. Study Attrition** | Goal: To judge the risk of attrition bias (likelihood that relationship between PF and outcome are different for completing and non-completing participants). |  |  |
| Proportion of baseline sample available for analysis | Response rate (i.e., proportion of study sample completing the study and providing outcome data) is adequate. | Yes |  |
| Attempts to collect information on participants who dropped out | Attempts to collect information on participants who dropped out of the study are described. | No |  |
| Reasons and potential impact of subjects lost to follow-up | Reasons for loss to follow-up are provided. | No |  |
| Outcome and prognostic factor information on those lost to follow-up | Participants lost to follow-up are adequately described for key characteristics (LIST)./ There are no important differences between key characteristics (LIST) and outcomes in participants who completed the study and those who did not. | No |  |
| **Study Attrition Summary** | Loss to follow-up (from baseline sample to study population analyzed) is not associated with key characteristics (i.e., the study data adequately represent the sample) sufficient to limit potential bias to the observed relationship between PF and outcome. |  | Moderate |
|  | | | |
| **3. Prognostic Factor Measurement** | Goal: To judge the risk of measurement bias related to how PF was measured (differential measurement of PF related to the level of outcome). |  |  |
| Definition of the PF | A clear definition or description of 'PF' is provided (e.g., including dose, level, duration of exposure, and clear specification of the method of measurement). | Partial |  |
| Valid and Reliable Measurement of PF | Method of PF measurement is adequately valid and reliable to limit misclassification bias (e.g., may include relevant outside sources of information on measurement properties, also characteristics, such as blind measurement and limited reliance on recall)./ Continuous variables are reported or appropriate cut-points (i.e., not data-dependent) are used. | Partial |  |
| Method and Setting of PF Measurement | The method and setting of measurement of PF is the same for all study participants. | Unsure |  |
| Proportion of data on PF available for analysis | Adequate proportion of the study sample has complete data for PF variable. | Partial |  |
| Method used for missing data | Appropriate methods of imputation are used for missing 'PF' data. | No |  |
| **PF Measurement Summary** | PF is adequately measured in study participants to sufficiently limit potential bias. |  | Moderate |
|  | | | |
| 4. Outcome Measurement | Goal: To judge the risk of bias related to the measurement of outcome (differential measurement of outcome related to the baseline level of PF). |  |  |
| Definition of the Outcome | A clear definition of outcome is provided, including duration of follow-up and level and extent of the outcome construct. | Yes |  |
| Valid and Reliable Measurement of Outcome | The method of outcome measurement used is adequately valid and reliable to limit misclassification bias (e.g., may include relevant outside sources of information on measurement properties, also characteristics, such as blind measurement and confirmation of outcome with valid and reliable test). | Yes |  |
| Method and Setting of Outcome Measurement | The method and setting of outcome measurement is the same for all study participants. | Yes |  |
| **Outcome Measurement Summary** | Outcome of interest is adequately measured in study participants to sufficiently limit potential bias. |  | Low |
|  | | | |
| **5. Study Confounding** | Goal: To judge the risk of bias due to confounding (i.e. the effect of PF is distorted by another factor that is related to PF and outcome). |  |  |
| Important Confounders Measured | All important confounders, including treatments (key variables in conceptual model: LIST), are measured. | Partial |  |
| Definition of the confounding factor | Clear definitions of the important confounders measured are provided (e.g., including dose, level, and duration of exposures). | Partial |  |
| Valid and Reliable Measurement of Confounders | Measurement of all important confounders is adequately valid and reliable (e.g., may include relevant outside sources of information on measurement properties, also characteristics, such as blind measurement and limited reliance on recall). | Yes |  |
| Method and Setting of Confounding Measurement | The method and setting of confounding measurement are the same for all study participants. | Yes |  |
| Method used for missing data | Appropriate methods are used if imputation is used for missing confounder data. | No |  |
| Appropriate Accounting for Confounding | Important potential confounders are accounted for in the study design (e.g., matching for key variables, stratification, or initial assembly of comparable groups). /Important potential confounders are accounted for in the analysis (i.e., appropriate adjustment). | Yes |  |
| **Study Confounding Summary** | Important potential confounders are appropriately accounted for, limiting potential bias with respect to the relationship between PF and outcome . |  | Low |
|  | | | |
| **6. Statistical Analysis and Reporting** | Goal: To judge the risk of bias related to the statistical analysis and presentation of results. |  |  |
| Presentation of analytical strategy | There is sufficient presentation of data to assess the adequacy of the analysis. | Yes |  |
| Model development strategy | The strategy for model building (i.e., inclusion of variables in the statistical model) is appropriate and is based on a conceptual framework or model./ The selected statistical model is adequate for the design of the study. | Yes |  |
| Reporting of results | There is no selective reporting of results. | Yes |  |
| **Statistical Analysis and Presentation Summary** | The statistical analysis is appropriate for the design of the study, limiting potential for presentation of invalid or spurious results. |  | Low |

Poor outcomes at discharge: QUIPS analysis

| Study title: | Varicella Zoster Virus Encephalitis in Denmark From 2015 to 2019—A Nationwide Prospective Cohort Study | | |
| --- | --- | --- | --- |
| Study author/ data: | Herlin et al., 2021 | | |
| Study identifier: | 10.1093/cid/ciaa185 | | |
|  | | | |
| Biases | Issues to consider for judging overall rating of "Risk of bias" | Rating of reporting | Rating of "Risk of bias" |
| **1. Study Participation** | Goal: To judge the risk of selection bias (likelihood that relationship between PF and outcome is different for participants and eligible non-participants). |  |  |
| Source of target population | The source population or population of interest is adequately described for key characteristics (LIST). | Yes |  |
| Method used to identify population | The sampling frame and recruitment are adequately described, including methods to identify the sample sufficient to limit potential bias (number and type used, e.g., referral patterns in health care) | Partial |  |
| Recruitment period | Period of recruitment is adequately described | Yes |  |
| Place of recruitment | Place of recruitment (setting and geographic location) are adequately described | Yes |  |
| Inclusion and exclusion criteria | Inclusion and exclusion criteria are adequately described (e.g., including explicit diagnostic criteria or “zero time” description). | Yes |  |
| Adequate study participation | There is adequate participation in the study by eligible individuals | No |  |
| Baseline characteristics | The baseline study sample (i.e., individuals entering the study) is adequately described for key characteristics (LIST). | Yes |  |
| **Summary Study participation** | The study sample represents the population of interest on key characteristics, sufficient to limit potential bias of the observed relationship between PF and outcome. |  | Low |
|  | | | |
| **2. Study Attrition** | Goal: To judge the risk of attrition bias (likelihood that relationship between PF and outcome are different for completing and non-completing participants). |  |  |
| Proportion of baseline sample available for analysis | Response rate (i.e., proportion of study sample completing the study and providing outcome data) is adequate. | Yes |  |
| Attempts to collect information on participants who dropped out | Attempts to collect information on participants who dropped out of the study are described. | No |  |
| Reasons and potential impact of subjects lost to follow-up | Reasons for loss to follow-up are provided. | No |  |
| Outcome and prognostic factor information on those lost to follow-up | Participants lost to follow-up are adequately described for key characteristics (LIST)./ There are no important differences between key characteristics (LIST) and outcomes in participants who completed the study and those who did not. | No |  |
| **Study Attrition Summary** | Loss to follow-up (from baseline sample to study population analyzed) is not associated with key characteristics (i.e., the study data adequately represent the sample) sufficient to limit potential bias to the observed relationship between PF and outcome. |  | Low |
|  | | | |
| **3. Prognostic Factor Measurement** | Goal: To judge the risk of measurement bias related to how PF was measured (differential measurement of PF related to the level of outcome). |  |  |
| Definition of the PF | A clear definition or description of 'PF' is provided (e.g., including dose, level, duration of exposure, and clear specification of the method of measurement). | Yes |  |
| Valid and Reliable Measurement of PF | Method of PF measurement is adequately valid and reliable to limit misclassification bias (e.g., may include relevant outside sources of information on measurement properties, also characteristics, such as blind measurement and limited reliance on recall)./ Continuous variables are reported or appropriate cut-points (i.e., not data-dependent) are used. | Yes |  |
| Method and Setting of PF Measurement | The method and setting of measurement of PF is the same for all study participants. | Yes |  |
| Proportion of data on PF available for analysis | Adequate proportion of the study sample has complete data for PF variable. | Yes |  |
| Method used for missing data | Appropriate methods of imputation are used for missing 'PF' data. | No |  |
| **PF Measurement Summary** | PF is adequately measured in study participants to sufficiently limit potential bias. |  | Low |
|  | | | |
| 4. Outcome Measurement | Goal: To judge the risk of bias related to the measurement of outcome (differential measurement of outcome related to the baseline level of PF). |  |  |
| Definition of the Outcome | A clear definition of outcome is provided, including duration of follow-up and level and extent of the outcome construct. | Yes |  |
| Valid and Reliable Measurement of Outcome | The method of outcome measurement used is adequately valid and reliable to limit misclassification bias (e.g., may include relevant outside sources of information on measurement properties, also characteristics, such as blind measurement and confirmation of outcome with valid and reliable test). | Yes |  |
| Method and Setting of Outcome Measurement | The method and setting of outcome measurement is the same for all study participants. | Yes |  |
| **Outcome Measurement Summary** | Outcome of interest is adequately measured in study participants to sufficiently limit potential bias. |  | Low |
|  | | | |
| **5. Study Confounding** | Goal: To judge the risk of bias due to confounding (i.e. the effect of PF is distorted by another factor that is related to PF and outcome). |  |  |
| Important Confounders Measured | All important confounders, including treatments (key variables in conceptual model: LIST), are measured. | Partial |  |
| Definition of the confounding factor | Clear definitions of the important confounders measured are provided (e.g., including dose, level, and duration of exposures). | Yes |  |
| Valid and Reliable Measurement of Confounders | Measurement of all important confounders is adequately valid and reliable (e.g., may include relevant outside sources of information on measurement properties, also characteristics, such as blind measurement and limited reliance on recall). | Yes |  |
| Method and Setting of Confounding Measurement | The method and setting of confounding measurement are the same for all study participants. | Yes |  |
| Method used for missing data | Appropriate methods are used if imputation is used for missing confounder data. | Partial |  |
| Appropriate Accounting for Confounding | Important potential confounders are accounted for in the study design (e.g., matching for key variables, stratification, or initial assembly of comparable groups). /Important potential confounders are accounted for in the analysis (i.e., appropriate adjustment). | Partial |  |
| **Study Confounding Summary** | Important potential confounders are appropriately accounted for, limiting potential bias with respect to the relationship between PF and outcome . |  | Low |
|  | | | |
| **6. Statistical Analysis and Reporting** | Goal: To judge the risk of bias related to the statistical analysis and presentation of results. |  |  |
| Presentation of analytical strategy | There is sufficient presentation of data to assess the adequacy of the analysis. | Yes |  |
| Model development strategy | The strategy for model building (i.e., inclusion of variables in the statistical model) is appropriate and is based on a conceptual framework or model./ The selected statistical model is adequate for the design of the study. | Yes |  |
| Reporting of results | There is no selective reporting of results. | No |  |
| **Statistical Analysis and Presentation Summary** | The statistical analysis is appropriate for the design of the study, limiting potential for presentation of invalid or spurious results. |  | Low |
|  | | | |
| Study title: | Clinical characteristics and outcome of clinically diagnosed viral encephalitis in southwest China | | |
| Study author/ data: | Zhao et al., 2015 | | |
| Study identifier: | 10.1007/s10072-015-2333-8 | | |
|  | | | |
| Biases | Issues to consider for judging overall rating of "Risk of bias" | Rating of reporting | Rating of "Risk of bias" |
| **1. Study Participation** | Goal: To judge the risk of selection bias (likelihood that relationship between PF and outcome is different for participants and eligible non-participants). |  |  |
| Source of target population | The source population or population of interest is adequately described for key characteristics (LIST). | Partial |  |
| Method used to identify population | The sampling frame and recruitment are adequately described, including methods to identify the sample sufficient to limit potential bias (number and type used, e.g., referral patterns in health care) | No |  |
| Recruitment period | Period of recruitment is adequately described | No |  |
| Place of recruitment | Place of recruitment (setting and geographic location) are adequately described | Yes |  |
| Inclusion and exclusion criteria | Inclusion and exclusion criteria are adequately described (e.g., including explicit diagnostic criteria or “zero time” description). | Partial |  |
| Adequate study participation | There is adequate participation in the study by eligible individuals | Yes |  |
| Baseline characteristics | The baseline study sample (i.e., individuals entering the study) is adequately described for key characteristics (LIST). | Partial |  |
| **Summary Study participation** | The study sample represents the population of interest on key characteristics, sufficient to limit potential bias of the observed relationship between PF and outcome. |  | Moderate |
|  | | | |
| **2. Study Attrition** | Goal: To judge the risk of attrition bias (likelihood that relationship between PF and outcome are different for completing and non-completing participants). |  |  |
| Proportion of baseline sample available for analysis | Response rate (i.e., proportion of study sample completing the study and providing outcome data) is adequate. | Yes |  |
| Attempts to collect information on participants who dropped out | Attempts to collect information on participants who dropped out of the study are described. | No |  |
| Reasons and potential impact of subjects lost to follow-up | Reasons for loss to follow-up are provided. | No |  |
| Outcome and prognostic factor information on those lost to follow-up | Participants lost to follow-up are adequately described for key characteristics (LIST)./ There are no important differences between key characteristics (LIST) and outcomes in participants who completed the study and those who did not. | No |  |
| **Study Attrition Summary** | Loss to follow-up (from baseline sample to study population analyzed) is not associated with key characteristics (i.e., the study data adequately represent the sample) sufficient to limit potential bias to the observed relationship between PF and outcome. |  | Low |
|  | | | |
| **3. Prognostic Factor Measurement** | Goal: To judge the risk of measurement bias related to how PF was measured (differential measurement of PF related to the level of outcome). |  |  |
| Definition of the PF | A clear definition or description of 'PF' is provided (e.g., including dose, level, duration of exposure, and clear specification of the method of measurement). | Partial |  |
| Valid and Reliable Measurement of PF | Method of PF measurement is adequately valid and reliable to limit misclassification bias (e.g., may include relevant outside sources of information on measurement properties, also characteristics, such as blind measurement and limited reliance on recall)./ Continuous variables are reported or appropriate cut-points (i.e., not data-dependent) are used. | Yes |  |
| Method and Setting of PF Measurement | The method and setting of measurement of PF is the same for all study participants. | Yes |  |
| Proportion of data on PF available for analysis | Adequate proportion of the study sample has complete data for PF variable. | Yes |  |
| Method used for missing data | Appropriate methods of imputation are used for missing 'PF' data. | No |  |
| **PF Measurement Summary** | PF is adequately measured in study participants to sufficiently limit potential bias. |  | Low |
|  | | | |
| 4. Outcome Measurement | Goal: To judge the risk of bias related to the measurement of outcome (differential measurement of outcome related to the baseline level of PF). |  |  |
| Definition of the Outcome | A clear definition of outcome is provided, including duration of follow-up and level and extent of the outcome construct. | Yes |  |
| Valid and Reliable Measurement of Outcome | The method of outcome measurement used is adequately valid and reliable to limit misclassification bias (e.g., may include relevant outside sources of information on measurement properties, also characteristics, such as blind measurement and confirmation of outcome with valid and reliable test). | Yes |  |
| Method and Setting of Outcome Measurement | The method and setting of outcome measurement is the same for all study participants. | Yes |  |
| **Outcome Measurement Summary** | Outcome of interest is adequately measured in study participants to sufficiently limit potential bias. |  | Low |
|  | | | |
| **5. Study Confounding** | Goal: To judge the risk of bias due to confounding (i.e. the effect of PF is distorted by another factor that is related to PF and outcome). |  |  |
| Important Confounders Measured | All important confounders, including treatments (key variables in conceptual model: LIST), are measured. | Partial |  |
| Definition of the confounding factor | Clear definitions of the important confounders measured are provided (e.g., including dose, level, and duration of exposures). | Partial |  |
| Valid and Reliable Measurement of Confounders | Measurement of all important confounders is adequately valid and reliable (e.g., may include relevant outside sources of information on measurement properties, also characteristics, such as blind measurement and limited reliance on recall). | Yes |  |
| Method and Setting of Confounding Measurement | The method and setting of confounding measurement are the same for all study participants. | Yes |  |
| Method used for missing data | Appropriate methods are used if imputation is used for missing confounder data. | No |  |
| Appropriate Accounting for Confounding | Important potential confounders are accounted for in the study design (e.g., matching for key variables, stratification, or initial assembly of comparable groups). /Important potential confounders are accounted for in the analysis (i.e., appropriate adjustment). | Partial |  |
| **Study Confounding Summary** | Important potential confounders are appropriately accounted for, limiting potential bias with respect to the relationship between PF and outcome . |  | Moderate |
|  | | | |
| **6. Statistical Analysis and Reporting** | Goal: To judge the risk of bias related to the statistical analysis and presentation of results. |  |  |
| Presentation of analytical strategy | There is sufficient presentation of data to assess the adequacy of the analysis. | Yes |  |
| Model development strategy | The strategy for model building (i.e., inclusion of variables in the statistical model) is appropriate and is based on a conceptual framework or model./ The selected statistical model is adequate for the design of the study. | Partial |  |
| Reporting of results | There is no selective reporting of results. | Unsure |  |
| **Statistical Analysis and Presentation Summary** | The statistical analysis is appropriate for the design of the study, limiting potential for presentation of invalid or spurious results. |  | Low |
|  | | | |
| Study title: | Predictors of outcome in HSV encephalitis |  |  |
| Study author/ data: | Singh et al., 2016 |  |  |
| Study identifier: | 10.1007/s00415-015-7960-8 |  |  |
|  | | | |
| Biases | Issues to consider for judging overall rating of "Risk of bias" | Rating of reporting | Rating of "Risk of bias" |
| **1. Study Participation** | Goal: To judge the risk of selection bias (likelihood that relationship between PF and outcome is different for participants and eligible non-participants). |  |  |
| Source of target population | The source population or population of interest is adequately described for key characteristics (LIST). | Yes |  |
| Method used to identify population | The sampling frame and recruitment are adequately described, including methods to identify the sample sufficient to limit potential bias (number and type used, e.g., referral patterns in health care) | Partial |  |
| Recruitment period | Period of recruitment is adequately described | Yes |  |
| Place of recruitment | Place of recruitment (setting and geographic location) are adequately described | Yes |  |
| Inclusion and exclusion criteria | Inclusion and exclusion criteria are adequately described (e.g., including explicit diagnostic criteria or “zero time” description). | Yes |  |
| Adequate study participation | There is adequate participation in the study by eligible individuals | Yes |  |
| Baseline characteristics | The baseline study sample (i.e., individuals entering the study) is adequately described for key characteristics (LIST). | Partial |  |
| **Summary Study participation** | The study sample represents the population of interest on key characteristics, sufficient to limit potential bias of the observed relationship between PF and outcome. |  | Low |
|  | | | |
| **2. Study Attrition** | Goal: To judge the risk of attrition bias (likelihood that relationship between PF and outcome are different for completing and non-completing participants). |  |  |
| Proportion of baseline sample available for analysis | Response rate (i.e., proportion of study sample completing the study and providing outcome data) is adequate. | Yes |  |
| Attempts to collect information on participants who dropped out | Attempts to collect information on participants who dropped out of the study are described. | Yes |  |
| Reasons and potential impact of subjects lost to follow-up | Reasons for loss to follow-up are provided. | No |  |
| Outcome and prognostic factor information on those lost to follow-up | Participants lost to follow-up are adequately described for key characteristics (LIST)./ There are no important differences between key characteristics (LIST) and outcomes in participants who completed the study and those who did not. | Partial |  |
| **Study Attrition Summary** | Loss to follow-up (from baseline sample to study population analyzed) is not associated with key characteristics (i.e., the study data adequately represent the sample) sufficient to limit potential bias to the observed relationship between PF and outcome. |  | Low |
|  | | | |
| **3. Prognostic Factor Measurement** | Goal: To judge the risk of measurement bias related to how PF was measured (differential measurement of PF related to the level of outcome). |  |  |
| Definition of the PF | A clear definition or description of 'PF' is provided (e.g., including dose, level, duration of exposure, and clear specification of the method of measurement). | Yes |  |
| Valid and Reliable Measurement of PF | Method of PF measurement is adequately valid and reliable to limit misclassification bias (e.g., may include relevant outside sources of information on measurement properties, also characteristics, such as blind measurement and limited reliance on recall)./ Continuous variables are reported or appropriate cut-points (i.e., not data-dependent) are used. | Yes |  |
| Method and Setting of PF Measurement | The method and setting of measurement of PF is the same for all study participants. | Yes |  |
| Proportion of data on PF available for analysis | Adequate proportion of the study sample has complete data for PF variable. | Yes |  |
| Method used for missing data | Appropriate methods of imputation are used for missing 'PF' data. | No |  |
| **PF Measurement Summary** | PF is adequately measured in study participants to sufficiently limit potential bias. |  | Low |
|  | | | |
| 4. Outcome Measurement | Goal: To judge the risk of bias related to the measurement of outcome (differential measurement of outcome related to the baseline level of PF). |  |  |
| Definition of the Outcome | A clear definition of outcome is provided, including duration of follow-up and level and extent of the outcome construct. | Yes |  |
| Valid and Reliable Measurement of Outcome | The method of outcome measurement used is adequately valid and reliable to limit misclassification bias (e.g., may include relevant outside sources of information on measurement properties, also characteristics, such as blind measurement and confirmation of outcome with valid and reliable test). | Yes |  |
| Method and Setting of Outcome Measurement | The method and setting of outcome measurement is the same for all study participants. | Yes |  |
| **Outcome Measurement Summary** | Outcome of interest is adequately measured in study participants to sufficiently limit potential bias. |  | Low |
|  | | | |
| **5. Study Confounding** | Goal: To judge the risk of bias due to confounding (i.e. the effect of PF is distorted by another factor that is related to PF and outcome). |  |  |
| Important Confounders Measured | All important confounders, including treatments (key variables in conceptual model: LIST), are measured. | Partial |  |
| Definition of the confounding factor | Clear definitions of the important confounders measured are provided (e.g., including dose, level, and duration of exposures). | Yes |  |
| Valid and Reliable Measurement of Confounders | Measurement of all important confounders is adequately valid and reliable (e.g., may include relevant outside sources of information on measurement properties, also characteristics, such as blind measurement and limited reliance on recall). | Partial |  |
| Method and Setting of Confounding Measurement | The method and setting of confounding measurement are the same for all study participants. | Yes |  |
| Method used for missing data | Appropriate methods are used if imputation is used for missing confounder data. | No |  |
| Appropriate Accounting for Confounding | Important potential confounders are accounted for in the study design (e.g., matching for key variables, stratification, or initial assembly of comparable groups). /Important potential confounders are accounted for in the analysis (i.e., appropriate adjustment). | Partial |  |
| **Study Confounding Summary** | Important potential confounders are appropriately accounted for, limiting potential bias with respect to the relationship between PF and outcome . |  | Low |
|  | | | |
| **6. Statistical Analysis and Reporting** | Goal: To judge the risk of bias related to the statistical analysis and presentation of results. |  |  |
| Presentation of analytical strategy | There is sufficient presentation of data to assess the adequacy of the analysis. | Yes |  |
| Model development strategy | The strategy for model building (i.e., inclusion of variables in the statistical model) is appropriate and is based on a conceptual framework or model./ The selected statistical model is adequate for the design of the study. | Yes |  |
| Reporting of results | There is no selective reporting of results. | Yes |  |
| **Statistical Analysis and Presentation Summary** | The statistical analysis is appropriate for the design of the study, limiting potential for presentation of invalid or spurious results. |  | Low |
|  | | | |
| Study title: | Elevated levels of cerebrospinal fluid S100B are associated with brain injury and unfavorable outcomes in children with central nervous system infections | | |
| Study author/ data: | Peng et al., 2017 |  |  |
| Study identifier: | 10.3109/00207454.2015.1135334 |  |  |
|  | | | |
| Biases | Issues to consider for judging overall rating of "Risk of bias" | Rating of reporting | Rating of "Risk of bias" |
| **1. Study Participation** | Goal: To judge the risk of selection bias (likelihood that relationship between PF and outcome is different for participants and eligible non-participants). |  |  |
| Source of target population | The source population or population of interest is adequately described for key characteristics (LIST). | No |  |
| Method used to identify population | The sampling frame and recruitment are adequately described, including methods to identify the sample sufficient to limit potential bias (number and type used, e.g., referral patterns in health care) | Partial |  |
| Recruitment period | Period of recruitment is adequately described | Yes |  |
| Place of recruitment | Place of recruitment (setting and geographic location) are adequately described | Yes |  |
| Inclusion and exclusion criteria | Inclusion and exclusion criteria are adequately described (e.g., including explicit diagnostic criteria or “zero time” description). | Partial |  |
| Adequate study participation | There is adequate participation in the study by eligible individuals | Yes |  |
| Baseline characteristics | The baseline study sample (i.e., individuals entering the study) is adequately described for key characteristics (LIST). | Partial |  |
| **Summary Study participation** | The study sample represents the population of interest on key characteristics, sufficient to limit potential bias of the observed relationship between PF and outcome. |  | Moderate |
|  | | | |
| **2. Study Attrition** | Goal: To judge the risk of attrition bias (likelihood that relationship between PF and outcome are different for completing and non-completing participants). |  |  |
| Proportion of baseline sample available for analysis | Response rate (i.e., proportion of study sample completing the study and providing outcome data) is adequate. | Yes |  |
| Attempts to collect information on participants who dropped out | Attempts to collect information on participants who dropped out of the study are described. | No |  |
| Reasons and potential impact of subjects lost to follow-up | Reasons for loss to follow-up are provided. | No |  |
| Outcome and prognostic factor information on those lost to follow-up | Participants lost to follow-up are adequately described for key characteristics (LIST)./ There are no important differences between key characteristics (LIST) and outcomes in participants who completed the study and those who did not. | No |  |
| **Study Attrition Summary** | Loss to follow-up (from baseline sample to study population analyzed) is not associated with key characteristics (i.e., the study data adequately represent the sample) sufficient to limit potential bias to the observed relationship between PF and outcome. |  | Low |
|  | | | |
| **3. Prognostic Factor Measurement** | Goal: To judge the risk of measurement bias related to how PF was measured (differential measurement of PF related to the level of outcome). |  |  |
| Definition of the PF | A clear definition or description of 'PF' is provided (e.g., including dose, level, duration of exposure, and clear specification of the method of measurement). | Yes |  |
| Valid and Reliable Measurement of PF | Method of PF measurement is adequately valid and reliable to limit misclassification bias (e.g., may include relevant outside sources of information on measurement properties, also characteristics, such as blind measurement and limited reliance on recall)./ Continuous variables are reported or appropriate cut-points (i.e., not data-dependent) are used. | Yes |  |
| Method and Setting of PF Measurement | The method and setting of measurement of PF is the same for all study participants. | Yes |  |
| Proportion of data on PF available for analysis | Adequate proportion of the study sample has complete data for PF variable. | Yes |  |
| Method used for missing data | Appropriate methods of imputation are used for missing 'PF' data. | No |  |
| **PF Measurement Summary** | PF is adequately measured in study participants to sufficiently limit potential bias. |  | Low |
|  | | | |
| 4. Outcome Measurement | Goal: To judge the risk of bias related to the measurement of outcome (differential measurement of outcome related to the baseline level of PF). |  |  |
| Definition of the Outcome | A clear definition of outcome is provided, including duration of follow-up and level and extent of the outcome construct. | Yes |  |
| Valid and Reliable Measurement of Outcome | The method of outcome measurement used is adequately valid and reliable to limit misclassification bias (e.g., may include relevant outside sources of information on measurement properties, also characteristics, such as blind measurement and confirmation of outcome with valid and reliable test). | Yes |  |
| Method and Setting of Outcome Measurement | The method and setting of outcome measurement is the same for all study participants. | Yes |  |
| **Outcome Measurement Summary** | Outcome of interest is adequately measured in study participants to sufficiently limit potential bias. |  | Low |
|  | | | |
| **5. Study Confounding** | Goal: To judge the risk of bias due to confounding (i.e. the effect of PF is distorted by another factor that is related to PF and outcome). |  |  |
| Important Confounders Measured | All important confounders, including treatments (key variables in conceptual model: LIST), are measured. | Yes |  |
| Definition of the confounding factor | Clear definitions of the important confounders measured are provided (e.g., including dose, level, and duration of exposures). | Partial |  |
| Valid and Reliable Measurement of Confounders | Measurement of all important confounders is adequately valid and reliable (e.g., may include relevant outside sources of information on measurement properties, also characteristics, such as blind measurement and limited reliance on recall). | Yes |  |
| Method and Setting of Confounding Measurement | The method and setting of confounding measurement are the same for all study participants. | Yes |  |
| Method used for missing data | Appropriate methods are used if imputation is used for missing confounder data. | No |  |
| Appropriate Accounting for Confounding | Important potential confounders are accounted for in the study design (e.g., matching for key variables, stratification, or initial assembly of comparable groups). /Important potential confounders are accounted for in the analysis (i.e., appropriate adjustment). | Yes |  |
| **Study Confounding Summary** | Important potential confounders are appropriately accounted for, limiting potential bias with respect to the relationship between PF and outcome . |  | Low |
|  | | | |
| **6. Statistical Analysis and Reporting** | Goal: To judge the risk of bias related to the statistical analysis and presentation of results. |  |  |
| Presentation of analytical strategy | There is sufficient presentation of data to assess the adequacy of the analysis. | Yes |  |
| Model development strategy | The strategy for model building (i.e., inclusion of variables in the statistical model) is appropriate and is based on a conceptual framework or model./ The selected statistical model is adequate for the design of the study. | Yes |  |
| Reporting of results | There is no selective reporting of results. | Yes |  |
| **Statistical Analysis and Presentation Summary** | The statistical analysis is appropriate for the design of the study, limiting potential for presentation of invalid or spurious results. |  | Low |
|  | | | |
| Study title: | Prognostic Factors Among Children With Acute Encephalitis/Encephalopathy Associated With Viral and Other Pathogens | | |
| Study author/ data: | Hatachi et al., 2021 | | |
| Study identifier: | 10.1093/cid/ciaa536 | | |
|  | | | |
| Biases | Issues to consider for judging overall rating of "Risk of bias" | Rating of reporting | Rating of "Risk of bias" |
| **1. Study Participation** | Goal: To judge the risk of selection bias (likelihood that relationship between PF and outcome is different for participants and eligible non-participants). |  |  |
| Source of target population | The source population or population of interest is adequately described for key characteristics (LIST). | Yes |  |
| Method used to identify population | The sampling frame and recruitment are adequately described, including methods to identify the sample sufficient to limit potential bias (number and type used, e.g., referral patterns in health care) | Partial |  |
| Recruitment period | Period of recruitment is adequately described | Yes |  |
| Place of recruitment | Place of recruitment (setting and geographic location) are adequately described | Yes |  |
| Inclusion and exclusion criteria | Inclusion and exclusion criteria are adequately described (e.g., including explicit diagnostic criteria or “zero time” description). | Yes |  |
| Adequate study participation | There is adequate participation in the study by eligible individuals | Yes |  |
| Baseline characteristics | The baseline study sample (i.e., individuals entering the study) is adequately described for key characteristics (LIST). | Partial |  |
| **Summary Study participation** | The study sample represents the population of interest on key characteristics, sufficient to limit potential bias of the observed relationship between PF and outcome. |  | Low |
|  | | | |
| **2. Study Attrition** | Goal: To judge the risk of attrition bias (likelihood that relationship between PF and outcome are different for completing and non-completing participants). |  |  |
| Proportion of baseline sample available for analysis | Response rate (i.e., proportion of study sample completing the study and providing outcome data) is adequate. | Yes |  |
| Attempts to collect information on participants who dropped out | Attempts to collect information on participants who dropped out of the study are described. | No |  |
| Reasons and potential impact of subjects lost to follow-up | Reasons for loss to follow-up are provided. | No |  |
| Outcome and prognostic factor information on those lost to follow-up | Participants lost to follow-up are adequately described for key characteristics (LIST)./ There are no important differences between key characteristics (LIST) and outcomes in participants who completed the study and those who did not. | No |  |
| **Study Attrition Summary** | Loss to follow-up (from baseline sample to study population analyzed) is not associated with key characteristics (i.e., the study data adequately represent the sample) sufficient to limit potential bias to the observed relationship between PF and outcome. |  | Low |
|  | | | |
| **3. Prognostic Factor Measurement** | Goal: To judge the risk of measurement bias related to how PF was measured (differential measurement of PF related to the level of outcome). |  |  |
| Definition of the PF | A clear definition or description of 'PF' is provided (e.g., including dose, level, duration of exposure, and clear specification of the method of measurement). | Partial |  |
| Valid and Reliable Measurement of PF | Method of PF measurement is adequately valid and reliable to limit misclassification bias (e.g., may include relevant outside sources of information on measurement properties, also characteristics, such as blind measurement and limited reliance on recall)./ Continuous variables are reported or appropriate cut-points (i.e., not data-dependent) are used. | Yes |  |
| Method and Setting of PF Measurement | The method and setting of measurement of PF is the same for all study participants. | Yes |  |
| Proportion of data on PF available for analysis | Adequate proportion of the study sample has complete data for PF variable. | Yes |  |
| Method used for missing data | Appropriate methods of imputation are used for missing 'PF' data. | No |  |
| **PF Measurement Summary** | PF is adequately measured in study participants to sufficiently limit potential bias. |  | Low |
|  | | | |
| 4. Outcome Measurement | Goal: To judge the risk of bias related to the measurement of outcome (differential measurement of outcome related to the baseline level of PF). |  |  |
| Definition of the Outcome | A clear definition of outcome is provided, including duration of follow-up and level and extent of the outcome conYesstruct. | Yes |  |
| Valid and Reliable Measurement of Outcome | The method of outcome measurement used is adequately valid and reliable to limit misclassification bias (e.g., may include relevant outside sources of information on measurement properties, also characteristics, such as blind measurement and confirmation of outcome with valid and reliable test). | Partial |  |
| Method and Setting of Outcome Measurement | The method and setting of outcome measurement is the same for all study participants. | Yes |  |
| **Outcome Measurement Summary** | Outcome of interest is adequately measured in study participants to sufficiently limit potential bias. |  | Low |
|  | | | |
| **5. Study Confounding** | Goal: To judge the risk of bias due to confounding (i.e. the effect of PF is distorted by another factor that is related to PF and outcome). |  |  |
| Important Confounders Measured | All important confounders, including treatments (key variables in conceptual model: LIST), are measured. | Partial |  |
| Definition of the confounding factor | Clear definitions of the important confounders measured are provided (e.g., including dose, level, and duration of exposures). | Yes |  |
| Valid and Reliable Measurement of Confounders | Measurement of all important confounders is adequately valid and reliable (e.g., may include relevant outside sources of information on measurement properties, also characteristics, such as blind measurement and limited reliance on recall). | Yes |  |
| Method and Setting of Confounding Measurement | The method and setting of confounding measurement are the same for all study participants. | Yes |  |
| Method used for missing data | Appropriate methods are used if imputation is used for missing confounder data. | No |  |
| Appropriate Accounting for Confounding | Important potential confounders are accounted for in the study design (e.g., matching for key variables, stratification, or initial assembly of comparable groups). /Important potential confounders are accounted for in the analysis (i.e., appropriate adjustment). | Yes |  |
| **Study Confounding Summary** | Important potential confounders are appropriately accounted for, limiting potential bias with respect to the relationship between PF and outcome . |  | Low |
|  | | | |
| **6. Statistical Analysis and Reporting** | Goal: To judge the risk of bias related to the statistical analysis and presentation of results. |  |  |
| Presentation of analytical strategy | There is sufficient presentation of data to assess the adequacy of the analysis. | Yes |  |
| Model development strategy | The strategy for model building (i.e., inclusion of variables in the statistical model) is appropriate and is based on a conceptual framework or model./ The selected statistical model is adequate for the design of the study. | Yes |  |
| Reporting of results | There is no selective reporting of results. | Yes |  |
| **Statistical Analysis and Presentation Summary** | The statistical analysis is appropriate for the design of the study, limiting potential for presentation of invalid or spurious results. |  | Low |
|  | | | |
| Study title: | Predictive Factors of Short-Term Neurologic  Outcome in Children With Encephalitis | | |
| Study author/ data: | Klein et al., 1994 |  |  |
| Study identifier: | 10.1016/0887-8994%2894%2990007-8 |  |  |
|  |  |  |  |
| Biases | Issues to consider for judging overall rating of "Risk of bias" | Rating of reporting | Rating of "Risk of bias" |
| **1. Study Participation** | Goal: To judge the risk of selection bias (likelihood that relationship between PF and outcome is different for participants and eligible non-participants). |  |  |
| Source of target population | The source population or population of interest is adequately described for key characteristics (LIST). | No |  |
| Method used to identify population | The sampling frame and recruitment are adequately described, including methods to identify the sample sufficient to limit potential bias (number and type used, e.g., referral patterns in health care) | Partial |  |
| Recruitment period | Period of recruitment is adequately described | Partial |  |
| Place of recruitment | Place of recruitment (setting and geographic location) are adequately described | Yes |  |
| Inclusion and exclusion criteria | Inclusion and exclusion criteria are adequately described (e.g., including explicit diagnostic criteria or “zero time” description). | Partial |  |
| Adequate study participation | There is adequate participation in the study by eligible individuals | Yes |  |
| Baseline characteristics | The baseline study sample (i.e., individuals entering the study) is adequately described for key characteristics (LIST). | No |  |
| **Summary Study participation** | The study sample represents the population of interest on key characteristics, sufficient to limit potential bias of the observed relationship between PF and outcome. |  | Moderate |
|  | | | |
| **2. Study Attrition** | Goal: To judge the risk of attrition bias (likelihood that relationship between PF and outcome are different for completing and non-completing participants). |  |  |
| Proportion of baseline sample available for analysis | Response rate (i.e., proportion of study sample completing the study and providing outcome data) is adequate. | No |  |
| Attempts to collect information on participants who dropped out | Attempts to collect information on participants who dropped out of the study are described. | No |  |
| Reasons and potential impact of subjects lost to follow-up | Reasons for loss to follow-up are provided. | No |  |
| Outcome and prognostic factor information on those lost to follow-up | Participants lost to follow-up are adequately described for key characteristics (LIST)./ There are no important differences between key characteristics (LIST) and outcomes in participants who completed the study and those who did not. | No |  |
| **Study Attrition Summary** | Loss to follow-up (from baseline sample to study population analyzed) is not associated with key characteristics (i.e., the study data adequately represent the sample) sufficient to limit potential bias to the observed relationship between PF and outcome. |  | Moderate |
|  | | | |
| **3. Prognostic Factor Measurement** | Goal: To judge the risk of measurement bias related to how PF was measured (differential measurement of PF related to the level of outcome). |  |  |
| Definition of the PF | A clear definition or description of 'PF' is provided (e.g., including dose, level, duration of exposure, and clear specification of the method of measurement). | Partial |  |
| Valid and Reliable Measurement of PF | Method of PF measurement is adequately valid and reliable to limit misclassification bias (e.g., may include relevant outside sources of information on measurement properties, also characteristics, such as blind measurement and limited reliance on recall)./ Continuous variables are reported or appropriate cut-points (i.e., not data-dependent) are used. | Unsure |  |
| Method and Setting of PF Measurement | The method and setting of measurement of PF is the same for all study participants. | Yes |  |
| Proportion of data on PF available for analysis | Adequate proportion of the study sample has complete data for PF variable. | No |  |
| Method used for missing data | Appropriate methods of imputation are used for missing 'PF' data. | No |  |
| **PF Measurement Summary** | PF is adequately measured in study participants to sufficiently limit potential bias. |  | Moderate |
|  | | | |
| 4. Outcome Measurement | Goal: To judge the risk of bias related to the measurement of outcome (differential measurement of outcome related to the baseline level of PF). |  |  |
| Definition of the Outcome | A clear definition of outcome is provided, including duration of follow-up and level and extent of the outcome construct. | Partial |  |
| Valid and Reliable Measurement of Outcome | The method of outcome measurement used is adequately valid and reliable to limit misclassification bias (e.g., may include relevant outside sources of information on measurement properties, also characteristics, such as blind measurement and confirmation of outcome with valid and reliable test). | No |  |
| Method and Setting of Outcome Measurement | The method and setting of outcome measurement is the same for all study participants. | Unsure |  |
| **Outcome Measurement Summary** | Outcome of interest is adequately measured in study participants to sufficiently limit potential bias. |  | High |
|  | | | |
| **5. Study Confounding** | Goal: To judge the risk of bias due to confounding (i.e. the effect of PF is distorted by another factor that is related to PF and outcome). |  |  |
| Important Confounders Measured | All important confounders, including treatments (key variables in conceptual model: LIST), are measured. | Partial |  |
| Definition of the confounding factor | Clear definitions of the important confounders measured are provided (e.g., including dose, level, and duration of exposures). | No |  |
| Valid and Reliable Measurement of Confounders | Measurement of all important confounders is adequately valid and reliable (e.g., may include relevant outside sources of information on measurement properties, also characteristics, such as blind measurement and limited reliance on recall). | Partial |  |
| Method and Setting of Confounding Measurement | The method and setting of confounding measurement are the same for all study participants. | Partial |  |
| Method used for missing data | Appropriate methods are used if imputation is used for missing confounder data. | No |  |
| Appropriate Accounting for Confounding | Important potential confounders are accounted for in the study design (e.g., matching for key variables, stratification, or initial assembly of comparable groups). /Important potential confounders are accounted for in the analysis (i.e., appropriate adjustment). | Partial |  |
| **Study Confounding Summary** | Important potential confounders are appropriately accounted for, limiting potential bias with respect to the relationship between PF and outcome . |  | Moderate |
|  | | | |
| **6. Statistical Analysis and Reporting** | Goal: To judge the risk of bias related to the statistical analysis and presentation of results. |  |  |
| Presentation of analytical strategy | There is sufficient presentation of data to assess the adequacy of the analysis. | Yes |  |
| Model development strategy | The strategy for model building (i.e., inclusion of variables in the statistical model) is appropriate and is based on a conceptual framework or model./ The selected statistical model is adequate for the design of the study. | Yes |  |
| Reporting of results | There is no selective reporting of results. | Unsure |  |
| **Statistical Analysis and Presentation Summary** | The statistical analysis is appropriate for the design of the study, limiting potential for presentation of invalid or spurious results. |  | Low |
|  | | | |
| Study title: | The Epidemiology, Clinical Features, and Long-Term Prognosis of Japanese Encephalitis in Central Sarawak, Malaysia, 1997–2005 | | |
| Study author/ data: | Ooi et al., 2008 | | |
| Study identifier: | 10.1086/590008 | | |
|  | | | |
| Biases | Issues to consider for judging overall rating of "Risk of bias" | Rating of reporting | Rating of "Risk of bias" |
| **1. Study Participation** | Goal: To judge the risk of selection bias (likelihood that relationship between PF and outcome is different for participants and eligible non-participants). |  |  |
| Source of target population | The source population or population of interest is adequately described for key characteristics (LIST). | Partial |  |
| Method used to identify population | The sampling frame and recruitment are adequately described, including methods to identify the sample sufficient to limit potential bias (number and type used, e.g., referral patterns in health care) | Partial |  |
| Recruitment period | Period of recruitment is adequately described | Yes |  |
| Place of recruitment | Place of recruitment (setting and geographic location) are adequately described | Yes |  |
| Inclusion and exclusion criteria | Inclusion and exclusion criteria are adequately described (e.g., including explicit diagnostic criteria or “zero time” description). | Partial |  |
| Adequate study participation | There is adequate participation in the study by eligible individuals | Yes |  |
| Baseline characteristics | The baseline study sample (i.e., individuals entering the study) is adequately described for key characteristics (LIST). | Partial |  |
| **Summary Study participation** | The study sample represents the population of interest on key characteristics, sufficient to limit potential bias of the observed relationship between PF and outcome. |  | Moderate |
|  | | | |
| **2. Study Attrition** | Goal: To judge the risk of attrition bias (likelihood that relationship between PF and outcome are different for completing and non-completing participants). |  |  |
| Proportion of baseline sample available for analysis | Response rate (i.e., proportion of study sample completing the study and providing outcome data) is adequate. | Yes |  |
| Attempts to collect information on participants who dropped out | Attempts to collect information on participants who dropped out of the study are described. | No |  |
| Reasons and potential impact of subjects lost to follow-up | Reasons for loss to follow-up are provided. | No |  |
| Outcome and prognostic factor information on those lost to follow-up | Participants lost to follow-up are adequately described for key characteristics (LIST)./ There are no important differences between key characteristics (LIST) and outcomes in participants who completed the study and those who did not. | No |  |
| **Study Attrition Summary** | Loss to follow-up (from baseline sample to study population analyzed) is not associated with key characteristics (i.e., the study data adequately represent the sample) sufficient to limit potential bias to the observed relationship between PF and outcome. |  | Low |
|  | | | |
| **3. Prognostic Factor Measurement** | Goal: To judge the risk of measurement bias related to how PF was measured (differential measurement of PF related to the level of outcome). |  |  |
| Definition of the PF | A clear definition or description of 'PF' is provided (e.g., including dose, level, duration of exposure, and clear specification of the method of measurement). | Partial |  |
| Valid and Reliable Measurement of PF | Method of PF measurement is adequately valid and reliable to limit misclassification bias (e.g., may include relevant outside sources of information on measurement properties, also characteristics, such as blind measurement and limited reliance on recall)./ Continuous variables are reported or appropriate cut-points (i.e., not data-dependent) are used. | Partial |  |
| Method and Setting of PF Measurement | The method and setting of measurement of PF is the same for all study participants. | Yes |  |
| Proportion of data on PF available for analysis | Adequate proportion of the study sample has complete data for PF variable. | Yes |  |
| Method used for missing data | Appropriate methods of imputation are used for missing 'PF' data. | No |  |
| **PF Measurement Summary** | PF is adequately measured in study participants to sufficiently limit potential bias. |  | Moderate |
|  | | | |
| 4. Outcome Measurement | Goal: To judge the risk of bias related to the measurement of outcome (differential measurement of outcome related to the baseline level of PF). |  |  |
| Definition of the Outcome | A clear definition of outcome is provided, including duration of follow-up and level and extent of the outcome construct. | Yes |  |
| Valid and Reliable Measurement of Outcome | The method of outcome measurement used is adequately valid and reliable to limit misclassification bias (e.g., may include relevant outside sources of information on measurement properties, also characteristics, such as blind measurement and confirmation of outcome with valid and reliable test). | Yes |  |
| Method and Setting of Outcome Measurement | The method and setting of outcome measurement is the same for all study participants. | Yes |  |
| **Outcome Measurement Summary** | Outcome of interest is adequately measured in study participants to sufficiently limit potential bias. |  | Low |
|  | | | |
| **5. Study Confounding** | Goal: To judge the risk of bias due to confounding (i.e. the effect of PF is distorted by another factor that is related to PF and outcome). |  |  |
| Important Confounders Measured | All important confounders, including treatments (key variables in conceptual model: LIST), are measured. | Partial |  |
| Definition of the confounding factor | Clear definitions of the important confounders measured are provided (e.g., including dose, level, and duration of exposures). | Partial |  |
| Valid and Reliable Measurement of Confounders | Measurement of all important confounders is adequately valid and reliable (e.g., may include relevant outside sources of information on measurement properties, also characteristics, such as blind measurement and limited reliance on recall). | Partial |  |
| Method and Setting of Confounding Measurement | The method and setting of confounding measurement are the same for all study participants. | Yes |  |
| Method used for missing data | Appropriate methods are used if imputation is used for missing confounder data. | Unsure |  |
| Appropriate Accounting for Confounding | Important potential confounders are accounted for in the study design (e.g., matching for key variables, stratification, or initial assembly of comparable groups). /Important potential confounders are accounted for in the analysis (i.e., appropriate adjustment). | Yes |  |
| **Study Confounding Summary** | Important potential confounders are appropriately accounted for, limiting potential bias with respect to the relationship between PF and outcome . |  | Low |
|  | | | |
| **6. Statistical Analysis and Reporting** | Goal: To judge the risk of bias related to the statistical analysis and presentation of results. |  |  |
| Presentation of analytical strategy | There is sufficient presentation of data to assess the adequacy of the analysis. | Yes |  |
| Model development strategy | The strategy for model building (i.e., inclusion of variables in the statistical model) is appropriate and is based on a conceptual framework or model./ The selected statistical model is adequate for the design of the study. | Yes |  |
| Reporting of results | There is no selective reporting of results. | Yes |  |
| **Statistical Analysis and Presentation Summary** | The statistical analysis is appropriate for the design of the study, limiting potential for presentation of invalid or spurious results. |  | Low |
|  | | | |
| Study title: | Influence of Malnutrition on Adverse Outcome in Children with Confirmed or Probable Viral Encephalitis: A Prospective Observational Study | | |
| Study author/ data: | Singh et al., 2015 | | |
| Study identifier: | 10.1155/2015/407473 | | |
|  | | | |
| Biases | Issues to consider for judging overall rating of "Risk of bias" | Rating of reporting | Rating of "Risk of bias" |
| **1. Study Participation** | Goal: To judge the risk of selection bias (likelihood that relationship between PF and outcome is different for participants and eligible non-participants). |  |  |
| Source of target population | The source population or population of interest is adequately described for key characteristics (LIST). | Partial |  |
| Method used to identify population | The sampling frame and recruitment are adequately described, including methods to identify the sample sufficient to limit potential bias (number and type used, e.g., referral patterns in health care) | Partial |  |
| Recruitment period | Period of recruitment is adequately described | Yes |  |
| Place of recruitment | Place of recruitment (setting and geographic location) are adequately described | Yes |  |
| Inclusion and exclusion criteria | Inclusion and exclusion criteria are adequately described (e.g., including explicit diagnostic criteria or “zero time” description). | Partial |  |
| Adequate study participation | There is adequate participation in the study by eligible individuals | Yes |  |
| Baseline characteristics | The baseline study sample (i.e., individuals entering the study) is adequately described for key characteristics (LIST). | Partial |  |
| **Summary Study participation** | The study sample represents the population of interest on key characteristics, sufficient to limit potential bias of the observed relationship between PF and outcome. |  | Low |
|  | | | |
| **2. Study Attrition** | Goal: To judge the risk of attrition bias (likelihood that relationship between PF and outcome are different for completing and non-completing participants). |  |  |
| Proportion of baseline sample available for analysis | Response rate (i.e., proportion of study sample completing the study and providing outcome data) is adequate. | Yes |  |
| Attempts to collect information on participants who dropped out | Attempts to collect information on participants who dropped out of the study are described. | No |  |
| Reasons and potential impact of subjects lost to follow-up | Reasons for loss to follow-up are provided. | No |  |
| Outcome and prognostic factor information on those lost to follow-up | Participants lost to follow-up are adequately described for key characteristics (LIST)./ There are no important differences between key characteristics (LIST) and outcomes in participants who completed the study and those who did not. | No |  |
| **Study Attrition Summary** | Loss to follow-up (from baseline sample to study population analyzed) is not associated with key characteristics (i.e., the study data adequately represent the sample) sufficient to limit potential bias to the observed relationship between PF and outcome. |  |  |
|  | | | |
| **3. Prognostic Factor Measurement** | Goal: To judge the risk of measurement bias related to how PF was measured (differential measurement of PF related to the level of outcome). |  |  |
| Definition of the PF | A clear definition or description of 'PF' is provided (e.g., including dose, level, duration of exposure, and clear specification of the method of measurement). | No |  |
| Valid and Reliable Measurement of PF | Method of PF measurement is adequately valid and reliable to limit misclassification bias (e.g., may include relevant outside sources of information on measurement properties, also characteristics, such as blind measurement and limited reliance on recall)./ Continuous variables are reported or appropriate cut-points (i.e., not data-dependent) are used. | Partial |  |
| Method and Setting of PF Measurement | The method and setting of measurement of PF is the same for all study participants. | Yes |  |
| Proportion of data on PF available for analysis | Adequate proportion of the study sample has complete data for PF variable. | Yes |  |
| Method used for missing data | Appropriate methods of imputation are used for missing 'PF' data. | No |  |
| **PF Measurement Summary** | PF is adequately measured in study participants to sufficiently limit potential bias. |  | Moderate |
|  | | | |
| 4. Outcome Measurement | Goal: To judge the risk of bias related to the measurement of outcome (differential measurement of outcome related to the baseline level of PF). |  |  |
| Definition of the Outcome | A clear definition of outcome is provided, including duration of follow-up and level and extent of the outcome construct. | Partial |  |
| Valid and Reliable Measurement of Outcome | The method of outcome measurement used is adequately valid and reliable to limit misclassification bias (e.g., may include relevant outside sources of information on measurement properties, also characteristics, such as blind measurement and confirmation of outcome with valid and reliable test). | Partial |  |
| Method and Setting of Outcome Measurement | The method and setting of outcome measurement is the same for all study participants. | Yes |  |
| **Outcome Measurement Summary** | Outcome of interest is adequately measured in study participants to sufficiently limit potential bias. |  | Moderate |
|  | | | |
| **5. Study Confounding** | Goal: To judge the risk of bias due to confounding (i.e. the effect of PF is distorted by another factor that is related to PF and outcome). |  |  |
| Important Confounders Measured | All important confounders, including treatments (key variables in conceptual model: LIST), are measured. | Partial |  |
| Definition of the confounding factor | Clear definitions of the important confounders measured are provided (e.g., including dose, level, and duration of exposures). | Partial |  |
| Valid and Reliable Measurement of Confounders | Measurement of all important confounders is adequately valid and reliable (e.g., may include relevant outside sources of information on measurement properties, also characteristics, such as blind measurement and limited reliance on recall). | Yes |  |
| Method and Setting of Confounding Measurement | The method and setting of confounding measurement are the same for all study participants. | Yes |  |
| Method used for missing data | Appropriate methods are used if imputation is used for missing confounder data. | No |  |
| Appropriate Accounting for Confounding | Important potential confounders are accounted for in the study design (e.g., matching for key variables, stratification, or initial assembly of comparable groups). /Important potential confounders are accounted for in the analysis (i.e., appropriate adjustment). | Partial |  |
| **Study Confounding Summary** | Important potential confounders are appropriately accounted for, limiting potential bias with respect to the relationship between PF and outcome . |  | Low |
|  | | | |
| **6. Statistical Analysis and Reporting** | Goal: To judge the risk of bias related to the statistical analysis and presentation of results. |  |  |
| Presentation of analytical strategy | There is sufficient presentation of data to assess the adequacy of the analysis. | Yes |  |
| Model development strategy | The strategy for model building (i.e., inclusion of variables in the statistical model) is appropriate and is based on a conceptual framework or model./ The selected statistical model is adequate for the design of the study. | Yes |  |
| Reporting of results | There is no selective reporting of results. | Unsure |  |
| **Statistical Analysis and Presentation Summary** | The statistical analysis is appropriate for the design of the study, limiting potential for presentation of invalid or spurious results. |  | Low |
|  | | | |
| Study title: | Determining the clinical characteristics and prognostic factors for the outcomes of Japanese encephalitis in adults: A multicenter study from southern Taiwan | | |
| Study author/ data: | Lo et al., 2019 | | |
| Study identifier: | 10.1016/j.jmii.2019.08.010 | | |
|  | | | |
| Biases | Issues to consider for judging overall rating of "Risk of bias" | Rating of reporting | Rating of "Risk of bias" |
| **1. Study Participation** | Goal: To judge the risk of selection bias (likelihood that relationship between PF and outcome is different for participants and eligible non-participants). |  |  |
| Source of target population | The source population or population of interest is adequately described for key characteristics (LIST). | Partial |  |
| Method used to identify population | The sampling frame and recruitment are adequately described, including methods to identify the sample sufficient to limit potential bias (number and type used, e.g., referral patterns in health care) | Partial |  |
| Recruitment period | Period of recruitment is adequately described | Yes |  |
| Place of recruitment | Place of recruitment (setting and geographic location) are adequately described | Yes |  |
| Inclusion and exclusion criteria | Inclusion and exclusion criteria are adequately described (e.g., including explicit diagnostic criteria or “zero time” description). | Partial |  |
| Adequate study participation | There is adequate participation in the study by eligible individuals | Yes |  |
| Baseline characteristics | The baseline study sample (i.e., individuals entering the study) is adequately described for key characteristics (LIST). | Yes |  |
| **Summary Study participation** | The study sample represents the population of interest on key characteristics, sufficient to limit potential bias of the observed relationship between PF and outcome. |  | Low |
|  | | | |
| **2. Study Attrition** | Goal: To judge the risk of attrition bias (likelihood that relationship between PF and outcome are different for completing and non-completing participants). |  |  |
| Proportion of baseline sample available for analysis | Response rate (i.e., proportion of study sample completing the study and providing outcome data) is adequate. | Yes |  |
| Attempts to collect information on participants who dropped out | Attempts to collect information on participants who dropped out of the study are described. | No |  |
| Reasons and potential impact of subjects lost to follow-up | Reasons for loss to follow-up are provided. | Yes |  |
| Outcome and prognostic factor information on those lost to follow-up | Participants lost to follow-up are adequately described for key characteristics (LIST)./ There are no important differences between key characteristics (LIST) and outcomes in participants who completed the study and those who did not. | No |  |
| **Study Attrition Summary** | Loss to follow-up (from baseline sample to study population analyzed) is not associated with key characteristics (i.e., the study data adequately represent the sample) sufficient to limit potential bias to the observed relationship between PF and outcome. |  | High |
|  | | | |
| **3. Prognostic Factor Measurement** | Goal: To judge the risk of measurement bias related to how PF was measured (differential measurement of PF related to the level of outcome). |  |  |
| Definition of the PF | A clear definition or description of 'PF' is provided (e.g., including dose, level, duration of exposure, and clear specification of the method of measurement). | Partial |  |
| Valid and Reliable Measurement of PF | Method of PF measurement is adequately valid and reliable to limit misclassification bias (e.g., may include relevant outside sources of information on measurement properties, also characteristics, such as blind measurement and limited reliance on recall)./ Continuous variables are reported or appropriate cut-points (i.e., not data-dependent) are used. | Partial |  |
| Method and Setting of PF Measurement | The method and setting of measurement of PF is the same for all study participants. | No |  |
| Proportion of data on PF available for analysis | Adequate proportion of the study sample has complete data for PF variable. | Yes |  |
| Method used for missing data | Appropriate methods of imputation are used for missing 'PF' data. | No |  |
| **PF Measurement Summary** | PF is adequately measured in study participants to sufficiently limit potential bias. |  | Moderate |
|  | | | |
| 4. Outcome Measurement | Goal: To judge the risk of bias related to the measurement of outcome (differential measurement of outcome related to the baseline level of PF). |  |  |
| Definition of the Outcome | A clear definition of outcome is provided, including duration of follow-up and level and extent of the outcome construct. | Yes |  |
| Valid and Reliable Measurement of Outcome | The method of outcome measurement used is adequately valid and reliable to limit misclassification bias (e.g., may include relevant outside sources of information on measurement properties, also characteristics, such as blind measurement and confirmation of outcome with valid and reliable test). | Yes |  |
| Method and Setting of Outcome Measurement | The method and setting of outcome measurement is the same for all study participants. | No |  |
| **Outcome Measurement Summary** | Outcome of interest is adequately measured in study participants to sufficiently limit potential bias. |  | Moderate |
|  | | | |
| **5. Study Confounding** | Goal: To judge the risk of bias due to confounding (i.e. the effect of PF is distorted by another factor that is related to PF and outcome). |  |  |
| Important Confounders Measured | All important confounders, including treatments (key variables in conceptual model: LIST), are measured. | Yes |  |
| Definition of the confounding factor | Clear definitions of the important confounders measured are provided (e.g., including dose, level, and duration of exposures). | Partial |  |
| Valid and Reliable Measurement of Confounders | Measurement of all important confounders is adequately valid and reliable (e.g., may include relevant outside sources of information on measurement properties, also characteristics, such as blind measurement and limited reliance on recall). | Yes |  |
| Method and Setting of Confounding Measurement | The method and setting of confounding measurement are the same for all study participants. | No |  |
| Method used for missing data | Appropriate methods are used if imputation is used for missing confounder data. | No |  |
| Appropriate Accounting for Confounding | Important potential confounders are accounted for in the study design (e.g., matching for key variables, stratification, or initial assembly of comparable groups). /Important potential confounders are accounted for in the analysis (i.e., appropriate adjustment). | Partial |  |
| **Study Confounding Summary** | Important potential confounders are appropriately accounted for, limiting potential bias with respect to the relationship between PF and outcome . |  | Low |
|  | | | |
| **6. Statistical Analysis and Reporting** | Goal: To judge the risk of bias related to the statistical analysis and presentation of results. |  |  |
| Presentation of analytical strategy | There is sufficient presentation of data to assess the adequacy of the analysis. | Yes |  |
| Model development strategy | The strategy for model building (i.e., inclusion of variables in the statistical model) is appropriate and is based on a conceptual framework or model./ The selected statistical model is adequate for the design of the study. | Yes |  |
| Reporting of results | There is no selective reporting of results. | No |  |
| **Statistical Analysis and Presentation Summary** | The statistical analysis is appropriate for the design of the study, limiting potential for presentation of invalid or spurious results. |  | Low |
|  | | | |
| Study title: | Clinical and immunological risk factors for severe disease in Japanese encephalitis | | |
| Study author/ data: | Libraty et al., 2002 | | |
| Study identifier: | 10.1016/S0035-9203%2802%2990294-4 | | |
|  | | | |
| Biases | Issues to consider for judging overall rating of "Risk of bias" | Rating of reporting | Rating of "Risk of bias" |
| **1. Study Participation** | Goal: To judge the risk of selection bias (likelihood that relationship between PF and outcome is different for participants and eligible non-participants). |  |  |
| Source of target population | The source population or population of interest is adequately described for key characteristics (LIST). | Yes |  |
| Method used to identify population | The sampling frame and recruitment are adequately described, including methods to identify the sample sufficient to limit potential bias (number and type used, e.g., referral patterns in health care) | Partial |  |
| Recruitment period | Period of recruitment is adequately described | Yes |  |
| Place of recruitment | Place of recruitment (setting and geographic location) are adequately described | Yes |  |
| Inclusion and exclusion criteria | Inclusion and exclusion criteria are adequately described (e.g., including explicit diagnostic criteria or “zero time” description). | No |  |
| Adequate study participation | There is adequate participation in the study by eligible individuals | Yes |  |
| Baseline characteristics | The baseline study sample (i.e., individuals entering the study) is adequately described for key characteristics (LIST). | No |  |
| **Summary Study participation** | The study sample represents the population of interest on key characteristics, sufficient to limit potential bias of the observed relationship between PF and outcome. |  | Low |
|  | | | |
| **2. Study Attrition** | Goal: To judge the risk of attrition bias (likelihood that relationship between PF and outcome are different for completing and non-completing participants). |  |  |
| Proportion of baseline sample available for analysis | Response rate (i.e., proportion of study sample completing the study and providing outcome data) is adequate. | Yes |  |
| Attempts to collect information on participants who dropped out | Attempts to collect information on participants who dropped out of the study are described. | No |  |
| Reasons and potential impact of subjects lost to follow-up | Reasons for loss to follow-up are provided. | No |  |
| Outcome and prognostic factor information on those lost to follow-up | Participants lost to follow-up are adequately described for key characteristics (LIST)./ There are no important differences between key characteristics (LIST) and outcomes in participants who completed the study and those who did not. | No |  |
| **Study Attrition Summary** | Loss to follow-up (from baseline sample to study population analyzed) is not associated with key characteristics (i.e., the study data adequately represent the sample) sufficient to limit potential bias to the observed relationship between PF and outcome. |  | Low |
|  | | | |
| **3. Prognostic Factor Measurement** | Goal: To judge the risk of measurement bias related to how PF was measured (differential measurement of PF related to the level of outcome). |  |  |
| Definition of the PF | A clear definition or description of 'PF' is provided (e.g., including dose, level, duration of exposure, and clear specification of the method of measurement). | Partial |  |
| Valid and Reliable Measurement of PF | Method of PF measurement is adequately valid and reliable to limit misclassification bias (e.g., may include relevant outside sources of information on measurement properties, also characteristics, such as blind measurement and limited reliance on recall)./ Continuous variables are reported or appropriate cut-points (i.e., not data-dependent) are used. | Partial |  |
| Method and Setting of PF Measurement | The method and setting of measurement of PF is the same for all study participants. | Yes |  |
| Proportion of data on PF available for analysis | Adequate proportion of the study sample has complete data for PF variable. | Yes |  |
| Method used for missing data | Appropriate methods of imputation are used for missing 'PF' data. | No |  |
| **PF Measurement Summary** | PF is adequately measured in study participants to sufficiently limit potential bias. |  | Moderate |
|  | | | |
| 4. Outcome Measurement | Goal: To judge the risk of bias related to the measurement of outcome (differential measurement of outcome related to the baseline level of PF). |  |  |
| Definition of the Outcome | A clear definition of outcome is provided, including duration of follow-up and level and extent of the outcome construct. | Yes |  |
| Valid and Reliable Measurement of Outcome | The method of outcome measurement used is adequately valid and reliable to limit misclassification bias (e.g., may include relevant outside sources of information on measurement properties, also characteristics, such as blind measurement and confirmation of outcome with valid and reliable test). | Partial |  |
| Method and Setting of Outcome Measurement | The method and setting of outcome measurement is the same for all study participants. | Yes |  |
| **Outcome Measurement Summary** | Outcome of interest is adequately measured in study participants to sufficiently limit potential bias. |  |  |
|  | | | |
| **5. Study Confounding** | Goal: To judge the risk of bias due to confounding (i.e. the effect of PF is distorted by another factor that is related to PF and outcome). |  |  |
| Important Confounders Measured | All important confounders, including treatments (key variables in conceptual model: LIST), are measured. | Yes |  |
| Definition of the confounding factor | Clear definitions of the important confounders measured are provided (e.g., including dose, level, and duration of exposures). | Yes |  |
| Valid and Reliable Measurement of Confounders | Measurement of all important confounders is adequately valid and reliable (e.g., may include relevant outside sources of information on measurement properties, also characteristics, such as blind measurement and limited reliance on recall). | Yes |  |
| Method and Setting of Confounding Measurement | The method and setting of confounding measurement are the same for all study participants. | Yes |  |
| Method used for missing data | Appropriate methods are used if imputation is used for missing confounder data. | No |  |
| Appropriate Accounting for Confounding | Important potential confounders are accounted for in the study design (e.g., matching for key variables, stratification, or initial assembly of comparable groups). /Important potential confounders are accounted for in the analysis (i.e., appropriate adjustment). | Partial |  |
| **Study Confounding Summary** | Important potential confounders are appropriately accounted for, limiting potential bias with respect to the relationship between PF and outcome . |  | Low |
|  | | | |
| **6. Statistical Analysis and Reporting** | Goal: To judge the risk of bias related to the statistical analysis and presentation of results. |  |  |
| Presentation of analytical strategy | There is sufficient presentation of data to assess the adequacy of the analysis. | Yes |  |
| Model development strategy | The strategy for model building (i.e., inclusion of variables in the statistical model) is appropriate and is based on a conceptual framework or model./ The selected statistical model is adequate for the design of the study. | Yes |  |
| Reporting of results | There is no selective reporting of results. | Unsure |  |
| **Statistical Analysis and Presentation Summary** | The statistical analysis is appropriate for the design of the study, limiting potential for presentation of invalid or spurious results. |  | Low |
|  | | | |
| Study title: | The spectrum of acute encephalitis | | |
| Study author/ data: | Singh et al., 2014 | | |
| Study identifier: | 10.1212/WNL.0000000000001190 | | |
|  | | | |
| Biases | Issues to consider for judging overall rating of "Risk of bias" | Rating of reporting | Rating of "Risk of bias" |
| **1. Study Participation** | Goal: To judge the risk of selection bias (likelihood that relationship between PF and outcome is different for participants and eligible non-participants). |  |  |
| Source of target population | The source population or population of interest is adequately described for key characteristics (LIST). | Yes |  |
| Method used to identify population | The sampling frame and recruitment are adequately described, including methods to identify the sample sufficient to limit potential bias (number and type used, e.g., referral patterns in health care) | Yes |  |
| Recruitment period | Period of recruitment is adequately described | Yes |  |
| Place of recruitment | Place of recruitment (setting and geographic location) are adequately described | Yes |  |
| Inclusion and exclusion criteria | Inclusion and exclusion criteria are adequately described (e.g., including explicit diagnostic criteria or “zero time” description). | Yes |  |
| Adequate study participation | There is adequate participation in the study by eligible individuals | Yes |  |
| Baseline characteristics | The baseline study sample (i.e., individuals entering the study) is adequately described for key characteristics (LIST). | Partial |  |
| **Summary Study participation** | The study sample represents the population of interest on key characteristics, sufficient to limit potential bias of the observed relationship between PF and outcome. |  | Low |
|  | | | |
| **2. Study Attrition** | Goal: To judge the risk of attrition bias (likelihood that relationship between PF and outcome are different for completing and non-completing participants). |  |  |
| Proportion of baseline sample available for analysis | Response rate (i.e., proportion of study sample completing the study and providing outcome data) is adequate. | Yes |  |
| Attempts to collect information on participants who dropped out | Attempts to collect information on participants who dropped out of the study are described. | No |  |
| Reasons and potential impact of subjects lost to follow-up | Reasons for loss to follow-up are provided. | No |  |
| Outcome and prognostic factor information on those lost to follow-up | Participants lost to follow-up are adequately described for key characteristics (LIST)./ There are no important differences between key characteristics (LIST) and outcomes in participants who completed the study and those who did not. | No |  |
| **Study Attrition Summary** | Loss to follow-up (from baseline sample to study population analyzed) is not associated with key characteristics (i.e., the study data adequately represent the sample) sufficient to limit potential bias to the observed relationship between PF and outcome. |  | Low |
|  | | | |
| **3. Prognostic Factor Measurement** | Goal: To judge the risk of measurement bias related to how PF was measured (differential measurement of PF related to the level of outcome). |  |  |
| Definition of the PF | A clear definition or description of 'PF' is provided (e.g., including dose, level, duration of exposure, and clear specification of the method of measurement). | Yes |  |
| Valid and Reliable Measurement of PF | Method of PF measurement is adequately valid and reliable to limit misclassification bias (e.g., may include relevant outside sources of information on measurement properties, also characteristics, such as blind measurement and limited reliance on recall)./ Continuous variables are reported or appropriate cut-points (i.e., not data-dependent) are used. | Yes |  |
| Method and Setting of PF Measurement | The method and setting of measurement of PF is the same for all study participants. | No |  |
| Proportion of data on PF available for analysis | Adequate proportion of the study sample has complete data for PF variable. | Yes |  |
| Method used for missing data | Appropriate methods of imputation are used for missing 'PF' data. | No |  |
| **PF Measurement Summary** | PF is adequately measured in study participants to sufficiently limit potential bias. |  | Low |
|  | | | |
| 4. Outcome Measurement | Goal: To judge the risk of bias related to the measurement of outcome (differential measurement of outcome related to the baseline level of PF). |  |  |
| Definition of the Outcome | A clear definition of outcome is provided, including duration of follow-up and level and extent of the outcome construct. | Yes |  |
| Valid and Reliable Measurement of Outcome | The method of outcome measurement used is adequately valid and reliable to limit misclassification bias (e.g., may include relevant outside sources of information on measurement properties, also characteristics, such as blind measurement and confirmation of outcome with valid and reliable test). | Yes |  |
| Method and Setting of Outcome Measurement | The method and setting of outcome measurement is the same for all study participants. | Yes |  |
| **Outcome Measurement Summary** | Outcome of interest is adequately measured in study participants to sufficiently limit potential bias. |  | Low |
|  | | | |
| **5. Study Confounding** | Goal: To judge the risk of bias due to confounding (i.e. the effect of PF is distorted by another factor that is related to PF and outcome). |  |  |
| Important Confounders Measured | All important confounders, including treatments (key variables in conceptual model: LIST), are measured. | Yes |  |
| Definition of the confounding factor | Clear definitions of the important confounders measured are provided (e.g., including dose, level, and duration of exposures). | Yes |  |
| Valid and Reliable Measurement of Confounders | Measurement of all important confounders is adequately valid and reliable (e.g., may include relevant outside sources of information on measurement properties, also characteristics, such as blind measurement and limited reliance on recall). | Yes |  |
| Method and Setting of Confounding Measurement | The method and setting of confounding measurement are the same for all study participants. | Yes |  |
| Method used for missing data | Appropriate methods are used if imputation is used for missing confounder data. | Yes |  |
| Appropriate Accounting for Confounding | Important potential confounders are accounted for in the study design (e.g., matching for key variables, stratification, or initial assembly of comparable groups). /Important potential confounders are accounted for in the analysis (i.e., appropriate adjustment). | Yes |  |
| **Study Confounding Summary** | Important potential confounders are appropriately accounted for, limiting potential bias with respect to the relationship between PF and outcome . |  | Low |
|  | | | |
| **6. Statistical Analysis and Reporting** | Goal: To judge the risk of bias related to the statistical analysis and presentation of results. |  |  |
| Presentation of analytical strategy | There is sufficient presentation of data to assess the adequacy of the analysis. | Yes |  |
| Model development strategy | The strategy for model building (i.e., inclusion of variables in the statistical model) is appropriate and is based on a conceptual framework or model./ The selected statistical model is adequate for the design of the study. | Yes |  |
| Reporting of results | There is no selective reporting of results. | Yes |  |
| **Statistical Analysis and Presentation Summary** | The statistical analysis is appropriate for the design of the study, limiting potential for presentation of invalid or spurious results. |  | Low |
|  | | | |
| Study title: | Characteristics, management, and outcomes of patients with infectious encephalitis requiring intensive care: A prospective multicentre observational study | | |
| Study author/ data: | Fillatre et al., 2023 | | |
| Study identifier: | 10.1016/j.jcrc.2023.154300 | | |
|  | | | |
| Biases | Issues to consider for judging overall rating of "Risk of bias" | Rating of reporting | Rating of "Risk of bias" |
| **1. Study Participation** | Goal: To judge the risk of selection bias (likelihood that relationship between PF and outcome is different for participants and eligible non-participants). |  |  |
| Source of target population | The source population or population of interest is adequately described for key characteristics (LIST). | Yes |  |
| Method used to identify population | The sampling frame and recruitment are adequately described, including methods to identify the sample sufficient to limit potential bias (number and type used, e.g., referral patterns in health care) | Partial |  |
| Recruitment period | Period of recruitment is adequately described | Yes |  |
| Place of recruitment | Place of recruitment (setting and geographic location) are adequately described | Yes |  |
| Inclusion and exclusion criteria | Inclusion and exclusion criteria are adequately described (e.g., including explicit diagnostic criteria or “zero time” description). | Yes |  |
| Adequate study participation | There is adequate participation in the study by eligible individuals | Yes |  |
| Baseline characteristics | The baseline study sample (i.e., individuals entering the study) is adequately described for key characteristics (LIST). | Yes |  |
| **Summary Study participation** | The study sample represents the population of interest on key characteristics, sufficient to limit potential bias of the observed relationship between PF and outcome. |  | Low |
|  | | | |
| **2. Study Attrition** | Goal: To judge the risk of attrition bias (likelihood that relationship between PF and outcome are different for completing and non-completing participants). |  |  |
| Proportion of baseline sample available for analysis | Response rate (i.e., proportion of study sample completing the study and providing outcome data) is adequate. | Yes |  |
| Attempts to collect information on participants who dropped out | Attempts to collect information on participants who dropped out of the study are described. | No |  |
| Reasons and potential impact of subjects lost to follow-up | Reasons for loss to follow-up are provided. | No |  |
| Outcome and prognostic factor information on those lost to follow-up | Participants lost to follow-up are adequately described for key characteristics (LIST)./ There are no important differences between key characteristics (LIST) and outcomes in participants who completed the study and those who did not. | No |  |
| **Study Attrition Summary** | Loss to follow-up (from baseline sample to study population analyzed) is not associated with key characteristics (i.e., the study data adequately represent the sample) sufficient to limit potential bias to the observed relationship between PF and outcome. |  | Low |
|  | | | |
| **3. Prognostic Factor Measurement** | Goal: To judge the risk of measurement bias related to how PF was measured (differential measurement of PF related to the level of outcome). |  |  |
| Definition of the PF | A clear definition or description of 'PF' is provided (e.g., including dose, level, duration of exposure, and clear specification of the method of measurement). | Partial |  |
| Valid and Reliable Measurement of PF | Method of PF measurement is adequately valid and reliable to limit misclassification bias (e.g., may include relevant outside sources of information on measurement properties, also characteristics, such as blind measurement and limited reliance on recall)./ Continuous variables are reported or appropriate cut-points (i.e., not data-dependent) are used. | Yes |  |
| Method and Setting of PF Measurement | The method and setting of measurement of PF is the same for all study participants. | No |  |
| Proportion of data on PF available for analysis | Adequate proportion of the study sample has complete data for PF variable. | Yes |  |
| Method used for missing data | Appropriate methods of imputation are used for missing 'PF' data. | No |  |
| **PF Measurement Summary** | PF is adequately measured in study participants to sufficiently limit potential bias. |  | Low |
|  | | | |
| 4. Outcome Measurement | Goal: To judge the risk of bias related to the measurement of outcome (differential measurement of outcome related to the baseline level of PF). |  |  |
| Definition of the Outcome | A clear definition of outcome is provided, including duration of follow-up and level and extent of the outcome construct. | Yes |  |
| Valid and Reliable Measurement of Outcome | The method of outcome measurement used is adequately valid and reliable to limit misclassification bias (e.g., may include relevant outside sources of information on measurement properties, also characteristics, such as blind measurement and confirmation of outcome with valid and reliable test). | Yes |  |
| Method and Setting of Outcome Measurement | The method and setting of outcome measurement is the same for all study participants. | Partial |  |
| **Outcome Measurement Summary** | Outcome of interest is adequately measured in study participants to sufficiently limit potential bias. |  | Low |
|  | | | |
| **5. Study Confounding** | Goal: To judge the risk of bias due to confounding (i.e. the effect of PF is distorted by another factor that is related to PF and outcome). |  |  |
| Important Confounders Measured | All important confounders, including treatments (key variables in conceptual model: LIST), are measured. | Partial |  |
| Definition of the confounding factor | Clear definitions of the important confounders measured are provided (e.g., including dose, level, and duration of exposures). | No |  |
| Valid and Reliable Measurement of Confounders | Measurement of all important confounders is adequately valid and reliable (e.g., may include relevant outside sources of information on measurement properties, also characteristics, such as blind measurement and limited reliance on recall). | Unsure |  |
| Method and Setting of Confounding Measurement | The method and setting of confounding measurement are the same for all study participants. | No |  |
| Method used for missing data | Appropriate methods are used if imputation is used for missing confounder data. | No |  |
| Appropriate Accounting for Confounding | Important potential confounders are accounted for in the study design (e.g., matching for key variables, stratification, or initial assembly of comparable groups). /Important potential confounders are accounted for in the analysis (i.e., appropriate adjustment). | Yes |  |
| **Study Confounding Summary** | Important potential confounders are appropriately accounted for, limiting potential bias with respect to the relationship between PF and outcome . |  | Moderate |
|  | | | |
| **6. Statistical Analysis and Reporting** | Goal: To judge the risk of bias related to the statistical analysis and presentation of results. |  |  |
| Presentation of analytical strategy | There is sufficient presentation of data to assess the adequacy of the analysis. | Yes |  |
| Model development strategy | The strategy for model building (i.e., inclusion of variables in the statistical model) is appropriate and is based on a conceptual framework or model./ The selected statistical model is adequate for the design of the study. | Yes |  |
| Reporting of results | There is no selective reporting of results. | Yes |  |
| **Statistical Analysis and Presentation Summary** | The statistical analysis is appropriate for the design of the study, limiting potential for presentation of invalid or spurious results. |  | Low |
|  | | | |
| Study title: | Childhood encephalitis in the Greater Mekong region (the SouthEast Asia Encephalitis Project): a multicentre prospective study | | |
| Study author/ data: | Pommier et al., 2022 | | |
| Study identifier: | 10.1016/S2214-109X%2822%2900174-7 | | |
|  | | | |
| Biases | Issues to consider for judging overall rating of "Risk of bias" | Rating of reporting | Rating of "Risk of bias" |
| **1. Study Participation** | Goal: To judge the risk of selection bias (likelihood that relationship between PF and outcome is different for participants and eligible non-participants). |  |  |
| Source of target population | The source population or population of interest is adequately described for key characteristics (LIST). | Yes |  |
| Method used to identify population | The sampling frame and recruitment are adequately described, including methods to identify the sample sufficient to limit potential bias (number and type used, e.g., referral patterns in health care) | Partial |  |
| Recruitment period | Period of recruitment is adequately described | Yes |  |
| Place of recruitment | Place of recruitment (setting and geographic location) are adequately described | Yes |  |
| Inclusion and exclusion criteria | Inclusion and exclusion criteria are adequately described (e.g., including explicit diagnostic criteria or “zero time” description). | Partial |  |
| Adequate study participation | There is adequate participation in the study by eligible individuals | Yes |  |
| Baseline characteristics | The baseline study sample (i.e., individuals entering the study) is adequately described for key characteristics (LIST). | Yes |  |
| **Summary Study participation** | The study sample represents the population of interest on key characteristics, sufficient to limit potential bias of the observed relationship between PF and outcome. |  | Low |
|  | | | |
| **2. Study Attrition** | Goal: To judge the risk of attrition bias (likelihood that relationship between PF and outcome are different for completing and non-completing participants). |  |  |
| Proportion of baseline sample available for analysis | Response rate (i.e., proportion of study sample completing the study and providing outcome data) is adequate. | Yes |  |
| Attempts to collect information on participants who dropped out | Attempts to collect information on participants who dropped out of the study are described. | No |  |
| Reasons and potential impact of subjects lost to follow-up | Reasons for loss to follow-up are provided. | No |  |
| Outcome and prognostic factor information on those lost to follow-up | Participants lost to follow-up are adequately described for key characteristics (LIST)./ There are no important differences between key characteristics (LIST) and outcomes in participants who completed the study and those who did not. | No |  |
| **Study Attrition Summary** | Loss to follow-up (from baseline sample to study population analyzed) is not associated with key characteristics (i.e., the study data adequately represent the sample) sufficient to limit potential bias to the observed relationship between PF and outcome. |  | Low |
|  | | | |
| **3. Prognostic Factor Measurement** | Goal: To judge the risk of measurement bias related to how PF was measured (differential measurement of PF related to the level of outcome). |  |  |
| Definition of the PF | A clear definition or description of 'PF' is provided (e.g., including dose, level, duration of exposure, and clear specification of the method of measurement). | Yes |  |
| Valid and Reliable Measurement of PF | Method of PF measurement is adequately valid and reliable to limit misclassification bias (e.g., may include relevant outside sources of information on measurement properties, also characteristics, such as blind measurement and limited reliance on recall)./ Continuous variables are reported or appropriate cut-points (i.e., not data-dependent) are used. | Yes |  |
| Method and Setting of PF Measurement | The method and setting of measurement of PF is the same for all study participants. | Yes |  |
| Proportion of data on PF available for analysis | Adequate proportion of the study sample has complete data for PF variable. | Yes |  |
| Method used for missing data | Appropriate methods of imputation are used for missing 'PF' data. | Yes |  |
| **PF Measurement Summary** | PF is adequately measured in study participants to sufficiently limit potential bias. |  | Low |
|  | | | |
| 4. Outcome Measurement | Goal: To judge the risk of bias related to the measurement of outcome (differential measurement of outcome related to the baseline level of PF). |  |  |
| Definition of the Outcome | A clear definition of outcome is provided, including duration of follow-up and level and extent of the outcome construct. | Yes |  |
| Valid and Reliable Measurement of Outcome | The method of outcome measurement used is adequately valid and reliable to limit misclassification bias (e.g., may include relevant outside sources of information on measurement properties, also characteristics, such as blind measurement and confirmation of outcome with valid and reliable test). | Yes |  |
| Method and Setting of Outcome Measurement | The method and setting of outcome measurement is the same for all study participants. | Yes |  |
| **Outcome Measurement Summary** | Outcome of interest is adequately measured in study participants to sufficiently limit potential bias. |  | Low |
|  | | | |
| **5. Study Confounding** | Goal: To judge the risk of bias due to confounding (i.e. the effect of PF is distorted by another factor that is related to PF and outcome). |  |  |
| Important Confounders Measured | All important confounders, including treatments (key variables in conceptual model: LIST), are measured. | Yes |  |
| Definition of the confounding factor | Clear definitions of the important confounders measured are provided (e.g., including dose, level, and duration of exposures). | Yes |  |
| Valid and Reliable Measurement of Confounders | Measurement of all important confounders is adequately valid and reliable (e.g., may include relevant outside sources of information on measurement properties, also characteristics, such as blind measurement and limited reliance on recall). | Yes |  |
| Method and Setting of Confounding Measurement | The method and setting of confounding measurement are the same for all study participants. | Yes |  |
| Method used for missing data | Appropriate methods are used if imputation is used for missing confounder data. | Yes |  |
| Appropriate Accounting for Confounding | Important potential confounders are accounted for in the study design (e.g., matching for key variables, stratification, or initial assembly of comparable groups). /Important potential confounders are accounted for in the analysis (i.e., appropriate adjustment). | Yes |  |
| **Study Confounding Summary** | Important potential confounders are appropriately accounted for, limiting potential bias with respect to the relationship between PF and outcome . |  | Low |
|  | | | |
| **6. Statistical Analysis and Reporting** | Goal: To judge the risk of bias related to the statistical analysis and presentation of results. |  |  |
| Presentation of analytical strategy | There is sufficient presentation of data to assess the adequacy of the analysis. | Yes |  |
| Model development strategy | The strategy for model building (i.e., inclusion of variables in the statistical model) is appropriate and is based on a conceptual framework or model./ The selected statistical model is adequate for the design of the study. | Yes |  |
| Reporting of results | There is no selective reporting of results. | Yes |  |
| **Statistical Analysis and Presentation Summary** | The statistical analysis is appropriate for the design of the study, limiting potential for presentation of invalid or spurious results. |  | Low |
|  | | | |
| Study title: | Clinical Epidemiology, Risk Factors, and Outcomes of Encephalitis in Older Adults | | |
| Study author/ data: | Hansen et al., 2020 | | |
| Study identifier: | 10.1093/cid/ciz635 | | |
|  | | | |
| Biases | Issues to consider for judging overall rating of "Risk of bias" | Rating of reporting | Rating of "Risk of bias" |
| **1. Study Participation** | Goal: To judge the risk of selection bias (likelihood that relationship between PF and outcome is different for participants and eligible non-participants). |  |  |
| Source of target population | The source population or population of interest is adequately described for key characteristics (LIST). | Yes |  |
| Method used to identify population | The sampling frame and recruitment are adequately described, including methods to identify the sample sufficient to limit potential bias (number and type used, e.g., referral patterns in health care) | Yes |  |
| Recruitment period | Period of recruitment is adequately described | Yes |  |
| Place of recruitment | Place of recruitment (setting and geographic location) are adequately described | Yes |  |
| Inclusion and exclusion criteria | Inclusion and exclusion criteria are adequately described (e.g., including explicit diagnostic criteria or “zero time” description). | Yes |  |
| Adequate study participation | There is adequate participation in the study by eligible individuals | Yes |  |
| Baseline characteristics | The baseline study sample (i.e., individuals entering the study) is adequately described for key characteristics (LIST). | Yes |  |
| **Summary Study participation** | The study sample represents the population of interest on key characteristics, sufficient to limit potential bias of the observed relationship between PF and outcome. |  | Low |
|  | | | |
| **2. Study Attrition** | Goal: To judge the risk of attrition bias (likelihood that relationship between PF and outcome are different for completing and non-completing participants). |  |  |
| Proportion of baseline sample available for analysis | Response rate (i.e., proportion of study sample completing the study and providing outcome data) is adequate. | Yes |  |
| Attempts to collect information on participants who dropped out | Attempts to collect information on participants who dropped out of the study are described. | No |  |
| Reasons and potential impact of subjects lost to follow-up | Reasons for loss to follow-up are provided. | No |  |
| Outcome and prognostic factor information on those lost to follow-up | Participants lost to follow-up are adequately described for key characteristics (LIST)./ There are no important differences between key characteristics (LIST) and outcomes in participants who completed the study and those who did not. | No |  |
| **Study Attrition Summary** | Loss to follow-up (from baseline sample to study population analyzed) is not associated with key characteristics (i.e., the study data adequately represent the sample) sufficient to limit potential bias to the observed relationship between PF and outcome. |  | Low |
|  | | | |
| **3. Prognostic Factor Measurement** | Goal: To judge the risk of measurement bias related to how PF was measured (differential measurement of PF related to the level of outcome). |  |  |
| Definition of the PF | A clear definition or description of 'PF' is provided (e.g., including dose, level, duration of exposure, and clear specification of the method of measurement). | Partial |  |
| Valid and Reliable Measurement of PF | Method of PF measurement is adequately valid and reliable to limit misclassification bias (e.g., may include relevant outside sources of information on measurement properties, also characteristics, such as blind measurement and limited reliance on recall)./ Continuous variables are reported or appropriate cut-points (i.e., not data-dependent) are used. | Unsure |  |
| Method and Setting of PF Measurement | The method and setting of measurement of PF is the same for all study participants. | No |  |
| Proportion of data on PF available for analysis | Adequate proportion of the study sample has complete data for PF variable. | Yes |  |
| Method used for missing data | Appropriate methods of imputation are used for missing 'PF' data. | Unsure |  |
| **PF Measurement Summary** | PF is adequately measured in study participants to sufficiently limit potential bias. |  | Moderate |
|  | | | |
| 4. Outcome Measurement | Goal: To judge the risk of bias related to the measurement of outcome (differential measurement of outcome related to the baseline level of PF). |  |  |
| Definition of the Outcome | A clear definition of outcome is provided, including duration of follow-up and level and extent of the outcome construct. | Yes |  |
| Valid and Reliable Measurement of Outcome | The method of outcome measurement used is adequately valid and reliable to limit misclassification bias (e.g., may include relevant outside sources of information on measurement properties, also characteristics, such as blind measurement and confirmation of outcome with valid and reliable test). | Yes |  |
| Method and Setting of Outcome Measurement | The method and setting of outcome measurement is the same for all study participants. | No |  |
| **Outcome Measurement Summary** | Outcome of interest is adequately measured in study participants to sufficiently limit potential bias. |  | Low |
|  | | | |
| **5. Study Confounding** | Goal: To judge the risk of bias due to confounding (i.e. the effect of PF is distorted by another factor that is related to PF and outcome). |  |  |
| Important Confounders Measured | All important confounders, including treatments (key variables in conceptual model: LIST), are measured. | Partial |  |
| Definition of the confounding factor | Clear definitions of the important confounders measured are provided (e.g., including dose, level, and duration of exposures). | Partial |  |
| Valid and Reliable Measurement of Confounders | Measurement of all important confounders is adequately valid and reliable (e.g., may include relevant outside sources of information on measurement properties, also characteristics, such as blind measurement and limited reliance on recall). | Unsure |  |
| Method and Setting of Confounding Measurement | The method and setting of confounding measurement are the same for all study participants. | No |  |
| Method used for missing data | Appropriate methods are used if imputation is used for missing confounder data. | Unsure |  |
| Appropriate Accounting for Confounding | Important potential confounders are accounted for in the study design (e.g., matching for key variables, stratification, or initial assembly of comparable groups). /Important potential confounders are accounted for in the analysis (i.e., appropriate adjustment). | Yes |  |
| **Study Confounding Summary** | Important potential confounders are appropriately accounted for, limiting potential bias with respect to the relationship between PF and outcome . |  | Moderate |
|  | | | |
| **6. Statistical Analysis and Reporting** | Goal: To judge the risk of bias related to the statistical analysis and presentation of results. |  |  |
| Presentation of analytical strategy | There is sufficient presentation of data to assess the adequacy of the analysis. | Yes |  |
| Model development strategy | The strategy for model building (i.e., inclusion of variables in the statistical model) is appropriate and is based on a conceptual framework or model./ The selected statistical model is adequate for the design of the study. | Yes |  |
| Reporting of results | There is no selective reporting of results. | Yes |  |
| **Statistical Analysis and Presentation Summary** | The statistical analysis is appropriate for the design of the study, limiting potential for presentation of invalid or spurious results. |  | Low |
|  | | | |
| Study title: | Eastern Equine Encephalitis in Children, Massachusetts and New Hampshire,USA, 1970–2010 | | |
| Study author/ data: | Silverman et al., 2013 | | |
| Study identifier: | 10.3201/eid1902.120039 | | |
|  | | | |
| Biases | Issues to consider for judging overall rating of "Risk of bias" | Rating of reporting | Rating of "Risk of bias" |
| **1. Study Participation** | Goal: To judge the risk of selection bias (likelihood that relationship between PF and outcome is different for participants and eligible non-participants). |  |  |
| Source of target population | The source population or population of interest is adequately described for key characteristics (LIST). | Partial |  |
| Method used to identify population | The sampling frame and recruitment are adequately described, including methods to identify the sample sufficient to limit potential bias (number and type used, e.g., referral patterns in health care) | No |  |
| Recruitment period | Period of recruitment is adequately described | Yes |  |
| Place of recruitment | Place of recruitment (setting and geographic location) are adequately described | Yes |  |
| Inclusion and exclusion criteria | Inclusion and exclusion criteria are adequately described (e.g., including explicit diagnostic criteria or “zero time” description). | No |  |
| Adequate study participation | There is adequate participation in the study by eligible individuals | No |  |
| Baseline characteristics | The baseline study sample (i.e., individuals entering the study) is adequately described for key characteristics (LIST). | Yes |  |
| **Summary Study participation** | The study sample represents the population of interest on key characteristics, sufficient to limit potential bias of the observed relationship between PF and outcome. |  | High |
|  | | | |
| **2. Study Attrition** | Goal: To judge the risk of attrition bias (likelihood that relationship between PF and outcome are different for completing and non-completing participants). |  |  |
| Proportion of baseline sample available for analysis | Response rate (i.e., proportion of study sample completing the study and providing outcome data) is adequate. | No |  |
| Attempts to collect information on participants who dropped out | Attempts to collect information on participants who dropped out of the study are described. | No |  |
| Reasons and potential impact of subjects lost to follow-up | Reasons for loss to follow-up are provided. | No |  |
| Outcome and prognostic factor information on those lost to follow-up | Participants lost to follow-up are adequately described for key characteristics (LIST)./ There are no important differences between key characteristics (LIST) and outcomes in participants who completed the study and those who did not. | No |  |
| **Study Attrition Summary** | Loss to follow-up (from baseline sample to study population analyzed) is not associated with key characteristics (i.e., the study data adequately represent the sample) sufficient to limit potential bias to the observed relationship between PF and outcome. |  | High |
|  | | | |
| **3. Prognostic Factor Measurement** | Goal: To judge the risk of measurement bias related to how PF was measured (differential measurement of PF related to the level of outcome). |  |  |
| Definition of the PF | A clear definition or description of 'PF' is provided (e.g., including dose, level, duration of exposure, and clear specification of the method of measurement). | Partial |  |
| Valid and Reliable Measurement of PF | Method of PF measurement is adequately valid and reliable to limit misclassification bias (e.g., may include relevant outside sources of information on measurement properties, also characteristics, such as blind measurement and limited reliance on recall)./ Continuous variables are reported or appropriate cut-points (i.e., not data-dependent) are used. | Partial |  |
| Method and Setting of PF Measurement | The method and setting of measurement of PF is the same for all study participants. | No |  |
| Proportion of data on PF available for analysis | Adequate proportion of the study sample has complete data for PF variable. | Yes |  |
| Method used for missing data | Appropriate methods of imputation are used for missing 'PF' data. | No |  |
| **PF Measurement Summary** | PF is adequately measured in study participants to sufficiently limit potential bias. |  | Moderate |
|  | | | |
| 4. Outcome Measurement | Goal: To judge the risk of bias related to the measurement of outcome (differential measurement of outcome related to the baseline level of PF). |  |  |
| Definition of the Outcome | A clear definition of outcome is provided, including duration of follow-up and level and extent of the outcome construct. | Yes |  |
| Valid and Reliable Measurement of Outcome | The method of outcome measurement used is adequately valid and reliable to limit misclassification bias (e.g., may include relevant outside sources of information on measurement properties, also characteristics, such as blind measurement and confirmation of outcome with valid and reliable test). | Yes |  |
| Method and Setting of Outcome Measurement | The method and setting of outcome measurement is the same for all study participants. | Yes |  |
| **Outcome Measurement Summary** | Outcome of interest is adequately measured in study participants to sufficiently limit potential bias. |  | Low |
|  | | | |
| **5. Study Confounding** | Goal: To judge the risk of bias due to confounding (i.e. the effect of PF is distorted by another factor that is related to PF and outcome). |  |  |
| Important Confounders Measured | All important confounders, including treatments (key variables in conceptual model: LIST), are measured. | No |  |
| Definition of the confounding factor | Clear definitions of the important confounders measured are provided (e.g., including dose, level, and duration of exposures). | No |  |
| Valid and Reliable Measurement of Confounders | Measurement of all important confounders is adequately valid and reliable (e.g., may include relevant outside sources of information on measurement properties, also characteristics, such as blind measurement and limited reliance on recall). | No |  |
| Method and Setting of Confounding Measurement | The method and setting of confounding measurement are the same for all study participants. | No |  |
| Method used for missing data | Appropriate methods are used if imputation is used for missing confounder data. | No |  |
| Appropriate Accounting for Confounding | Important potential confounders are accounted for in the study design (e.g., matching for key variables, stratification, or initial assembly of comparable groups). /Important potential confounders are accounted for in the analysis (i.e., appropriate adjustment). | No |  |
| **Study Confounding Summary** | Important potential confounders are appropriately accounted for, limiting potential bias with respect to the relationship between PF and outcome . |  | High |
|  | | | |
| **6. Statistical Analysis and Reporting** | Goal: To judge the risk of bias related to the statistical analysis and presentation of results. |  |  |
| Presentation of analytical strategy | There is sufficient presentation of data to assess the adequacy of the analysis. | No |  |
| Model development strategy | The strategy for model building (i.e., inclusion of variables in the statistical model) is appropriate and is based on a conceptual framework or model./ The selected statistical model is adequate for the design of the study. | No |  |
| Reporting of results | There is no selective reporting of results. | Unsure |  |
| **Statistical Analysis and Presentation Summary** | The statistical analysis is appropriate for the design of the study, limiting potential for presentation of invalid or spurious results. |  | High |
|  | | | |
| Study title: | Diagnosis and Therapy of Infectious Encephalitis in Children: A Ten-Years Retrospective Study | | |
| Study author/ data: | Pata et al., 2021 | | |
| Study identifier: | 10.1097/INF.0000000000003070 | | |
|  | | | |
| Biases | Issues to consider for judging overall rating of "Risk of bias" | Rating of reporting | Rating of "Risk of bias" |
| **1. Study Participation** | Goal: To judge the risk of selection bias (likelihood that relationship between PF and outcome is different for participants and eligible non-participants). |  |  |
| Source of target population | The source population or population of interest is adequately described for key characteristics (LIST). | Partial |  |
| Method used to identify population | The sampling frame and recruitment are adequately described, including methods to identify the sample sufficient to limit potential bias (number and type used, e.g., referral patterns in health care) | Partial |  |
| Recruitment period | Period of recruitment is adequately described | Yes |  |
| Place of recruitment | Place of recruitment (setting and geographic location) are adequately described | Yes |  |
| Inclusion and exclusion criteria | Inclusion and exclusion criteria are adequately described (e.g., including explicit diagnostic criteria or “zero time” description). | Partial |  |
| Adequate study participation | There is adequate participation in the study by eligible individuals | Unsure |  |
| Baseline characteristics | The baseline study sample (i.e., individuals entering the study) is adequately described for key characteristics (LIST). | Partial |  |
| **Summary Study participation** | The study sample represents the population of interest on key characteristics, sufficient to limit potential bias of the observed relationship between PF and outcome. |  | Moderate |
|  | | | |
| **2. Study Attrition** | Goal: To judge the risk of attrition bias (likelihood that relationship between PF and outcome are different for completing and non-completing participants). |  |  |
| Proportion of baseline sample available for analysis | Response rate (i.e., proportion of study sample completing the study and providing outcome data) is adequate. | No |  |
| Attempts to collect information on participants who dropped out | Attempts to collect information on participants who dropped out of the study are described. | No |  |
| Reasons and potential impact of subjects lost to follow-up | Reasons for loss to follow-up are provided. | No |  |
| Outcome and prognostic factor information on those lost to follow-up | Participants lost to follow-up are adequately described for key characteristics (LIST)./ There are no important differences between key characteristics (LIST) and outcomes in participants who completed the study and those who did not. | No |  |
| **Study Attrition Summary** | Loss to follow-up (from baseline sample to study population analyzed) is not associated with key characteristics (i.e., the study data adequately represent the sample) sufficient to limit potential bias to the observed relationship between PF and outcome. |  | High |
|  | | | |
| **3. Prognostic Factor Measurement** | Goal: To judge the risk of measurement bias related to how PF was measured (differential measurement of PF related to the level of outcome). |  |  |
| Definition of the PF | A clear definition or description of 'PF' is provided (e.g., including dose, level, duration of exposure, and clear specification of the method of measurement). | Partial |  |
| Valid and Reliable Measurement of PF | Method of PF measurement is adequately valid and reliable to limit misclassification bias (e.g., may include relevant outside sources of information on measurement properties, also characteristics, such as blind measurement and limited reliance on recall)./ Continuous variables are reported or appropriate cut-points (i.e., not data-dependent) are used. | Partial |  |
| Method and Setting of PF Measurement | The method and setting of measurement of PF is the same for all study participants. | Yes |  |
| Proportion of data on PF available for analysis | Adequate proportion of the study sample has complete data for PF variable. | No |  |
| Method used for missing data | Appropriate methods of imputation are used for missing 'PF' data. | No |  |
| **PF Measurement Summary** | PF is adequately measured in study participants to sufficiently limit potential bias. |  | High |
|  | | | |
| 4. Outcome Measurement | Goal: To judge the risk of bias related to the measurement of outcome (differential measurement of outcome related to the baseline level of PF). |  |  |
| Definition of the Outcome | A clear definition of outcome is provided, including duration of follow-up and level and extent of the outcome construct. | No |  |
| Valid and Reliable Measurement of Outcome | The method of outcome measurement used is adequately valid and reliable to limit misclassification bias (e.g., may include relevant outside sources of information on measurement properties, also characteristics, such as blind measurement and confirmation of outcome with valid and reliable test). | No |  |
| Method and Setting of Outcome Measurement | The method and setting of outcome measurement is the same for all study participants. | No |  |
| **Outcome Measurement Summary** | Outcome of interest is adequately measured in study participants to sufficiently limit potential bias. |  | High |
|  | | | |
| **5. Study Confounding** | Goal: To judge the risk of bias due to confounding (i.e. the effect of PF is distorted by another factor that is related to PF and outcome). |  |  |
| Important Confounders Measured | All important confounders, including treatments (key variables in conceptual model: LIST), are measured. | No |  |
| Definition of the confounding factor | Clear definitions of the important confounders measured are provided (e.g., including dose, level, and duration of exposures). | No |  |
| Valid and Reliable Measurement of Confounders | Measurement of all important confounders is adequately valid and reliable (e.g., may include relevant outside sources of information on measurement properties, also characteristics, such as blind measurement and limited reliance on recall). | No |  |
| Method and Setting of Confounding Measurement | The method and setting of confounding measurement are the same for all study participants. | No |  |
| Method used for missing data | Appropriate methods are used if imputation is used for missing confounder data. | No |  |
| Appropriate Accounting for Confounding | Important potential confounders are accounted for in the study design (e.g., matching for key variables, stratification, or initial assembly of comparable groups). /Important potential confounders are accounted for in the analysis (i.e., appropriate adjustment). | No |  |
| **Study Confounding Summary** | Important potential confounders are appropriately accounted for, limiting potential bias with respect to the relationship between PF and outcome . |  | High |
|  | | | |
| **6. Statistical Analysis and Reporting** | Goal: To judge the risk of bias related to the statistical analysis and presentation of results. |  |  |
| Presentation of analytical strategy | There is sufficient presentation of data to assess the adequacy of the analysis. | No |  |
| Model development strategy | The strategy for model building (i.e., inclusion of variables in the statistical model) is appropriate and is based on a conceptual framework or model./ The selected statistical model is adequate for the design of the study. | No |  |
| Reporting of results | There is no selective reporting of results. | Unsure |  |
| **Statistical Analysis and Presentation Summary** | The statistical analysis is appropriate for the design of the study, limiting potential for presentation of invalid or spurious results. |  | High |
|  | | | |
| Study title: | Herpes simplex encephalitis: Clinical presentation, neurological sequelae and new prognostic factors. Ten years of experience | | |
| Study author/ data: | Riancho et al., 2013 | | |
| Study identifier: | 10.1007/s10072-013-1475-9 | | |
|  | | | |
| Biases | Issues to consider for judging overall rating of "Risk of bias" | Rating of reporting | Rating of "Risk of bias" |
| **1. Study Participation** | Goal: To judge the risk of selection bias (likelihood that relationship between PF and outcome is different for participants and eligible non-participants). |  |  |
| Source of target population | The source population or population of interest is adequately described for key characteristics (LIST). | No |  |
| Method used to identify population | The sampling frame and recruitment are adequately described, including methods to identify the sample sufficient to limit potential bias (number and type used, e.g., referral patterns in health care) | Partial |  |
| Recruitment period | Period of recruitment is adequately described | Partial |  |
| Place of recruitment | Place of recruitment (setting and geographic location) are adequately described | Yes |  |
| Inclusion and exclusion criteria | Inclusion and exclusion criteria are adequately described (e.g., including explicit diagnostic criteria or “zero time” description). | No |  |
| Adequate study participation | There is adequate participation in the study by eligible individuals | Yes |  |
| Baseline characteristics | The baseline study sample (i.e., individuals entering the study) is adequately described for key characteristics (LIST). | No |  |
| **Summary Study participation** | The study sample represents the population of interest on key characteristics, sufficient to limit potential bias of the observed relationship between PF and outcome. |  | Moderate |
|  | | | |
| **2. Study Attrition** | Goal: To judge the risk of attrition bias (likelihood that relationship between PF and outcome are different for completing and non-completing participants). |  |  |
| Proportion of baseline sample available for analysis | Response rate (i.e., proportion of study sample completing the study and providing outcome data) is adequate. | Partial |  |
| Attempts to collect information on participants who dropped out | Attempts to collect information on participants who dropped out of the study are described. | No |  |
| Reasons and potential impact of subjects lost to follow-up | Reasons for loss to follow-up are provided. | No |  |
| Outcome and prognostic factor information on those lost to follow-up | Participants lost to follow-up are adequately described for key characteristics (LIST)./ There are no important differences between key characteristics (LIST) and outcomes in participants who completed the study and those who did not. | No |  |
| **Study Attrition Summary** | Loss to follow-up (from baseline sample to study population analyzed) is not associated with key characteristics (i.e., the study data adequately represent the sample) sufficient to limit potential bias to the observed relationship between PF and outcome. |  | High |
|  | | | |
| **3. Prognostic Factor Measurement** | Goal: To judge the risk of measurement bias related to how PF was measured (differential measurement of PF related to the level of outcome). |  |  |
| Definition of the PF | A clear definition or description of 'PF' is provided (e.g., including dose, level, duration of exposure, and clear specification of the method of measurement). | No |  |
| Valid and Reliable Measurement of PF | Method of PF measurement is adequately valid and reliable to limit misclassification bias (e.g., may include relevant outside sources of information on measurement properties, also characteristics, such as blind measurement and limited reliance on recall)./ Continuous variables are reported or appropriate cut-points (i.e., not data-dependent) are used. | Partial |  |
| Method and Setting of PF Measurement | The method and setting of measurement of PF is the same for all study participants. | Yes |  |
| Proportion of data on PF available for analysis | Adequate proportion of the study sample has complete data for PF variable. | No |  |
| Method used for missing data | Appropriate methods of imputation are used for missing 'PF' data. | No |  |
| **PF Measurement Summary** | PF is adequately measured in study participants to sufficiently limit potential bias. |  | High |
|  | | | |
| 4. Outcome Measurement | Goal: To judge the risk of bias related to the measurement of outcome (differential measurement of outcome related to the baseline level of PF). |  |  |
| Definition of the Outcome | A clear definition of outcome is provided, including duration of follow-up and level and extent of the outcome construct. | No |  |
| Valid and Reliable Measurement of Outcome | The method of outcome measurement used is adequately valid and reliable to limit misclassification bias (e.g., may include relevant outside sources of information on measurement properties, also characteristics, such as blind measurement and confirmation of outcome with valid and reliable test). | No |  |
| Method and Setting of Outcome Measurement | The method and setting of outcome measurement is the same for all study participants. | No |  |
| **Outcome Measurement Summary** | Outcome of interest is adequately measured in study participants to sufficiently limit potential bias. |  | High |
|  | | | |
| **5. Study Confounding** | Goal: To judge the risk of bias due to confounding (i.e. the effect of PF is distorted by another factor that is related to PF and outcome). |  |  |
| Important Confounders Measured | All important confounders, including treatments (key variables in conceptual model: LIST), are measured. | No |  |
| Definition of the confounding factor | Clear definitions of the important confounders measured are provided (e.g., including dose, level, and duration of exposures). | No |  |
| Valid and Reliable Measurement of Confounders | Measurement of all important confounders is adequately valid and reliable (e.g., may include relevant outside sources of information on measurement properties, also characteristics, such as blind measurement and limited reliance on recall). | No |  |
| Method and Setting of Confounding Measurement | The method and setting of confounding measurement are the same for all study participants. | No |  |
| Method used for missing data | Appropriate methods are used if imputation is used for missing confounder data. | No |  |
| Appropriate Accounting for Confounding | Important potential confounders are accounted for in the study design (e.g., matching for key variables, stratification, or initial assembly of comparable groups). /Important potential confounders are accounted for in the analysis (i.e., appropriate adjustment). | No |  |
| **Study Confounding Summary** | Important potential confounders are appropriately accounted for, limiting potential bias with respect to the relationship between PF and outcome . |  | High |
|  | | | |
| **6. Statistical Analysis and Reporting** | Goal: To judge the risk of bias related to the statistical analysis and presentation of results. |  |  |
| Presentation of analytical strategy | There is sufficient presentation of data to assess the adequacy of the analysis. | No |  |
| Model development strategy | The strategy for model building (i.e., inclusion of variables in the statistical model) is appropriate and is based on a conceptual framework or model./ The selected statistical model is adequate for the design of the study. | No |  |
| Reporting of results | There is no selective reporting of results. | Unsure |  |
| **Statistical Analysis and Presentation Summary** | The statistical analysis is appropriate for the design of the study, limiting potential for presentation of invalid or spurious results. |  | High |
|  | | | |
| Study title: | Clinical and prognostic features among children with acute encephalitis syndrome in Nepal; a retrospective study | | |
| Study author/ data: | Rayamajhi et al, 2011 | | |
| Study identifier: | 10.1186/1471-2334-11-294 | | |
|  | | | |
| Biases | Issues to consider for judging overall rating of "Risk of bias" | Rating of reporting | Rating of "Risk of bias" |
| **1. Study Participation** | Goal: To judge the risk of selection bias (likelihood that relationship between PF and outcome is different for participants and eligible non-participants). |  |  |
| Source of target population | The source population or population of interest is adequately described for key characteristics (LIST). | Yes |  |
| Method used to identify population | The sampling frame and recruitment are adequately described, including methods to identify the sample sufficient to limit potential bias (number and type used, e.g., referral patterns in health care) | Partial |  |
| Recruitment period | Period of recruitment is adequately described | Yes |  |
| Place of recruitment | Place of recruitment (setting and geographic location) are adequately described | Yes |  |
| Inclusion and exclusion criteria | Inclusion and exclusion criteria are adequately described (e.g., including explicit diagnostic criteria or “zero time” description). | Yes |  |
| Adequate study participation | There is adequate participation in the study by eligible individuals | Yes |  |
| Baseline characteristics | The baseline study sample (i.e., individuals entering the study) is adequately described for key characteristics (LIST). | Yes |  |
| **Summary Study participation** | The study sample represents the population of interest on key characteristics, sufficient to limit potential bias of the observed relationship between PF and outcome. |  | Low |
|  | | | |
| **2. Study Attrition** | Goal: To judge the risk of attrition bias (likelihood that relationship between PF and outcome are different for completing and non-completing participants). |  |  |
| Proportion of baseline sample available for analysis | Response rate (i.e., proportion of study sample completing the study and providing outcome data) is adequate. | Yes |  |
| Attempts to collect information on participants who dropped out | Attempts to collect information on participants who dropped out of the study are described. | No |  |
| Reasons and potential impact of subjects lost to follow-up | Reasons for loss to follow-up are provided. | Yes |  |
| Outcome and prognostic factor information on those lost to follow-up | Participants lost to follow-up are adequately described for key characteristics (LIST)./ There are no important differences between key characteristics (LIST) and outcomes in participants who completed the study and those who did not. | No |  |
| **Study Attrition Summary** | Loss to follow-up (from baseline sample to study population analyzed) is not associated with key characteristics (i.e., the study data adequately represent the sample) sufficient to limit potential bias to the observed relationship between PF and outcome. |  | Moderate |
|  | | | |
| **3. Prognostic Factor Measurement** | Goal: To judge the risk of measurement bias related to how PF was measured (differential measurement of PF related to the level of outcome). |  |  |
| Definition of the PF | A clear definition or description of 'PF' is provided (e.g., including dose, level, duration of exposure, and clear specification of the method of measurement). | Partial |  |
| Valid and Reliable Measurement of PF | Method of PF measurement is adequately valid and reliable to limit misclassification bias (e.g., may include relevant outside sources of information on measurement properties, also characteristics, such as blind measurement and limited reliance on recall)./ Continuous variables are reported or appropriate cut-points (i.e., not data-dependent) are used. | Yes |  |
| Method and Setting of PF Measurement | The method and setting of measurement of PF is the same for all study participants. | Yes |  |
| Proportion of data on PF available for analysis | Adequate proportion of the study sample has complete data for PF variable. | Yes |  |
| Method used for missing data | Appropriate methods of imputation are used for missing 'PF' data. | Yes |  |
| **PF Measurement Summary** | PF is adequately measured in study participants to sufficiently limit potential bias. |  | Low |
|  | | | |
| 4. Outcome Measurement | Goal: To judge the risk of bias related to the measurement of outcome (differential measurement of outcome related to the baseline level of PF). |  |  |
| Definition of the Outcome | A clear definition of outcome is provided, including duration of follow-up and level and extent of the outcome construct. | Partial |  |
| Valid and Reliable Measurement of Outcome | The method of outcome measurement used is adequately valid and reliable to limit misclassification bias (e.g., may include relevant outside sources of information on measurement properties, also characteristics, such as blind measurement and confirmation of outcome with valid and reliable test). | Yes |  |
| Method and Setting of Outcome Measurement | The method and setting of outcome measurement is the same for all study participants. | Yes |  |
| **Outcome Measurement Summary** | Outcome of interest is adequately measured in study participants to sufficiently limit potential bias. |  | Low |
|  | | | |
| **5. Study Confounding** | Goal: To judge the risk of bias due to confounding (i.e. the effect of PF is distorted by another factor that is related to PF and outcome). |  |  |
| Important Confounders Measured | All important confounders, including treatments (key variables in conceptual model: LIST), are measured. | Yes |  |
| Definition of the confounding factor | Clear definitions of the important confounders measured are provided (e.g., including dose, level, and duration of exposures). | No |  |
| Valid and Reliable Measurement of Confounders | Measurement of all important confounders is adequately valid and reliable (e.g., may include relevant outside sources of information on measurement properties, also characteristics, such as blind measurement and limited reliance on recall). | Unsure |  |
| Method and Setting of Confounding Measurement | The method and setting of confounding measurement are the same for all study participants. | Yes |  |
| Method used for missing data | Appropriate methods are used if imputation is used for missing confounder data. | Yes |  |
| Appropriate Accounting for Confounding | Important potential confounders are accounted for in the study design (e.g., matching for key variables, stratification, or initial assembly of comparable groups). /Important potential confounders are accounted for in the analysis (i.e., appropriate adjustment). | Yes |  |
| **Study Confounding Summary** | Important potential confounders are appropriately accounted for, limiting potential bias with respect to the relationship between PF and outcome . |  | Low |
|  | | | |
| **6. Statistical Analysis and Reporting** | Goal: To judge the risk of bias related to the statistical analysis and presentation of results. |  |  |
| Presentation of analytical strategy | There is sufficient presentation of data to assess the adequacy of the analysis. | Yes |  |
| Model development strategy | The strategy for model building (i.e., inclusion of variables in the statistical model) is appropriate and is based on a conceptual framework or model./ The selected statistical model is adequate for the design of the study. | Yes |  |
| Reporting of results | There is no selective reporting of results. | Yes |  |
| **Statistical Analysis and Presentation Summary** | The statistical analysis is appropriate for the design of the study, limiting potential for presentation of invalid or spurious results. |  | Low |
|  | | | |
| Study title: | Acute herpes simplex encephalitis: Clinical assessment and prognostic data | | |
| Study author/ data: | Marton et al., 1996 | | |
| Study identifier: | 10.1111/j.1600-0404.1996.tb00191.x | | |
|  | | | |
| Biases | Issues to consider for judging overall rating of "Risk of bias" | Rating of reporting | Rating of "Risk of bias" |
| **1. Study Participation** | Goal: To judge the risk of selection bias (likelihood that relationship between PF and outcome is different for participants and eligible non-participants). |  |  |
| Source of target population | The source population or population of interest is adequately described for key characteristics (LIST). | No |  |
| Method used to identify population | The sampling frame and recruitment are adequately described, including methods to identify the sample sufficient to limit potential bias (number and type used, e.g., referral patterns in health care) | Partial |  |
| Recruitment period | Period of recruitment is adequately described | Partial |  |
| Place of recruitment | Place of recruitment (setting and geographic location) are adequately described | Partial |  |
| Inclusion and exclusion criteria | Inclusion and exclusion criteria are adequately described (e.g., including explicit diagnostic criteria or “zero time” description). | No |  |
| Adequate study participation | There is adequate participation in the study by eligible individuals | Unsure |  |
| Baseline characteristics | The baseline study sample (i.e., individuals entering the study) is adequately described for key characteristics (LIST). | No |  |
| **Summary Study participation** | The study sample represents the population of interest on key characteristics, sufficient to limit potential bias of the observed relationship between PF and outcome. |  | High |
|  | | | |
| **2. Study Attrition** | Goal: To judge the risk of attrition bias (likelihood that relationship between PF and outcome are different for completing and non-completing participants). |  |  |
| Proportion of baseline sample available for analysis | Response rate (i.e., proportion of study sample completing the study and providing outcome data) is adequate. | Yes |  |
| Attempts to collect information on participants who dropped out | Attempts to collect information on participants who dropped out of the study are described. | No |  |
| Reasons and potential impact of subjects lost to follow-up | Reasons for loss to follow-up are provided. | No |  |
| Outcome and prognostic factor information on those lost to follow-up | Participants lost to follow-up are adequately described for key characteristics (LIST)./ There are no important differences between key characteristics (LIST) and outcomes in participants who completed the study and those who did not. | No |  |
| **Study Attrition Summary** | Loss to follow-up (from baseline sample to study population analyzed) is not associated with key characteristics (i.e., the study data adequately represent the sample) sufficient to limit potential bias to the observed relationship between PF and outcome. |  | Low |
|  | | | |
| **3. Prognostic Factor Measurement** | Goal: To judge the risk of measurement bias related to how PF was measured (differential measurement of PF related to the level of outcome). |  |  |
| Definition of the PF | A clear definition or description of 'PF' is provided (e.g., including dose, level, duration of exposure, and clear specification of the method of measurement). | No |  |
| Valid and Reliable Measurement of PF | Method of PF measurement is adequately valid and reliable to limit misclassification bias (e.g., may include relevant outside sources of information on measurement properties, also characteristics, such as blind measurement and limited reliance on recall)./ Continuous variables are reported or appropriate cut-points (i.e., not data-dependent) are used. | No |  |
| Method and Setting of PF Measurement | The method and setting of measurement of PF is the same for all study participants. | No |  |
| Proportion of data on PF available for analysis | Adequate proportion of the study sample has complete data for PF variable. | No |  |
| Method used for missing data | Appropriate methods of imputation are used for missing 'PF' data. | No |  |
| **PF Measurement Summary** | PF is adequately measured in study participants to sufficiently limit potential bias. |  | High |
|  | | | |
| 4. Outcome Measurement | Goal: To judge the risk of bias related to the measurement of outcome (differential measurement of outcome related to the baseline level of PF). |  |  |
| Definition of the Outcome | A clear definition of outcome is provided, including duration of follow-up and level and extent of the outcome construct. | Partial |  |
| Valid and Reliable Measurement of Outcome | The method of outcome measurement used is adequately valid and reliable to limit misclassification bias (e.g., may include relevant outside sources of information on measurement properties, also characteristics, such as blind measurement and confirmation of outcome with valid and reliable test). | No |  |
| Method and Setting of Outcome Measurement | The method and setting of outcome measurement is the same for all study participants. | No |  |
| **Outcome Measurement Summary** | Outcome of interest is adequately measured in study participants to sufficiently limit potential bias. |  | High |
|  | | | |
| **5. Study Confounding** | Goal: To judge the risk of bias due to confounding (i.e. the effect of PF is distorted by another factor that is related to PF and outcome). |  |  |
| Important Confounders Measured | All important confounders, including treatments (key variables in conceptual model: LIST), are measured. | No |  |
| Definition of the confounding factor | Clear definitions of the important confounders measured are provided (e.g., including dose, level, and duration of exposures). | No |  |
| Valid and Reliable Measurement of Confounders | Measurement of all important confounders is adequately valid and reliable (e.g., may include relevant outside sources of information on measurement properties, also characteristics, such as blind measurement and limited reliance on recall). | No |  |
| Method and Setting of Confounding Measurement | The method and setting of confounding measurement are the same for all study participants. | No |  |
| Method used for missing data | Appropriate methods are used if imputation is used for missing confounder data. | No |  |
| Appropriate Accounting for Confounding | Important potential confounders are accounted for in the study design (e.g., matching for key variables, stratification, or initial assembly of comparable groups). /Important potential confounders are accounted for in the analysis (i.e., appropriate adjustment). | No |  |
| **Study Confounding Summary** | Important potential confounders are appropriately accounted for, limiting potential bias with respect to the relationship between PF and outcome . |  | High |
|  | | | |
| **6. Statistical Analysis and Reporting** | Goal: To judge the risk of bias related to the statistical analysis and presentation of results. |  |  |
| Presentation of analytical strategy | There is sufficient presentation of data to assess the adequacy of the analysis. | Yes |  |
| Model development strategy | The strategy for model building (i.e., inclusion of variables in the statistical model) is appropriate and is based on a conceptual framework or model./ The selected statistical model is adequate for the design of the study. | No |  |
| Reporting of results | There is no selective reporting of results. | Yes |  |
| **Statistical Analysis and Presentation Summary** | The statistical analysis is appropriate for the design of the study, limiting potential for presentation of invalid or spurious results. |  | Moderate |
|  | | | |
| Study title: | Prognostic indicators of childhood acute viral encephalitis | |  |
| Study author/ data: | Bhutto et al., 1999 |  |  |
| Study identifier: | PMID: 10695286 |  |  |
|  | | | |
| Biases | Issues to consider for judging overall rating of "Risk of bias" | Rating of reporting | Rating of "Risk of bias" |
| **1. Study Participation** | Goal: To judge the risk of selection bias (likelihood that relationship between PF and outcome is different for participants and eligible non-participants). |  |  |
| Source of target population | The source population or population of interest is adequately described for key characteristics (LIST). | Partial |  |
| Method used to identify population | The sampling frame and recruitment are adequately described, including methods to identify the sample sufficient to limit potential bias (number and type used, e.g., referral patterns in health care) | No |  |
| Recruitment period | Period of recruitment is adequately described | Partial |  |
| Place of recruitment | Place of recruitment (setting and geographic location) are adequately described | Yes |  |
| Inclusion and exclusion criteria | Inclusion and exclusion criteria are adequately described (e.g., including explicit diagnostic criteria or “zero time” description). | Partial |  |
| Adequate study participation | There is adequate participation in the study by eligible individuals | Yes |  |
| Baseline characteristics | The baseline study sample (i.e., individuals entering the study) is adequately described for key characteristics (LIST). | Partial |  |
| **Summary Study participation** | The study sample represents the population of interest on key characteristics, sufficient to limit potential bias of the observed relationship between PF and outcome. |  | Moderate |
|  | | | |
| **2. Study Attrition** | Goal: To judge the risk of attrition bias (likelihood that relationship between PF and outcome are different for completing and non-completing participants). |  |  |
| Proportion of baseline sample available for analysis | Response rate (i.e., proportion of study sample completing the study and providing outcome data) is adequate. | Partial |  |
| Attempts to collect information on participants who dropped out | Attempts to collect information on participants who dropped out of the study are described. | No |  |
| Reasons and potential impact of subjects lost to follow-up | Reasons for loss to follow-up are provided. | No |  |
| Outcome and prognostic factor information on those lost to follow-up | Participants lost to follow-up are adequately described for key characteristics (LIST)./ There are no important differences between key characteristics (LIST) and outcomes in participants who completed the study and those who did not. | No |  |
| **Study Attrition Summary** | Loss to follow-up (from baseline sample to study population analyzed) is not associated with key characteristics (i.e., the study data adequately represent the sample) sufficient to limit potential bias to the observed relationship between PF and outcome. |  | Low |
|  | | | |
| **3. Prognostic Factor Measurement** | Goal: To judge the risk of measurement bias related to how PF was measured (differential measurement of PF related to the level of outcome). |  |  |
| Definition of the PF | A clear definition or description of 'PF' is provided (e.g., including dose, level, duration of exposure, and clear specification of the method of measurement). | Partial |  |
| Valid and Reliable Measurement of PF | Method of PF measurement is adequately valid and reliable to limit misclassification bias (e.g., may include relevant outside sources of information on measurement properties, also characteristics, such as blind measurement and limited reliance on recall)./ Continuous variables are reported or appropriate cut-points (i.e., not data-dependent) are used. | Partial |  |
| Method and Setting of PF Measurement | The method and setting of measurement of PF is the same for all study participants. | Yes |  |
| Proportion of data on PF available for analysis | Adequate proportion of the study sample has complete data for PF variable. | Partial |  |
| Method used for missing data | Appropriate methods of imputation are used for missing 'PF' data. | No |  |
| **PF Measurement Summary** | PF is adequately measured in study participants to sufficiently limit potential bias. |  | Moderate |
|  | | | |
| 4. Outcome Measurement | Goal: To judge the risk of bias related to the measurement of outcome (differential measurement of outcome related to the baseline level of PF). |  |  |
| Definition of the Outcome | A clear definition of outcome is provided, including duration of follow-up and level and extent of the outcome construct. | No |  |
| Valid and Reliable Measurement of Outcome | The method of outcome measurement used is adequately valid and reliable to limit misclassification bias (e.g., may include relevant outside sources of information on measurement properties, also characteristics, such as blind measurement and confirmation of outcome with valid and reliable test). | No |  |
| Method and Setting of Outcome Measurement | The method and setting of outcome measurement is the same for all study participants. | Yes |  |
| **Outcome Measurement Summary** | Outcome of interest is adequately measured in study participants to sufficiently limit potential bias. |  | Moderate |
|  | | | |
| **5. Study Confounding** | Goal: To judge the risk of bias due to confounding (i.e. the effect of PF is distorted by another factor that is related to PF and outcome). |  |  |
| Important Confounders Measured | All important confounders, including treatments (key variables in conceptual model: LIST), are measured. | No |  |
| Definition of the confounding factor | Clear definitions of the important confounders measured are provided (e.g., including dose, level, and duration of exposures). | No |  |
| Valid and Reliable Measurement of Confounders | Measurement of all important confounders is adequately valid and reliable (e.g., may include relevant outside sources of information on measurement properties, also characteristics, such as blind measurement and limited reliance on recall). | No |  |
| Method and Setting of Confounding Measurement | The method and setting of confounding measurement are the same for all study participants. | No |  |
| Method used for missing data | Appropriate methods are used if imputation is used for missing confounder data. | No |  |
| Appropriate Accounting for Confounding | Important potential confounders are accounted for in the study design (e.g., matching for key variables, stratification, or initial assembly of comparable groups). /Important potential confounders are accounted for in the analysis (i.e., appropriate adjustment). | No |  |
| **Study Confounding Summary** | Important potential confounders are appropriately accounted for, limiting potential bias with respect to the relationship between PF and outcome . |  | High |
|  | | | |
| **6. Statistical Analysis and Reporting** | Goal: To judge the risk of bias related to the statistical analysis and presentation of results. |  |  |
| Presentation of analytical strategy | There is sufficient presentation of data to assess the adequacy of the analysis. | Yes |  |
| Model development strategy | The strategy for model building (i.e., inclusion of variables in the statistical model) is appropriate and is based on a conceptual framework or model./ The selected statistical model is adequate for the design of the study. | No |  |
| Reporting of results | There is no selective reporting of results. | Unsure |  |
| **Statistical Analysis and Presentation Summary** | The statistical analysis is appropriate for the design of the study, limiting potential for presentation of invalid or spurious results. |  | Moderate |
|  | | | |
| Study title: | Clinical analysis of 17 cases of Japanese encephalitis experienced in the last ten years | | |
| Study author/ data: | Takada et al., 1989 | | |
| Study identifier: | 10.11150/kansenshogakuzasshi1970.63.494 | | |
|  | | | |
| Biases | Issues to consider for judging overall rating of "Risk of bias" | Rating of reporting | Rating of "Risk of bias" |
| **1. Study Participation** | Goal: To judge the risk of selection bias (likelihood that relationship between PF and outcome is different for participants and eligible non-participants). |  |  |
| Source of target population | The source population or population of interest is adequately described for key characteristics (LIST). | No |  |
| Method used to identify population | The sampling frame and recruitment are adequately described, including methods to identify the sample sufficient to limit potential bias (number and type used, e.g., referral patterns in health care) | Partial |  |
| Recruitment period | Period of recruitment is adequately described | Yes |  |
| Place of recruitment | Place of recruitment (setting and geographic location) are adequately described | Yes |  |
| Inclusion and exclusion criteria | Inclusion and exclusion criteria are adequately described (e.g., including explicit diagnostic criteria or “zero time” description). | No |  |
| Adequate study participation | There is adequate participation in the study by eligible individuals | No |  |
| Baseline characteristics | The baseline study sample (i.e., individuals entering the study) is adequately described for key characteristics (LIST). | No |  |
| **Summary Study participation** | The study sample represents the population of interest on key characteristics, sufficient to limit potential bias of the observed relationship between PF and outcome. |  | High |
|  | | | |
| **2. Study Attrition** | Goal: To judge the risk of attrition bias (likelihood that relationship between PF and outcome are different for completing and non-completing participants). |  |  |
| Proportion of baseline sample available for analysis | Response rate (i.e., proportion of study sample completing the study and providing outcome data) is adequate. | Yes |  |
| Attempts to collect information on participants who dropped out | Attempts to collect information on participants who dropped out of the study are described. | No |  |
| Reasons and potential impact of subjects lost to follow-up | Reasons for loss to follow-up are provided. | No |  |
| Outcome and prognostic factor information on those lost to follow-up | Participants lost to follow-up are adequately described for key characteristics (LIST)./ There are no important differences between key characteristics (LIST) and outcomes in participants who completed the study and those who did not. | No |  |
| **Study Attrition Summary** | Loss to follow-up (from baseline sample to study population analyzed) is not associated with key characteristics (i.e., the study data adequately represent the sample) sufficient to limit potential bias to the observed relationship between PF and outcome. |  | Low |
|  | | | |
| **3. Prognostic Factor Measurement** | Goal: To judge the risk of measurement bias related to how PF was measured (differential measurement of PF related to the level of outcome). |  |  |
| Definition of the PF | A clear definition or description of 'PF' is provided (e.g., including dose, level, duration of exposure, and clear specification of the method of measurement). | Partial |  |
| Valid and Reliable Measurement of PF | Method of PF measurement is adequately valid and reliable to limit misclassification bias (e.g., may include relevant outside sources of information on measurement properties, also characteristics, such as blind measurement and limited reliance on recall)./ Continuous variables are reported or appropriate cut-points (i.e., not data-dependent) are used. | No |  |
| Method and Setting of PF Measurement | The method and setting of measurement of PF is the same for all study participants. | Yes |  |
| Proportion of data on PF available for analysis | Adequate proportion of the study sample has complete data for PF variable. | Yes |  |
| Method used for missing data | Appropriate methods of imputation are used for missing 'PF' data. | No |  |
| **PF Measurement Summary** | PF is adequately measured in study participants to sufficiently limit potential bias. |  | Moderate |
|  | | | |
| 4. Outcome Measurement | Goal: To judge the risk of bias related to the measurement of outcome (differential measurement of outcome related to the baseline level of PF). |  |  |
| Definition of the Outcome | A clear definition of outcome is provided, including duration of follow-up and level and extent of the outcome construct. | No |  |
| Valid and Reliable Measurement of Outcome | The method of outcome measurement used is adequately valid and reliable to limit misclassification bias (e.g., may include relevant outside sources of information on measurement properties, also characteristics, such as blind measurement and confirmation of outcome with valid and reliable test). | No |  |
| Method and Setting of Outcome Measurement | The method and setting of outcome measurement is the same for all study participants. | No |  |
| **Outcome Measurement Summary** | Outcome of interest is adequately measured in study participants to sufficiently limit potential bias. |  | High |
|  | | | |
| **5. Study Confounding** | Goal: To judge the risk of bias due to confounding (i.e. the effect of PF is distorted by another factor that is related to PF and outcome). |  |  |
| Important Confounders Measured | All important confounders, including treatments (key variables in conceptual model: LIST), are measured. | Partial |  |
| Definition of the confounding factor | Clear definitions of the important confounders measured are provided (e.g., including dose, level, and duration of exposures). | No |  |
| Valid and Reliable Measurement of Confounders | Measurement of all important confounders is adequately valid and reliable (e.g., may include relevant outside sources of information on measurement properties, also characteristics, such as blind measurement and limited reliance on recall). | No |  |
| Method and Setting of Confounding Measurement | The method and setting of confounding measurement are the same for all study participants. | No |  |
| Method used for missing data | Appropriate methods are used if imputation is used for missing confounder data. | No |  |
| Appropriate Accounting for Confounding | Important potential confounders are accounted for in the study design (e.g., matching for key variables, stratification, or initial assembly of comparable groups). /Important potential confounders are accounted for in the analysis (i.e., appropriate adjustment). | No |  |
| **Study Confounding Summary** | Important potential confounders are appropriately accounted for, limiting potential bias with respect to the relationship between PF and outcome . |  | High |
|  | | | |
| **6. Statistical Analysis and Reporting** | Goal: To judge the risk of bias related to the statistical analysis and presentation of results. |  |  |
| Presentation of analytical strategy | There is sufficient presentation of data to assess the adequacy of the analysis. | No |  |
| Model development strategy | The strategy for model building (i.e., inclusion of variables in the statistical model) is appropriate and is based on a conceptual framework or model./ The selected statistical model is adequate for the design of the study. | No |  |
| Reporting of results | There is no selective reporting of results. | No |  |
| **Statistical Analysis and Presentation Summary** | The statistical analysis is appropriate for the design of the study, limiting potential for presentation of invalid or spurious results. |  | High |
|  | | | |
| Study title: | Seizures and raised intracranial pressure in Vietnamese patients with Japanese encephalitis | | |
| Study author/ data: | Solomon et al., 2002 | | |
| Study identifier: | 10.1093/brain/awf116 | | |
|  | | | |
| Biases | Issues to consider for judging overall rating of "Risk of bias" | Rating of reporting | Rating of "Risk of bias" |
| **1. Study Participation** | Goal: To judge the risk of selection bias (likelihood that relationship between PF and outcome is different for participants and eligible non-participants). |  |  |
| Source of target population | The source population or population of interest is adequately described for key characteristics (LIST). | Yes |  |
| Method used to identify population | The sampling frame and recruitment are adequately described, including methods to identify the sample sufficient to limit potential bias (number and type used, e.g., referral patterns in health care) | Partial |  |
| Recruitment period | Period of recruitment is adequately described | Yes |  |
| Place of recruitment | Place of recruitment (setting and geographic location) are adequately described | Yes |  |
| Inclusion and exclusion criteria | Inclusion and exclusion criteria are adequately described (e.g., including explicit diagnostic criteria or “zero time” description). | Yes |  |
| Adequate study participation | There is adequate participation in the study by eligible individuals | Partial |  |
| Baseline characteristics | The baseline study sample (i.e., individuals entering the study) is adequately described for key characteristics (LIST). | Yes |  |
| **Summary Study participation** | The study sample represents the population of interest on key characteristics, sufficient to limit potential bias of the observed relationship between PF and outcome. |  | Low |
|  | | | |
| **2. Study Attrition** | Goal: To judge the risk of attrition bias (likelihood that relationship between PF and outcome are different for completing and non-completing participants). |  |  |
| Proportion of baseline sample available for analysis | Response rate (i.e., proportion of study sample completing the study and providing outcome data) is adequate. | Yes |  |
| Attempts to collect information on participants who dropped out | Attempts to collect information on participants who dropped out of the study are described. | No |  |
| Reasons and potential impact of subjects lost to follow-up | Reasons for loss to follow-up are provided. | No |  |
| Outcome and prognostic factor information on those lost to follow-up | Participants lost to follow-up are adequately described for key characteristics (LIST)./ There are no important differences between key characteristics (LIST) and outcomes in participants who completed the study and those who did not. | No |  |
| **Study Attrition Summary** | Loss to follow-up (from baseline sample to study population analyzed) is not associated with key characteristics (i.e., the study data adequately represent the sample) sufficient to limit potential bias to the observed relationship between PF and outcome. |  | Low |
|  | | | |
| **3. Prognostic Factor Measurement** | Goal: To judge the risk of measurement bias related to how PF was measured (differential measurement of PF related to the level of outcome). |  |  |
| Definition of the PF | A clear definition or description of 'PF' is provided (e.g., including dose, level, duration of exposure, and clear specification of the method of measurement). | Yes |  |
| Valid and Reliable Measurement of PF | Method of PF measurement is adequately valid and reliable to limit misclassification bias (e.g., may include relevant outside sources of information on measurement properties, also characteristics, such as blind measurement and limited reliance on recall)./ Continuous variables are reported or appropriate cut-points (i.e., not data-dependent) are used. | Yes |  |
| Method and Setting of PF Measurement | The method and setting of measurement of PF is the same for all study participants. | Yes |  |
| Proportion of data on PF available for analysis | Adequate proportion of the study sample has complete data for PF variable. | Yes |  |
| Method used for missing data | Appropriate methods of imputation are used for missing 'PF' data. | Yes |  |
| **PF Measurement Summary** | PF is adequately measured in study participants to sufficiently limit potential bias. |  | Low |
|  | | | |
| 4. Outcome Measurement | Goal: To judge the risk of bias related to the measurement of outcome (differential measurement of outcome related to the baseline level of PF). |  |  |
| Definition of the Outcome | A clear definition of outcome is provided, including duration of follow-up and level and extent of the outcome construct. | Yes |  |
| Valid and Reliable Measurement of Outcome | The method of outcome measurement used is adequately valid and reliable to limit misclassification bias (e.g., may include relevant outside sources of information on measurement properties, also characteristics, such as blind measurement and confirmation of outcome with valid and reliable test). | Partial |  |
| Method and Setting of Outcome Measurement | The method and setting of outcome measurement is the same for all study participants. | Yes |  |
| **Outcome Measurement Summary** | Outcome of interest is adequately measured in study participants to sufficiently limit potential bias. |  | Low |
|  | | | |
| **5. Study Confounding** | Goal: To judge the risk of bias due to confounding (i.e. the effect of PF is distorted by another factor that is related to PF and outcome). |  |  |
| Important Confounders Measured | All important confounders, including treatments (key variables in conceptual model: LIST), are measured. | Partial |  |
| Definition of the confounding factor | Clear definitions of the important confounders measured are provided (e.g., including dose, level, and duration of exposures). | No |  |
| Valid and Reliable Measurement of Confounders | Measurement of all important confounders is adequately valid and reliable (e.g., may include relevant outside sources of information on measurement properties, also characteristics, such as blind measurement and limited reliance on recall). | No |  |
| Method and Setting of Confounding Measurement | The method and setting of confounding measurement are the same for all study participants. | Yes |  |
| Method used for missing data | Appropriate methods are used if imputation is used for missing confounder data. | No |  |
| Appropriate Accounting for Confounding | Important potential confounders are accounted for in the study design (e.g., matching for key variables, stratification, or initial assembly of comparable groups). /Important potential confounders are accounted for in the analysis (i.e., appropriate adjustment). | Yes |  |
| **Study Confounding Summary** | Important potential confounders are appropriately accounted for, limiting potential bias with respect to the relationship between PF and outcome . |  | Low |
|  | | | |
| **6. Statistical Analysis and Reporting** | Goal: To judge the risk of bias related to the statistical analysis and presentation of results. |  |  |
| Presentation of analytical strategy | There is sufficient presentation of data to assess the adequacy of the analysis. | Yes |  |
| Model development strategy | The strategy for model building (i.e., inclusion of variables in the statistical model) is appropriate and is based on a conceptual framework or model./ The selected statistical model is adequate for the design of the study. | Yes |  |
| Reporting of results | There is no selective reporting of results. | Yes |  |
| **Statistical Analysis and Presentation Summary** | The statistical analysis is appropriate for the design of the study, limiting potential for presentation of invalid or spurious results. |  | Low |

Poor outcomes at >6 months: QUIPS analysis

| Study title: | Outcome of and Prognostic Factors for Herpes Simplex Encephalitis in Adult Patients: Results of a Multicenter Study | | |
| --- | --- | --- | --- |
| Study author/ data: | Raschilas et al., 2002 | | |
| Study identifier: | 10.1086/341405 | | |
|  | | | |
| Biases | Issues to consider for judging overall rating of "Risk of bias" | Rating of reporting | Rating of "Risk of bias" |
| **1. Study Participation** | Goal: To judge the risk of selection bias (likelihood that relationship between PF and outcome is different for participants and eligible non-participants). |  |  |
| Source of target population | The source population or population of interest is adequately described for key characteristics (LIST). | Partial |  |
| Method used to identify population | The sampling frame and recruitment are adequately described, including methods to identify the sample sufficient to limit potential bias (number and type used, e.g., referral patterns in health care) | Partial |  |
| Recruitment period | Period of recruitment is adequately described | Yes |  |
| Place of recruitment | Place of recruitment (setting and geographic location) are adequately described | Yes |  |
| Inclusion and exclusion criteria | Inclusion and exclusion criteria are adequately described (e.g., including explicit diagnostic criteria or “zero time” description). | Yes |  |
| Adequate study participation | There is adequate participation in the study by eligible individuals | Yes |  |
| Baseline characteristics | The baseline study sample (i.e., individuals entering the study) is adequately described for key characteristics (LIST). | Partial |  |
| **Summary Study participation** | The study sample represents the population of interest on key characteristics, sufficient to limit potential bias of the observed relationship between PF and outcome. |  | Low |
|  | | | |
| **2. Study Attrition** | Goal: To judge the risk of attrition bias (likelihood that relationship between PF and outcome are different for completing and non-completing participants). |  |  |
| Proportion of baseline sample available for analysis | Response rate (i.e., proportion of study sample completing the study and providing outcome data) is adequate. | Yes |  |
| Attempts to collect information on participants who dropped out | Attempts to collect information on participants who dropped out of the study are described. | No |  |
| Reasons and potential impact of subjects lost to follow-up | Reasons for loss to follow-up are provided. | No |  |
| Outcome and prognostic factor information on those lost to follow-up | Participants lost to follow-up are adequately described for key characteristics (LIST)./ There are no important differences between key characteristics (LIST) and outcomes in participants who completed the study and those who did not. | No |  |
| **Study Attrition Summary** | Loss to follow-up (from baseline sample to study population analyzed) is not associated with key characteristics (i.e., the study data adequately represent the sample) sufficient to limit potential bias to the observed relationship between PF and outcome. |  | High |
|  | | | |
| **3. Prognostic Factor Measurement** | Goal: To judge the risk of measurement bias related to how PF was measured (differential measurement of PF related to the level of outcome). |  |  |
| Definition of the PF | A clear definition or description of 'PF' is provided (e.g., including dose, level, duration of exposure, and clear specification of the method of measurement). | Partial |  |
| Valid and Reliable Measurement of PF | Method of PF measurement is adequately valid and reliable to limit misclassification bias (e.g., may include relevant outside sources of information on measurement properties, also characteristics, such as blind measurement and limited reliance on recall)./ Continuous variables are reported or appropriate cut-points (i.e., not data-dependent) are used. | Yes |  |
| Method and Setting of PF Measurement | The method and setting of measurement of PF is the same for all study participants. | Yes |  |
| Proportion of data on PF available for analysis | Adequate proportion of the study sample has complete data for PF variable. | Yes |  |
| Method used for missing data | Appropriate methods of imputation are used for missing 'PF' data. | No |  |
| **PF Measurement Summary** | PF is adequately measured in study participants to sufficiently limit potential bias. |  | Low |
|  | | | |
| 4. Outcome Measurement | Goal: To judge the risk of bias related to the measurement of outcome (differential measurement of outcome related to the baseline level of PF). |  |  |
| Definition of the Outcome | A clear definition of outcome is provided, including duration of follow-up and level and extent of the outcome construct. | Yes |  |
| Valid and Reliable Measurement of Outcome | The method of outcome measurement used is adequately valid and reliable to limit misclassification bias (e.g., may include relevant outside sources of information on measurement properties, also characteristics, such as blind measurement and confirmation of outcome with valid and reliable test). | Yes |  |
| Method and Setting of Outcome Measurement | The method and setting of outcome measurement is the same for all study participants. | Yes |  |
| **Outcome Measurement Summary** | Outcome of interest is adequately measured in study participants to sufficiently limit potential bias. |  | Low |
|  | | | |
| **5. Study Confounding** | Goal: To judge the risk of bias due to confounding (i.e. the effect of PF is distorted by another factor that is related to PF and outcome). |  |  |
| Important Confounders Measured | All important confounders, including treatments (key variables in conceptual model: LIST), are measured. | Partial |  |
| Definition of the confounding factor | Clear definitions of the important confounders measured are provided (e.g., including dose, level, and duration of exposures). | Partial |  |
| Valid and Reliable Measurement of Confounders | Measurement of all important confounders is adequately valid and reliable (e.g., may include relevant outside sources of information on measurement properties, also characteristics, such as blind measurement and limited reliance on recall). | Yes |  |
| Method and Setting of Confounding Measurement | The method and setting of confounding measurement are the same for all study participants. | Yes |  |
| Method used for missing data | Appropriate methods are used if imputation is used for missing confounder data. | Yes |  |
| Appropriate Accounting for Confounding | Important potential confounders are accounted for in the study design (e.g., matching for key variables, stratification, or initial assembly of comparable groups). /Important potential confounders are accounted for in the analysis (i.e., appropriate adjustment). | Yes |  |
| **Study Confounding Summary** | Important potential confounders are appropriately accounted for, limiting potential bias with respect to the relationship between PF and outcome . |  | Low |
|  | | | |
| **6. Statistical Analysis and Reporting** | Goal: To judge the risk of bias related to the statistical analysis and presentation of results. |  |  |
| Presentation of analytical strategy | There is sufficient presentation of data to assess the adequacy of the analysis. | Yes |  |
| Model development strategy | The strategy for model building (i.e., inclusion of variables in the statistical model) is appropriate and is based on a conceptual framework or model./ The selected statistical model is adequate for the design of the study. | Yes |  |
| Reporting of results | There is no selective reporting of results. | Yes |  |
| **Statistical Analysis and Presentation Summary** | The statistical analysis is appropriate for the design of the study, limiting potential for presentation of invalid or spurious results. |  | Low |
|  | | | |
| Study title: | Prognostic Value of Initial Standard EEG and MRI in Patients with Herpes Simplex Encephalitis | | |
| Study author/ data: | Kim et al., 2016 | | |
| Study identifier: | 10.3988/jcn.2016.12.2.224 | | |
|  | | | |
| Biases | Issues to consider for judging overall rating of "Risk of bias" | Rating of reporting | Rating of "Risk of bias" |
| **1. Study Participation** | Goal: To judge the risk of selection bias (likelihood that relationship between PF and outcome is different for participants and eligible non-participants). |  |  |
| Source of target population | The source population or population of interest is adequately described for key characteristics (LIST). | Partial |  |
| Method used to identify population | The sampling frame and recruitment are adequately described, including methods to identify the sample sufficient to limit potential bias (number and type used, e.g., referral patterns in health care) | Partial |  |
| Recruitment period | Period of recruitment is adequately described | Yes |  |
| Place of recruitment | Place of recruitment (setting and geographic location) are adequately described | Yes |  |
| Inclusion and exclusion criteria | Inclusion and exclusion criteria are adequately described (e.g., including explicit diagnostic criteria or “zero time” description). | Yes |  |
| Adequate study participation | There is adequate participation in the study by eligible individuals | Yes |  |
| Baseline characteristics | The baseline study sample (i.e., individuals entering the study) is adequately described for key characteristics (LIST). | Partial |  |
| **Summary Study participation** | The study sample represents the population of interest on key characteristics, sufficient to limit potential bias of the observed relationship between PF and outcome. |  | Low |
|  | | | |
| **2. Study Attrition** | Goal: To judge the risk of attrition bias (likelihood that relationship between PF and outcome are different for completing and non-completing participants). |  |  |
| Proportion of baseline sample available for analysis | Response rate (i.e., proportion of study sample completing the study and providing outcome data) is adequate. | Yes |  |
| Attempts to collect information on participants who dropped out | Attempts to collect information on participants who dropped out of the study are described. | No |  |
| Reasons and potential impact of subjects lost to follow-up | Reasons for loss to follow-up are provided. | No |  |
| Outcome and prognostic factor information on those lost to follow-up | Participants lost to follow-up are adequately described for key characteristics (LIST)./ There are no important differences between key characteristics (LIST) and outcomes in participants who completed the study and those who did not. | No |  |
| **Study Attrition Summary** | Loss to follow-up (from baseline sample to study population analyzed) is not associated with key characteristics (i.e., the study data adequately represent the sample) sufficient to limit potential bias to the observed relationship between PF and outcome. |  | Low |
|  | | | |
| **3. Prognostic Factor Measurement** | Goal: To judge the risk of measurement bias related to how PF was measured (differential measurement of PF related to the level of outcome). |  |  |
| Definition of the PF | A clear definition or description of 'PF' is provided (e.g., including dose, level, duration of exposure, and clear specification of the method of measurement). | Yes |  |
| Valid and Reliable Measurement of PF | Method of PF measurement is adequately valid and reliable to limit misclassification bias (e.g., may include relevant outside sources of information on measurement properties, also characteristics, such as blind measurement and limited reliance on recall)./ Continuous variables are reported or appropriate cut-points (i.e., not data-dependent) are used. | Yes |  |
| Method and Setting of PF Measurement | The method and setting of measurement of PF is the same for all study participants. | Yes |  |
| Proportion of data on PF available for analysis | Adequate proportion of the study sample has complete data for PF variable. | Yes |  |
| Method used for missing data | Appropriate methods of imputation are used for missing 'PF' data. | No |  |
| **PF Measurement Summary** | PF is adequately measured in study participants to sufficiently limit potential bias. |  | Low |
|  | | | |
| 4. Outcome Measurement | Goal: To judge the risk of bias related to the measurement of outcome (differential measurement of outcome related to the baseline level of PF). |  |  |
| Definition of the Outcome | A clear definition of outcome is provided, including duration of follow-up and level and extent of the outcome construct. | Yes |  |
| Valid and Reliable Measurement of Outcome | The method of outcome measurement used is adequately valid and reliable to limit misclassification bias (e.g., may include relevant outside sources of information on measurement properties, also characteristics, such as blind measurement and confirmation of outcome with valid and reliable test). | Unsure |  |
| Method and Setting of Outcome Measurement | The method and setting of outcome measurement is the same for all study participants. | Yes |  |
| **Outcome Measurement Summary** | Outcome of interest is adequately measured in study participants to sufficiently limit potential bias. |  | Low |
|  | | | |
| **5. Study Confounding** | Goal: To judge the risk of bias due to confounding (i.e. the effect of PF is distorted by another factor that is related to PF and outcome). |  |  |
| Important Confounders Measured | All important confounders, including treatments (key variables in conceptual model: LIST), are measured. | Partial |  |
| Definition of the confounding factor | Clear definitions of the important confounders measured are provided (e.g., including dose, level, and duration of exposures). | Partial |  |
| Valid and Reliable Measurement of Confounders | Measurement of all important confounders is adequately valid and reliable (e.g., may include relevant outside sources of information on measurement properties, also characteristics, such as blind measurement and limited reliance on recall). | Yes |  |
| Method and Setting of Confounding Measurement | The method and setting of confounding measurement are the same for all study participants. | Yes |  |
| Method used for missing data | Appropriate methods are used if imputation is used for missing confounder data. | Yes |  |
| Appropriate Accounting for Confounding | Important potential confounders are accounted for in the study design (e.g., matching for key variables, stratification, or initial assembly of comparable groups). /Important potential confounders are accounted for in the analysis (i.e., appropriate adjustment). | Yes |  |
| **Study Confounding Summary** | Important potential confounders are appropriately accounted for, limiting potential bias with respect to the relationship between PF and outcome . |  | Low |
|  | | | |
| **6. Statistical Analysis and Reporting** | Goal: To judge the risk of bias related to the statistical analysis and presentation of results. |  |  |
| Presentation of analytical strategy | There is sufficient presentation of data to assess the adequacy of the analysis. | Yes |  |
| Model development strategy | The strategy for model building (i.e., inclusion of variables in the statistical model) is appropriate and is based on a conceptual framework or model./ The selected statistical model is adequate for the design of the study. | Yes |  |
| Reporting of results | There is no selective reporting of results. | Yes |  |
| **Statistical Analysis and Presentation Summary** | The statistical analysis is appropriate for the design of the study, limiting potential for presentation of invalid or spurious results. |  | Low |
|  | | | |
| Study title: | Determining the Clinical Characteristics, Treatment Strategies, and Prognostic Factors for Mycoplasma pneumoniae Encephalitis in Children: A Multicenter Study in China | | |
| Study author/ data: | Fan et al., 2023 | | |
| Study identifier: | 10.3988/jcn.2022.0328 | | |
|  | | | |
| Biases | Issues to consider for judging overall rating of "Risk of bias" | Rating of reporting | Rating of "Risk of bias" |
| **1. Study Participation** | Goal: To judge the risk of selection bias (likelihood that relationship between PF and outcome is different for participants and eligible non-participants). |  |  |
| Source of target population | The source population or population of interest is adequately described for key characteristics (LIST). | Yes |  |
| Method used to identify population | The sampling frame and recruitment are adequately described, including methods to identify the sample sufficient to limit potential bias (number and type used, e.g., referral patterns in health care) | Partial |  |
| Recruitment period | Period of recruitment is adequately described | Yes |  |
| Place of recruitment | Place of recruitment (setting and geographic location) are adequately described | Yes |  |
| Inclusion and exclusion criteria | Inclusion and exclusion criteria are adequately described (e.g., including explicit diagnostic criteria or “zero time” description). | Partial |  |
| Adequate study participation | There is adequate participation in the study by eligible individuals | Yes |  |
| Baseline characteristics | The baseline study sample (i.e., individuals entering the study) is adequately described for key characteristics (LIST). | Yes |  |
| **Summary Study participation** | The study sample represents the population of interest on key characteristics, sufficient to limit potential bias of the observed relationship between PF and outcome. |  | Low |
|  | | | |
| **2. Study Attrition** | Goal: To judge the risk of attrition bias (likelihood that relationship between PF and outcome are different for completing and non-completing participants). |  |  |
| Proportion of baseline sample available for analysis | Response rate (i.e., proportion of study sample completing the study and providing outcome data) is adequate. | Yes |  |
| Attempts to collect information on participants who dropped out | Attempts to collect information on participants who dropped out of the study are described. | No |  |
| Reasons and potential impact of subjects lost to follow-up | Reasons for loss to follow-up are provided. | No |  |
| Outcome and prognostic factor information on those lost to follow-up | Participants lost to follow-up are adequately described for key characteristics (LIST)./ There are no important differences between key characteristics (LIST) and outcomes in participants who completed the study and those who did not. | No |  |
| **Study Attrition Summary** | Loss to follow-up (from baseline sample to study population analyzed) is not associated with key characteristics (i.e., the study data adequately represent the sample) sufficient to limit potential bias to the observed relationship between PF and outcome. |  | Low |
|  | | | |
| **3. Prognostic Factor Measurement** | Goal: To judge the risk of measurement bias related to how PF was measured (differential measurement of PF related to the level of outcome). |  |  |
| Definition of the PF | A clear definition or description of 'PF' is provided (e.g., including dose, level, duration of exposure, and clear specification of the method of measurement). | Partial |  |
| Valid and Reliable Measurement of PF | Method of PF measurement is adequately valid and reliable to limit misclassification bias (e.g., may include relevant outside sources of information on measurement properties, also characteristics, such as blind measurement and limited reliance on recall)./ Continuous variables are reported or appropriate cut-points (i.e., not data-dependent) are used. | Unsure |  |
| Method and Setting of PF Measurement | The method and setting of measurement of PF is the same for all study participants. | No |  |
| Proportion of data on PF available for analysis | Adequate proportion of the study sample has complete data for PF variable. | Yes |  |
| Method used for missing data | Appropriate methods of imputation are used for missing 'PF' data. | No |  |
| **PF Measurement Summary** | PF is adequately measured in study participants to sufficiently limit potential bias. |  | Moderate |
|  | | | |
| 4. Outcome Measurement | Goal: To judge the risk of bias related to the measurement of outcome (differential measurement of outcome related to the baseline level of PF). |  |  |
| Definition of the Outcome | A clear definition of outcome is provided, including duration of follow-up and level and extent of the outcome construct. | Yes |  |
| Valid and Reliable Measurement of Outcome | The method of outcome measurement used is adequately valid and reliable to limit misclassification bias (e.g., may include relevant outside sources of information on measurement properties, also characteristics, such as blind measurement and confirmation of outcome with valid and reliable test). | Yes |  |
| Method and Setting of Outcome Measurement | The method and setting of outcome measurement is the same for all study participants. | Unsure |  |
| **Outcome Measurement Summary** | Outcome of interest is adequately measured in study participants to sufficiently limit potential bias. |  | Low |
|  | | | |
| **5. Study Confounding** | Goal: To judge the risk of bias due to confounding (i.e. the effect of PF is distorted by another factor that is related to PF and outcome). |  |  |
| Important Confounders Measured | All important confounders, including treatments (key variables in conceptual model: LIST), are measured. | Partial |  |
| Definition of the confounding factor | Clear definitions of the important confounders measured are provided (e.g., including dose, level, and duration of exposures). | Partial |  |
| Valid and Reliable Measurement of Confounders | Measurement of all important confounders is adequately valid and reliable (e.g., may include relevant outside sources of information on measurement properties, also characteristics, such as blind measurement and limited reliance on recall). | Yes |  |
| Method and Setting of Confounding Measurement | The method and setting of confounding measurement are the same for all study participants. | Yes |  |
| Method used for missing data | Appropriate methods are used if imputation is used for missing confounder data. | No |  |
| Appropriate Accounting for Confounding | Important potential confounders are accounted for in the study design (e.g., matching for key variables, stratification, or initial assembly of comparable groups). /Important potential confounders are accounted for in the analysis (i.e., appropriate adjustment). | Yes |  |
| **Study Confounding Summary** | Important potential confounders are appropriately accounted for, limiting potential bias with respect to the relationship between PF and outcome . |  | Low |
|  | | | |
| **6. Statistical Analysis and Reporting** | Goal: To judge the risk of bias related to the statistical analysis and presentation of results. |  |  |
| Presentation of analytical strategy | There is sufficient presentation of data to assess the adequacy of the analysis. | Yes |  |
| Model development strategy | The strategy for model building (i.e., inclusion of variables in the statistical model) is appropriate and is based on a conceptual framework or model./ The selected statistical model is adequate for the design of the study. | Yes |  |
| Reporting of results | There is no selective reporting of results. | Yes |  |
| **Statistical Analysis and Presentation Summary** | The statistical analysis is appropriate for the design of the study, limiting potential for presentation of invalid or spurious results. |  | Low |
|  | | | |
| Study title: | Adult herpes simplex encephalitis: Fifteen years’ experience | | |
| Study author/ data: | Riera-Mestre et al., 2009 | | |
| Study identifier: | 10.1016/j.eimc.2008.05.006 | | |
|  | | | |
| Biases | Issues to consider for judging overall rating of "Risk of bias" | Rating of reporting | Rating of "Risk of bias" |
| **1. Study Participation** | Goal: To judge the risk of selection bias (likelihood that relationship between PF and outcome is different for participants and eligible non-participants). |  |  |
| Source of target population | The source population or population of interest is adequately described for key characteristics (LIST). | Partial |  |
| Method used to identify population | The sampling frame and recruitment are adequately described, including methods to identify the sample sufficient to limit potential bias (number and type used, e.g., referral patterns in health care) | Partial |  |
| Recruitment period | Period of recruitment is adequately described | Yes |  |
| Place of recruitment | Place of recruitment (setting and geographic location) are adequately described | Yes |  |
| Inclusion and exclusion criteria | Inclusion and exclusion criteria are adequately described (e.g., including explicit diagnostic criteria or “zero time” description). | No |  |
| Adequate study participation | There is adequate participation in the study by eligible individuals | Yes |  |
| Baseline characteristics | The baseline study sample (i.e., individuals entering the study) is adequately described for key characteristics (LIST). | Partial |  |
| **Summary Study participation** | The study sample represents the population of interest on key characteristics, sufficient to limit potential bias of the observed relationship between PF and outcome. |  | Moderate |
|  | | | |
| **2. Study Attrition** | Goal: To judge the risk of attrition bias (likelihood that relationship between PF and outcome are different for completing and non-completing participants). |  |  |
| Proportion of baseline sample available for analysis | Response rate (i.e., proportion of study sample completing the study and providing outcome data) is adequate. | Yes |  |
| Attempts to collect information on participants who dropped out | Attempts to collect information on participants who dropped out of the study are described. | No |  |
| Reasons and potential impact of subjects lost to follow-up | Reasons for loss to follow-up are provided. | No |  |
| Outcome and prognostic factor information on those lost to follow-up | Participants lost to follow-up are adequately described for key characteristics (LIST)./ There are no important differences between key characteristics (LIST) and outcomes in participants who completed the study and those who did not. | No |  |
| **Study Attrition Summary** | Loss to follow-up (from baseline sample to study population analyzed) is not associated with key characteristics (i.e., the study data adequately represent the sample) sufficient to limit potential bias to the observed relationship between PF and outcome. |  | Low |
|  | | | |
| **3. Prognostic Factor Measurement** | Goal: To judge the risk of measurement bias related to how PF was measured (differential measurement of PF related to the level of outcome). |  |  |
| Definition of the PF | A clear definition or description of 'PF' is provided (e.g., including dose, level, duration of exposure, and clear specification of the method of measurement). | Yes |  |
| Valid and Reliable Measurement of PF | Method of PF measurement is adequately valid and reliable to limit misclassification bias (e.g., may include relevant outside sources of information on measurement properties, also characteristics, such as blind measurement and limited reliance on recall)./ Continuous variables are reported or appropriate cut-points (i.e., not data-dependent) are used. | Yes |  |
| Method and Setting of PF Measurement | The method and setting of measurement of PF is the same for all study participants. | Yes |  |
| Proportion of data on PF available for analysis | Adequate proportion of the study sample has complete data for PF variable. | Yes |  |
| Method used for missing data | Appropriate methods of imputation are used for missing 'PF' data. | No |  |
| **PF Measurement Summary** | PF is adequately measured in study participants to sufficiently limit potential bias. |  | Low |
|  | | | |
| 4. Outcome Measurement | Goal: To judge the risk of bias related to the measurement of outcome (differential measurement of outcome related to the baseline level of PF). |  |  |
| Definition of the Outcome | A clear definition of outcome is provided, including duration of follow-up and level and extent of the outcome construct. | Yes |  |
| Valid and Reliable Measurement of Outcome | The method of outcome measurement used is adequately valid and reliable to limit misclassification bias (e.g., may include relevant outside sources of information on measurement properties, also characteristics, such as blind measurement and confirmation of outcome with valid and reliable test). | Yes |  |
| Method and Setting of Outcome Measurement | The method and setting of outcome measurement is the same for all study participants. | Yes |  |
| **Outcome Measurement Summary** | Outcome of interest is adequately measured in study participants to sufficiently limit potential bias. |  | Low |
|  | | | |
| **5. Study Confounding** | Goal: To judge the risk of bias due to confounding (i.e. the effect of PF is distorted by another factor that is related to PF and outcome). |  |  |
| Important Confounders Measured | All important confounders, including treatments (key variables in conceptual model: LIST), are measured. | Yes |  |
| Definition of the confounding factor | Clear definitions of the important confounders measured are provided (e.g., including dose, level, and duration of exposures). | Yes |  |
| Valid and Reliable Measurement of Confounders | Measurement of all important confounders is adequately valid and reliable (e.g., may include relevant outside sources of information on measurement properties, also characteristics, such as blind measurement and limited reliance on recall). | Yes |  |
| Method and Setting of Confounding Measurement | The method and setting of confounding measurement are the same for all study participants. | Yes |  |
| Method used for missing data | Appropriate methods are used if imputation is used for missing confounder data. | No |  |
| Appropriate Accounting for Confounding | Important potential confounders are accounted for in the study design (e.g., matching for key variables, stratification, or initial assembly of comparable groups). /Important potential confounders are accounted for in the analysis (i.e., appropriate adjustment). | Yes |  |
| **Study Confounding Summary** | Important potential confounders are appropriately accounted for, limiting potential bias with respect to the relationship between PF and outcome . |  | Low |
|  | | | |
| **6. Statistical Analysis and Reporting** | Goal: To judge the risk of bias related to the statistical analysis and presentation of results. |  |  |
| Presentation of analytical strategy | There is sufficient presentation of data to assess the adequacy of the analysis. | Yes |  |
| Model development strategy | The strategy for model building (i.e., inclusion of variables in the statistical model) is appropriate and is based on a conceptual framework or model./ The selected statistical model is adequate for the design of the study. | Yes |  |
| Reporting of results | There is no selective reporting of results. | Yes |  |
| **Statistical Analysis and Presentation Summary** | The statistical analysis is appropriate for the design of the study, limiting potential for presentation of invalid or spurious results. |  | Low |
|  | | | |
| Study title: | Outcome of children with Japanese encephalitis and predictors of outcome in southwestern China | | |
| Study author/ data: | Ma and Jiang et al., 2013 | | |
| Study identifier: | 10.1093/trstmh/trt064 | | |
|  | | | |
| Biases | Issues to consider for judging overall rating of "Risk of bias" | Rating of reporting | Rating of "Risk of bias" |
| **1. Study Participation** | Goal: To judge the risk of selection bias (likelihood that relationship between PF and outcome is different for participants and eligible non-participants). |  |  |
| Source of target population | The source population or population of interest is adequately described for key characteristics (LIST). | Partial |  |
| Method used to identify population | The sampling frame and recruitment are adequately described, including methods to identify the sample sufficient to limit potential bias (number and type used, e.g., referral patterns in health care) | Partial |  |
| Recruitment period | Period of recruitment is adequately described | Yes |  |
| Place of recruitment | Place of recruitment (setting and geographic location) are adequately described | Yes |  |
| Inclusion and exclusion criteria | Inclusion and exclusion criteria are adequately described (e.g., including explicit diagnostic criteria or “zero time” description). | Partial |  |
| Adequate study participation | There is adequate participation in the study by eligible individuals | Yes |  |
| Baseline characteristics | The baseline study sample (i.e., individuals entering the study) is adequately described for key characteristics (LIST). | Yes |  |
| **Summary Study participation** | The study sample represents the population of interest on key characteristics, sufficient to limit potential bias of the observed relationship between PF and outcome. |  | Low |
|  | | | |
| **2. Study Attrition** | Goal: To judge the risk of attrition bias (likelihood that relationship between PF and outcome are different for completing and non-completing participants). |  |  |
| Proportion of baseline sample available for analysis | Response rate (i.e., proportion of study sample completing the study and providing outcome data) is adequate. | Yes |  |
| Attempts to collect information on participants who dropped out | Attempts to collect information on participants who dropped out of the study are described. | Partial |  |
| Reasons and potential impact of subjects lost to follow-up | Reasons for loss to follow-up are provided. | Yes |  |
| Outcome and prognostic factor information on those lost to follow-up | Participants lost to follow-up are adequately described for key characteristics (LIST)./ There are no important differences between key characteristics (LIST) and outcomes in participants who completed the study and those who did not. | No |  |
| **Study Attrition Summary** | Loss to follow-up (from baseline sample to study population analyzed) is not associated with key characteristics (i.e., the study data adequately represent the sample) sufficient to limit potential bias to the observed relationship between PF and outcome. |  | Moderate |
|  | | | |
| **3. Prognostic Factor Measurement** | Goal: To judge the risk of measurement bias related to how PF was measured (differential measurement of PF related to the level of outcome). |  |  |
| Definition of the PF | A clear definition or description of 'PF' is provided (e.g., including dose, level, duration of exposure, and clear specification of the method of measurement). | Partial |  |
| Valid and Reliable Measurement of PF | Method of PF measurement is adequately valid and reliable to limit misclassification bias (e.g., may include relevant outside sources of information on measurement properties, also characteristics, such as blind measurement and limited reliance on recall)./ Continuous variables are reported or appropriate cut-points (i.e., not data-dependent) are used. | Yes |  |
| Method and Setting of PF Measurement | The method and setting of measurement of PF is the same for all study participants. | Yes |  |
| Proportion of data on PF available for analysis | Adequate proportion of the study sample has complete data for PF variable. | Yes |  |
| Method used for missing data | Appropriate methods of imputation are used for missing 'PF' data. | No |  |
| **PF Measurement Summary** | PF is adequately measured in study participants to sufficiently limit potential bias. |  | Low |
|  | | | |
| 4. Outcome Measurement | Goal: To judge the risk of bias related to the measurement of outcome (differential measurement of outcome related to the baseline level of PF). |  |  |
| Definition of the Outcome | A clear definition of outcome is provided, including duration of follow-up and level and extent of the outcome construct. | Yes |  |
| Valid and Reliable Measurement of Outcome | The method of outcome measurement used is adequately valid and reliable to limit misclassification bias (e.g., may include relevant outside sources of information on measurement properties, also characteristics, such as blind measurement and confirmation of outcome with valid and reliable test). | Yes |  |
| Method and Setting of Outcome Measurement | The method and setting of outcome measurement is the same for all study participants. | Yes |  |
| **Outcome Measurement Summary** | Outcome of interest is adequately measured in study participants to sufficiently limit potential bias. |  | Low |
|  | | | |
| **5. Study Confounding** | Goal: To judge the risk of bias due to confounding (i.e. the effect of PF is distorted by another factor that is related to PF and outcome). |  |  |
| Important Confounders Measured | All important confounders, including treatments (key variables in conceptual model: LIST), are measured. | Partial |  |
| Definition of the confounding factor | Clear definitions of the important confounders measured are provided (e.g., including dose, level, and duration of exposures). | Partial |  |
| Valid and Reliable Measurement of Confounders | Measurement of all important confounders is adequately valid and reliable (e.g., may include relevant outside sources of information on measurement properties, also characteristics, such as blind measurement and limited reliance on recall). | Partial |  |
| Method and Setting of Confounding Measurement | The method and setting of confounding measurement are the same for all study participants. | Unsure |  |
| Method used for missing data | Appropriate methods are used if imputation is used for missing confounder data. | No |  |
| Appropriate Accounting for Confounding | Important potential confounders are accounted for in the study design (e.g., matching for key variables, stratification, or initial assembly of comparable groups). /Important potential confounders are accounted for in the analysis (i.e., appropriate adjustment). | Yes |  |
| **Study Confounding Summary** | Important potential confounders are appropriately accounted for, limiting potential bias with respect to the relationship between PF and outcome . |  | Low |
|  | | | |
| **6. Statistical Analysis and Reporting** | Goal: To judge the risk of bias related to the statistical analysis and presentation of results. |  |  |
| Presentation of analytical strategy | There is sufficient presentation of data to assess the adequacy of the analysis. | Yes |  |
| Model development strategy | The strategy for model building (i.e., inclusion of variables in the statistical model) is appropriate and is based on a conceptual framework or model./ The selected statistical model is adequate for the design of the study. | Yes |  |
| Reporting of results | There is no selective reporting of results. | Yes |  |
| **Statistical Analysis and Presentation Summary** | The statistical analysis is appropriate for the design of the study, limiting potential for presentation of invalid or spurious results. |  | Low |
|  | | | |
| Study title: | Clinical Value of Dorsal Medulla Oblongata Involvement Detected With Conventional Magnetic Resonance Imaging for Prediction of Outcome in Children With Enterovirus 71-Related Brainstem Encephalitis | | |
| Study author/ data: | Liu et al., 2019 | | |
| Study identifier: | 10.1097/INF.0000000000002041 | | |
|  | | | |
| Biases | Issues to consider for judging overall rating of "Risk of bias" | Rating of reporting | Rating of "Risk of bias" |
| **1. Study Participation** | Goal: To judge the risk of selection bias (likelihood that relationship between PF and outcome is different for participants and eligible non-participants). |  |  |
| Source of target population | The source population or population of interest is adequately described for key characteristics (LIST). | No |  |
| Method used to identify population | The sampling frame and recruitment are adequately described, including methods to identify the sample sufficient to limit potential bias (number and type used, e.g., referral patterns in health care) | Partial |  |
| Recruitment period | Period of recruitment is adequately described | Yes |  |
| Place of recruitment | Place of recruitment (setting and geographic location) are adequately described | Yes |  |
| Inclusion and exclusion criteria | Inclusion and exclusion criteria are adequately described (e.g., including explicit diagnostic criteria or “zero time” description). | Yes |  |
| Adequate study participation | There is adequate participation in the study by eligible individuals | Yes |  |
| Baseline characteristics | The baseline study sample (i.e., individuals entering the study) is adequately described for key characteristics (LIST). | Yes |  |
| **Summary Study participation** | The study sample represents the population of interest on key characteristics, sufficient to limit potential bias of the observed relationship between PF and outcome. |  | Low |
|  | | | |
| **2. Study Attrition** | Goal: To judge the risk of attrition bias (likelihood that relationship between PF and outcome are different for completing and non-completing participants). |  |  |
| Proportion of baseline sample available for analysis | Response rate (i.e., proportion of study sample completing the study and providing outcome data) is adequate. | Yes |  |
| Attempts to collect information on participants who dropped out | Attempts to collect information on participants who dropped out of the study are described. | No |  |
| Reasons and potential impact of subjects lost to follow-up | Reasons for loss to follow-up are provided. | No |  |
| Outcome and prognostic factor information on those lost to follow-up | Participants lost to follow-up are adequately described for key characteristics (LIST)./ There are no important differences between key characteristics (LIST) and outcomes in participants who completed the study and those who did not. | No |  |
| **Study Attrition Summary** | Loss to follow-up (from baseline sample to study population analyzed) is not associated with key characteristics (i.e., the study data adequately represent the sample) sufficient to limit potential bias to the observed relationship between PF and outcome. |  | Low |
|  | | | |
| **3. Prognostic Factor Measurement** | Goal: To judge the risk of measurement bias related to how PF was measured (differential measurement of PF related to the level of outcome). |  |  |
| Definition of the PF | A clear definition or description of 'PF' is provided (e.g., including dose, level, duration of exposure, and clear specification of the method of measurement). | Partial |  |
| Valid and Reliable Measurement of PF | Method of PF measurement is adequately valid and reliable to limit misclassification bias (e.g., may include relevant outside sources of information on measurement properties, also characteristics, such as blind measurement and limited reliance on recall)./ Continuous variables are reported or appropriate cut-points (i.e., not data-dependent) are used. | Partial |  |
| Method and Setting of PF Measurement | The method and setting of measurement of PF is the same for all study participants. | Partial |  |
| Proportion of data on PF available for analysis | Adequate proportion of the study sample has complete data for PF variable. | Yes |  |
| Method used for missing data | Appropriate methods of imputation are used for missing 'PF' data. | No |  |
| **PF Measurement Summary** | PF is adequately measured in study participants to sufficiently limit potential bias. |  | Moderate |
|  | | | |
| 4. Outcome Measurement | Goal: To judge the risk of bias related to the measurement of outcome (differential measurement of outcome related to the baseline level of PF). |  |  |
| Definition of the Outcome | A clear definition of outcome is provided, including duration of follow-up and level and extent of the outcome construct. | Yes |  |
| Valid and Reliable Measurement of Outcome | The method of outcome measurement used is adequately valid and reliable to limit misclassification bias (e.g., may include relevant outside sources of information on measurement properties, also characteristics, such as blind measurement and confirmation of outcome with valid and reliable test). | Yes |  |
| Method and Setting of Outcome Measurement | The method and setting of outcome measurement is the same for all study participants. | Partial |  |
| **Outcome Measurement Summary** | Outcome of interest is adequately measured in study participants to sufficiently limit potential bias. |  | Low |
|  | | | |
| **5. Study Confounding** | Goal: To judge the risk of bias due to confounding (i.e. the effect of PF is distorted by another factor that is related to PF and outcome). |  |  |
| Important Confounders Measured | All important confounders, including treatments (key variables in conceptual model: LIST), are measured. | Yes |  |
| Definition of the confounding factor | Clear definitions of the important confounders measured are provided (e.g., including dose, level, and duration of exposures). | Partial |  |
| Valid and Reliable Measurement of Confounders | Measurement of all important confounders is adequately valid and reliable (e.g., may include relevant outside sources of information on measurement properties, also characteristics, such as blind measurement and limited reliance on recall). | Partial |  |
| Method and Setting of Confounding Measurement | The method and setting of confounding measurement are the same for all study participants. | Yes |  |
| Method used for missing data | Appropriate methods are used if imputation is used for missing confounder data. | No |  |
| Appropriate Accounting for Confounding | Important potential confounders are accounted for in the study design (e.g., matching for key variables, stratification, or initial assembly of comparable groups). /Important potential confounders are accounted for in the analysis (i.e., appropriate adjustment). | Partial |  |
| **Study Confounding Summary** | Important potential confounders are appropriately accounted for, limiting potential bias with respect to the relationship between PF and outcome . |  | Moderate |
|  | | | |
| **6. Statistical Analysis and Reporting** | Goal: To judge the risk of bias related to the statistical analysis and presentation of results. |  |  |
| Presentation of analytical strategy | There is sufficient presentation of data to assess the adequacy of the analysis. | Yes |  |
| Model development strategy | The strategy for model building (i.e., inclusion of variables in the statistical model) is appropriate and is based on a conceptual framework or model./ The selected statistical model is adequate for the design of the study. | Yes |  |
| Reporting of results | There is no selective reporting of results. | Unsure |  |
| **Statistical Analysis and Presentation Summary** | The statistical analysis is appropriate for the design of the study, limiting potential for presentation of invalid or spurious results. |  | Low |
|  | | | |
| Study title: | Viral Encephalitis in Children: Detection with Technetium-99m HMPAO Brain Single-Photon Emission CT and Its Value in Prediction of Outcome | | |
| Study author/ data: | Kao et al., 1994 | | |
| Study identifier: | PMID: 7976951 | | |
|  | | | |
| Biases | Issues to consider for judging overall rating of "Risk of bias" | Rating of reporting | Rating of "Risk of bias" |
| **1. Study Participation** | Goal: To judge the risk of selection bias (likelihood that relationship between PF and outcome is different for participants and eligible non-participants). |  |  |
| Source of target population | The source population or population of interest is adequately described for key characteristics (LIST). | No |  |
| Method used to identify population | The sampling frame and recruitment are adequately described, including methods to identify the sample sufficient to limit potential bias (number and type used, e.g., referral patterns in health care) | No |  |
| Recruitment period | Period of recruitment is adequately described | No |  |
| Place of recruitment | Place of recruitment (setting and geographic location) are adequately described | No |  |
| Inclusion and exclusion criteria | Inclusion and exclusion criteria are adequately described (e.g., including explicit diagnostic criteria or “zero time” description). | Partial |  |
| Adequate study participation | There is adequate participation in the study by eligible individuals | No |  |
| Baseline characteristics | The baseline study sample (i.e., individuals entering the study) is adequately described for key characteristics (LIST). | No |  |
| **Summary Study participation** | The study sample represents the population of interest on key characteristics, sufficient to limit potential bias of the observed relationship between PF and outcome. |  | High |
|  | | | |
| **2. Study Attrition** | Goal: To judge the risk of attrition bias (likelihood that relationship between PF and outcome are different for completing and non-completing participants). |  |  |
| Proportion of baseline sample available for analysis | Response rate (i.e., proportion of study sample completing the study and providing outcome data) is adequate. | No |  |
| Attempts to collect information on participants who dropped out | Attempts to collect information on participants who dropped out of the study are described. | No |  |
| Reasons and potential impact of subjects lost to follow-up | Reasons for loss to follow-up are provided. | No |  |
| Outcome and prognostic factor information on those lost to follow-up | Participants lost to follow-up are adequately described for key characteristics (LIST)./ There are no important differences between key characteristics (LIST) and outcomes in participants who completed the study and those who did not. | No |  |
| **Study Attrition Summary** | Loss to follow-up (from baseline sample to study population analyzed) is not associated with key characteristics (i.e., the study data adequately represent the sample) sufficient to limit potential bias to the observed relationship between PF and outcome. |  | Low |
|  | | | |
| **3. Prognostic Factor Measurement** | Goal: To judge the risk of measurement bias related to how PF was measured (differential measurement of PF related to the level of outcome). |  |  |
| Definition of the PF | A clear definition or description of 'PF' is provided (e.g., including dose, level, duration of exposure, and clear specification of the method of measurement). | No |  |
| Valid and Reliable Measurement of PF | Method of PF measurement is adequately valid and reliable to limit misclassification bias (e.g., may include relevant outside sources of information on measurement properties, also characteristics, such as blind measurement and limited reliance on recall)./ Continuous variables are reported or appropriate cut-points (i.e., not data-dependent) are used. | Unsure |  |
| Method and Setting of PF Measurement | The method and setting of measurement of PF is the same for all study participants. | Yes |  |
| Proportion of data on PF available for analysis | Adequate proportion of the study sample has complete data for PF variable. | Partial |  |
| Method used for missing data | Appropriate methods of imputation are used for missing 'PF' data. | No |  |
| **PF Measurement Summary** | PF is adequately measured in study participants to sufficiently limit potential bias. |  | High |
|  | | | |
| 4. Outcome Measurement | Goal: To judge the risk of bias related to the measurement of outcome (differential measurement of outcome related to the baseline level of PF). |  |  |
| Definition of the Outcome | A clear definition of outcome is provided, including duration of follow-up and level and extent of the outcome construct. | No |  |
| Valid and Reliable Measurement of Outcome | The method of outcome measurement used is adequately valid and reliable to limit misclassification bias (e.g., may include relevant outside sources of information on measurement properties, also characteristics, such as blind measurement and confirmation of outcome with valid and reliable test). | No |  |
| Method and Setting of Outcome Measurement | The method and setting of outcome measurement is the same for all study participants. | Partial |  |
| **Outcome Measurement Summary** | Outcome of interest is adequately measured in study participants to sufficiently limit potential bias. |  | Moderate |
|  | | | |
| **5. Study Confounding** | Goal: To judge the risk of bias due to confounding (i.e. the effect of PF is distorted by another factor that is related to PF and outcome). |  |  |
| Important Confounders Measured | All important confounders, including treatments (key variables in conceptual model: LIST), are measured. | No |  |
| Definition of the confounding factor | Clear definitions of the important confounders measured are provided (e.g., including dose, level, and duration of exposures). | No |  |
| Valid and Reliable Measurement of Confounders | Measurement of all important confounders is adequately valid and reliable (e.g., may include relevant outside sources of information on measurement properties, also characteristics, such as blind measurement and limited reliance on recall). | No |  |
| Method and Setting of Confounding Measurement | The method and setting of confounding measurement are the same for all study participants. | No |  |
| Method used for missing data | Appropriate methods are used if imputation is used for missing confounder data. | No |  |
| Appropriate Accounting for Confounding | Important potential confounders are accounted for in the study design (e.g., matching for key variables, stratification, or initial assembly of comparable groups). /Important potential confounders are accounted for in the analysis (i.e., appropriate adjustment). | No |  |
| **Study Confounding Summary** | Important potential confounders are appropriately accounted for, limiting potential bias with respect to the relationship between PF and outcome . |  | High |
|  | | | |
| **6. Statistical Analysis and Reporting** | Goal: To judge the risk of bias related to the statistical analysis and presentation of results. |  |  |
| Presentation of analytical strategy | There is sufficient presentation of data to assess the adequacy of the analysis. | No |  |
| Model development strategy | The strategy for model building (i.e., inclusion of variables in the statistical model) is appropriate and is based on a conceptual framework or model./ The selected statistical model is adequate for the design of the study. | No |  |
| Reporting of results | There is no selective reporting of results. | Unsure |  |
| **Statistical Analysis and Presentation Summary** | The statistical analysis is appropriate for the design of the study, limiting potential for presentation of invalid or spurious results. |  | High |
|  | | | |
| Study title: | Extent of disability among paediatric Japanese encephalitis survivors and predictors of poor outcome: a retrospective cohort study in North India | | |
| Study author/ data: | Srivastava et al., 2022 |  |  |
| Study identifier: | 10.1136/bmjopen-2022- 060795 |  |  |
|  | | | |
| Biases | Issues to consider for judging overall rating of "Risk of bias" | Rating of reporting | Rating of "Risk of bias" |
| **1. Study Participation** | Goal: To judge the risk of selection bias (likelihood that relationship between PF and outcome is different for participants and eligible non-participants). |  |  |
| Source of target population | The source population or population of interest is adequately described for key characteristics (LIST). | Yes |  |
| Method used to identify population | The sampling frame and recruitment are adequately described, including methods to identify the sample sufficient to limit potential bias (number and type used, e.g., referral patterns in health care) | Partial |  |
| Recruitment period | Period of recruitment is adequately described | Yes |  |
| Place of recruitment | Place of recruitment (setting and geographic location) are adequately described | Yes |  |
| Inclusion and exclusion criteria | Inclusion and exclusion criteria are adequately described (e.g., including explicit diagnostic criteria or “zero time” description). | Yes |  |
| Adequate study participation | There is adequate participation in the study by eligible individuals | Yes |  |
| Baseline characteristics | The baseline study sample (i.e., individuals entering the study) is adequately described for key characteristics (LIST). | Yes |  |
| **Summary Study participation** | The study sample represents the population of interest on key characteristics, sufficient to limit potential bias of the observed relationship between PF and outcome. |  | Low |
|  | | | |
| **2. Study Attrition** | Goal: To judge the risk of attrition bias (likelihood that relationship between PF and outcome are different for completing and non-completing participants). |  |  |
| Proportion of baseline sample available for analysis | Response rate (i.e., proportion of study sample completing the study and providing outcome data) is adequate. | Yes |  |
| Attempts to collect information on participants who dropped out | Attempts to collect information on participants who dropped out of the study are described. | Yes |  |
| Reasons and potential impact of subjects lost to follow-up | Reasons for loss to follow-up are provided. | Yes |  |
| Outcome and prognostic factor information on those lost to follow-up | Participants lost to follow-up are adequately described for key characteristics (LIST)./ There are no important differences between key characteristics (LIST) and outcomes in participants who completed the study and those who did not. | Partial |  |
| **Study Attrition Summary** | Loss to follow-up (from baseline sample to study population analyzed) is not associated with key characteristics (i.e., the study data adequately represent the sample) sufficient to limit potential bias to the observed relationship between PF and outcome. |  | Low |
|  | | | |
| **3. Prognostic Factor Measurement** | Goal: To judge the risk of measurement bias related to how PF was measured (differential measurement of PF related to the level of outcome). |  |  |
| Definition of the PF | A clear definition or description of 'PF' is provided (e.g., including dose, level, duration of exposure, and clear specification of the method of measurement). | Partial |  |
| Valid and Reliable Measurement of PF | Method of PF measurement is adequately valid and reliable to limit misclassification bias (e.g., may include relevant outside sources of information on measurement properties, also characteristics, such as blind measurement and limited reliance on recall)./ Continuous variables are reported or appropriate cut-points (i.e., not data-dependent) are used. | Yes |  |
| Method and Setting of PF Measurement | The method and setting of measurement of PF is the same for all study participants. | Yes |  |
| Proportion of data on PF available for analysis | Adequate proportion of the study sample has complete data for PF variable. | Yes |  |
| Method used for missing data | Appropriate methods of imputation are used for missing 'PF' data. | No |  |
| **PF Measurement Summary** | PF is adequately measured in study participants to sufficiently limit potential bias. |  | Low |
|  | | | |
| 4. Outcome Measurement | Goal: To judge the risk of bias related to the measurement of outcome (differential measurement of outcome related to the baseline level of PF). |  |  |
| Definition of the Outcome | A clear definition of outcome is provided, including duration of follow-up and level and extent of the outcome construct. | Yes |  |
| Valid and Reliable Measurement of Outcome | The method of outcome measurement used is adequately valid and reliable to limit misclassification bias (e.g., may include relevant outside sources of information on measurement properties, also characteristics, such as blind measurement and confirmation of outcome with valid and reliable test). | Yes |  |
| Method and Setting of Outcome Measurement | The method and setting of outcome measurement is the same for all study participants. | Yes |  |
| **Outcome Measurement Summary** | Outcome of interest is adequately measured in study participants to sufficiently limit potential bias. |  | Low |
|  | | | |
| **5. Study Confounding** | Goal: To judge the risk of bias due to confounding (i.e. the effect of PF is distorted by another factor that is related to PF and outcome). |  |  |
| Important Confounders Measured | All important confounders, including treatments (key variables in conceptual model: LIST), are measured. | Yes |  |
| Definition of the confounding factor | Clear definitions of the important confounders measured are provided (e.g., including dose, level, and duration of exposures). | Partial |  |
| Valid and Reliable Measurement of Confounders | Measurement of all important confounders is adequately valid and reliable (e.g., may include relevant outside sources of information on measurement properties, also characteristics, such as blind measurement and limited reliance on recall). | Partial |  |
| Method and Setting of Confounding Measurement | The method and setting of confounding measurement are the same for all study participants. | Yes |  |
| Method used for missing data | Appropriate methods are used if imputation is used for missing confounder data. | Yes |  |
| Appropriate Accounting for Confounding | Important potential confounders are accounted for in the study design (e.g., matching for key variables, stratification, or initial assembly of comparable groups). /Important potential confounders are accounted for in the analysis (i.e., appropriate adjustment). | Yes |  |
| **Study Confounding Summary** | Important potential confounders are appropriately accounted for, limiting potential bias with respect to the relationship between PF and outcome . |  | Low |
|  | | | |
| **6. Statistical Analysis and Reporting** | Goal: To judge the risk of bias related to the statistical analysis and presentation of results. |  |  |
| Presentation of analytical strategy | There is sufficient presentation of data to assess the adequacy of the analysis. | Yes |  |
| Model development strategy | The strategy for model building (i.e., inclusion of variables in the statistical model) is appropriate and is based on a conceptual framework or model./ The selected statistical model is adequate for the design of the study. | Yes |  |
| Reporting of results | There is no selective reporting of results. | Yes |  |
| **Statistical Analysis and Presentation Summary** | The statistical analysis is appropriate for the design of the study, limiting potential for presentation of invalid or spurious results. |  | Low |
|  | | | |
| Study title: | Neurologic outcome of VZV encephalitis one year after ICU admission: a multicenter cohort study | | |
| Study author/ data: | Mirouse et al., 2020 | | |
| Study identifier: | 10.1186/s13613-022-01002-y | | |
|  | | | |
| Biases | Issues to consider for judging overall rating of "Risk of bias" | Rating of reporting | Rating of "Risk of bias" |
| **1. Study Participation** | Goal: To judge the risk of selection bias (likelihood that relationship between PF and outcome is different for participants and eligible non-participants). |  |  |
| Source of target population | The source population or population of interest is adequately described for key characteristics (LIST). | Yes |  |
| Method used to identify population | The sampling frame and recruitment are adequately described, including methods to identify the sample sufficient to limit potential bias (number and type used, e.g., referral patterns in health care) | Yes |  |
| Recruitment period | Period of recruitment is adequately described | Yes |  |
| Place of recruitment | Place of recruitment (setting and geographic location) are adequately described | Yes |  |
| Inclusion and exclusion criteria | Inclusion and exclusion criteria are adequately described (e.g., including explicit diagnostic criteria or “zero time” description). | Yes |  |
| Adequate study participation | There is adequate participation in the study by eligible individuals | Yes |  |
| Baseline characteristics | The baseline study sample (i.e., individuals entering the study) is adequately described for key characteristics (LIST). | Yes |  |
| **Summary Study participation** | The study sample represents the population of interest on key characteristics, sufficient to limit potential bias of the observed relationship between PF and outcome. |  | Low |
|  | | | |
| **2. Study Attrition** | Goal: To judge the risk of attrition bias (likelihood that relationship between PF and outcome are different for completing and non-completing participants). |  |  |
| Proportion of baseline sample available for analysis | Response rate (i.e., proportion of study sample completing the study and providing outcome data) is adequate. | Yes |  |
| Attempts to collect information on participants who dropped out | Attempts to collect information on participants who dropped out of the study are described. | No |  |
| Reasons and potential impact of subjects lost to follow-up | Reasons for loss to follow-up are provided. | Yes |  |
| Outcome and prognostic factor information on those lost to follow-up | Participants lost to follow-up are adequately described for key characteristics (LIST)./ There are no important differences between key characteristics (LIST) and outcomes in participants who completed the study and those who did not. | Yes |  |
| **Study Attrition Summary** | Loss to follow-up (from baseline sample to study population analyzed) is not associated with key characteristics (i.e., the study data adequately represent the sample) sufficient to limit potential bias to the observed relationship between PF and outcome. |  | Low |
|  | | | |
| **3. Prognostic Factor Measurement** | Goal: To judge the risk of measurement bias related to how PF was measured (differential measurement of PF related to the level of outcome). |  |  |
| Definition of the PF | A clear definition or description of 'PF' is provided (e.g., including dose, level, duration of exposure, and clear specification of the method of measurement). | Yes |  |
| Valid and Reliable Measurement of PF | Method of PF measurement is adequately valid and reliable to limit misclassification bias (e.g., may include relevant outside sources of information on measurement properties, also characteristics, such as blind measurement and limited reliance on recall)./ Continuous variables are reported or appropriate cut-points (i.e., not data-dependent) are used. | Yes |  |
| Method and Setting of PF Measurement | The method and setting of measurement of PF is the same for all study participants. | Yes |  |
| Proportion of data on PF available for analysis | Adequate proportion of the study sample has complete data for PF variable. | Yes |  |
| Method used for missing data | Appropriate methods of imputation are used for missing 'PF' data. | Unsure |  |
| **PF Measurement Summary** | PF is adequately measured in study participants to sufficiently limit potential bias. |  | Low |
|  | | | |
| 4. Outcome Measurement | Goal: To judge the risk of bias related to the measurement of outcome (differential measurement of outcome related to the baseline level of PF). |  |  |
| Definition of the Outcome | A clear definition of outcome is provided, including duration of follow-up and level and extent of the outcome construct. | Yes |  |
| Valid and Reliable Measurement of Outcome | The method of outcome measurement used is adequately valid and reliable to limit misclassification bias (e.g., may include relevant outside sources of information on measurement properties, also characteristics, such as blind measurement and confirmation of outcome with valid and reliable test). | Yes |  |
| Method and Setting of Outcome Measurement | The method and setting of outcome measurement is the same for all study participants. | Yes |  |
| **Outcome Measurement Summary** | Outcome of interest is adequately measured in study participants to sufficiently limit potential bias. |  | Low |
|  | | | |
| **5. Study Confounding** | Goal: To judge the risk of bias due to confounding (i.e. the effect of PF is distorted by another factor that is related to PF and outcome). |  |  |
| Important Confounders Measured | All important confounders, including treatments (key variables in conceptual model: LIST), are measured. | Yes |  |
| Definition of the confounding factor | Clear definitions of the important confounders measured are provided (e.g., including dose, level, and duration of exposures). | Yes |  |
| Valid and Reliable Measurement of Confounders | Measurement of all important confounders is adequately valid and reliable (e.g., may include relevant outside sources of information on measurement properties, also characteristics, such as blind measurement and limited reliance on recall). | Yes |  |
| Method and Setting of Confounding Measurement | The method and setting of confounding measurement are the same for all study participants. | Yes |  |
| Method used for missing data | Appropriate methods are used if imputation is used for missing confounder data. | Yes |  |
| Appropriate Accounting for Confounding | Important potential confounders are accounted for in the study design (e.g., matching for key variables, stratification, or initial assembly of comparable groups). /Important potential confounders are accounted for in the analysis (i.e., appropriate adjustment). | Yes |  |
| **Study Confounding Summary** | Important potential confounders are appropriately accounted for, limiting potential bias with respect to the relationship between PF and outcome . |  | Low |
|  | | | |
| **6. Statistical Analysis and Reporting** | Goal: To judge the risk of bias related to the statistical analysis and presentation of results. |  |  |
| Presentation of analytical strategy | There is sufficient presentation of data to assess the adequacy of the analysis. | Yes |  |
| Model development strategy | The strategy for model building (i.e., inclusion of variables in the statistical model) is appropriate and is based on a conceptual framework or model./ The selected statistical model is adequate for the design of the study. | Yes |  |
| Reporting of results | There is no selective reporting of results. | Yes |  |
| **Statistical Analysis and Presentation Summary** | The statistical analysis is appropriate for the design of the study, limiting potential for presentation of invalid or spurious results. |  | Low |
|  | | | |
| Study title: | The neutrophil-to-lymphocyte and monocyte-to-lymphocyte ratios are independently associated with clinical outcomes of viral encephalitis | | |
| Study author/ data: | He et al., 2023 | | |
| Study identifier: | 10.3389/fneur.2022.1051865 | | |
|  | | | |
| Biases | Issues to consider for judging overall rating of "Risk of bias" | Rating of reporting | Rating of "Risk of bias" |
| **1. Study Participation** | Goal: To judge the risk of selection bias (likelihood that relationship between PF and outcome is different for participants and eligible non-participants). |  |  |
| Source of target population | The source population or population of interest is adequately described for key characteristics (LIST). | No |  |
| Method used to identify population | The sampling frame and recruitment are adequately described, including methods to identify the sample sufficient to limit potential bias (number and type used, e.g., referral patterns in health care) | Partial |  |
| Recruitment period | Period of recruitment is adequately described | Yes |  |
| Place of recruitment | Place of recruitment (setting and geographic location) are adequately described | Yes |  |
| Inclusion and exclusion criteria | Inclusion and exclusion criteria are adequately described (e.g., including explicit diagnostic criteria or “zero time” description). | Yes |  |
| Adequate study participation | There is adequate participation in the study by eligible individuals | Yes |  |
| Baseline characteristics | The baseline study sample (i.e., individuals entering the study) is adequately described for key characteristics (LIST). | Partial |  |
| **Summary Study participation** | The study sample represents the population of interest on key characteristics, sufficient to limit potential bias of the observed relationship between PF and outcome. |  | Moderate |
|  | | | |
| **2. Study Attrition** | Goal: To judge the risk of attrition bias (likelihood that relationship between PF and outcome are different for completing and non-completing participants). |  |  |
| Proportion of baseline sample available for analysis | Response rate (i.e., proportion of study sample completing the study and providing outcome data) is adequate. | Yes |  |
| Attempts to collect information on participants who dropped out | Attempts to collect information on participants who dropped out of the study are described. | No |  |
| Reasons and potential impact of subjects lost to follow-up | Reasons for loss to follow-up are provided. | No |  |
| Outcome and prognostic factor information on those lost to follow-up | Participants lost to follow-up are adequately described for key characteristics (LIST)./ There are no important differences between key characteristics (LIST) and outcomes in participants who completed the study and those who did not. | No |  |
| **Study Attrition Summary** | Loss to follow-up (from baseline sample to study population analyzed) is not associated with key characteristics (i.e., the study data adequately represent the sample) sufficient to limit potential bias to the observed relationship between PF and outcome. |  | Low |
|  | | | |
| **3. Prognostic Factor Measurement** | Goal: To judge the risk of measurement bias related to how PF was measured (differential measurement of PF related to the level of outcome). |  |  |
| Definition of the PF | A clear definition or description of 'PF' is provided (e.g., including dose, level, duration of exposure, and clear specification of the method of measurement). | Partial |  |
| Valid and Reliable Measurement of PF | Method of PF measurement is adequately valid and reliable to limit misclassification bias (e.g., may include relevant outside sources of information on measurement properties, also characteristics, such as blind measurement and limited reliance on recall)./ Continuous variables are reported or appropriate cut-points (i.e., not data-dependent) are used. | Unsure |  |
| Method and Setting of PF Measurement | The method and setting of measurement of PF is the same for all study participants. | Yes |  |
| Proportion of data on PF available for analysis | Adequate proportion of the study sample has complete data for PF variable. | Yes |  |
| Method used for missing data | Appropriate methods of imputation are used for missing 'PF' data. | No |  |
| **PF Measurement Summary** | PF is adequately measured in study participants to sufficiently limit potential bias. |  | Low |
|  | | | |
| 4. Outcome Measurement | Goal: To judge the risk of bias related to the measurement of outcome (differential measurement of outcome related to the baseline level of PF). |  |  |
| Definition of the Outcome | A clear definition of outcome is provided, including duration of follow-up and level and extent of the outcome construct. | Yes |  |
| Valid and Reliable Measurement of Outcome | The method of outcome measurement used is adequately valid and reliable to limit misclassification bias (e.g., may include relevant outside sources of information on measurement properties, also characteristics, such as blind measurement and confirmation of outcome with valid and reliable test). | Yes |  |
| Method and Setting of Outcome Measurement | The method and setting of outcome measurement is the same for all study participants. | Yes |  |
| **Outcome Measurement Summary** | Outcome of interest is adequately measured in study participants to sufficiently limit potential bias. |  | Low |
|  | | | |
| **5. Study Confounding** | Goal: To judge the risk of bias due to confounding (i.e. the effect of PF is distorted by another factor that is related to PF and outcome). |  |  |
| Important Confounders Measured | All important confounders, including treatments (key variables in conceptual model: LIST), are measured. | Yes |  |
| Definition of the confounding factor | Clear definitions of the important confounders measured are provided (e.g., including dose, level, and duration of exposures). | Yes |  |
| Valid and Reliable Measurement of Confounders | Measurement of all important confounders is adequately valid and reliable (e.g., may include relevant outside sources of information on measurement properties, also characteristics, such as blind measurement and limited reliance on recall). | Yes |  |
| Method and Setting of Confounding Measurement | The method and setting of confounding measurement are the same for all study participants. | Yes |  |
| Method used for missing data | Appropriate methods are used if imputation is used for missing confounder data. | Yes |  |
| Appropriate Accounting for Confounding | Important potential confounders are accounted for in the study design (e.g., matching for key variables, stratification, or initial assembly of comparable groups). /Important potential confounders are accounted for in the analysis (i.e., appropriate adjustment). | Yes |  |
| **Study Confounding Summary** | Important potential confounders are appropriately accounted for, limiting potential bias with respect to the relationship between PF and outcome . |  | Low |
|  | | | |
| **6. Statistical Analysis and Reporting** | Goal: To judge the risk of bias related to the statistical analysis and presentation of results. |  |  |
| Presentation of analytical strategy | There is sufficient presentation of data to assess the adequacy of the analysis. | Yes |  |
| Model development strategy | The strategy for model building (i.e., inclusion of variables in the statistical model) is appropriate and is based on a conceptual framework or model./ The selected statistical model is adequate for the design of the study. | Yes |  |
| Reporting of results | There is no selective reporting of results. | Yes |  |
| **Statistical Analysis and Presentation Summary** | The statistical analysis is appropriate for the design of the study, limiting potential for presentation of invalid or spurious results. |  | Low |
|  | | | |
| Study title: | Prognosis analysis of 112 cases with Japanese encephalitis in adults | | |
| Study author/ data: | Wang et al., 2020 | | |
| Study identifier: | 10.3760/cma.j.issn.0376-2491.2020.07.013 | | |
|  | | | |
| Biases | Issues to consider for judging overall rating of "Risk of bias" | Rating of reporting | Rating of "Risk of bias" |
| **1. Study Participation** | Goal: To judge the risk of selection bias (likelihood that relationship between PF and outcome is different for participants and eligible non-participants). |  |  |
| Source of target population | The source population or population of interest is adequately described for key characteristics (LIST). | No |  |
| Method used to identify population | The sampling frame and recruitment are adequately described, including methods to identify the sample sufficient to limit potential bias (number and type used, e.g., referral patterns in health care) | Partial |  |
| Recruitment period | Period of recruitment is adequately described | Yes |  |
| Place of recruitment | Place of recruitment (setting and geographic location) are adequately described | Yes |  |
| Inclusion and exclusion criteria | Inclusion and exclusion criteria are adequately described (e.g., including explicit diagnostic criteria or “zero time” description). | Partial |  |
| Adequate study participation | There is adequate participation in the study by eligible individuals | Yes |  |
| Baseline characteristics | The baseline study sample (i.e., individuals entering the study) is adequately described for key characteristics (LIST). | Partial |  |
| **Summary Study participation** | The study sample represents the population of interest on key characteristics, sufficient to limit potential bias of the observed relationship between PF and outcome. |  | Moderate |
|  | | | |
| **2. Study Attrition** | Goal: To judge the risk of attrition bias (likelihood that relationship between PF and outcome are different for completing and non-completing participants). |  |  |
| Proportion of baseline sample available for analysis | Response rate (i.e., proportion of study sample completing the study and providing outcome data) is adequate. | Yes |  |
| Attempts to collect information on participants who dropped out | Attempts to collect information on participants who dropped out of the study are described. | No |  |
| Reasons and potential impact of subjects lost to follow-up | Reasons for loss to follow-up are provided. | No |  |
| Outcome and prognostic factor information on those lost to follow-up | Participants lost to follow-up are adequately described for key characteristics (LIST)./ There are no important differences between key characteristics (LIST) and outcomes in participants who completed the study and those who did not. | No |  |
| **Study Attrition Summary** | Loss to follow-up (from baseline sample to study population analyzed) is not associated with key characteristics (i.e., the study data adequately represent the sample) sufficient to limit potential bias to the observed relationship between PF and outcome. |  | Low |
|  | | | |
| **3. Prognostic Factor Measurement** | Goal: To judge the risk of measurement bias related to how PF was measured (differential measurement of PF related to the level of outcome). |  |  |
| Definition of the PF | A clear definition or description of 'PF' is provided (e.g., including dose, level, duration of exposure, and clear specification of the method of measurement). | Partial |  |
| Valid and Reliable Measurement of PF | Method of PF measurement is adequately valid and reliable to limit misclassification bias (e.g., may include relevant outside sources of information on measurement properties, also characteristics, such as blind measurement and limited reliance on recall)./ Continuous variables are reported or appropriate cut-points (i.e., not data-dependent) are used. | Yes |  |
| Method and Setting of PF Measurement | The method and setting of measurement of PF is the same for all study participants. | Yes |  |
| Proportion of data on PF available for analysis | Adequate proportion of the study sample has complete data for PF variable. | Yes |  |
| Method used for missing data | Appropriate methods of imputation are used for missing 'PF' data. | No |  |
| **PF Measurement Summary** | PF is adequately measured in study participants to sufficiently limit potential bias. |  | Low |
|  | | | |
| 4. Outcome Measurement | Goal: To judge the risk of bias related to the measurement of outcome (differential measurement of outcome related to the baseline level of PF). |  |  |
| Definition of the Outcome | A clear definition of outcome is provided, including duration of follow-up and level and extent of the outcome construct. | Yes |  |
| Valid and Reliable Measurement of Outcome | The method of outcome measurement used is adequately valid and reliable to limit misclassification bias (e.g., may include relevant outside sources of information on measurement properties, also characteristics, such as blind measurement and confirmation of outcome with valid and reliable test). | Yes |  |
| Method and Setting of Outcome Measurement | The method and setting of outcome measurement is the same for all study participants. | Yes |  |
| **Outcome Measurement Summary** | Outcome of interest is adequately measured in study participants to sufficiently limit potential bias. |  | Low |
|  | | | |
| **5. Study Confounding** | Goal: To judge the risk of bias due to confounding (i.e. the effect of PF is distorted by another factor that is related to PF and outcome). |  |  |
| Important Confounders Measured | All important confounders, including treatments (key variables in conceptual model: LIST), are measured. | Yes |  |
| Definition of the confounding factor | Clear definitions of the important confounders measured are provided (e.g., including dose, level, and duration of exposures). | Partial |  |
| Valid and Reliable Measurement of Confounders | Measurement of all important confounders is adequately valid and reliable (e.g., may include relevant outside sources of information on measurement properties, also characteristics, such as blind measurement and limited reliance on recall). | Yes |  |
| Method and Setting of Confounding Measurement | The method and setting of confounding measurement are the same for all study participants. | Yes |  |
| Method used for missing data | Appropriate methods are used if imputation is used for missing confounder data. | Partial |  |
| Appropriate Accounting for Confounding | Important potential confounders are accounted for in the study design (e.g., matching for key variables, stratification, or initial assembly of comparable groups). /Important potential confounders are accounted for in the analysis (i.e., appropriate adjustment). | Yes |  |
| **Study Confounding Summary** | Important potential confounders are appropriately accounted for, limiting potential bias with respect to the relationship between PF and outcome . |  | Low |
|  | | | |
| **6. Statistical Analysis and Reporting** | Goal: To judge the risk of bias related to the statistical analysis and presentation of results. |  |  |
| Presentation of analytical strategy | There is sufficient presentation of data to assess the adequacy of the analysis. | Yes |  |
| Model development strategy | The strategy for model building (i.e., inclusion of variables in the statistical model) is appropriate and is based on a conceptual framework or model./ The selected statistical model is adequate for the design of the study. | Yes |  |
| Reporting of results | There is no selective reporting of results. | Yes |  |
| **Statistical Analysis and Presentation Summary** | The statistical analysis is appropriate for the design of the study, limiting potential for presentation of invalid or spurious results. |  | Low |
|  | | | |
| Study title: | Research on early diagnosis and impact prognostic factors of herpes simplex encephalitis | | |
| Study author/ data: | Zhang et al., 2016 | | |
| Study identifier: | ISSN:1940-5901/IJCEM0015720 | | |
|  | | | |
| Biases | Issues to consider for judging overall rating of "Risk of bias" | Rating of reporting | Rating of "Risk of bias" |
| **1. Study Participation** | Goal: To judge the risk of selection bias (likelihood that relationship between PF and outcome is different for participants and eligible non-participants). |  |  |
| Source of target population | The source population or population of interest is adequately described for key characteristics (LIST). | No |  |
| Method used to identify population | The sampling frame and recruitment are adequately described, including methods to identify the sample sufficient to limit potential bias (number and type used, e.g., referral patterns in health care) | Partial |  |
| Recruitment period | Period of recruitment is adequately described | Yes |  |
| Place of recruitment | Place of recruitment (setting and geographic location) are adequately described | Yes |  |
| Inclusion and exclusion criteria | Inclusion and exclusion criteria are adequately described (e.g., including explicit diagnostic criteria or “zero time” description). | No |  |
| Adequate study participation | There is adequate participation in the study by eligible individuals | Yes |  |
| Baseline characteristics | The baseline study sample (i.e., individuals entering the study) is adequately described for key characteristics (LIST). | No |  |
| **Summary Study participation** | The study sample represents the population of interest on key characteristics, sufficient to limit potential bias of the observed relationship between PF and outcome. |  | Moderate |
|  | | | |
| **2. Study Attrition** | Goal: To judge the risk of attrition bias (likelihood that relationship between PF and outcome are different for completing and non-completing participants). |  |  |
| Proportion of baseline sample available for analysis | Response rate (i.e., proportion of study sample completing the study and providing outcome data) is adequate. | Yes |  |
| Attempts to collect information on participants who dropped out | Attempts to collect information on participants who dropped out of the study are described. | No |  |
| Reasons and potential impact of subjects lost to follow-up | Reasons for loss to follow-up are provided. | No |  |
| Outcome and prognostic factor information on those lost to follow-up | Participants lost to follow-up are adequately described for key characteristics (LIST)./ There are no important differences between key characteristics (LIST) and outcomes in participants who completed the study and those who did not. | No |  |
| **Study Attrition Summary** | Loss to follow-up (from baseline sample to study population analyzed) is not associated with key characteristics (i.e., the study data adequately represent the sample) sufficient to limit potential bias to the observed relationship between PF and outcome. |  | Low |
|  | | | |
| **3. Prognostic Factor Measurement** | Goal: To judge the risk of measurement bias related to how PF was measured (differential measurement of PF related to the level of outcome). |  |  |
| Definition of the PF | A clear definition or description of 'PF' is provided (e.g., including dose, level, duration of exposure, and clear specification of the method of measurement). | No |  |
| Valid and Reliable Measurement of PF | Method of PF measurement is adequately valid and reliable to limit misclassification bias (e.g., may include relevant outside sources of information on measurement properties, also characteristics, such as blind measurement and limited reliance on recall)./ Continuous variables are reported or appropriate cut-points (i.e., not data-dependent) are used. | Unsure |  |
| Method and Setting of PF Measurement | The method and setting of measurement of PF is the same for all study participants. | Unsure |  |
| Proportion of data on PF available for analysis | Adequate proportion of the study sample has complete data for PF variable. | Yes |  |
| Method used for missing data | Appropriate methods of imputation are used for missing 'PF' data. | No |  |
| **PF Measurement Summary** | PF is adequately measured in study participants to sufficiently limit potential bias. |  | Moderate |
|  | | | |
| 4. Outcome Measurement | Goal: To judge the risk of bias related to the measurement of outcome (differential measurement of outcome related to the baseline level of PF). |  |  |
| Definition of the Outcome | A clear definition of outcome is provided, including duration of follow-up and level and extent of the outcome construct. | Yes |  |
| Valid and Reliable Measurement of Outcome | The method of outcome measurement used is adequately valid and reliable to limit misclassification bias (e.g., may include relevant outside sources of information on measurement properties, also characteristics, such as blind measurement and confirmation of outcome with valid and reliable test). | Yes |  |
| Method and Setting of Outcome Measurement | The method and setting of outcome measurement is the same for all study participants. | Yes |  |
| **Outcome Measurement Summary** | Outcome of interest is adequately measured in study participants to sufficiently limit potential bias. |  | No |
|  | | | |
| **5. Study Confounding** | Goal: To judge the risk of bias due to confounding (i.e. the effect of PF is distorted by another factor that is related to PF and outcome). |  |  |
| Important Confounders Measured | All important confounders, including treatments (key variables in conceptual model: LIST), are measured. | No |  |
| Definition of the confounding factor | Clear definitions of the important confounders measured are provided (e.g., including dose, level, and duration of exposures). | No |  |
| Valid and Reliable Measurement of Confounders | Measurement of all important confounders is adequately valid and reliable (e.g., may include relevant outside sources of information on measurement properties, also characteristics, such as blind measurement and limited reliance on recall). | Partial |  |
| Method and Setting of Confounding Measurement | The method and setting of confounding measurement are the same for all study participants. | Partial |  |
| Method used for missing data | Appropriate methods are used if imputation is used for missing confounder data. | No |  |
| Appropriate Accounting for Confounding | Important potential confounders are accounted for in the study design (e.g., matching for key variables, stratification, or initial assembly of comparable groups). /Important potential confounders are accounted for in the analysis (i.e., appropriate adjustment). | No |  |
| **Study Confounding Summary** | Important potential confounders are appropriately accounted for, limiting potential bias with respect to the relationship between PF and outcome . |  | High |
|  | | | |
| **6. Statistical Analysis and Reporting** | Goal: To judge the risk of bias related to the statistical analysis and presentation of results. |  |  |
| Presentation of analytical strategy | There is sufficient presentation of data to assess the adequacy of the analysis. | Partial |  |
| Model development strategy | The strategy for model building (i.e., inclusion of variables in the statistical model) is appropriate and is based on a conceptual framework or model./ The selected statistical model is adequate for the design of the study. | No |  |
| Reporting of results | There is no selective reporting of results. | Unsure |  |
| **Statistical Analysis and Presentation Summary** | The statistical analysis is appropriate for the design of the study, limiting potential for presentation of invalid or spurious results. |  | High |
|  | | | |
| Study title: | Clinical characteristics and outcome of clinically diagnosed viral encephalitis in southwest China | | |
| Study author/ data: | Zhao et al., 2015 |  |  |
| Study identifier: | 10.1007/s10072-015-2333-8 |  |  |
|  | | | |
| Biases | Issues to consider for judging overall rating of "Risk of bias" | Rating of reporting | Rating of "Risk of bias" |
| **1. Study Participation** | Goal: To judge the risk of selection bias (likelihood that relationship between PF and outcome is different for participants and eligible non-participants). |  |  |
| Source of target population | The source population or population of interest is adequately described for key characteristics (LIST). | Partial |  |
| Method used to identify population | The sampling frame and recruitment are adequately described, including methods to identify the sample sufficient to limit potential bias (number and type used, e.g., referral patterns in health care) | No |  |
| Recruitment period | Period of recruitment is adequately described | No |  |
| Place of recruitment | Place of recruitment (setting and geographic location) are adequately described | Yes |  |
| Inclusion and exclusion criteria | Inclusion and exclusion criteria are adequately described (e.g., including explicit diagnostic criteria or “zero time” description). | Partial |  |
| Adequate study participation | There is adequate participation in the study by eligible individuals | Yes |  |
| Baseline characteristics | The baseline study sample (i.e., individuals entering the study) is adequately described for key characteristics (LIST). | Partial |  |
| **Summary Study participation** | The study sample represents the population of interest on key characteristics, sufficient to limit potential bias of the observed relationship between PF and outcome. |  | Moderate |
|  | | | |
| **2. Study Attrition** | Goal: To judge the risk of attrition bias (likelihood that relationship between PF and outcome are different for completing and non-completing participants). |  |  |
| Proportion of baseline sample available for analysis | Response rate (i.e., proportion of study sample completing the study and providing outcome data) is adequate. | Yes |  |
| Attempts to collect information on participants who dropped out | Attempts to collect information on participants who dropped out of the study are described. | No |  |
| Reasons and potential impact of subjects lost to follow-up | Reasons for loss to follow-up are provided. | No |  |
| Outcome and prognostic factor information on those lost to follow-up | Participants lost to follow-up are adequately described for key characteristics (LIST)./ There are no important differences between key characteristics (LIST) and outcomes in participants who completed the study and those who did not. | No |  |
| **Study Attrition Summary** | Loss to follow-up (from baseline sample to study population analyzed) is not associated with key characteristics (i.e., the study data adequately represent the sample) sufficient to limit potential bias to the observed relationship between PF and outcome. |  | Moderate |
|  | | | |
| **3. Prognostic Factor Measurement** | Goal: To judge the risk of measurement bias related to how PF was measured (differential measurement of PF related to the level of outcome). |  |  |
| Definition of the PF | A clear definition or description of 'PF' is provided (e.g., including dose, level, duration of exposure, and clear specification of the method of measurement). | Partial |  |
| Valid and Reliable Measurement of PF | Method of PF measurement is adequately valid and reliable to limit misclassification bias (e.g., may include relevant outside sources of information on measurement properties, also characteristics, such as blind measurement and limited reliance on recall)./ Continuous variables are reported or appropriate cut-points (i.e., not data-dependent) are used. | Yes |  |
| Method and Setting of PF Measurement | The method and setting of measurement of PF is the same for all study participants. | Yes |  |
| Proportion of data on PF available for analysis | Adequate proportion of the study sample has complete data for PF variable. | Yes |  |
| Method used for missing data | Appropriate methods of imputation are used for missing 'PF' data. | No |  |
| **PF Measurement Summary** | PF is adequately measured in study participants to sufficiently limit potential bias. |  | Low |
|  | | | |
| 4. Outcome Measurement | Goal: To judge the risk of bias related to the measurement of outcome (differential measurement of outcome related to the baseline level of PF). |  |  |
| Definition of the Outcome | A clear definition of outcome is provided, including duration of follow-up and level and extent of the outcome construct. | Yes |  |
| Valid and Reliable Measurement of Outcome | The method of outcome measurement used is adequately valid and reliable to limit misclassification bias (e.g., may include relevant outside sources of information on measurement properties, also characteristics, such as blind measurement and confirmation of outcome with valid and reliable test). | Yes |  |
| Method and Setting of Outcome Measurement | The method and setting of outcome measurement is the same for all study participants. | Yes |  |
| **Outcome Measurement Summary** | Outcome of interest is adequately measured in study participants to sufficiently limit potential bias. |  | Low |
|  | | | |
| **5. Study Confounding** | Goal: To judge the risk of bias due to confounding (i.e. the effect of PF is distorted by another factor that is related to PF and outcome). |  |  |
| Important Confounders Measured | All important confounders, including treatments (key variables in conceptual model: LIST), are measured. | Partial |  |
| Definition of the confounding factor | Clear definitions of the important confounders measured are provided (e.g., including dose, level, and duration of exposures). | Partial |  |
| Valid and Reliable Measurement of Confounders | Measurement of all important confounders is adequately valid and reliable (e.g., may include relevant outside sources of information on measurement properties, also characteristics, such as blind measurement and limited reliance on recall). | Yes |  |
| Method and Setting of Confounding Measurement | The method and setting of confounding measurement are the same for all study participants. | Yes |  |
| Method used for missing data | Appropriate methods are used if imputation is used for missing confounder data. | No |  |
| Appropriate Accounting for Confounding | Important potential confounders are accounted for in the study design (e.g., matching for key variables, stratification, or initial assembly of comparable groups). /Important potential confounders are accounted for in the analysis (i.e., appropriate adjustment). | Partial |  |
| **Study Confounding Summary** | Important potential confounders are appropriately accounted for, limiting potential bias with respect to the relationship between PF and outcome . |  | Moderate |
|  | | | |
| **6. Statistical Analysis and Reporting** | Goal: To judge the risk of bias related to the statistical analysis and presentation of results. |  |  |
| Presentation of analytical strategy | There is sufficient presentation of data to assess the adequacy of the analysis. | Yes |  |
| Model development strategy | The strategy for model building (i.e., inclusion of variables in the statistical model) is appropriate and is based on a conceptual framework or model./ The selected statistical model is adequate for the design of the study. | Partial |  |
| Reporting of results | There is no selective reporting of results. | Unsure |  |
| **Statistical Analysis and Presentation Summary** | The statistical analysis is appropriate for the design of the study, limiting potential for presentation of invalid or spurious results. |  | Low |
|  | | | |
| Study title: | Predictors of outcome in HSV encephalitis | | |
| Study author/ data: | Singh et al., 2016 | | |
| Study identifier: | 10.1007/s00415-015-7960-8 | | |
|  | | | |
| Biases | Issues to consider for judging overall rating of "Risk of bias" | Rating of reporting | Rating of "Risk of bias" |
| **1. Study Participation** | Goal: To judge the risk of selection bias (likelihood that relationship between PF and outcome is different for participants and eligible non-participants). |  |  |
| Source of target population | The source population or population of interest is adequately described for key characteristics (LIST). | Yes |  |
| Method used to identify population | The sampling frame and recruitment are adequately described, including methods to identify the sample sufficient to limit potential bias (number and type used, e.g., referral patterns in health care) | Partial |  |
| Recruitment period | Period of recruitment is adequately described | Yes |  |
| Place of recruitment | Place of recruitment (setting and geographic location) are adequately described | Yes |  |
| Inclusion and exclusion criteria | Inclusion and exclusion criteria are adequately described (e.g., including explicit diagnostic criteria or “zero time” description). | Yes |  |
| Adequate study participation | There is adequate participation in the study by eligible individuals | Yes |  |
| Baseline characteristics | The baseline study sample (i.e., individuals entering the study) is adequately described for key characteristics (LIST). | Partial |  |
| **Summary Study participation** | The study sample represents the population of interest on key characteristics, sufficient to limit potential bias of the observed relationship between PF and outcome. |  | Low |
|  | | | |
| **2. Study Attrition** | Goal: To judge the risk of attrition bias (likelihood that relationship between PF and outcome are different for completing and non-completing participants). |  |  |
| Proportion of baseline sample available for analysis | Response rate (i.e., proportion of study sample completing the study and providing outcome data) is adequate. | Yes |  |
| Attempts to collect information on participants who dropped out | Attempts to collect information on participants who dropped out of the study are described. | Yes |  |
| Reasons and potential impact of subjects lost to follow-up | Reasons for loss to follow-up are provided. | No |  |
| Outcome and prognostic factor information on those lost to follow-up | Participants lost to follow-up are adequately described for key characteristics (LIST)./ There are no important differences between key characteristics (LIST) and outcomes in participants who completed the study and those who did not. | Partial |  |
| **Study Attrition Summary** | Loss to follow-up (from baseline sample to study population analyzed) is not associated with key characteristics (i.e., the study data adequately represent the sample) sufficient to limit potential bias to the observed relationship between PF and outcome. |  | Low |
|  | | | |
| **3. Prognostic Factor Measurement** | Goal: To judge the risk of measurement bias related to how PF was measured (differential measurement of PF related to the level of outcome). |  |  |
| Definition of the PF | A clear definition or description of 'PF' is provided (e.g., including dose, level, duration of exposure, and clear specification of the method of measurement). | Yes |  |
| Valid and Reliable Measurement of PF | Method of PF measurement is adequately valid and reliable to limit misclassification bias (e.g., may include relevant outside sources of information on measurement properties, also characteristics, such as blind measurement and limited reliance on recall)./ Continuous variables are reported or appropriate cut-points (i.e., not data-dependent) are used. | Yes |  |
| Method and Setting of PF Measurement | The method and setting of measurement of PF is the same for all study participants. | Yes |  |
| Proportion of data on PF available for analysis | Adequate proportion of the study sample has complete data for PF variable. | Yes |  |
| Method used for missing data | Appropriate methods of imputation are used for missing 'PF' data. | No |  |
| **PF Measurement Summary** | PF is adequately measured in study participants to sufficiently limit potential bias. |  | Low |
|  | | | |
| 4. Outcome Measurement | Goal: To judge the risk of bias related to the measurement of outcome (differential measurement of outcome related to the baseline level of PF). |  |  |
| Definition of the Outcome | A clear definition of outcome is provided, including duration of follow-up and level and extent of the outcome construct. | Yes |  |
| Valid and Reliable Measurement of Outcome | The method of outcome measurement used is adequately valid and reliable to limit misclassification bias (e.g., may include relevant outside sources of information on measurement properties, also characteristics, such as blind measurement and confirmation of outcome with valid and reliable test). | Yes |  |
| Method and Setting of Outcome Measurement | The method and setting of outcome measurement is the same for all study participants. | Yes |  |
| **Outcome Measurement Summary** | Outcome of interest is adequately measured in study participants to sufficiently limit potential bias. |  | Low |
|  | | | |
| **5. Study Confounding** | Goal: To judge the risk of bias due to confounding (i.e. the effect of PF is distorted by another factor that is related to PF and outcome). |  |  |
| Important Confounders Measured | All important confounders, including treatments (key variables in conceptual model: LIST), are measured. | Partial |  |
| Definition of the confounding factor | Clear definitions of the important confounders measured are provided (e.g., including dose, level, and duration of exposures). | Yes |  |
| Valid and Reliable Measurement of Confounders | Measurement of all important confounders is adequately valid and reliable (e.g., may include relevant outside sources of information on measurement properties, also characteristics, such as blind measurement and limited reliance on recall). | Partial |  |
| Method and Setting of Confounding Measurement | The method and setting of confounding measurement are the same for all study participants. | Yes |  |
| Method used for missing data | Appropriate methods are used if imputation is used for missing confounder data. | No |  |
| Appropriate Accounting for Confounding | Important potential confounders are accounted for in the study design (e.g., matching for key variables, stratification, or initial assembly of comparable groups). /Important potential confounders are accounted for in the analysis (i.e., appropriate adjustment). | Partial |  |
| **Study Confounding Summary** | Important potential confounders are appropriately accounted for, limiting potential bias with respect to the relationship between PF and outcome . |  | Low |
|  | | | |
| **6. Statistical Analysis and Reporting** | Goal: To judge the risk of bias related to the statistical analysis and presentation of results. |  |  |
| Presentation of analytical strategy | There is sufficient presentation of data to assess the adequacy of the analysis. | Yes |  |
| Model development strategy | The strategy for model building (i.e., inclusion of variables in the statistical model) is appropriate and is based on a conceptual framework or model./ The selected statistical model is adequate for the design of the study. | Yes |  |
| Reporting of results | There is no selective reporting of results. | Yes |  |
| **Statistical Analysis and Presentation Summary** | The statistical analysis is appropriate for the design of the study, limiting potential for presentation of invalid or spurious results. |  | Low |
|  | | | |
| Study title: | The Epidemiology, Clinical Features, and Long-Term Prognosis of Japanese Encephalitis in Central Sarawak, Malaysia, 1997–2005 | | |
| Study author/ data: | Ooi et al., 2008 | | |
| Study identifier: | 10.1086/590008 | | |
|  | | | |
| Biases | Issues to consider for judging overall rating of "Risk of bias" | Rating of reporting | Rating of "Risk of bias" |
| **1. Study Participation** | Goal: To judge the risk of selection bias (likelihood that relationship between PF and outcome is different for participants and eligible non-participants). |  |  |
| Source of target population | The source population or population of interest is adequately described for key characteristics (LIST). | Partial |  |
| Method used to identify population | The sampling frame and recruitment are adequately described, including methods to identify the sample sufficient to limit potential bias (number and type used, e.g., referral patterns in health care) | Partial |  |
| Recruitment period | Period of recruitment is adequately described | Yes |  |
| Place of recruitment | Place of recruitment (setting and geographic location) are adequately described | Yes |  |
| Inclusion and exclusion criteria | Inclusion and exclusion criteria are adequately described (e.g., including explicit diagnostic criteria or “zero time” description). | Partial |  |
| Adequate study participation | There is adequate participation in the study by eligible individuals | Yes |  |
| Baseline characteristics | The baseline study sample (i.e., individuals entering the study) is adequately described for key characteristics (LIST). | Partial |  |
| **Summary Study participation** | The study sample represents the population of interest on key characteristics, sufficient to limit potential bias of the observed relationship between PF and outcome. |  | Moderate |
|  | | | |
| **2. Study Attrition** | Goal: To judge the risk of attrition bias (likelihood that relationship between PF and outcome are different for completing and non-completing participants). |  |  |
| Proportion of baseline sample available for analysis | Response rate (i.e., proportion of study sample completing the study and providing outcome data) is adequate. | Yes |  |
| Attempts to collect information on participants who dropped out | Attempts to collect information on participants who dropped out of the study are described. | No |  |
| Reasons and potential impact of subjects lost to follow-up | Reasons for loss to follow-up are provided. | No |  |
| Outcome and prognostic factor information on those lost to follow-up | Participants lost to follow-up are adequately described for key characteristics (LIST)./ There are no important differences between key characteristics (LIST) and outcomes in participants who completed the study and those who did not. | No |  |
| **Study Attrition Summary** | Loss to follow-up (from baseline sample to study population analyzed) is not associated with key characteristics (i.e., the study data adequately represent the sample) sufficient to limit potential bias to the observed relationship between PF and outcome. |  | Low |
|  | | | |
| **3. Prognostic Factor Measurement** | Goal: To judge the risk of measurement bias related to how PF was measured (differential measurement of PF related to the level of outcome). |  |  |
| Definition of the PF | A clear definition or description of 'PF' is provided (e.g., including dose, level, duration of exposure, and clear specification of the method of measurement). | Partial |  |
| Valid and Reliable Measurement of PF | Method of PF measurement is adequately valid and reliable to limit misclassification bias (e.g., may include relevant outside sources of information on measurement properties, also characteristics, such as blind measurement and limited reliance on recall)./ Continuous variables are reported or appropriate cut-points (i.e., not data-dependent) are used. | Partial |  |
| Method and Setting of PF Measurement | The method and setting of measurement of PF is the same for all study participants. | Yes |  |
| Proportion of data on PF available for analysis | Adequate proportion of the study sample has complete data for PF variable. | Yes |  |
| Method used for missing data | Appropriate methods of imputation are used for missing 'PF' data. | No |  |
| **PF Measurement Summary** | PF is adequately measured in study participants to sufficiently limit potential bias. |  | Moderate |
|  | | | |
| 4. Outcome Measurement | Goal: To judge the risk of bias related to the measurement of outcome (differential measurement of outcome related to the baseline level of PF). |  |  |
| Definition of the Outcome | A clear definition of outcome is provided, including duration of follow-up and level and extent of the outcome construct. | Yes |  |
| Valid and Reliable Measurement of Outcome | The method of outcome measurement used is adequately valid and reliable to limit misclassification bias (e.g., may include relevant outside sources of information on measurement properties, also characteristics, such as blind measurement and confirmation of outcome with valid and reliable test). | Yes |  |
| Method and Setting of Outcome Measurement | The method and setting of outcome measurement is the same for all study participants. | Yes |  |
| **Outcome Measurement Summary** | Outcome of interest is adequately measured in study participants to sufficiently limit potential bias. |  | Low |
|  | | | |
| **5. Study Confounding** | Goal: To judge the risk of bias due to confounding (i.e. the effect of PF is distorted by another factor that is related to PF and outcome). |  |  |
| Important Confounders Measured | All important confounders, including treatments (key variables in conceptual model: LIST), are measured. | Partial |  |
| Definition of the confounding factor | Clear definitions of the important confounders measured are provided (e.g., including dose, level, and duration of exposures). | Partial |  |
| Valid and Reliable Measurement of Confounders | Measurement of all important confounders is adequately valid and reliable (e.g., may include relevant outside sources of information on measurement properties, also characteristics, such as blind measurement and limited reliance on recall). | Partial |  |
| Method and Setting of Confounding Measurement | The method and setting of confounding measurement are the same for all study participants. | Yes |  |
| Method used for missing data | Appropriate methods are used if imputation is used for missing confounder data. | Unsure |  |
| Appropriate Accounting for Confounding | Important potential confounders are accounted for in the study design (e.g., matching for key variables, stratification, or initial assembly of comparable groups). /Important potential confounders are accounted for in the analysis (i.e., appropriate adjustment). | Yes |  |
| **Study Confounding Summary** | Important potential confounders are appropriately accounted for, limiting potential bias with respect to the relationship between PF and outcome . |  | Low |
|  | | | |
| **6. Statistical Analysis and Reporting** | Goal: To judge the risk of bias related to the statistical analysis and presentation of results. |  |  |
| Presentation of analytical strategy | There is sufficient presentation of data to assess the adequacy of the analysis. | Yes |  |
| Model development strategy | The strategy for model building (i.e., inclusion of variables in the statistical model) is appropriate and is based on a conceptual framework or model./ The selected statistical model is adequate for the design of the study. | Yes |  |
| Reporting of results | There is no selective reporting of results. | Yes |  |
| **Statistical Analysis and Presentation Summary** | The statistical analysis is appropriate for the design of the study, limiting potential for presentation of invalid or spurious results. |  | Low |
|  | | | |
| Study title: | Herpes simplex virus encephalitis: Clinical manifestations, diagnosis and outcome in 106 adult patients | | |
| Study author/ data: | Sili et al., 2014 |  |  |
| Study identifier: | 10.1016/j.jcv.2014.03.010 |  |  |
|  | | | |
| Biases | Issues to consider for judging overall rating of "Risk of bias" | Rating of reporting | Rating of "Risk of bias" |
| **1. Study Participation** | Goal: To judge the risk of selection bias (likelihood that relationship between PF and outcome is different for participants and eligible non-participants). |  |  |
| Source of target population | The source population or population of interest is adequately described for key characteristics (LIST). | Partial |  |
| Method used to identify population | The sampling frame and recruitment are adequately described, including methods to identify the sample sufficient to limit potential bias (number and type used, e.g., referral patterns in health care) | Partial |  |
| Recruitment period | Period of recruitment is adequately described | Yes |  |
| Place of recruitment | Place of recruitment (setting and geographic location) are adequately described | Yes |  |
| Inclusion and exclusion criteria | Inclusion and exclusion criteria are adequately described (e.g., including explicit diagnostic criteria or “zero time” description). | Yes |  |
| Adequate study participation | There is adequate participation in the study by eligible individuals | Yes |  |
| Baseline characteristics | The baseline study sample (i.e., individuals entering the study) is adequately described for key characteristics (LIST). | Partial |  |
| **Summary Study participation** | The study sample represents the population of interest on key characteristics, sufficient to limit potential bias of the observed relationship between PF and outcome. |  | Low |
|  | | | |
| **2. Study Attrition** | Goal: To judge the risk of attrition bias (likelihood that relationship between PF and outcome are different for completing and non-completing participants). |  |  |
| Proportion of baseline sample available for analysis | Response rate (i.e., proportion of study sample completing the study and providing outcome data) is adequate. | Yes |  |
| Attempts to collect information on participants who dropped out | Attempts to collect information on participants who dropped out of the study are described. | No |  |
| Reasons and potential impact of subjects lost to follow-up | Reasons for loss to follow-up are provided. | Partial |  |
| Outcome and prognostic factor information on those lost to follow-up | Participants lost to follow-up are adequately described for key characteristics (LIST)./ There are no important differences between key characteristics (LIST) and outcomes in participants who completed the study and those who did not. | Partial |  |
| **Study Attrition Summary** | Loss to follow-up (from baseline sample to study population analyzed) is not associated with key characteristics (i.e., the study data adequately represent the sample) sufficient to limit potential bias to the observed relationship between PF and outcome. |  | Low |
|  | | | |
| **3. Prognostic Factor Measurement** | Goal: To judge the risk of measurement bias related to how PF was measured (differential measurement of PF related to the level of outcome). |  |  |
| Definition of the PF | A clear definition or description of 'PF' is provided (e.g., including dose, level, duration of exposure, and clear specification of the method of measurement). | Yes |  |
| Valid and Reliable Measurement of PF | Method of PF measurement is adequately valid and reliable to limit misclassification bias (e.g., may include relevant outside sources of information on measurement properties, also characteristics, such as blind measurement and limited reliance on recall)./ Continuous variables are reported or appropriate cut-points (i.e., not data-dependent) are used. | Yes |  |
| Method and Setting of PF Measurement | The method and setting of measurement of PF is the same for all study participants. | Yes |  |
| Proportion of data on PF available for analysis | Adequate proportion of the study sample has complete data for PF variable. | Yes |  |
| Method used for missing data | Appropriate methods of imputation are used for missing 'PF' data. | No |  |
| **PF Measurement Summary** | PF is adequately measured in study participants to sufficiently limit potential bias. |  | Low |
|  | | | |
| 4. Outcome Measurement | Goal: To judge the risk of bias related to the measurement of outcome (differential measurement of outcome related to the baseline level of PF). |  |  |
| Definition of the Outcome | A clear definition of outcome is provided, including duration of follow-up and level and extent of the outcome construct. | Yes |  |
| Valid and Reliable Measurement of Outcome | The method of outcome measurement used is adequately valid and reliable to limit misclassification bias (e.g., may include relevant outside sources of information on measurement properties, also characteristics, such as blind measurement and confirmation of outcome with valid and reliable test). | Partial |  |
| Method and Setting of Outcome Measurement | The method and setting of outcome measurement is the same for all study participants. | Partial |  |
| **Outcome Measurement Summary** | Outcome of interest is adequately measured in study participants to sufficiently limit potential bias. |  | Moderate |
|  | | | |
| **5. Study Confounding** | Goal: To judge the risk of bias due to confounding (i.e. the effect of PF is distorted by another factor that is related to PF and outcome). |  |  |
| Important Confounders Measured | All important confounders, including treatments (key variables in conceptual model: LIST), are measured. | Yes |  |
| Definition of the confounding factor | Clear definitions of the important confounders measured are provided (e.g., including dose, level, and duration of exposures). | Yes |  |
| Valid and Reliable Measurement of Confounders | Measurement of all important confounders is adequately valid and reliable (e.g., may include relevant outside sources of information on measurement properties, also characteristics, such as blind measurement and limited reliance on recall). | Yes |  |
| Method and Setting of Confounding Measurement | The method and setting of confounding measurement are the same for all study participants. | Yes |  |
| Method used for missing data | Appropriate methods are used if imputation is used for missing confounder data. | No |  |
| Appropriate Accounting for Confounding | Important potential confounders are accounted for in the study design (e.g., matching for key variables, stratification, or initial assembly of comparable groups). /Important potential confounders are accounted for in the analysis (i.e., appropriate adjustment). | Yes |  |
| **Study Confounding Summary** | Important potential confounders are appropriately accounted for, limiting potential bias with respect to the relationship between PF and outcome . |  | Low |
|  | | | |
| **6. Statistical Analysis and Reporting** | Goal: To judge the risk of bias related to the statistical analysis and presentation of results. |  |  |
| Presentation of analytical strategy | There is sufficient presentation of data to assess the adequacy of the analysis. | Yes |  |
| Model development strategy | The strategy for model building (i.e., inclusion of variables in the statistical model) is appropriate and is based on a conceptual framework or model./ The selected statistical model is adequate for the design of the study. | Yes |  |
| Reporting of results | There is no selective reporting of results. | Yes |  |
| **Statistical Analysis and Presentation Summary** | The statistical analysis is appropriate for the design of the study, limiting potential for presentation of invalid or spurious results. |  | Low |
|  | | | |
| Study title: | Clinical characteristics and outcome of human herpesvirus-6 encephalitis after allogeneic hematopoietic stem cell transplantation | | |
| Study author/ data: | Ogata et al., 2017 | | |
| Study identifier: | 10.1038/bmt.2017.175 | | |
|  | | | |
| Biases | Issues to consider for judging overall rating of "Risk of bias" | Rating of reporting | Rating of "Risk of bias" |
| **1. Study Participation** | Goal: To judge the risk of selection bias (likelihood that relationship between PF and outcome is different for participants and eligible non-participants). |  |  |
| Source of target population | The source population or population of interest is adequately described for key characteristics (LIST). | Partial |  |
| Method used to identify population | The sampling frame and recruitment are adequately described, including methods to identify the sample sufficient to limit potential bias (number and type used, e.g., referral patterns in health care) | Partial |  |
| Recruitment period | Period of recruitment is adequately described | Yes |  |
| Place of recruitment | Place of recruitment (setting and geographic location) are adequately described | Yes |  |
| Inclusion and exclusion criteria | Inclusion and exclusion criteria are adequately described (e.g., including explicit diagnostic criteria or “zero time” description). | Yes |  |
| Adequate study participation | There is adequate participation in the study by eligible individuals | Yes |  |
| Baseline characteristics | The baseline study sample (i.e., individuals entering the study) is adequately described for key characteristics (LIST). | Yes |  |
| **Summary Study participation** | The study sample represents the population of interest on key characteristics, sufficient to limit potential bias of the observed relationship between PF and outcome. |  | Low |
|  | | | |
| **2. Study Attrition** | Goal: To judge the risk of attrition bias (likelihood that relationship between PF and outcome are different for completing and non-completing participants). |  |  |
| Proportion of baseline sample available for analysis | Response rate (i.e., proportion of study sample completing the study and providing outcome data) is adequate. | Partial |  |
| Attempts to collect information on participants who dropped out | Attempts to collect information on participants who dropped out of the study are described. | No |  |
| Reasons and potential impact of subjects lost to follow-up | Reasons for loss to follow-up are provided. | No |  |
| Outcome and prognostic factor information on those lost to follow-up | Participants lost to follow-up are adequately described for key characteristics (LIST)./ There are no important differences between key characteristics (LIST) and outcomes in participants who completed the study and those who did not. | No |  |
| **Study Attrition Summary** | Loss to follow-up (from baseline sample to study population analyzed) is not associated with key characteristics (i.e., the study data adequately represent the sample) sufficient to limit potential bias to the observed relationship between PF and outcome. |  | Moderate |
|  | | | |
| **3. Prognostic Factor Measurement** | Goal: To judge the risk of measurement bias related to how PF was measured (differential measurement of PF related to the level of outcome). |  |  |
| Definition of the PF | A clear definition or description of 'PF' is provided (e.g., including dose, level, duration of exposure, and clear specification of the method of measurement). | Yes |  |
| Valid and Reliable Measurement of PF | Method of PF measurement is adequately valid and reliable to limit misclassification bias (e.g., may include relevant outside sources of information on measurement properties, also characteristics, such as blind measurement and limited reliance on recall)./ Continuous variables are reported or appropriate cut-points (i.e., not data-dependent) are used. | Yes |  |
| Method and Setting of PF Measurement | The method and setting of measurement of PF is the same for all study participants. | Yes |  |
| Proportion of data on PF available for analysis | Adequate proportion of the study sample has complete data for PF variable. | Yes |  |
| Method used for missing data | Appropriate methods of imputation are used for missing 'PF' data. | No |  |
| **PF Measurement Summary** | PF is adequately measured in study participants to sufficiently limit potential bias. |  | Low |
|  | | | |
| 4. Outcome Measurement | Goal: To judge the risk of bias related to the measurement of outcome (differential measurement of outcome related to the baseline level of PF). |  |  |
| Definition of the Outcome | A clear definition of outcome is provided, including duration of follow-up and level and extent of the outcome construct. | Yes |  |
| Valid and Reliable Measurement of Outcome | The method of outcome measurement used is adequately valid and reliable to limit misclassification bias (e.g., may include relevant outside sources of information on measurement properties, also characteristics, such as blind measurement and confirmation of outcome with valid and reliable test). | Yes |  |
| Method and Setting of Outcome Measurement | The method and setting of outcome measurement is the same for all study participants. | Yes |  |
| **Outcome Measurement Summary** | Outcome of interest is adequately measured in study participants to sufficiently limit potential bias. |  | Low |
|  | | | |
| **5. Study Confounding** | Goal: To judge the risk of bias due to confounding (i.e. the effect of PF is distorted by another factor that is related to PF and outcome). |  |  |
| Important Confounders Measured | All important confounders, including treatments (key variables in conceptual model: LIST), are measured. | Yes |  |
| Definition of the confounding factor | Clear definitions of the important confounders measured are provided (e.g., including dose, level, and duration of exposures). | Yes |  |
| Valid and Reliable Measurement of Confounders | Measurement of all important confounders is adequately valid and reliable (e.g., may include relevant outside sources of information on measurement properties, also characteristics, such as blind measurement and limited reliance on recall). | Yes |  |
| Method and Setting of Confounding Measurement | The method and setting of confounding measurement are the same for all study participants. | Yes |  |
| Method used for missing data | Appropriate methods are used if imputation is used for missing confounder data. | No |  |
| Appropriate Accounting for Confounding | Important potential confounders are accounted for in the study design (e.g., matching for key variables, stratification, or initial assembly of comparable groups). /Important potential confounders are accounted for in the analysis (i.e., appropriate adjustment). | Yes |  |
| **Study Confounding Summary** | Important potential confounders are appropriately accounted for, limiting potential bias with respect to the relationship between PF and outcome . |  | Low |
|  | | | |
| **6. Statistical Analysis and Reporting** | Goal: To judge the risk of bias related to the statistical analysis and presentation of results. |  |  |
| Presentation of analytical strategy | There is sufficient presentation of data to assess the adequacy of the analysis. | Yes |  |
| Model development strategy | The strategy for model building (i.e., inclusion of variables in the statistical model) is appropriate and is based on a conceptual framework or model./ The selected statistical model is adequate for the design of the study. | Yes |  |
| Reporting of results | There is no selective reporting of results. | Yes |  |
| **Statistical Analysis and Presentation Summary** | The statistical analysis is appropriate for the design of the study, limiting potential for presentation of invalid or spurious results. |  | Low |
