# Supplementary material for: Clinical and Laboratory Predictors of Poor Neurological Outcomes Following Infectious Encephalitis: Systematic Review and Meta‐Analysis
Source: Eur J Neurol. 2025 Nov 26;32(12):e70445. doi: 10.1111/ene.70445 (PMC12649060; doi:10.1111/ene.70445)
Supplement: Supplementary file 3 — File S3: ene70445‐sup‐0003‐FileS3.docx. [file ENE-32-e70445-s001.docx]

**Supplementary File 3 – Exposure definitions**

**Serum analysis**^1^

Serum thrombocytopaenia:

< 150x10^3^/uL

**CSF analysis**^2^

CSF hypoglycorachia:

< 45mg/dL

CSF elevated protein count:

> 45mg/dL

CSF white cell count OR pleocytosis OR leukocytosis:

> 5 cells/mm^3^

**Investigations**

Osmotherapy: any intervention involving administration of mannitol, glycerol, hypertonic saline, or any combination of the above.

References

1. Balduini CL, Noris P. Platelet count and aging. Haematologica. 2014 Jun 1;99(6):953–5.

2. Welch H, Hasbun R. Lumbar puncture and cerebrospinal fluid analysis. In 2010. p. 31–49.
