# Supplementary material for: Clinical and Laboratory Predictors of Poor Neurological Outcomes Following Infectious Encephalitis: Systematic Review and Meta‐Analysis
Source: Eur J Neurol. 2025 Nov 26;32(12):e70445. doi: 10.1111/ene.70445 (PMC12649060; doi:10.1111/ene.70445)
Supplement: Supplementary file 4 — File S4: ene70445‐sup‐0004‐FileS4.docx. [file ENE-32-e70445-s003.docx]

**Supplementary File 4 –** **Inter-rater variability analysis of neurological disability scales**

**Task: Using the different neurological disability scales (Table 1), please grade the clinical outcome of the infectious encephalitis patients specified in each clinical scenario.**

Table 1

| mRS | GOS | LOS | Alternative* |
| --- | --- | --- | --- |
| Grade 0 =  No symptoms at all | Grade 5 =  Fully recovery or minor disability with resumption of normal life | Grade 5 =  Full recovery | Grade 5 =  Full recovery |
| Grade 1 =  No significant disability despite symptoms; able to carry out all usual duties and activities | Grade 4 = Independent with ADLs at home but may require some assistance outside of the home | Grade 4 =  Minor sequelae with no effect, or only minor effects, on physical function; or personality change; or on medication. | Grade 4 =  Minor sequelae including altered personality or clinical signs not affecting function |
| Grade 2 =  Slight disability; unable to carry out all previous activities, but able to look after own affairs without assistance |  | Grade 3 =  Moderate sequelae mildly affecting function, probably compatible with independent living | Grade 3 =  Moderate sequelae, affecting function, but compatible with independence |
| Grade 3 =  Moderate disability; requiring some help, but able to walk without assistance | Grade 3 =  Severe disability with permanent need for assistance with activities of daily living (ADLs) |  |  |
| Grade 4 = Moderately severe disability; unable to walk and attend to bodily needs without assistance |  | Grade 2 =  Severe sequelae, impairing function sufficient to make patient dependent | Grade 2 =  Severe sequelae, incompatible with independent living |
| Grade 5 =  Severe disability; bedridden, incontinent and requiring constant nursing care and attention | Grade 2 =  Prolonged state of unresponsiveness with absence of awareness of self or environment |  |  |
| Grade 6 =  Death | Grade 1 =  Death | Grade 1 =  Death | Grade 1 =  Death |

*Scale derived from neurological disability criteria described by Whitley et al (1986). This scale represents the origin of the majority of non-traditional neurological disability scales used by our included studies. The majority of these studies classify “death+/-residual neurological disability” as a poor outcome, which translates approximately into mRS grades 3-6, GOS grades 1-3, and LOS grades 1-3.

**Patient A**: A 52-year-old woman, previously healthy, developed viral encephalitis 3 months ago. She has mild forgetfulness and occasional fatigue but can perform all daily activities independently. Her MRI shows no significant abnormalities, and she has returned to work as a teacher. She takes no medications for residual symptoms.

**Patient B**: A 34-year-old man suffered severe encephalitis due to herpes simplex virus. He is now bedridden and dependent on 24-hour nursing care. He is non-verbal, unresponsive to stimuli, and on a feeding tube. EEG shows diffuse slowing, consistent with severe encephalopathy.

**Patient C**: A 45-year-old woman developed limb weakness following an episode of presumed encephalitis. She uses a walker for short distances and struggles with dressing and bathing but can eat independently. Neuropsychological testing reveals moderate deficits in executive function. She attends outpatient physical and occupational therapy twice weekly.

**Patient D**: A 60-year-old man had bacterial encephalitis 6 months ago and now experiences mild personality changes and occasional headaches. He works full-time but has minor difficulty with multitasking. MRI shows subtle atrophy in the frontal lobes, and he is on a low-dose mood stabilizer.

**Patient E**: A 29-year-old woman had severe encephalitis caused by Japanese encephalitis virus. She remains wheelchair-bound with spastic quadriparesis and severe cognitive deficits. She requires assistance with all daily living activities and lives in a long-term care facility. Brain imaging shows extensive basal ganglia damage.

**Patient F**: A 75-year-old man, previously independent, recovered from West Nile virus encephalitis. He has mild short-term memory impairment and reduced stamina, but he manages his finances and performs light housework without help. His family reports occasional confusion in the evenings (suspected sundowning).

**Patient G**: A 19-year-old college student had mild enteroviral encephalitis. She made a complete recovery and is back to her studies without any neurological or psychological sequelae. EEG and MRI were normal at her 3-month follow-up.

**Patient H**: A 67-year-old woman developed severe encephalitis of a presumed infectious aetiology. She is unable to walk, disoriented, and fully dependent for all activities of daily living. CSF analysis showed elevated inflammatory markers, and she is on immunosuppressive therapy.

**Patient I**: A 58-year-old man experienced arboviral encephalitis 8 months ago. He can walk short distances with a cane but has persistent left-sided weakness. Neurocognitive tests reveal mild aphasia and impaired problem-solving skills, limiting his ability to work.

**Patient J**: A 40-year-old woman had cryptococcal meningoencephalitis related to HIV. She has returned to work part-time but experiences moderate fatigue and memory lapses. She takes antiretroviral therapy and occasional acetaminophen for headaches but does not require physical assistance.

**Patient K**: A 50-year-old man developed encephalitis following a varicella-zoster infection. He has residual left-leg weakness, requiring a brace, but is otherwise independent. He uses public transportation and attends weekly physical therapy for gait training.

**Patient L**: A 72-year-old woman suffered from severe encephalitis and remains in a persistent vegetative state. She has no voluntary movements or response to commands. CT scans reveal diffuse cortical atrophy, and she is fed via a gastrostomy tube.

**Patient M**: A 25-year-old man had mild encephalitis caused by mumps. He experiences occasional tremors in his hands but works full-time as a mechanic and plays recreational sports. Neurological examination is unremarkable apart from a mild postural tremor.

**Patient N**: A 61-year-old woman suffered from JEV encephalitis and now struggles with significant fatigue and occasional confusion. She can perform light housework but needs help with shopping and meal preparation. Her MRI shows hippocampal atrophy, and she takes anti-epileptic medication for partial seizures.

**Patient O**: A 30-year-old man had Eastern equine encephalitis and is paralyzed below the waist. He lives independently in a wheelchair-accessible apartment, manages his finances, and works as a software engineer. His cognitive function is intact.

**Patient P**: A 48-year-old woman developed severe cognitive and behavioral issues after herpes simplex encephalitis. She experiences frequent mood swings, forgetfulness, and disorientation. She requires supervision for personal hygiene and medications and lives in an assisted living facility.

**Patient Q**: A 3-year-old girl was hospitalized for measles encephalitis. She now has significant developmental delays and requires assistance with all daily activities. Her speech is limited to single words, and she has spasticity in her legs, impairing her mobility. She undergoes physical and speech therapy regularly.

**Patient R**: A 9-year-old boy recovered from a mild case of viral encephalitis but now has difficulty concentrating in school and occasional emotional outbursts. His neuropsychological assessment shows mild deficits in attention and working memory. He receives additional academic support.

**Patient S**: A 55-year-old man had St. Louis encephalitis and now experiences occasional headaches and mood changes but is fully independent. He has resumed his hobbies, including gardening and cycling, and takes over-the-counter analgesics as needed.

**Patient T**: A 46-year-old woman had bacterial encephalitis with residual hemiparesis. She uses a walker for mobility and needs help with cooking and cleaning but is otherwise self-sufficient. She attends outpatient therapy and is optimistic about her recovery trajectory.

Results

Fleiss kappa analysis:

|  | Scale | Kappa |
| --- | --- | --- |
| 1 | mRS | 1.0000000 |
| 2 | GOS | 0.9330357 |
| 3 | LOS | 0.9294947 |
| 4 | Alternative | 0.9294947 |

Pearson R analysis:

|  | mRS | GOS | LOS | Alt |
| --- | --- | --- | --- | --- |
| mRS | 1.0000000 | 0.6955695 | 0.9656254 | 0.9656254 |
| GOS | 0.6955695 | 1.0000000 | 0.7375086 | 0.7375086 |
| LOS | 0.9656254 | 0.7375086 | 1.0000000 | 1.0000000 |
| Alt | 0.9656254 | 0.7375086 | 1.0000000 | 1.0000000 |
