# Supplementary material for: Clinical and Laboratory Predictors of Poor Neurological Outcomes Following Infectious Encephalitis: Systematic Review and Meta‐Analysis
Source: Eur J Neurol. 2025 Nov 26;32(12):e70445. doi: 10.1111/ene.70445 (PMC12649060; doi:10.1111/ene.70445)
Supplement: Supplementary file 5 — File S5: ene70445‐sup‐0005‐FileS5.docx. [file ENE-32-e70445-s002.docx]

**Supplementary File 5 – Subgroup and sensitivity analyses**

Mortality at discharge

*Paediatric subgroup analyses*

| **Exposures** | **Result** |
| --- | --- |
| Male | Pooled RR: 0.97 [95% CI: 0.69-1.35, p=0.86, n=5, I^2^ = 0% |
| Rural location | NA |
| Fever | Pooled RR: 2.06 [95% CI: 1.29-3.31, p=0.003, n=3, I^2^ = 0% |
| Focal neurological signs | Pooled RR: 2.36 [95% CI: 0.93-5.99, p=0.07, n=2, I^2^ = 20% |
| Seizure activity | Pooled RR: 2.06 [95% CI: 1.22-3.48, p=0.007, n=4, I^2^ = 0% |
| Serum thrombocytopaenia | NA |
| CSF leukocytosis | Pooled RR: 0.81 [95% CI: 0.49-1.35, p=0.42, n=6, I^2^ = 41% |
| CSF elevated protein | Pooled RR: 1.42 [95% CI: 0.56-3.57, p=0.46, n=3, I^2^ = 16% |
| EEG abnormalities | NA |
| Shock | NA |
| GCS<8 | Pooled RR: 3.48 [95% CI: 2.09-5.79, p=0.05, n=5, I^2^ = 58% |
| Intubation and ventilation | NA |

*JEV subgroup analyses*

| **Exposures** | **Result** |
| --- | --- |
| Male | NA |
| Rural location | NA |
| Fever | NA |
| Focal neurological signs | NA |
| Seizure activity | NA |
| Serum thrombocytopaenia | NA |
| CSF leukocytosis | NA |
| CSF elevated protein | Pooled RR: 3.60 [95% CI: 1.18-11.00, p=0.02, n=3, I^2^ = 66% |
| EEG abnormalities | NA |
| Shock | NA |
| GCS<8 | Pooled RR: 2.98 [95% CI: 2.44-3.64, p<0.00001, n=3, I^2^ = 0% |
| Intubation and ventilation | NA |

*Sensitivity analyses*

| **Exposures** | **Sensitivity analysis subtype** | | |
| --- | --- | --- | --- |
|  | **ICU-only cohorts excluded (n=1)** | **Immunocompromised cohorts excluded (n=0)** | **Anomalous direction of effect or effect size** |
| Male | NA | NA | NA |
| Rural location | NA | NA | NA |
| Fever | NA | NA | NA |
| Focal neurological signs | NA | NA | NA |
| Seizure activity | Pooled RR: 1.59 [95% CI: 0.96-2.63, p=0.07, n=11, I^2^ = 89% | NA | NA |
| Serum thrombocytopaenia | NA | NA | NA |
| CSF leukocytosis | NA | NA | NA |
| CSF elevated protein | NA | NA | Pooled RR: 3.27 [95% CI: 1.49-7.19], p = 0.003, n=5, I^2^ = 37% |
| EEG abnormalities | NA | NA | NA |
| Shock | NA | NA | NA |
| GCS<8 | NA | NA | NA |
| Intubation and ventilation | NA | NA | NA |

*Sensitivity analysis and comparison of outcomes of infectious encephalitis cohorts with and without serological or CSF-based evidence*

| **Exposures** | **Aetiological evidence of causative organism** | |
| --- | --- | --- |
|  | **With serological/ CSF-based evidence** | **Without serological-CSF based evidence** |
| Male | Pooled RR: 0.98 [95% CI: 0.68-1.41, p=0.91, n=4, I^2^ = 73% | **Pooled RR: 0.87 [95% CI: 0.77-0.99, p=0.03, n=6, I^2^ = 0%** |
| Rural location | NA (n=1) | NA (n=1) |
| Fever | Pooled RR: 0.96 [95% CI: 0.45-2.08, p=0.93, n=4, I^2^ = 0% | Pooled RR: 2.10 [95% CI: 1.29-3.43, p=0.003, n=2, I^2^ = 0% |
| Focal neurological signs | NA (n=0) | NA (n=3) |
| Seizure activity | Pooled RR: 1.36 [95% CI: 0.79-2.32, p=0.26, n=5, I^2^ = 63% | Pooled RR: 1.73 [95% CI: 0.81-3.71, p=0.16, n=7, I^2^ = 90% |
| Serum thrombocytopaenia | NA (n=1) | NA (n=1) |
| CSF leukocytosis | Pooled RR: 0.75 [95% CI: 0.38-1.46, p=0.39, n=3, I^2^ = 53% | Pooled RR: 0.94 [95% CI: 0.33-2.69, p=0.91, n=3, I^2^ = 49% |
| CSF elevated protein | Pooled RR: 1.42 [95% CI: 0.56-3.57, p=0.46, n=3, I^2^ = 16% | Pooled RR: 3.85 [95% CI: 1.03-14.32], p=0.04, n=3, I^2^ = 65% |
| EEG abnormalities | NA (n=1) | NA (n=1) |
| Shock | NA (n=0) | NA (n=2) |
| GCS<8 | NA (n=0) | NA (n=7) |
| Intubation and ventilation | NA (n=1) | NA (n=2) |

Poor neurological outcome at discharge

*Paediatric subgroup analyses*

| **Exposures** | **Result** |
| --- | --- |
| Male | Pooled RR: 0.89 [95% CI: 0.77-1.02, p=0.10, n=8, I^2^ = 0% |
| Rural location | Pooled RR: 1.81 [95% CI: 0.70-4.68, p=0.22, n=3, I^2^ = 36% |
| Fever | Pooled RR: 0.88 [95% CI: 0.66-1.18, p=0.41, n=4, I^2^ = 0% |
| Immunocompromised | NA |
| Focal neurological signs | Pooled RR: 1.52 [95% CI: 0.49-4.75, p=0.47, n=2, I^2^ = 94% |
| Seizure activity | Pooled RR: 1.38 [95% CI: 0.94-2.02, p=0.10, n=7, I^2^ = 75% |
| Status epilepticus | Pooled RR: 2.08 [95% CI: 0.93-14.67, p=0.08, n=3, I^2^ = 80% |
| Serum thrombocytopaenia | NA |
| CSF leukocytosis | Pooled RR: 0.82 [95% CI: 0.69-0.98, p=0.03, n=4, I^2^ = 0% |
| CSF elevated protein | Pooled RR: 1.34 [95% CI: 1.04-1.72, p=0.03, n=3, I^2^ = 0% |
| CSF hypoglycorachia | Pooled RR: 1.33 [95% CI: 0.96-1.82, p=0.08, n=2, I^2^ = 6% |
| EEG abnormalities | Pooled RR: 2.68 [95% CI: 1.02-7.03, p=0.04, n=2, I^2^ = 0% |
| CT abnormality | NA |
| MRI abnormality | NA |
| GCS<8 | Pooled RR: 2.48 [95% CI: 2.04-3.00, p<0.00001, n=6, I^2^ = 39% |
| Intubation and ventilation | Pooled RR: 4.24 [95% CI: 2.62-6.84, p<0.00001, n=3, I^2^ = 63% |
| Adjunctive steroid therapy | Pooled RR: 1.87 [95% CI: 1.12-3.11, p=0.02, n=3, I^2^ = 74% |
| Adjunctive osmotherapy | NA |

*JEV subgroup analyses*

| **Exposures** | **Result** |
| --- | --- |
| Male | Pooled RR: 0.86 [95% CI: 0.67-1.10], p=0.23, n=4, I^2^ = 0% |
| Rural location | Pooled RR: 2.23 [95% CI: 0.79-6.27, p=0.13, n=3, I^2^ =4 0% |
| Fever | Pooled RR: 2.31 [95% CI: 0.37-14.56, p=0.37, n=2, I^2^ = 0% |
| Immunocompromised | NA |
| Focal neurological signs | NA |
| Seizure activity | Pooled RR: 3.04 [95% CI: 1.16-7.95], p=0.02, n=3, I^2^ = 73% |
| Status epilepticus | NA |
| Serum thrombocytopaenia | NA |
| CSF leukocytosis | NA |
| CSF elevated protein | Pooled RR: 4.65 [95% CI: 1.31-16.44, p=0.02, n=2, I^2^ = 0% |
| CSF hypoglycorachia | NA |
| EEG abnormalities | NA |
| CT abnormality | NA |
| MRI abnormality | NA |
| GCS<8 | Pooled RR: 2.74 [95% CI: 2.04-3.70, p<0.00001, n=4, I^2^ = 0% |
| Intubation and ventilation | NA |
| Adjunctive steroid therapy | NA |
| Adjunctive osmotherapy | NA |

*HSV subgroup analyses*

| **Exposures** | **Result** |
| --- | --- |
| Male | Pooled RR: 0.81 [95% CI: 0.61-1.08, p=0.16, n=2, I^2^ = 0% |
| Rural location | NA |
| Fever | NA |
| Immunocompromised | NA |
| Focal neurological signs | NA |
| Seizure activity | NA |
| Status epilepticus | NA |
| Serum thrombocytopaenia | NA |
| CSF leukocytosis | NA |
| CSF elevated protein | NA |
| CSF hypoglycorachia | NA |
| EEG abnormalities | NA |
| CT abnormality | NA |
| MRI abnormality | NA |
| GCS<8 | NA |
| Intubation and ventilation | NA |
| Adjunctive steroid therapy | NA |
| Adjunctive osmotherapy | NA |

*Sensitivity analyses*

| **Exposures** | **Sensitivity analysis subtype** | | |
| --- | --- | --- | --- |
|  | **ICU-only cohorts excluded (n=1)** | **Immunocompromised cohorts excluded (n=0)** | **Anomalous direction of effect or effect size** |
| Male | NA | NA | NA |
| Rural location | NA | NA | NA |
| Fever | Pooled RR: 1.16 [95% CI: 0.89-1.50, p=0.27, n=8, I^2^ = 41% | NA | NA |
| Immunocompromised | NA | NA | NA |
| Focal neurological signs | NA | NA | NA |
| Seizure activity | Pooled RR: 1.53 [95% CI: 1.17-20.02, p=0.002, n=12, I^2^ = 78% | NA | NA |
| Status epilepticus | Pooled RR: 1.92 [95% CI: 0.94-3.90, p=0.07, n=4, I^2^ = 71% | NA | NA |
| Serum thrombocytopaenia | NA | NA | NA |
| CSF leukocytosis | NA | NA | NA |
| CSF elevated protein | NA | NA | NA |
| CSF hypoglycorachia | NA | NA | NA |
| EEG abnormalities | Pooled RR: 1.44 [95% CI: 1.08-1.91, p=0.01, n=5, I^2^ = 0% | NA | NA |
| CT abnormality | NA | NA | NA |
| MRI abnormality | NA | NA | NA |
| GCS<8 | Pooled RR: 2.38 [95% CI: 1.84-3.10, p<0.00001, n=11, I^2^ = 71% | NA | NA |
| Intubation and ventilation | Pooled RR: 3.63 [95% CI: 2.18-6.03, p<0.00001, n=6, I^2^ = 90% | NA | NA |
| Adjunctive steroid therapy | NA | NA | NA |
| Adjunctive osmotherapy | NA | NA | NA |

*Sensitivity analysis and comparison of outcomes of infectious encephalitis cohorts with and without serological or CSF-based evidence*

| **Exposures** | **Aetiological evidence of causative organism** | |
| --- | --- | --- |
|  | **With serological/ CSF-based evidence** | **Without serological-CSF based evidence** |
| Male | Pooled RR: 0.87 [95% CI: 0.74-1.02, p=0.08, n=8, I^2^ = 0% | Pooled RR: 0.78 [95% CI: 0.78-1.02, p=0.09, n=6, I^2^ = 0% |
| Rural location | NA (n=3) | NA (n=1) |
| Fever | Pooled RR: 1.15 [95% CI: 0.89-1.47, p=0.28, n=7, I^2^ = 47% | Pooled RR: 2.31 [95% CI: 0.37-14.56, p=0.37, n=2, I^2^ = 0% |
| Immunocompromised | Pooled RR: 3.42 [95% CI: 0.62-18.80, p=0.16, n=2, I^2^ = 87% | Pooled RR: 31.78 [95% CI: 0.44-7.10, p=0.42, n=2, I^2^ = 78% |
| Focal neurological signs | NA (n=1) | NA (n=5) |
| Seizure activity | Pooled RR: 2.50 [95% CI: 1.15-5.44, p=0.02, n=4, I^2^ = 65% | Pooled RR: 1.27 [95% CI: 0.96-1.69, p<0.00001, n=9, I^2^ = 80% |
| Status epilepticus | Pooled RR: 3.32 [95% CI: 0.56-19.65, p=0.19, n=2, I^2^ = 67% | Pooled RR: 1.54 [95% CI: 1.11-2.14, p=0.33, n=3, I^2^ = 10% |
| Serum thrombocytopaenia | NA (n=0) | NA (n=2) |
| CSF leukocytosis | NA (n=1) | NA (n=4) |
| CSF elevated protein | Pooled RR: 4.49 [95% CI: 1.45-13.96, p=0.009, n=3, I^2^ = 0% | Pooled RR: 1.22 [95% CI: 1.04-19.65, p=0.02, n=4, I^2^ = 0% |
| CSF hypoglycorachia | NA (n=0) | NA (n=3) |
| EEG abnormalities | NA (n=1) | NA (n=5) |
| CT abnormality | NA (n=0) | NA (n=2) |
| MRI abnormality | NA (n=0) | NA (n=2) |
| GCS<8 | Pooled RR: 2.54 [95% CI: 1.93-3.35, p<0.00001, n=6, I^2^ = 23% | Pooled RR: 2.33 [95% CI: 1.74-3.13, p<0.00001, n=6, I^2^ = 69% |
| Intubation and ventilation | Pooled RR: 2.40 [95% CI: 0.93-6.17, p=0.07, n=3, I^2^ = 88% | Pooled RR: 4.74 [95% CI: 3.07-7.31, p<0.00001, n=4, I^2^ = 80% |
| Adjunctive steroid therapy | NA (n=3) | NA (n=1) |
| Adjunctive osmotherapy | Pooled RR: 2.24 [95% CI: 1.47-3.41, p=0.0002, n=2, I^2^ = 0% | Pooled RR: 2.39 [95% CI: 2.00-2.87, p<0.00001, n=2, I^2^ = 0% |

*Sensitivity analysis and comparison of infectious encephalitis outcome measures*

| **Exposures** | **Infectious encephalitis outcome: neurological disability scales used** | |
| --- | --- | --- |
|  | **mRS or GOS** | **LOS or other** |
| Male | Pooled RR: 0.88 [95% CI: 0.78-1.00, p=0.05, n=11, I^2^ = 0% | Pooled RR: 0.88 [95% CI: 0.74-1.05, p=0.16, n=3, I^2^ = 0% |
| Seizure activity | Pooled RR: 1.40 [95% CI: 0.81-2.42, p=0.23, n=4, I^2^ = 87% | Pooled RR: 1.46 [95% CI: 1.05-2.02, p=0.02, n=9, I^2^ = 70% |
| GCS<8 | Pooled RR: 2.52 [95% CI: 1.38-4.59, p=0.003, n=4, I^2^ = 68% | Pooled RR: 2.61 [95% CI: 2.19-3.11, p<0.00001, n=8, I^2^ = 31% |

Poor neurological outcomes at >6 months

*Paediatric subgroup analyses*

| **Exposures** | **Result** |
| --- | --- |
| Male | Pooled RR: 0.94 [95% CI: 0.68-1.30, p=0.71, n=6, I^2^ = 0% |
| Rural location | NA |
| Fever | Pooled RR: 1.53 [95% CI: 0.75-3.12, p=0.24, n=4, I^2^ = 0% |
| Immunocompromised | NA |
| Focal neurological signs | NA |
| Seizure activity | Pooled RR: 4.58 [95% CI: 0.88-23.84, p=0.07, n=3, I^2^ = 91% |
| Status epilepticus | Pooled RR: 3.28 [95% CI: 1.01-10.63, p=0.05, n=3, I^2^ = 94% |
| EEG abnormalities | NA |
| CT abnormality | NA |
| MRI abnormality | NA |
| Shock | NA |
| GCS<8 | Pooled RR: 4.91 [95% CI: 2.95-8.17, p<0.00001, n=4, I^2^ = 0% |
| Intubation and ventilation | Pooled RR: 5.24 [95% CI: 0.58-47.67, p=0.14, n=2, I^2^ = 91% |
| Adjunctive steroid therapy | NA |

*JEV subgroup analyses*

| **Exposures** | **Result** |
| --- | --- |
| Male | Pooled RR: 1.06 [95% CI: 0.74-1.51, p=0.74, n=5, I^2^ = 13% |
| Rural location | Pooled RR: 1.06 [95% CI: 0.48-2.30, p=0.89, n=2, I^2^ = 0% |
| Fever | Pooled RR: 1.09 [95% CI: 0.72-1.65, p=0.70, n=4, I^2^ = 0% |
| Immunocompromised | NA |
| Focal neurological signs | Pooled RR: 0.76 [95% CI: 0.29-1.97, p=0.58, n=2, I^2^ = 55% |
| Seizure activity | Pooled RR: 3.10 [95% CI: 1.24-7.71], p < 0.00001, n=4, I^2^=89% |
| Status epilepticus | Pooled RR: 5.49 [95% CI: 3.32-9.08], p < 0.00001, n=2, I^2^ = 0% |
| Serum thrombocytopaenia | NA |
| EEG abnormalities | NA |
| CT abnormality | NA |
| MRI abnormality | NA |
| Shock | NA |
| GCS<8 | Pooled RR: 3.77 [95% CI: 2.30-6.18, p<0.00001, n=5, I^2^ = 62% |
| Intubation and ventilation | Pooled RR: 5.24 [95% CI: 0.58-47.67, p=0.14, n=2, I^2^ = 91% |
| Adjunctive steroid therapy | NA |

*HSV subgroup analyses*

| **Exposures** | **Result** |
| --- | --- |
| Male | Pooled RR: 0.87 [95% CI: 0.58-1.30, p=0.48, n=4, I^2^ = 0% |
| Rural location | NA |
| Fever | Pooled RR: 1.55 [95% CI: 0.72-3.33, p=0.27, n=2, I^2^ = 0% |
| Immunocompromised | NA |
| Focal neurological signs | Pooled RR: 1.00 [95% CI: 0.66-1.52, p=1.00, n=3, I^2^ = 0% |
| Seizure activity | Pooled RR: 0.86 [95% CI: 0.61-1.22], p < 0.39, n=4, I^2^=0 |
| Status epilepticus | Pooled RR: 1.94 [95% CI: 1.18-3.19], p=0.009, n=6, I^2^=0% |
| Serum thrombocytopaenia | NA |
| EEG abnormalities | NA |
| CT abnormality | NA |
| MRI abnormality | NA |
| ICU admission | NA |
| Shock | NA |
| GCS<8 | Pooled RR: 3.16 [95% CI: 1.99-5.01, p<0.00001, n=3, I^2^ = 0% |
| Intubation and ventilation | Pooled RR: 1.47 [95% CI: 0.90-2.39, p=0.13, n=2, I^2^ = 0% |
| Adjunctive steroid therapy | NA |

*Sensitivity analyses*

| **Exposures** | **Sensitivity analysis subtype** | | |
| --- | --- | --- | --- |
|  | **ICU-only cohorts excluded (n=1)** | **Immunocompromised cohorts excluded (n=1)** | **Anomalous direction of effect or effect size** |
| Male | NA | NA | NA |
| Rural location | NA | NA | NA |
| Fever | NA | NA | NA |
| Immunocompromised | NA | NA | NA |
| Focal neurological signs | Pooled RR: 0.95 [95% CI: 0.69-1.32, p=0.77, n=6, I^2^ = 0% | NA | NA |
| Seizure activity | Pooled RR: 1.59 [95% CI: 1.07-2.38, p=0.02, n=11, I^2^ = 78% | Pooled RR: 1.52 [95% CI: 1.00-2.32, p=0.05, n=11, I^2^ = 79% | NA |
| Status epilepticus | Pooled RR: 2.58 [95% CI: 1.30-5.15, p=0.007, n=5, I^2^ = 88% | NA | NA |
| Serum thrombocytopaenia | NA | NA | NA |
| CSF leukocytosis | NA | NA | NA |
| CSF elevated protein | NA | NA | NA |
| CSF hypoglycorachia | NA | NA | NA |
| EEG abnormalities | NA | NA | NA |
| CT abnormality | NA | NA | NA |
| MRI abnormality | NA | NA | NA |
| ICU admission | NA | NA | NA |
| Shock | NA | NA | NA |
| GCS<8 | NA | NA | NA |
| Intubation and ventilation | Pooled RR: 3.89 [95% CI: 1.16-13.04, p=0.03, n=5, I^2^ = 94% | NA | Pooled RR: 1.68 [95% CI: 1.16-2.44, p=0.006, n=4, I^2^ = 0% |
| Adjunctive steroid therapy | Pooled RR: 1.50 [95% CI: 0.70-3.22, p=0.29, n=2, I^2^ = 0% | NA | NA |
| Adjunctive osmotherapy | NA | NA | NA |

*Sensitivity analysis and comparison of outcomes of infectious encephalitis cohorts with and without serological or CSF-based evidence*

| **Exposures** | **Aetiological evidence of causative organism** | |
| --- | --- | --- |
|  | **With serological/ CSF-based evidence** | **Without serological-CSF based evidence** |
| Male | Pooled RR: 1.08 [95% CI: 0.87-1.34, p=0.48, n=7, I^2^ = 0% | Pooled RR: 1.09 [95% CI: 0.85-1.41, p=0.50, n=7, I^2^ = 0% |
| Rural location | NA (n=3) | NA (n=0) |
| Fever | Pooled RR: 0.96 [95% CI: 0.59-1.56, p=0.87, n=4, I^2^ = 0% | Pooled RR: 1.35 [95% CI: 0.91-2.02, p=0.14, n=4, I^2^ = 0% |
| Immunocompromised | NA (n=2) | NA (n=0) |
| Focal neurological signs | Pooled RR: 0.76 [95% CI: 0.47-1.24, p=0.27, n=3, I^2^ = 0% | Pooled RR: 1.03 [95% CI: 0.66-1.60, p=0.90, n=4, I^2^ = 8% |
| Seizure activity | Pooled RR: 1.57 [95% CI: 0.81-3.04, p=0.18, n=8, I^2^ = 82% | Pooled RR: 1.59 [95% CI: 1.01-2.48, p=0.04, n=4, I^2^ = 65% |
| Status epilepticus | NA (n=6) | NA (n=0) |
| EEG abnormalities | NA (n=2) | NA (n=1) |
| CT abnormality | NA (n=0) | NA (n=2) |
| MRI abnormality | NA (n=1) | NA (n=2) |
| GCS<8 | Pooled RR: 4.62 [95% CI: 2.97-7.18, p<0.00001, n=5, I^2^ = 0% | Pooled RR: 2.70 [95% CI: 1.99-3.67, p<0.00001, n=4, I^2^ = 28% |
| Intubation and ventilation | NA (n=5) | NA (n=1) |
| Adjunctive steroid therapy | NA (n=2) | NA (n=1) |

*Sensitivity analysis and comparison of infectious encephalitis outcome measures*

| **Exposures** | **Infectious encephalitis outcome: neurological disability scales used** | |
| --- | --- | --- |
|  | **mRS or GOS** | **LOS or other** |
| Male | Pooled RR: 1.12 [95% CI: 0.86-1.45, p=0.41, n=7, I^2^ = 0% | Pooled RR: 1.04 [95% CI: 0.81-1.34, p=0.73, n=7, I^2^ = 11% |
| Seizure activity | Pooled RR: 1.08 [95% CI: 0.61-1.92, p=0.80, n=6, I^2^ = 70% | Pooled RR: 2.12 [95% CI: 1.17-3.86, p=0.01, n=6, I^2^ = 83% |
| GCS<8 | Pooled RR: 1.55 [95% CI: 0.73-3.30, p=0.25, n=2, I^2^ = 83% | Pooled RR: 6.90 [95% CI: 2.85-16.72, p<0.0001, n=3, I^2^ = 37% |
