# Supplementary material for: Clinical and Laboratory Predictors of Poor Neurological Outcomes Following Infectious Encephalitis: Systematic Review and Meta‐Analysis
Source: Eur J Neurol. 2025 Nov 26;32(12):e70445. doi: 10.1111/ene.70445 (PMC12649060; doi:10.1111/ene.70445)
Supplement: Supplementary file 6 — File S6: ene70445‐sup‐0006‐FileS6.docx. [file ENE-32-e70445-s004.docx]

**Supplementary File 6 – Publication bias analysis**

*Egger’s test**

| **Outcome** | **Exposure** | **Result** |
| --- | --- | --- |
| Discharge mortality | Biological sex (male) | t = 2.01, df = 9, p-value = 0.0752 |
|  | Seizure activity | t = 1.69, df = 10, p-value = 0.1228 |
| Poor outcome at discharge | Biological sex (male) | t = 1.15, df = 12, p-value = 0.2723 |
|  | Seizure activity | t = 0.35, df = 11, p-value = 0.7334 |
|  | GCS<8 | t = -0.28, df = 10, p-value = 0.7885 |
| Poor outcome at >6 months | Biological sex (male) | t = -0.69, df = 12, p-value = 0.5014 |
|  | Seizure activity | t = -0.00, df = 10, p-value = 0.9968 |

*Egger’s test was only conducted for exposures per outcome with equal to or more than 10 data points.

*Funnel plots*

Discharge mortality


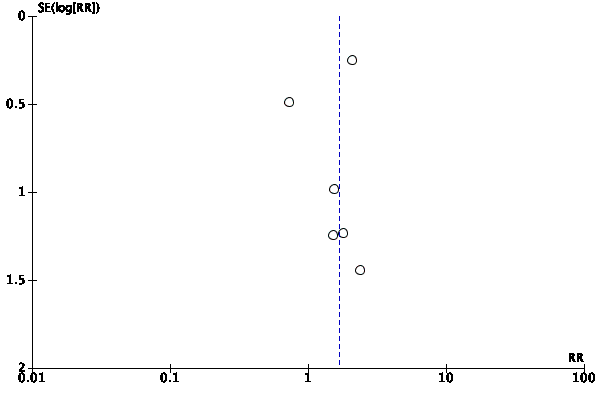

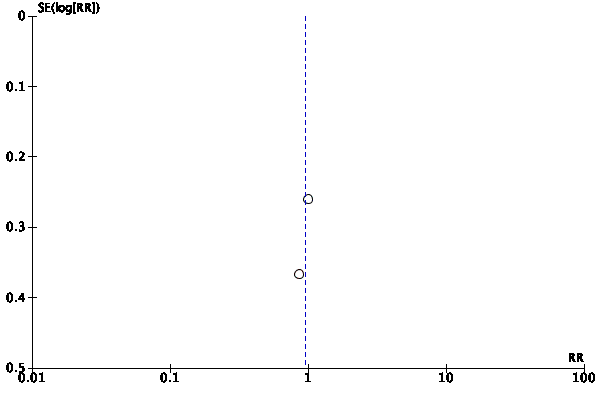

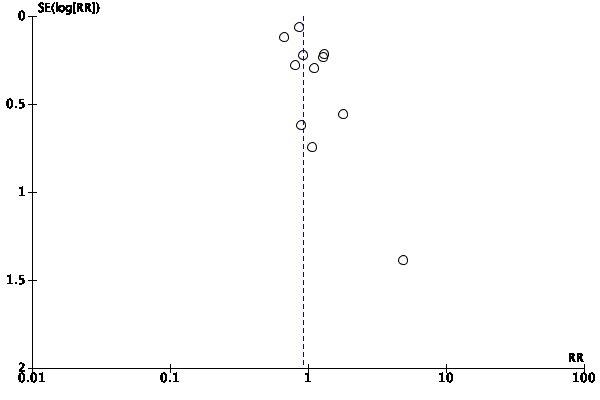


Rural (vs urban) location

Fever

Biological sex (male)


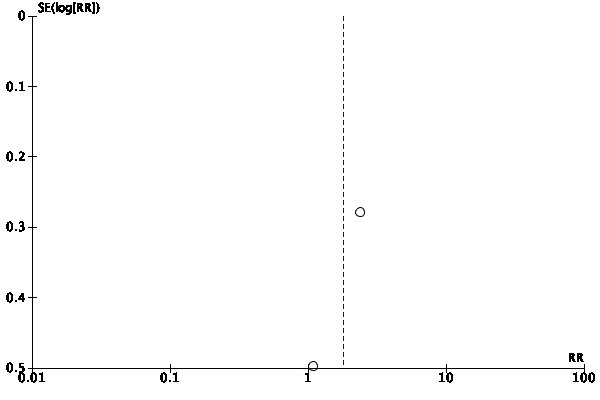

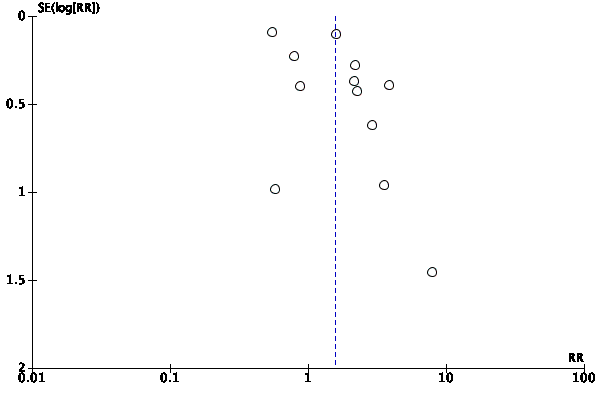


*
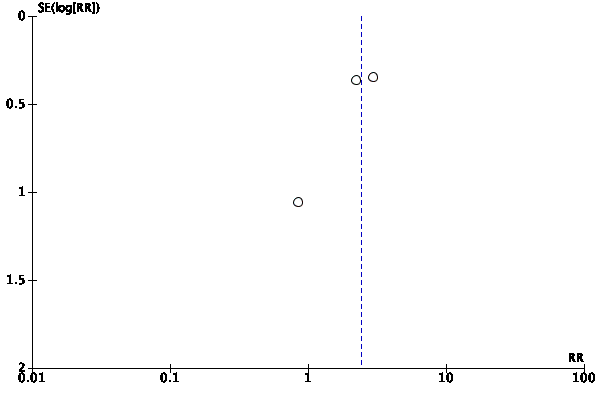
*

Serum thrombocytopaenia

Seizure activity

Focal neurological signs


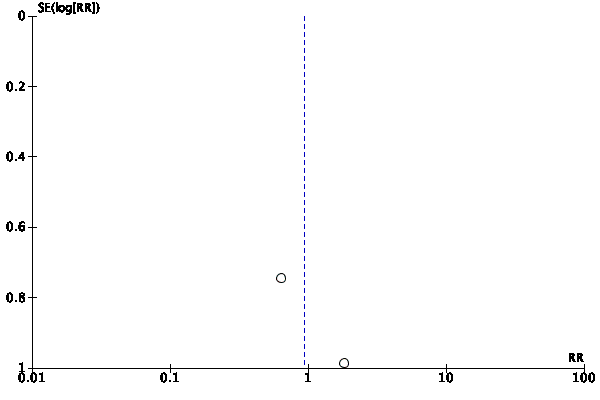
*
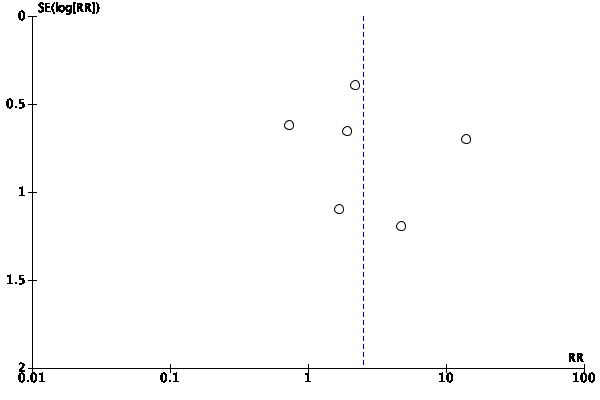

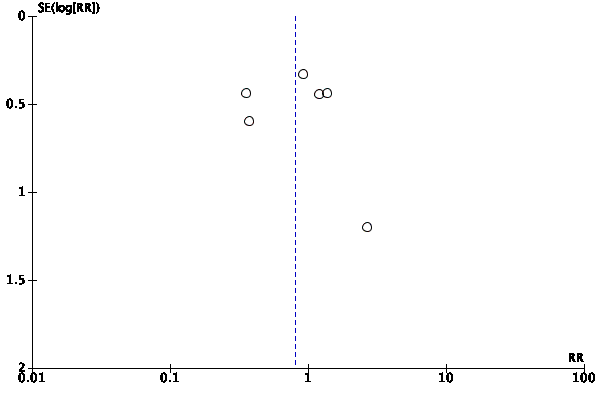
*

EEG abnormality

CSF leukocytosis

CSF elevated protein


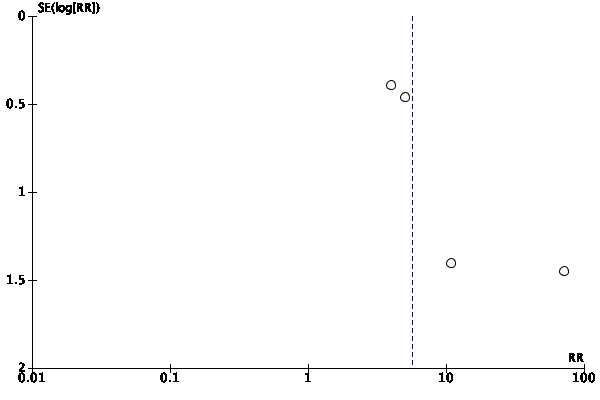

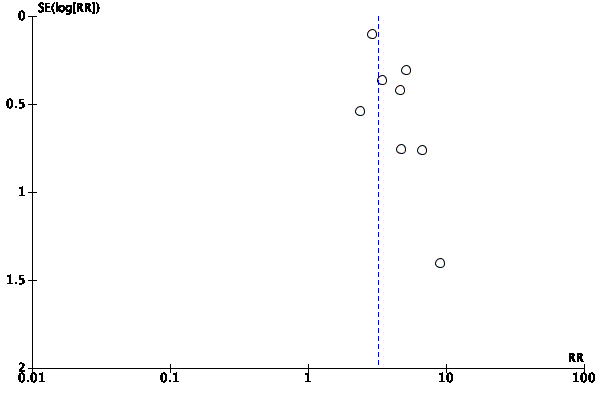


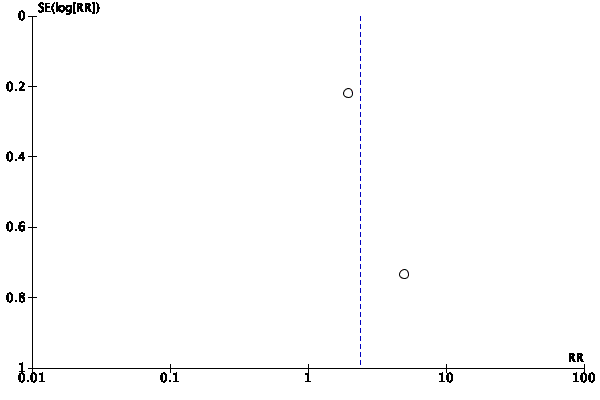


Shock

GCS<8

Intubation and ventilation

Poor outcome at discharge


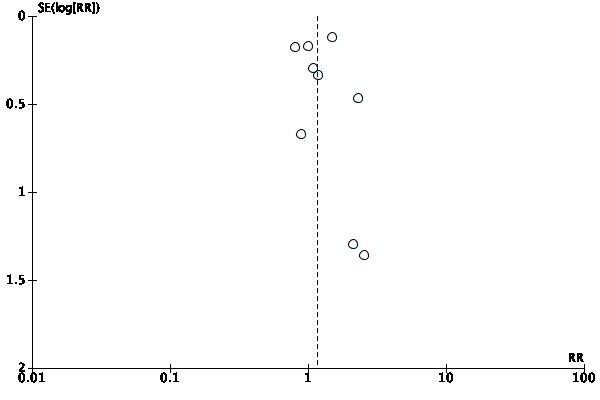

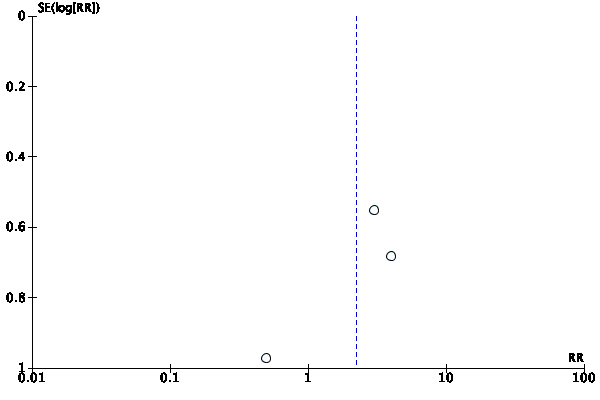


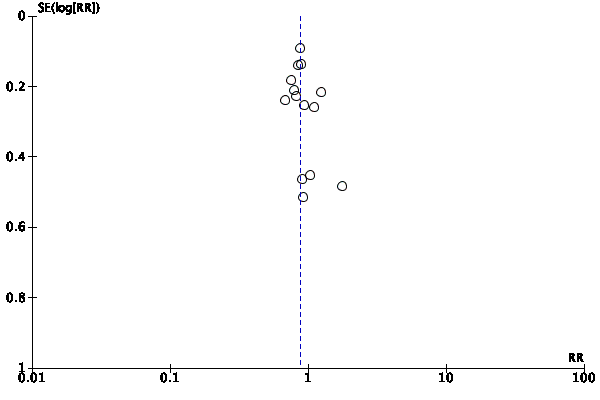


Fever

Rural (vs urban) location

Male


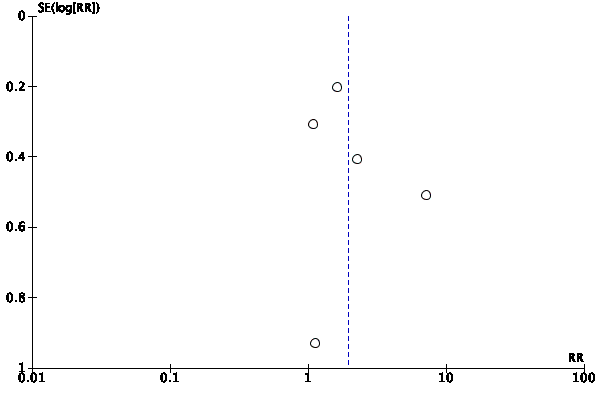


Status epilepticus


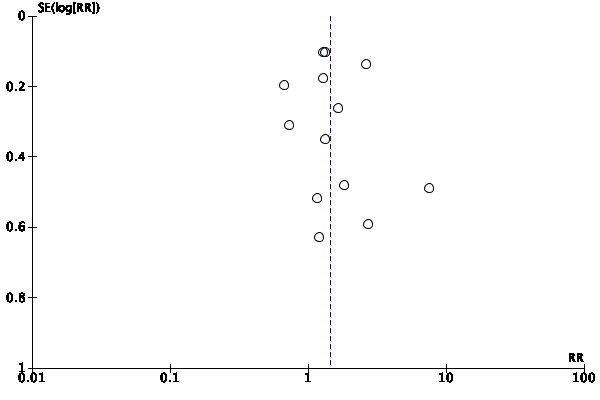


Seizure activity

Focal neurological signs


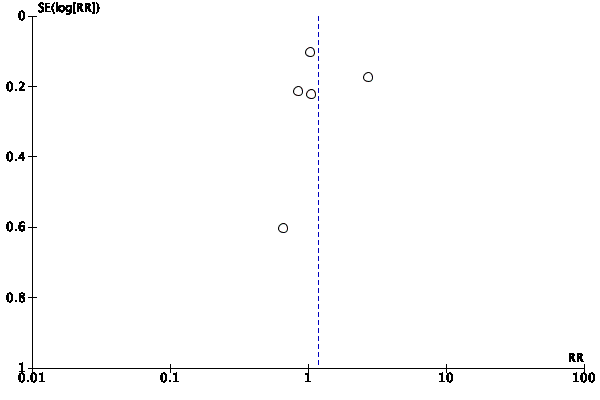

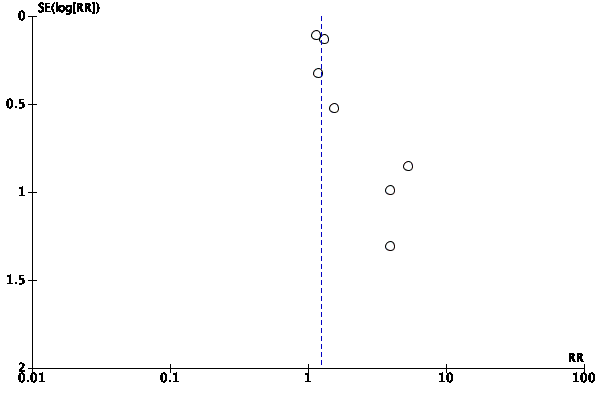


CSF elevated protein


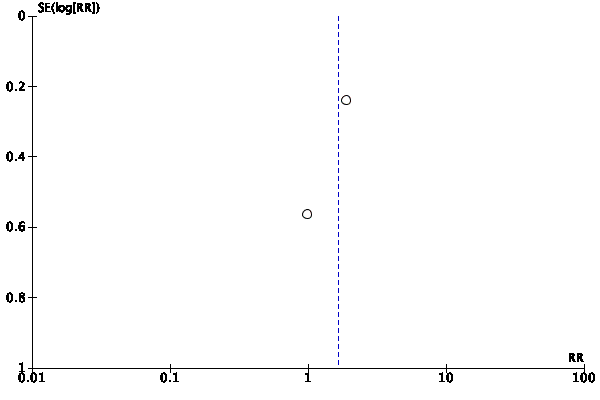


Serum thrombocytopaenia


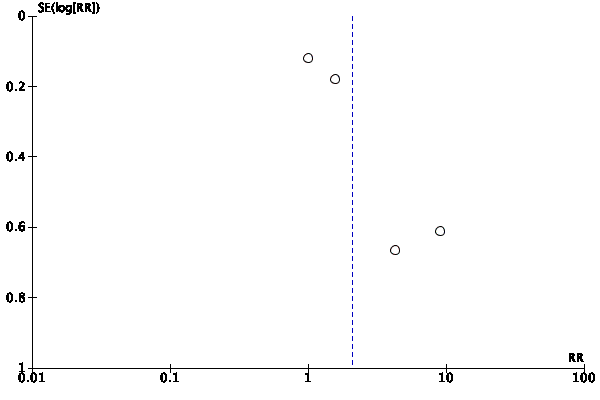


Immunocompromised


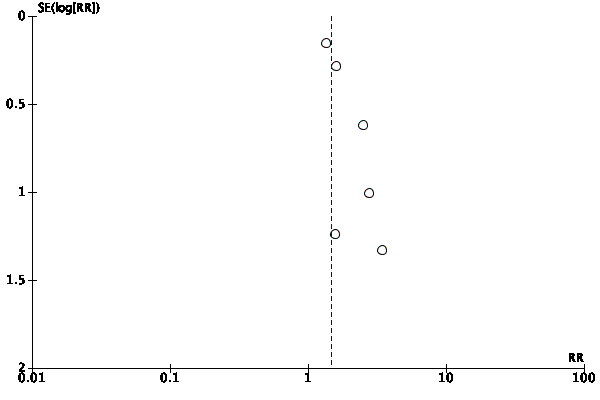

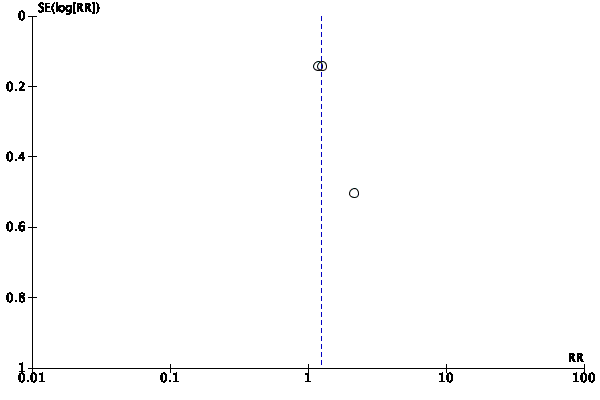

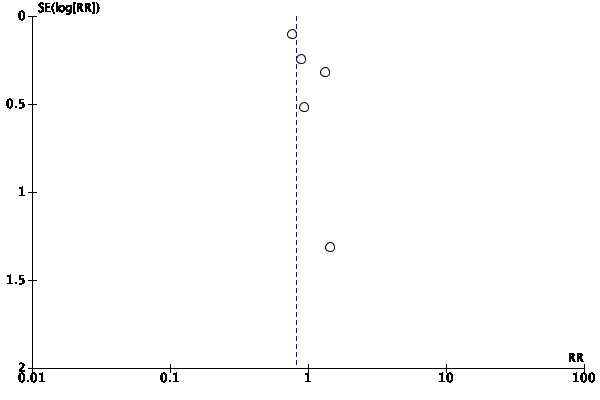


EEG abnormality

CSF leukocytosis

CSF hypoglycorachia


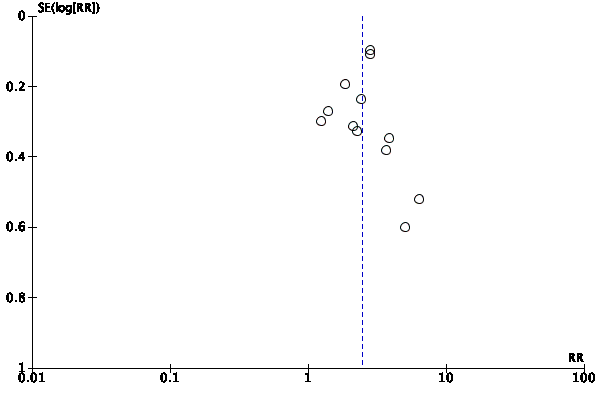


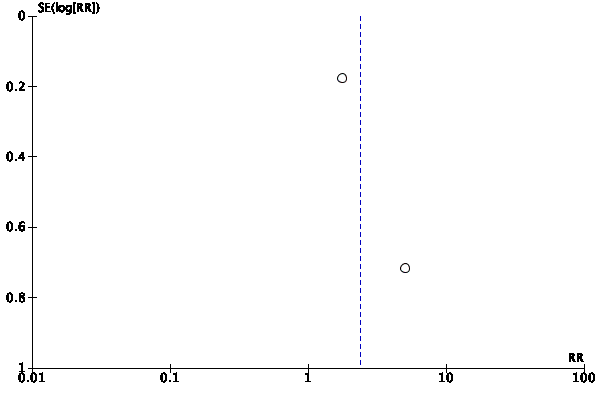

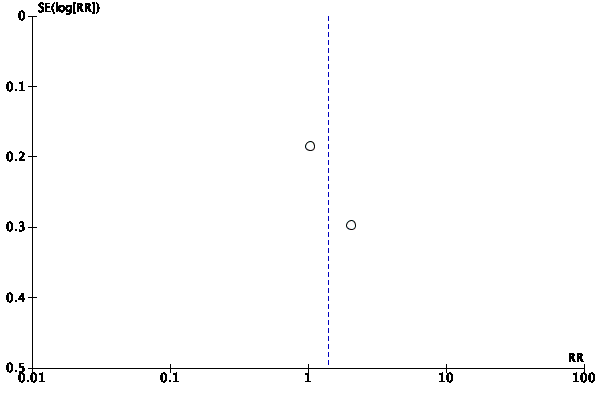


CT abnormality

MRI abnormality

GCS<8


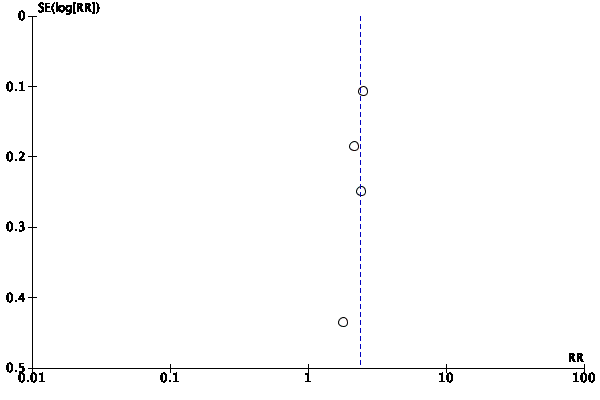


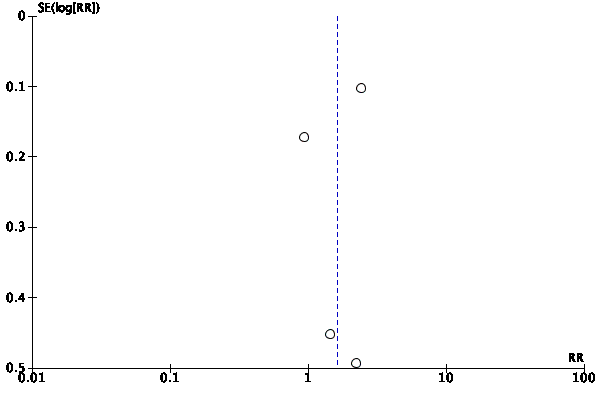

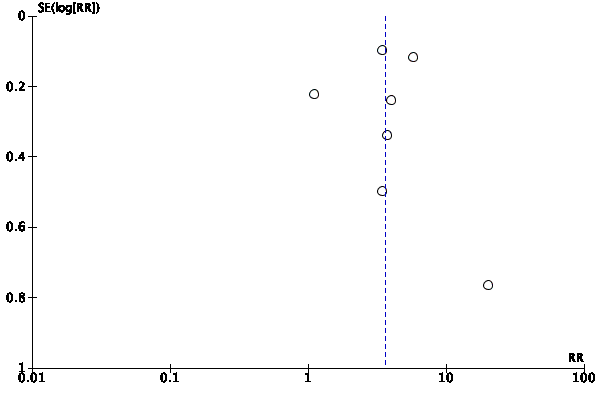


Osmotherapy

Intubation and ventilation

Steroid therapy

Poor outcome at > 6 months


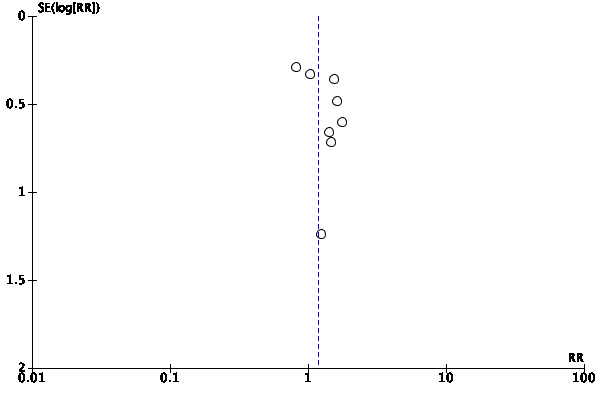

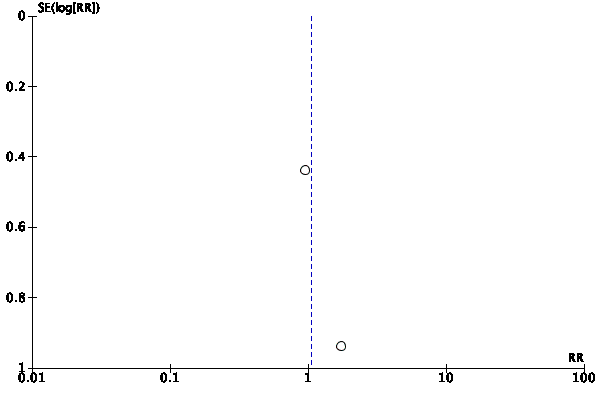

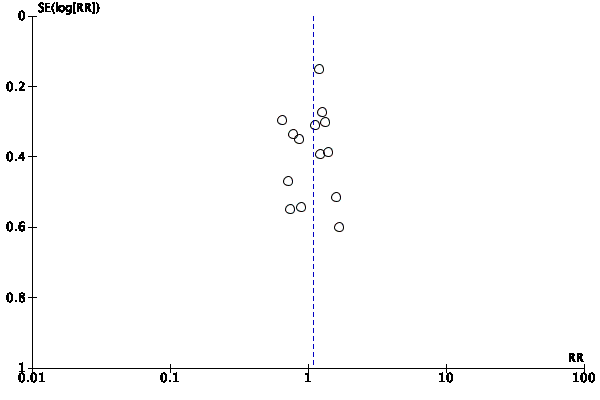


Fever

Rural (vs urban) location

Male


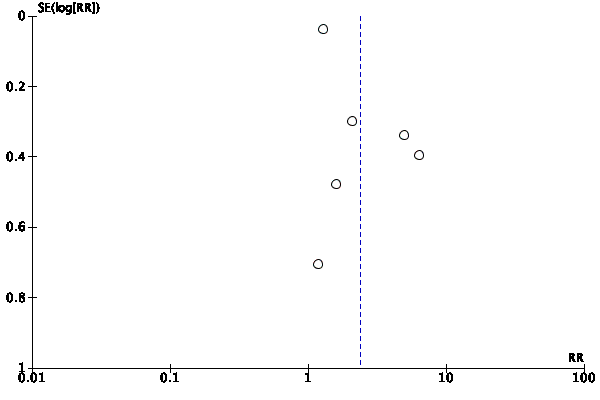


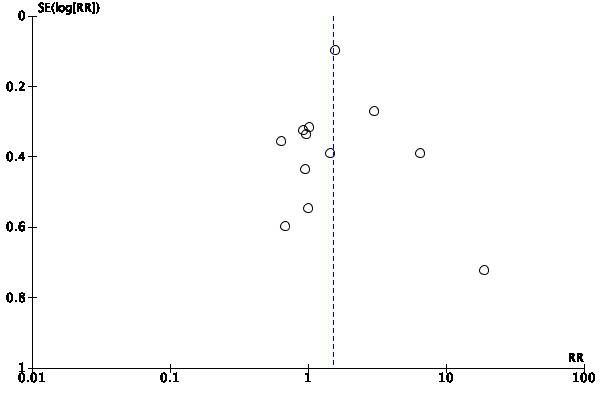

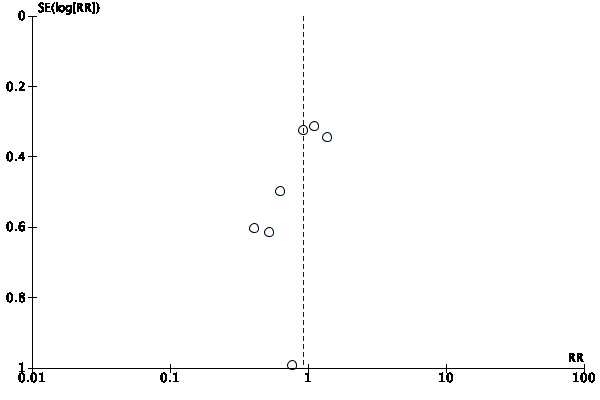


Status epilepticus

Seizure activity

Focal neurological signs


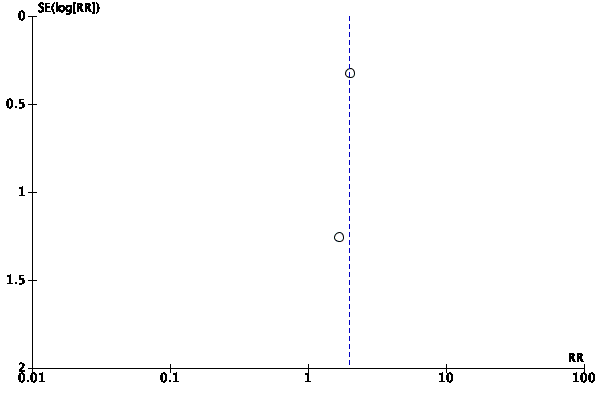

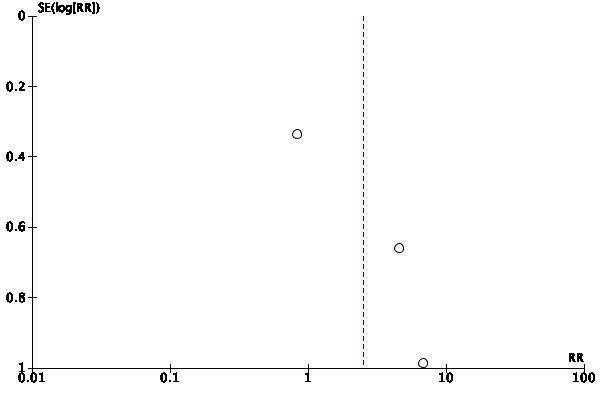

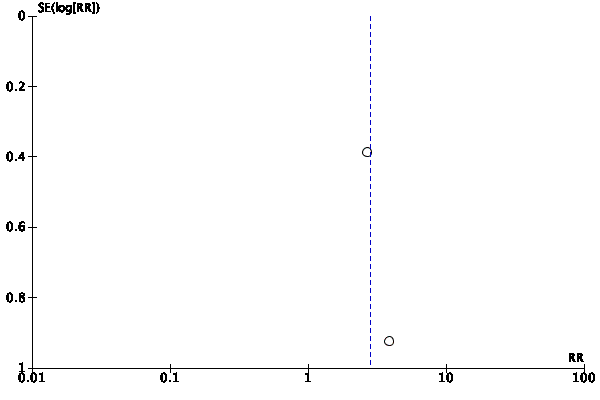


CT abnormality

EEG abnormalities

Immunocompromised


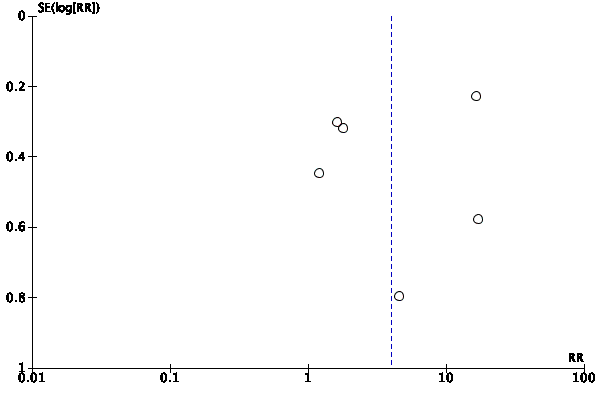


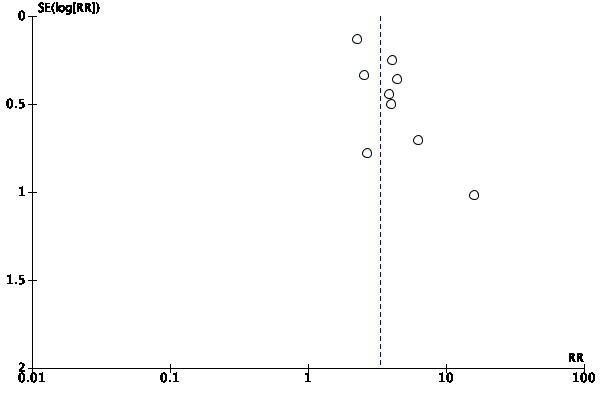

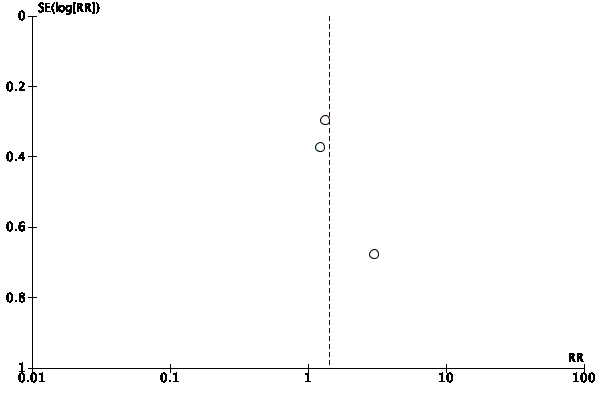


MRI abnormality

Intubation and ventilation

GCS<8


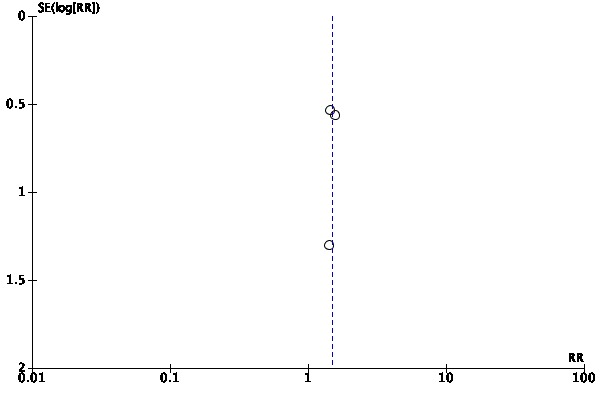


Steroid therapy
