# Supplementary material for: Clinical and Laboratory Predictors of Poor Neurological Outcomes Following Infectious Encephalitis: Systematic Review and Meta‐Analysis
Source: Eur J Neurol. 2025 Nov 26;32(12):e70445. doi: 10.1111/ene.70445 (PMC12649060; doi:10.1111/ene.70445)
Supplement: Supplementary file 7 — Table S1: ene70445‐sup‐0007‐TableS1.docx. [file ENE-32-e70445-s005.docx]

**Supplementary Table 1** **– Full characteristics of included studies and excluded studies with reasons for exclusion.**

| **Title** | **Author, date** | **Study design** | **Country** | **Male/ Female** | **Age in years (paediatric/ adult/ mixed)** | **Ethnicity % cohort** | **Socioeconomic status** | **Diagnosis** | **Evidence of aetiology** | **Sample size (n= patient number included in final analysis )** | Include/ excluded (with reason) |
| --- | --- | --- | --- | --- | --- | --- | --- | --- | --- | --- | --- |
| **Included studies** | | | | | | | | | | | |
| Computed tomography and EEG in herpes simplex encephalitis. Their value in diagnosis and prognosis. | Dutt et al, 1982 | Retrospective case series | England | Not specified | Not specified | Not specified | Not specified | HSV-E | Confirmed case of virus in CNS material or evidence of necrotising changes or HSV serology | 11 | Included |
| Fatal outcome in Japanese encephalitis | Burke et al, 1985 | Prospective cohort | Thailand | 34 male, 15 female | Paediatric (mean/ median/ mode not available) | Not specified | Not specified | JEV-E | CSF anti-JEV IgM ELISA positivity or isolation of JEV from CSF | 49 | Included |
| Correlation of tumor necrosis factor levels in the serum of cerebrospinal fluid with clinical outcome in Japanese encephalitis patients | Ravi et al, 1997 | Prospective cohort | India | 21 male, 12 female | Mixed (mean: 10.68, range: 3-40) | Not specified | Not specified | JEV-E | CSF based anti-JEV IgM ELISA and/or CSF based JEV antigen detection | 33 | Included |
| Clinical features of Nipah virus encephalitis among pig farmers in Malaysia | Goh et al, 2000 | Prospective cohort | Malaysia | 77 male, 17 female | Mixed (mean: 37, range: 13-68) | Chinese 83, Indian 14, other 3 | 97% pig farmers or occupation requiring direct animal contact | Nipah virus encephalitis | Mixed methods, including virology culture and ELISA | 94 | Included |
| Nipah encephalitis outbreak in Malaysia, clinical features in patients from Seremban | Chong et al, 2002 | Retrospective cohort | Malaysia | 91 male, 12 female | Mixed (mean: 38, range: 4-75) | Chinese 58, Indian 24, East Malaysian 4, Migrant 14, Indonesian 10, Nepalese 4 | >90% pig farmers | Nipah virus encephalitis | CSF-based anti-JEV IgM ELISA positivity | 103 | Included |
| Clinical outcome and neurological sequelae in serologically confirmed cases of Japanese encephalitis patients in Assam, India | Baruah et al, 2002 | Retrospective cohort | India | 23 male, 16 female | Paediatric (range: 3-12) | Not specified | Poor socioeconomic status' for majority of included patients (% undefined) | JEV-E | Serological anti-JEV IgM ELISA positivity | 39 | Included |
| Japanese encephalitis in and around Pondicherry, South India: A clinical appraisal and prognostic indicators for the outcome | Potula et al, 2003 | Retrospective cohort | India | 122 male, 90 female | Mixed (mean/ median/ mode not available) | Not specified | 100% rural areas/ low socioeconomic background | JEV-E | JEV-specific antigen in CSF or anti-JEV antibody in CSF | 145 | Included |
| Clinical investigation of hospitalized human cases of West Nile virus infection in Houston, Texas, 2002-2004 | Murray et al, 2008 | Retrospective cohort | North America | Not specified | Mixed (mean: 54, range 5 months - 95) | Not specified | Not specified | WNV-E | CSF or serum based anti-WNV IgM ELISA positivity | 113 | Included |
| BrainStem encephalitis associated with Chandipura in Andhra Pradesh outbreak | Rao et al, 2008 | Retrospective cohort | India | 59 male, 45 female | Paediatric (mean: 3.75, range: 9 months -11) | Not specified | Not specified | Chandipura virus encephalitis | Culture/CSF-based PCR/anti-Chandipura virus IgM antibodies | 104 | Included |
| Infectious encephalitis in France in 2007: A national prospective study | Mailles et al, 2009 | Prospective cohort | France | 154 male, 99 female | Mixed (mean: 50, range: 1 month - 89) | Not specified | Not specified | Mixed aetiology - all-cause encephalitis | Definitive identification of virus in CSF (by PCR, ELISA, or culture) | 249 | Included |
| Viral etiology of encephalitis in children in Southern Vietnam: Results of a one-year prospective descriptive study | VanTan et al, 2010 | Prospective cohort | Vietnam | 124 male, 70 female | Paediatric (median: 3, IQR: 1-7) | Not specified | Not specified | Mixed aetiology - viral encephalitis | Viral detection by PCR or culture in CSF or anti-viral IgM antibodies by serological test in CSF OR viral detection in both rectal and throat swabs EV as well as in blood by PCR, AND absence of other viruses detected in CSF | 194 | Included |
| Japanese encephalitis (JE). Part I: clinical profile of 1,282 adult acute cases of four epidemics | Sarkari et al, 2011 | Prospective case series | India | 751 male, 448 female | Mixed (mean/ median/ mode not available) | Not specified | Not specified | JEV-E | Mixed methods, centre dependent, CSF based anti-JEV IgM positivity and virus extraction from brain tissue samples | 1199 | Included |
| Clinical profile and outcome of japanese encephalities cases admitted at ICU from the month of June 2012 to November 2012 | Kakoti et al, 2013 | Prospective cohort | India | 32 male, 35 female | Paediatric (mean/ median/ mode not available) | Not specified | 90% from rural area, 63% belonging to low SE status | JEV-E | CSF-based anti-JEV IgM ELISA positivity | 61 | Included |
| An appraisal of clinicopathological parameters in Japanese encephalitis and changing epidemiological trends in upper Assam, India | Patgiri et al, 2014 | Prospective cross-sectional | India | 119 male, 75 female | Paediatric (mean/ median/ mode not available) | Not specified | 89.17% from rural area | JEV-E | CSF-based anti-JEV IgM ELISA positivity | 194 | Included |
| Severe fever with thrombocytopenia syndrome bunyavirus-related human encephalitis | Cui et al, 2015 | Retrospective cohort | China | 49 male, 54 female | Mixed (mean: 65.3, SD: ±9.8) | Not specified | Not specified | SFTS bunyavirus encephalitis | (1) isolation of SFTSV in cell culture, (2) detection of SFTSV RNA by a molecular method and (3) seroconversion or ≥4-fold increase of antibody titers between two serum samples collected at least 2 weeks apart. | 103 | Included |
| Burden of herpes simplex virus encephalitis in the United States | Modi et al, 2017 | Retrospective cohort | North America | 49 male, 51 female | Adult (median: 54, range: 37-72) | Not specified | 93% admissions to urban teaching hospitals | HSV-E | Not described - taken from NIS and KID cohorts of HCUP (US based national database) | 4871 | Included |
| Spectrum and outcome of acute infectious encephalitis/encephalopathy in an intensive care unit from India | Kalita et al, 2017 | Prospective cohort | India | 93 male, 71 female | Mixed (median: 35, range: 2-85) | Not specified | Not specified | Mixed aetiology - infectious encephalitis | Mixed methods | 164 | Included |
| Clinico-epidemiological characteristics of hospitalized acute encephalitis syndrome children and their correlation with case fatality rate. | Kakoti et al, 2020 | Prospective cohort | India | 84 male, 56 female | Paediatric (mean/ median/ mode not available) | Not specified | 94.3% admission from rural areas | JEV-E | CSF or serum based anti-JEV IgM ELISA positivity | 130 | Included |
| Clinical Features and Risk Factors for Mortality in Children With Acute Encephalitis Who Present to the Emergency Department | Hu et al, 2020 | Retrospective cohort | Taiwan | 88 male, 70 female | Paediatric (mean: 6.9, SD: ±4.6) | Not specified | Not specified | Mixed aetiology - all-cause encephalitis | Mixed methods (excluded NMDA encephalitis and other non-infectious encephalitic aetiologies) | 158 | Included |
| Clinical analysis of 17 cases of Japanese encephalitis experienced in the last ten years | Takada et al, 1989 | Retrospective case series | Japan | 7 male, 10 female | Adult (range: 33-91) | Not specified | Not specified | JEV-E | Serum haemaglutination/ complement fixation | 17 | Included |
| Predictive factors of short-term neurologic outcome in children with encephalitis | Klein et al, 1994 | Retrospective cohort | North America | 45 male, 30 female | Paediatric (mean: 6.4, range: 1 month - 19years) | Not specified | Not specified | Mixed aetiology - viral encephalitis | Either by clinical viral syndrome or by 4-fold rise in serum titre | 75 | Included |
| Acute herpes simplex encephalitis: Clinical assessment and prognostic data | Marton et al, 1996 | Retrospective cohort | Israel | 19 male, 11 female | Mixed (mean/ median/ mode not available) | Not specified | Not specified | HSV-E | CSF or serum serology, CSF PCR, or extraction from brain tissue | 30 | Included |
| Prognostic indicators of childhood acute viral encephalitis | Bhutto et al, 1999 | Retrospective cohort | Pakistan | 104 male, 42 female | Paediatric (mean/ median/ mode not available) | Not specified | Not specified | Mixed aetiology - viral encephalitis | Not specified - discharged with diagnosis of viral encephalitis (unclear what investigations were used to qualify this) | 147 | Included |
| Clinical and immunological risk factors for severe disease in Japanese encephalitis | Libraty et al, 2002 | Prospective cohort | Thailand | 56 male, 29 female | Paediatric (mean: 6.5, median: 6.0) | Not specified | Not specified | JEV-E | CSF or serum based anti-JEV IgM | 85 | Included |
| Seizures and raised intracranial pressure in Vietnamese patients with Japanese encephalitis | Solomon et al, 2002 | Prospective cohort | Vietnam | 82 male, 62 female | Mixed (mean/ median/ mode not available) | Not specified | 84% admissions from rural areas | JEV-E | CSF or serum based anti-JEV IgM | 144 | Included |
| The epidemiology, clinical features, and long-term prognosis of Japanese encephalitis in central Sarawak, Malaysia, 1997-2005 | Mong et al, 2008 | Mixed retrospective/ prospective cohort | Malaysia | 69 male, 49 male | Paediatric (mean/ median/ mode not available) | Iban 75%, Chinese 10%, Malay 10%, Other 5% | 83% admissions from rural areas | JEV-E | CSF-based anti-JEV IgM ELISA positivity | 118 | Included |
| Clinical and prognostic features among children with acute encephalitis syndrome in Nepal; a retrospective study | Rayamajhi et al, 2011 | Retrospective cohort | Nepal | 16 male, 26 female | Paediatric (mean/ median/ mode not available) | Not specified | Not specified | Mixed aetiology - viral encephalitis | Mixed methods - reliant on CSF and serum microbiology and serology to define JE and non-JE cases (extracted data from all AES suspects) | 145 | Included |
| Eastern equine encephalitis in children, Massachusetts and New Hampshire,USA, 1970-2010. | Silverman et al, 2013 | Retrospective case series | North America | 8 male, 7 female | Paediatric (mean: 6.7, range: 6 months -14.7) | Not specified | Not specified | EEV-E | Virological, CSF-based and serological confirmation | 15 | Included |
| Herpes simplex encephalitis: Clinical presentation, neurological sequelae and new prognostic factors. Ten years of experience | Riancho et al, 2013 | Retrospective cohort | Spain | 10 male, 16 female | Adult (median: 58, range: 23-90) | Not specified | Not specified | HSV-E | HSE + CSF PCR or radiological signs | 22 | Included |
| The spectrum of acute encephalitis: Causes, management, and predictors of outcome | Singh et al, 2014 | Retrospective cohort | North America | 46 male, 49 female | Adult (median: 72, range: 48-71) | Not specified | Not specified | Mixed aetiology - viral encephalitis | Not available - paper from author (single-centre EPR-based cohort study) and details of extraction are in supp material | 95 | Included |
| Influence of malnutrition on adverse outcome in children with confirmed or probable viral encephalitis: A prospective observational study | Singh et al, 2015 | Prospective cohort | India | Not specified | Paediatric (mean/ median/ mode not available) | Not specified | 82% admissions from rural areas | JEV-E/ EV-E | CSF PCR or IgM anti-JEV or EV | 114 | Included |
| Clinical characteristics and outcome of clinically diagnosed viral encephalitis in southwest China | Zhao et al, 2015 | Retrospective cohort | China | 626 male, 481 female | Mixed (mean: 38.7, range: 9-96) | Not specified | Not specified | Mixed aetiology - infectious encephalitis | AES criteria, excluding bacterial, fungal, or TB causes of AES - clinical diagnosis (primarily clinical diagnosis used) | 1107 | Included |
| Predictors of outcome in HSV encephalitis | Singh et al, 2016 | Retrospective cohort | North America | 32 male, 13 female | Adult (median: 66, IQR: 53.5–78) | Not specified | Not specified | HSV-E | CSF PCR | 45 | Included |
| Elevated levels of cerebrospinal fluid S100B are associated with brain injury and unfavorable outcomes in children with central nervous system infections | Peng et al, 2017 | Retrospective cohort | China | 38 male, 24 female | Paediatric (median: 2.5, range: 0.7-6.5) | Not specified | Not specified | Mixed aetiology - infectious encephalitis | Unclear but aetiology confirmed in portion of cases | 62 | Included |
| Determining the clinical characteristics and prognostic factors for the outcomes of Japanese encephalitis in adults: A multicenter study from southern Taiwan | Lo et al, 2019 | Retrospective cohort | Taiwan | 42 male, 26 female | Adult (median: 50, range: 22-74) | Not specified | 13.6% admissions from rural area, majority from urban or suburban areas | JEV-E | WHO JE criteria (CSF based, but also includes virus isolation from blood) | 68 | Included |
| Clinical epidemiology, risk factors, and outcomes of encephalitis in older adults | Hansen et al, 2020 | Retrospective cohort | North America | 184 male, 156 female | Adult (median: 48, range: 18-95) | Caucasian 41.3%, African American 34.4%, Hispanic 21.1%, Asian 3.2%. | Not specified | Mixed aetiology - all-cause encephalitis | Mixed methods (CSF and serum serology) | 340 | Included |
| Prognostic Factors among Children with Acute Encephalitis/Encephalopathy Associated with Viral and Other Pathogens | Hatachi et al, 2021 | Retrospective cohort | Japan (national cohort study) | Not specified (for encephalitis subgroup) | Not specified (for encephalitis subgroup) | Not specified (for encephalitis subgroup) | Not specified (for encephalitis subgroup) | Mixed aetiology - infectious encephalitis | Unclear, national cohort study with data extracted using ICD codes (assume ICD-10 codes require virological or serological diagnosis) | 2014 | Included |
| Varicella Zoster Virus Encephalitis in Denmark from 2015 to 2019-A Nationwide Prospective Cohort Study | Herlin et al, 2021 | Prospective cohort | Denmark | 47 male, 45 female | Adult (median: 75, IQR: 67–83) | Not specified | Not specified | VZV-E | CSF PCR or intrathecal antibody positivity | 92 | Included |
| Diagnosis and Therapy of Infectious Encephalitis in Children: A Ten-Years Retrospective Study | Pata et al, 2021 | Retrospective cohort | Italy | 34 male, 22 female | Paediatric: (mean: 4.7, IQR: 0.7–8.7) | Not specified | Not specified | Mixed aetiology - infectious encephalitis | Mixed methods (CSF, serum serology, cultures) | 53 | Included |
| Childhood encephalitis in the Greater Mekong region (the SouthEast Asia Encephalitis Project): a multicentre prospective study | Pommier et al, 2022 | Prospective cohort | Cambodia, Vietnam, Laos, and Myanmar | 369 male, 295 female | Paediatric (median: 4.3, IQR: 1.8–8.8) | Not specified | 50% admissions lived in accomodation with sheet metal or straw roofing | Mixed aetiology - all-cause encephalitis | Mixed methods (detailed CSF and serum-based PCR, ELISA, and virology/ culture) | 664 | Included |
| Characteristics, management, and outcomes of patients with infectious encephalitis requiring intensive care: A prospective multicentre observational study | Fillatre et al, 2023 | Prospective cohort | France | 298 male, 188 female | Adult (mean/ median/ mode not available) | Not specified | Not specified | Mixed aetiology - infectious encephalitis | Mixed methods across multiple centres at the discretion of the physician | 198 | Included |
| Viral encephalitis in children: Detection with technetium-99m HMPAO brain single-photon emission CT and its value in prediction of outcome | Kao et al, 1994 | Retrospective case series | China | 8 male, 10 female | Paediatric (mean: 8.9, range: 1-18) | Not specified | Not specified | Mixed aetiology - infectious encephalitis | Mixed methods (including EEG/ serology etc - generally unclear) | 18 | Included |
| Outcome of and prognostic factors for herpes simplex encephalitis in adult patients: Results of a multicenter study | Raschilas et al, 2002 | Retrospective cohort | France | 56 male, 37 female | Adult (mean: 53.5, SD: ±17.4) | Not specified | Not specified | HSV-E | CSF PCR HSV | 85 | Included |
| Adult herpes simplex encephalitis: Fifteen years' experience | Riera-Mestre et al, 2009 | Retrospective cohort | Spain | 22 male, 13 female | Adult (median: 54, IQR: 37–71) | Not specified | Not specified | JEV-E | CSF PCR HSV or consistent neuroimaging findings | 35 | Included |
| Outcome of children with japanese encephalitis and predictors of outcome in southwestern China | Ma and Jiang, 2013 | Retrospective cohort | China | 46 male, 41 female | Paediatric (mean/ median/ mode not available) | Not specified | Not specified | JEV-E | CSF-based or JEV-based anti-JEV serology | 87 | Included |
| Herpes simplex virus encephalitis: Clinical manifestations, diagnosis and outcome in 106 adult patients | Sili et al, 2014 | Retrospective cohort | Turkey | 63 male, 43 female | Adult: (mean: 44, range: 18–83) | Not specified | Not specified | HSV-E | CSF PCR HSV or consistent neuroimaging findings | 106 | Included |
| Prognostic Value of Initial Standard EEG and MRI in Patients with Herpes Simplex Encephalitis. | Kim et al, 2016 | Retrospective cohort | South Korea | 18 male, 11 female | Mixed (median: 41.21, range: 3–77) | Not specified | Not specified | HSV-E | CSF PCR HSV | 29 | Included |
| Research on early diagnosis and impact prognostic factors of herpes simplex encephalitis | Zhang et al, 2016 | Retrospective cohort | China | 21 male, 15 female | Paediatric (mean: 6.43) | Not specified | Not specified | HSV-E | CSF PCR or anti-IgM HSV | 36 | Included |
| Clinical characteristics and outcome of human herpesvirus-6 encephalitis after allogeneic hematopoietic stem cell transplantation | Ogata et al, 2017 | Retrospective cohort | Japan | 104 male, 40 female | Mixed (mean/ median/ mode not available) | Not specified | Not specified | HHV-6 encephalitis | CSF HHV-6 PCR | 145 | Included |
| Clinical value of dorsal medulla oblongata involvement detected with conventional magnetic resonance imaging for prediction of outcome in children with enterovirus 71-related brainstem encephalitis | Liu et al, 2019 | Retrospective cohort | China | 27 male, 19 female | Paediatric (mean: 15.5 months, SD ± 1.3) | Not specified | Not specified | EV-E | Mixed methods (including clinical presentation/ neuroimaging/ multi medium PCR agent) - have decided this doesn't match 'green' criteria as virological analysis took place in feces, for example, not blood or CSF | 46 | Included |
| Neurologic outcome of VZV encephalitis one year after ICU admission: a multicenter cohort study | Mirouse et al, 2022 | Retrospective cohort | France | 29 male, 26 female | Adult (median: 53, IQR 36–66) | Not specified | Not specified | VZV-E | CSF PCR VZV | 55 | Included |
| Prognosis analysis of 112 cases with Japanese encephalitis in adults | Wang et al, 2020 | Retrospective cohort | China | 54 male, 58 female | Adult (mean: 39.3, SD: ±20.8) | Not specified | Not specified | JEV-E | CSF anti-JEV IgM | 112 | Included |
| Study on the relationship between early clinical symptoms and prognosis of Japanese encephalitis: Based on group LASSO logistic regression model | Liu et al, 2021 | Retrospective cohort | China | 415 male, 451 female | Mixed (mean/ median/ mode not available) | Not specified | Not specified | JEV-E | Serum-based anti JEV IgM | 764 | Included |
| Extent of disability among paediatric Japanese encephalitis survivors and predictors of poor outcome: a retrospective cohort study in North India | Srivastava et al, 2022 | Retrospective cohort | India | 78 male, 71 female | Paediatric (median: 7.5, range: 1.5–15) | Not specified | 95% admission were from rural areas, 72% patients parents/ guardians work as 'daily wage labourers' | JEV-E | Laboratory-confirmed (no further clarification provided) | 149 | Included |
| The neutrophil-to-lymphocyte and monocyte-to-lymphocyte ratios are independently associated with clinical outcomes of viral encephalitis | He et al, 2023 | Retrospective cohort | China | 48 male, 33 female | Adult: (median: 39) | Not specified | Not specified | Mixed aetiology - infectious encephalitis | Mixed methods (no specific virological or serological investigation described) | 81 | Included |
| Determining the Clinical Characteristics, Treatment Strategies, and Prognostic Factors for Mycoplasma pneumoniae Encephalitis in Children: A Multicenter Study in China | Fan et al, 2023 | Retrospective cohort | China | 49 male, 38 female | Paediatric (median: 5.3, range: 6-10) | Chinese 100% | Not specified | Mycoplasma pneumoniae encephalitis | CSF PCR or IgM antiMycoP | 87 | Included |
| **Excluded studies** | | | | | | | | | | | |
| Early mental and neurological sequelae after Japanese B encephalitis | Huy et al, 1994 |  |  |  |  |  |  |  |  |  | Wrong timeframe |
| Neurophysiological changes in Japanese encephalitis | Kalita and Misra, 2002 |  |  |  |  |  |  |  |  |  | Wrong timeframe |
| Evaluation of combination therapy using aciclovir and corticosteroid in adult patients with herpes simplex virus encephalitis | Kamei et al, 2005 |  |  |  |  |  |  |  |  |  | Wrong timeframe |
| The correlation between neurological evaluations and neurological outcome in acute encephalitis: A hospital-based study | Wang et al, 2007 |  |  |  |  |  |  |  |  |  | Wrong timeframe |
| Clinical presentation, etiology, and survival in adult acute encephalitis syndrome in rural Central India | Joshi et al, 2013 |  |  |  |  |  |  |  |  |  | Wrong timeframe |
| Functional outcomes in adult patients with herpes simplex encephalitis admitted to the ICU: a multicenter cohort study | Jaquet et al, 2019 |  |  |  |  |  |  |  |  |  | Wrong timeframe |
| A study of dengue encephalitis with laboratory and clinical parameters in Tertiary Center of North India. | Li et al, 2020 |  |  |  |  |  |  |  |  |  | Wrong timeframe |
| A study of dengue encephalitis with laboratory and clinical parameters in Tertiary Center of North India. | Mehta et al, 2021 |  |  |  |  |  |  |  |  |  | Wrong timeframe |
| Study of Demographic Profile, Etiology, and Clinical Outcome in Patients Admitted With Acute Encephalitis Syndrome From the Western Part of India. | Roy and Khatri, 2022 |  |  |  |  |  |  |  |  |  | Wrong timeframe |
| Outcomes of adult patients with meningoencephalitis requiring intensive care: the international prospective multicenter EURECA study | Sonneville et al, 2022 |  |  |  |  |  |  |  |  |  | Wrong timeframe |
| Outcomes of adult patients with meningoencephalitis requiring intensive care: the international prospective multicenter EURECA study | Palmas et al, 2023 |  |  |  |  |  |  |  |  |  | Wrong timeframe |
| Virus exposure and neurodegenerative disease risk across national biobanks | Levine 2022 |  |  |  |  |  |  |  |  |  | Inappropriate outcome |
| Encephalitis in travellers: a prospective multicentre study | Picard 2023 |  |  |  |  |  |  |  |  |  | Data not analysable |
| Viral, Bacterial, Metabolic, and Autoimmune Causes of Severe Acute Encephalopathy in Sub-Saharan Africa: A Multicenter Cohort Study | Edridge 2023 |  |  |  |  |  |  |  |  |  | Inappropriate outcome |
| Neurological and neuromuscular manifestations in patients with West Nile neuroinvasive disease, Belgrade area, Serbia, season 2022 | Nikolic 2023 |  |  |  |  |  |  |  |  |  | Inappropriate patient aetiology |
| Diagnostic and prognostic value of neuron-specific enolase and S-100 protein in pediatric viral encephalitis | Alekseeva 2023 |  |  |  |  |  |  |  |  |  | Wrong study design |
| Early autoimmunity and outcome in virus encephalitis: a retrospective study based on tissue-based assay | Liu 2023 |  |  |  |  |  |  |  |  |  | Wrong patient population |
| Infectious encephalitis in elderly patients: a prospective multicentre observational study in France 2016-2019 | Petitgas 2023 |  |  |  |  |  |  |  |  |  | Overlapping patient cohort |
| THE PROGNOSTIC VALUE OF THE NPT TEST COMBINED WITH AMPLITUDE INTEGRATED ELECTROENCEPHALOGRAM IN CHILDREN WITH VE AND ITS BIORELIABILITY ANALYSIS | Liu 2023 |  |  |  |  |  |  |  |  |  | Data not analysable |
| Autoimmune complications and clinical outcomes of herpes simplex encephalitis in children: A case series | Ozcora 2023 |  |  |  |  |  |  |  |  |  | Inappropriate outcome |
| Clinical characteristics and short-term outcomes of Japanese encephalitis in pediatric and adult patients: a retrospective study in Northern China | Zhang 2023 |  |  |  |  |  |  |  |  |  | Data not analysable |
| Clinico-Etiological Profile and Predictors of Outcome in Acute Encephalitis Syndrome | Dhariwal 2023 |  |  |  |  |  |  |  |  |  | Wrong patient population |
| Epidemiology and Etiological Study of Adult Acute Encephalitis Syndrome in the Rural Setup of Purwanchal, India | Shahi 2023 |  |  |  |  |  |  |  |  |  | Wrong patient population |
| Role of Serum Ferritin and PRISM-III in Predicting Mortality in Children with Acute Encephalitis Syndrome in Northern India | Toteja 2022 |  |  |  |  |  |  |  |  |  | Full text not available |
| Epidemiological profile of acute viral encephalitis in a sample of egyptian children | Meligya 2018 |  |  |  |  |  |  |  |  |  | Data not analysable |
| Neurological features related to influenza virus in the pediatric population: a 3-year monocentric retrospective study | Goetz 2023 |  |  |  |  |  |  |  |  |  | Wrong patient population |
| Epidemiology, Etiology and Clinical Aspects of Childhood Acute Encephalitis in a Tertiary Pediatric Hospital in Costa Rica | Sevilla-Acosta 2021 |  |  |  |  |  |  |  |  |  | Inappropriate patient aetiology |
| Characteristics of enterovirus infection associated neurologic disease associated in a pediatric population in Spain | Castillo 2023 |  |  |  |  |  |  |  |  |  | Wrong patient population |
| Epilepsy following herpes simplex encephalitis - A case series | Hersh 2023 |  |  |  |  |  |  |  |  |  | Inappropriate outcome |
| A cross-sectional study to evaluate the clinical profile and predictor of adverse outcome in children with acute encephalitis syndrome | Sahay 2020 |  |  |  |  |  |  |  |  |  | Cannot access full text |
| The Clinical, Radiological, and Electrophysiological Profile of Children Presenting with Acute Fulminant Cerebral Edema Due to Suspected Encephalitis in an Eastern Indian Tertiary Care Center | Das 2023 |  |  |  |  |  |  |  |  |  | Inappropriate outcome |
| Electroencephalography for prognostication of outcome in adults with severe herpes simplex encephalitis | Jeantin 2023 |  |  |  |  |  |  |  |  |  | Overlapping patient cohort |
| Outcome of patients with meningitis and encephalitis at tertiary care hospital in Eastern Nepal | Kafle 2017 |  |  |  |  |  |  |  |  |  | Wrong patient population |
| NEUROLOGIC OUTCOMES IN HOSPITALIZED CHILDREN WITH ACUTE SARS-COV-2 OR MIS-C | Francoeur 2023 |  |  |  |  |  |  |  |  |  | Wrong patient population |
| IMPACT OF OBESITY ON CLINICAL OUTCOMES IN PATIENTS RECEIVING ACYCLOVIR FOR HSV ENCEPHALITIS | Carter 2023 |  |  |  |  |  |  |  |  |  | Data not analysable |
| Increased volume of cerebral oedema is associated with risk of acute seizure activity and adverse neurological outcomes in encephalitis - regional and volumetric analysis in a multi-centre cohort | Alam 2022 |  |  |  |  |  |  |  |  |  | Inappropriate patient aetiology |
| Factors Predictive of Varicella Zoster Virus Encephalitis/Meningitis: A Single-Center, Retrospective Study | Yuan 2022 |  |  |  |  |  |  |  |  |  | Wrong patient population |
| Comparison of functional outcome scales in paediatric acute encephalitis: Responsiveness and outcome predictors | Teo 2022 |  |  |  |  |  |  |  |  |  | Cannot access full text |
| Serum and cerebrospinal fluid phosphorylated neurofilament heavy subunit as a marker of neuroaxonal damage in tick-borne encephalitis | Fortova 2022 |  |  |  |  |  |  |  |  |  | Data not analysable |
| Chikungunya Encephalitis: an Inconsistently Reported Headache and Cause of Death in Patients with Pre-Existing Conditions | Hopkins 2022 |  |  |  |  |  |  |  |  |  | Wrong study design |
| Neuroimaging and neurological outcome of children with acute encephalitis | Poyhonen 2022 |  |  |  |  |  |  |  |  |  | Inappropriate patient aetiology |
| CSF Biomarkers in COVID-19 Associated Encephalopathy and Encephalitis Predict Long-Term Outcome | Guasp 2022 |  |  |  |  |  |  |  |  |  | Wrong patient population |
| Comorbid conditions as risk factors for West Nile neuroinvasive disease in Ontario, Canada: A population-based cohort study | Sutinen 2022 |  |  |  |  |  |  |  |  |  | Wrong patient population |
| The Comparison of Inflammatory Cytokines (IL-6 and IL-18) and Immune Cells in Japanese Encephalitis Patients With Different Progression | Zhou 2022 |  |  |  |  |  |  |  |  |  | Data not analysable |
| Efficacy and safety of intravenous high-dose immunoglobulin in treatment of the severe form of Japanese encephalitis | Chen 2022 |  |  |  |  |  |  |  |  |  | Data not analysable |
| Psychomotor Speed Predicts Outcome in Patients with Acute Meningitis and Encephalitis: A Prospective Observational Study | Koo 2022 |  |  |  |  |  |  |  |  |  | Data not analysable |
| Thrombocytopenia as a Prognostic Marker in Patients with Acute Encephalitis at a Tertiary Care Centre in Northern India | Bhardwaj 2022 |  |  |  |  |  |  |  |  |  | Cannot access full text |
| Pro-inflammatory and anti-inflamatory cytokine genes polymorphisms and susceptibility to Japanese encephalitis disease in the North Indian population | Ghildiyal 2022 |  |  |  |  |  |  |  |  |  | Data not analysable |
| Acute seizure risk in encephalitis; analysis and modelling of 436 patients from two independent multi-centre cohorts | Wood 2022 |  |  |  |  |  |  |  |  |  | Inappropriate outcome |
| Neurological performance after central nervous system complication of childhood chickenpox | Fraszczak 2021 |  |  |  |  |  |  |  |  |  | Wrong patient population |
| Characteristics and Long-term Prognosis of Danish Patients with Varicella Zoster Virus Detected in Cerebrospinal Fluid Compared with the Background Population | Omland 2021 |  |  |  |  |  |  |  |  |  | Wrong patient population |
| Enlarged periventricular space and periventricular lesion extension on baseline brain MRI predicts poor neurological outcomes in cryptococcus meningoencephalitis | Lee 2021 |  |  |  |  |  |  |  |  |  | Wrong patient population |
| Magnetic resonance tomography in the diagnostics of enteroviral encephalitis in children | Marchenko 2021 |  |  |  |  |  |  |  |  |  | Inappropriate outcome |
| Analysis on the epidemiological characteristics and disease burden of Japanese encephalitis in Gansu province | Zhao 2021 |  |  |  |  |  |  |  |  |  | Cannot access full text |
| Cerebrospinal fluid biomarkers of brain injury, inflammation and synaptic autoimmunity predict long-term neurocognitive outcome in herpes simplex encephalitis | Westman 2021 |  |  |  |  |  |  |  |  |  | Data not analysable |
| Clinical spectrum and outcome of meningoencephlitis in children less than 5 years, managed in a tertiary care hospital Rawalpindi 2018-19 | Asghar 2021 |  |  |  |  |  |  |  |  |  | Wrong patient population |
| Long-term neurological and healthcare burden of adults with japanese encephalitis: A nationwide study 2000-2015 | Chen 2021 |  |  |  |  |  |  |  |  |  | Inappropriate outcome |
| Hospital outcomes in aes cases from eastern india | Banik 2021 |  |  |  |  |  |  |  |  |  | Wrong patient population |
| Neurological and Cognitive Performance After Childhood Encephalitis | Poyhonen 2021 |  |  |  |  |  |  |  |  |  | Inappropriate outcome |
| Adjunctive steroids in adults with encephalitis: a propensity score analysis | Allana 2021 |  |  |  |  |  |  |  |  |  | Inappropriate patient aetiology |
| Assessment of Magnetic Resonance Imaging Changes and Functional Outcomes among Adults with Severe Herpes Simplex Encephalitis | Sarton 2021 |  |  |  |  |  |  |  |  |  | Overlapping patient cohort |
| Predicting Inpatient Mortality among Encephalitis Patients: A Novel Admission Risk Score | Hansen 2020 |  |  |  |  |  |  |  |  |  | Inappropriate patient aetiology |
| Predictors of Outcome in Clinically Diagnosed Viral Encephalitis Patients: A 5-Year Prospective Study | Feng 2020 |  |  |  |  |  |  |  |  |  | Cannot access full text |
| Etiology and prognosis of encephalitis in French Guianese children: a retrospective record-based study | Elenga 2020 |  |  |  |  |  |  |  |  |  | Inappropriate patient aetiology |
| Factors associated with outcome of acute encephalitis in children: A retrospective study of three referral hospitals | Santoso 2020 |  |  |  |  |  |  |  |  |  | Data not analysable |
| Cerebrospinal fluid biomarkers in patients with central nervous system infections: A retrospective study | DiStefano 2020 |  |  |  |  |  |  |  |  |  | Data not analysable |
| Longitudinal analysis of risk factors for clinical outcomes of enterobacteriaceae meningitis/ encephalitis in post-neurosurgical patients: A comparative cohort study during 2014-2019 | Shi 2020 |  |  |  |  |  |  |  |  |  | Wrong patient population |
| Clinical profile and predictor of adverse outcome in children with acute encephalitis syndrome: A cross-sectional study | Kuntal 2020 |  |  |  |  |  |  |  |  |  | Inappropriate patient aetiology |
| Acute varicella zoster encephalitis admitted to the ICU: A French multicentric cohort | Mirouse 2020 |  |  |  |  |  |  |  |  |  | Conference abstract |
| Infuenza-associated encephalitis: A French multicentric retrospective study in pediatric intensive care units | Cleuziou 2020 |  |  |  |  |  |  |  |  |  | Wrong patient population |
| Epidemiology and prognosis of acute encephalitis in pediatric intensive care unit | Lienard 2020 |  |  |  |  |  |  |  |  |  | Conference abstract |
| Encephalitis and aseptic meningitis: short-term and long-term outcome, quality of life and neuropsychological functioning | Quist-Paulsen 2019 |  |  |  |  |  |  |  |  |  | Wrong patient population |
| Spectrum of neurological complications in chikungunya fever: experience at a tertiary care centre and review of literature | Anand 2019 |  |  |  |  |  |  |  |  |  | Wrong patient population |
| Long-term seizure outcome in patients with status epilepticus due to acute encephalitis | Leng 2019 |  |  |  |  |  |  |  |  |  | Inappropriate outcome |
| Cytomegalovirus encephalitis in immunocompetent infants: A 15-year retrospective study at a single center | Guo 2019 |  |  |  |  |  |  |  |  |  | Inappropriate outcome |
| Characteristics and outcome of influenza-Associated encephalopathy/encephalitis among children in a tertiary pediatric hospital in Italy, 2017-2019 | Mastrolia 2019 |  |  |  |  |  |  |  |  |  | Wrong patient population |
| A Nationwide Survey of Pediatric-onset Japanese Encephalitis in Japan | Nanishi 2019 |  |  |  |  |  |  |  |  |  | Data not analysable |
| T3 level may be a helpful marker to predict disease prognosis of acute central nervous system viral infections | Tong 2019 |  |  |  |  |  |  |  |  |  | Wrong patient population |
| Clinical features, diagnosis, and outcome of encephalitis in French Guiana | Roux 2019 |  |  |  |  |  |  |  |  |  | Inappropriate patient aetiology |
| Electrographic Seizures in Patients with Acute Encephalitis | Viarasilpa 2019 |  |  |  |  |  |  |  |  |  | Inappropriate patient aetiology |
| Herpes simplex encephalitis: Outcomes from a 10-year retrospective single-center case series | Snider 2019 |  |  |  |  |  |  |  |  |  | Conference abstract |
| Etiology and short term outcome of acute febrile encephalopathy among children in a tertiary care centre in South India | Kunju 2019 |  |  |  |  |  |  |  |  |  | Wrong patient population |
| Predictors of poor prognosis of Japanese encephalitis in adults | Saikia 2019 |  |  |  |  |  |  |  |  |  | Overlapping patient cohort |
| Viral central nervous system infections in Canadian infants <90 days old: Encephalitis as a predictor of neurodevelopmental morbidity | Petel 2019 |  |  |  |  |  |  |  |  |  | Wrong patient population |
| Long-term outcome of acute central nervous system infection in children | Chen 2018 |  |  |  |  |  |  |  |  |  | Wrong patient population |
| Epstein-barr virus-associated encephalitis in a case-series of more than 40 patients | Dyachenko 2018 |  |  |  |  |  |  |  |  |  | Cannot access full text |
| Comparing molecular quantification of herpes simplex virus (HSV) in cerebrospinal fluid (CSF) with quantitative structural and functional disease severity in patients with HSV encephalitis (HSVE): Implications for improved therapeutic approaches | Ramirez 2018 |  |  |  |  |  |  |  |  |  | Data not analysable |
| Critical West Nile Neuroinvasive Disease | Hawkes 2018 |  |  |  |  |  |  |  |  |  | Wrong patient population |
| Frequency, symptoms, risk factors, and outcomes of autoimmune encephalitis after herpes simplex encephalitis: a prospective observational study and retrospective analysis | Armangue 2018 |  |  |  |  |  |  |  |  |  | Inappropriate outcome |
| Low TT4 as a predictor of poor outcomes in severe encephalitis: a multivariate analysis of 94 patients | Feng 2018 |  |  |  |  |  |  |  |  |  | Cannot access full text |
| Morbidity, mortality and long-term sequelae of West Nile virus disease in Quebec | Ouhoumanne 2018 |  |  |  |  |  |  |  |  |  | Wrong patient population |
| Infectious meningitis and encephalitis in adults in Denmark: a prospective nationwide observational cohort study (DASGIB) | Bodilsen 2018 |  |  |  |  |  |  |  |  |  | Wrong patient population |
| Clinical significance of IgM and IgA class anti-NMDAR antibodies in herpes simplex encephalitis | Westman 2018 |  |  |  |  |  |  |  |  |  | Inappropriate outcome |
| Association of ICAM-1 (K469E) and MCP-1-2518 A>G polymorphism with risk of Japanese encephalitis in North Indian population | Baluni 2018 |  |  |  |  |  |  |  |  |  | Data not analysable |
| Multimodal predictions of super-refractory status epilepticus and outcome in status epilepticus due to acute encephalitis | Yuan 2018 |  |  |  |  |  |  |  |  |  | Inappropriate outcome |
| Magnetic Resonance imaging to Predict Neurological Outcome in Children with Acute Encephalitis | Chan 2018 |  |  |  |  |  |  |  |  |  | Inappropriate patient aetiology |
| Prognostic factors in adults with encephalitis: An analysis of 340 cases | Hansen 2018 |  |  |  |  |  |  |  |  |  | Conference abstract |
| Characteristics, risk factors, and outcomes of encephalitis in older adults | Hansen 2018 |  |  |  |  |  |  |  |  |  | Conference abstract |
| Epidemiology, outcomes and prognosis of central nervous system infections in Singapore-preliminary results from the Singapore neurologic infections programme (SNIP) | Tan 2018 |  |  |  |  |  |  |  |  |  | Wrong patient population |
| Zika virus associated meningoencephalitis in children: Are we under reporting? | Oligbu 2018 |  |  |  |  |  |  |  |  |  | Wrong study design |
| Functional outcomes in adult patients with herpes simplex encephalitis admitted to the ICU: The HERPETICS multicenter study | Pierre 2018 |  |  |  |  |  |  |  |  |  | Conference abstract |
| FACTORS AFFECTING THE FATAL OUTCOME IN HIV-INFECTED PATIENTS WITH ENCEPHALITIS | Hvozdetska 2018 |  |  |  |  |  |  |  |  |  | Cannot access full text |
| Long-term outcomes and risk factors associated with acute encephalitis in children | Rao 2017 |  |  |  |  |  |  |  |  |  | Wrong study design |
| The clinical significance of neutrophilic pleocytosis in cerebrospinal fluid in patients with viral central nervous system infections | Jaijakul 2017 |  |  |  |  |  |  |  |  |  | Wrong patient population |
| The Spectrum and Burden of Influenza-Associated Neurological Disease in Children: Combined Encephalitis and Influenza Sentinel Site Surveillance from Australia, 2013-2015 | Britton 2017 |  |  |  |  |  |  |  |  |  | Wrong patient population |
| Increased rates of sequelae post-encephalitis in individuals attending primary care practices in the United Kingdom: a population-based retrospective cohort study | Granerod 2017 |  |  |  |  |  |  |  |  |  | Wrong study design |
| Significance of CCL2, CCL5 and CCR2 polymorphisms for adverse prognosis of Japanese encephalitis from an endemic population of India | Chowdhury 2017 |  |  |  |  |  |  |  |  |  | Data not analysable |
| Risk Factors and Prognosis of Secondary Epilepsy in Children with Viral Encephalitis | Huang 2017 |  |  |  |  |  |  |  |  |  | Inappropriate outcome |
| Peculiarities at clinical course and prognostic factors of the most common viral infections of the central nervous system | Pekova 2017 |  |  |  |  |  |  |  |  |  | Inappropriate outcome |
| Incidence and mortality of herpes simplex encephalitis in Denmark: A nationwide registry-based cohort study | Jorgensen 2017 |  |  |  |  |  |  |  |  |  | Data not analysable |
| Ai 2017 | Ai 2017 |  |  |  |  |  |  |  |  |  | Data not analysable |
| Chuck 2017 | Chuck 2017 |  |  |  |  |  |  |  |  |  | Conference abstract |
| Teo 2017 | Teo 2017 |  |  |  |  |  |  |  |  |  | Inappropriate patient aetiology |
| Calleri 2017 | Calleri 2017 |  |  |  |  |  |  |  |  |  | Wrong patient population |
| Balakrishnan 2016 | Balakrishnan 2016 |  |  |  |  |  |  |  |  |  | Inappropriate outcome |
| Mohammad 2016 | Mohammad 2016 |  |  |  |  |  |  |  |  |  | Inappropriate outcome |
| Lenhard 2016 | Lenhard 2016 |  |  |  |  |  |  |  |  |  | Inappropriate outcome |
| Kaewpoowat 2016 | Kaewpoowat 2016 |  |  |  |  |  |  |  |  |  | Wrong patient population |
| Acute varicella zoster encephalitis admitted to the ICU: A case series of 47 patients | Canet 2016 |  |  |  |  |  |  |  |  |  | Conference abstract |
| Utility of CSF cytokine/chemokines as markers of active intrathecal inflammation: Comparison of demyelinating, anti-NMDAR and enteroviral encephalitis | Kothur 2016 |  |  |  |  |  |  |  |  |  | Wrong patient population |
| Early-onset status epilepticus in patients with acute encephalitis | Sonneville 2016 |  |  |  |  |  |  |  |  |  | Inappropriate outcome |
| The Importance of Haematological and Biochemical Findings in Patients with West Nile Virus Neuroinvasive Disease | Urosevic 2016 |  |  |  |  |  |  |  |  |  | Wrong patient population |
| EEG abnormalities as diagnostic and prognostic factor for encephalitis | Gavrilovic 2016 |  |  |  |  |  |  |  |  |  | Wrong patient population |
| Childhood encephalitis: relationship between diffusion abnormalities and clinical outcome | Wong 2015 |  |  |  |  |  |  |  |  |  | Data not analysable |
| Paroxysmal Sympathetic Hyperactivity in Critically Ill Children with Encephalitis and Meningoencephalitis | Farias-Moeller 2015 |  |  |  |  |  |  |  |  |  | Inappropriate outcome |
| Epidemics of the central nervous system infections caused by west nile virus in the territory of the south backa district, Vojvodina, Serbia | Sevic 2015 |  |  |  |  |  |  |  |  |  | Wrong patient population |
| Long-term outcome of severe herpes simplex encephalitis: A population-based observational study | Jouan 2015 |  |  |  |  |  |  |  |  |  | Data not analysable |
| Epilepsy After Resolution of Presumed Childhood Encephalitis | Rismanchi 2015 |  |  |  |  |  |  |  |  |  | Inappropriate outcome |
| Electroencephalography for diagnosis and prognosis of acute encephalitis | Sutter 2015 |  |  |  |  |  |  |  |  |  | Inappropriate patient aetiology |
| EEG findings may predict outcomes of herpes simplex encephalitis | Fujii 2015 |  |  |  |  |  |  |  |  |  | Conference abstract |
| Long-term motor and cognitive outcome of acute encephalitis | Michaeli 2014 |  |  |  |  |  |  |  |  |  | Cannot access full text |
| Encephalitis Hospitalization Rates and Inpatient Mortality in the United States, 2000-2010 | George 2014 |  |  |  |  |  |  |  |  |  | Inappropriate patient aetiology |
| Nationwide survey of rotavirus-associated encephalopathy and sudden unexpected death in Japan | Kawamura 2014 |  |  |  |  |  |  |  |  |  | Wrong patient population |
| Clinical features, outcomes, and cerebrospinal fluid findings in adult patients with central nervous system (CNS) infections caused by varicella-zoster virus: Comparison with enterovirus CNS infections | Hong 2014 |  |  |  |  |  |  |  |  |  | Wrong patient population |
| Childhood rubella encephalitis: Diagnosis, management, and outcome | Chaari 2014 |  |  |  |  |  |  |  |  |  | Inappropriate outcome |
| Clinical characteristics and functional outcome of patients with West Nile neuroinvasive disease in Serbia | Milosevic 2014 |  |  |  |  |  |  |  |  |  | Wrong patient population |
| Clinical and laboratory predictors of outcome in patients with herpes simplex encephalitis | Khaled 2014 |  |  |  |  |  |  |  |  |  | Full text not available |
| Spectrum and outcome predictors of central nervous system infections in a neurological critical care unit in India: A retrospective review | Misra 2014 |  |  |  |  |  |  |  |  |  | Wrong patient population |
| Neurofilament heavy chain as a marker of neuroaxonal pathology and prognosis in acute encephalitis | Sellner 2014 |  |  |  |  |  |  |  |  |  | Data not analysable |
| West Nile virus neuroinvasive disease: Neurological manifestations and prospective longitudinal outcomes | HartJr 2014 |  |  |  |  |  |  |  |  |  | Wrong patient population |
| Assessment of pediatric cases with chickenpox and zona hospitalised at our inpatient clinics | Caliskan 2014 |  |  |  |  |  |  |  |  |  | Wrong patient population |
| Clinical and laboratory predictors of outcome in patients with herpes simplex encephalitis | Nour 2014 |  |  |  |  |  |  |  |  |  | Conference abstract |
| A study of dengue encephalitis in tertiary center of North India | Verma 2014 |  |  |  |  |  |  |  |  |  | Conference abstract |
| Thrombocytopenia as a prognostic factor in encephalitis | Asemota 2014 |  |  |  |  |  |  |  |  |  | Cannot access full text |
| Acute febrile encephalopathy in children and predictors of mortality | Bokade 2014 |  |  |  |  |  |  |  |  |  | Wrong patient population |
| Severe cases of tick-borne meningoencephalitis - - is age the only factor unfavourably influencing the patient's prognosis? | Smiskova 2013 |  |  |  |  |  |  |  |  |  | Cannot access full text |
| Brainstem encephalitis: Etiologies, treatment, and predictors of outcome | Tan 2013 |  |  |  |  |  |  |  |  |  | Wrong patient population |
| Clinical and radiological spectrum of Japanese encephalitis | Basumatary 2013 |  |  |  |  |  |  |  |  |  | Data not analysable |
| Predictors of outcome in acute encephalitis | Thakur 2013 |  |  |  |  |  |  |  |  |  | Inappropriate patient aetiology |
| Th1 immune response takeover among patients with severe Japanese encephalitis infection | Pujhari 2013 |  |  |  |  |  |  |  |  |  | Data not analysable |
| Tick-borne encephalitis carries a high risk of incomplete recovery in children | Fowler 2013 |  |  |  |  |  |  |  |  |  | Inappropriate outcome |
| Clinical and radiological predictors of outcome for murray valley encephalitis | Tsukahara 2013 |  |  |  |  |  |  |  |  |  | Data not analysable |
| Clinical and radiological predictors of outcome for murray valley encephalitis | Speers 2013 |  |  |  |  |  |  |  |  |  | Insufficient population size |
| Epidemiology, outcomes and predictors of recovery in childhood encephalitis: A hospital-based study | Dubray 2013 |  |  |  |  |  |  |  |  |  | Inappropriate patient aetiology |
| Radiological predictors of poor outcome in herpes simplex encephalitis | Bhargava 2013 |  |  |  |  |  |  |  |  |  | Conference abstract |
| Outcome of patients admitted to the ICU with west Nile virus neuroinvasive disease | Sakagianni 2013 |  |  |  |  |  |  |  |  |  | Conference abstract |
| Clinical spectrum and outcomes of patients with encephalitis requiring ICU admission | Sonneville 2013 |  |  |  |  |  |  |  |  |  | Conference abstract |
| Long-term clinical outcome and prognostic factor of herpes simplex encephalitis: Seizure manifestation and EEG findings | Kim 2013 |  |  |  |  |  |  |  |  |  | Conference abstract |
| Childhood encephalitis: Epidemiological, clinical and radiological characteristics and their impact on the outcome | Liptai 2013 |  |  |  |  |  |  |  |  |  | Conference abstract |
| Acute encephalitis in the ICU: Predictors of poor outcome | Thakur 2013 |  |  |  |  |  |  |  |  |  | Conference abstract |
| Clinical profile and outcome of japanese encephalities cases admitted at ICU from the month of June 2012 to November 2012 | Das 2013 |  |  |  |  |  |  |  |  |  | Conference abstract |
| Hyponatremia, acute kidney injury, and mortality in HIV-related toxoplasmic encephalitis | Liborio 2012 |  |  |  |  |  |  |  |  |  | Data not analysable |
| In enterovirus 71 encephalitis with cardio-respiratory compromise, elevated interleukin 1, interleukin 1 receptor antagonist, and granulocyte colony-stimulating factor levels are markers of poor prognosis | Griffiths 2012 |  |  |  |  |  |  |  |  |  | Data not analysable |
| Pediatric herpes simplex virus encephalitis: A critical multicentric analysis | Schleede 2012 |  |  |  |  |  |  |  |  |  | Conference abstract |
| Viral load, cytokine storm, TNF-alpha and TNF-alpha promoter polymorphism as possible predictors for disease progression in Japanese encephalitis | Pujhari 2012 |  |  |  |  |  |  |  |  |  | Conference abstract |
| The prognostic factors of acute encephalitis in children | Lee 2012 |  |  |  |  |  |  |  |  |  | Conference abstract |
| Medical risk factors for severe West Nile virus disease, United States, 2008-2010 | Lindsey 2012 |  |  |  |  |  |  |  |  |  | Wrong patient population |
| Role of IL-6 and neopterin in the pathogenesis of herpetic encephalitis | Bociaga-Jasik 2011 |  |  |  |  |  |  |  |  |  | Data not analysable |
| Seizure attacks in viral encephalitis: Infl uence on a course and outcome | Rzadkiewicz 2011 |  |  |  |  |  |  |  |  |  | Conference abstract |
| Varicella zoster encephalitis - Relationship between viral load, time and outcome | Michael 2011 |  |  |  |  |  |  |  |  |  | Conference abstract |
| Acute varicella zoster virus encephalitis in adults: Relationship between viral load, time, clinical features and outcome | BenedictDaniel 2011 |  |  |  |  |  |  |  |  |  | Conference abstract |
| Clinical predictors of outcome in children with acute febrile encephalopathy admitted in pediatric intensive care unit (PICU) | Bansal 2011 |  |  |  |  |  |  |  |  |  | Conference abstract |
| Determinants of outcomes following acute child encephalopathy and encephalitis: Pivotal effect of early and delayed cooling | Kawano 2011 |  |  |  |  |  |  |  |  |  | Data not analysable |
| [Multi-center investigation of the hospitalized children with 2009 influenza A (H1N1) infection] | 2009InfluenzaA(H1N1)ClinicalCaseInvestigationGroup 2010 |  |  |  |  |  |  |  |  |  | Wrong patient population |
| Predictors of a prolonged clinical course in adult patients with herpes simplex virus encephalitis | Taira 2009 |  |  |  |  |  |  |  |  |  | Overlapping patient cohort |
| Cluster analysis for identifying sub-groups and selecting potential discriminatory variables in human encephalitis | Hamid 2010 |  |  |  |  |  |  |  |  |  | Inappropriate study design |
| Acute disseminated encephalomyelitis (ADEM)-Functional outcome predictors | Lin 2010 |  |  |  |  |  |  |  |  |  | Wrong patient population |
| Long-term outcomes of acute encephalitis in childhood | Fowler 2010 |  |  |  |  |  |  |  |  |  | Cannot access full text |
| Brainstem encephalitis: An unusual presentation of herpes simplex virus infection | Livorsi 2010 |  |  |  |  |  |  |  |  |  | Wrong study design |
| Disability after encephalitis: Development and validation of a new outcome score | Lewthwaite 2010 |  |  |  |  |  |  |  |  |  | Wrong study design |
| A clinical study of status epilepticus related acute virus encephalitis | Huang 2009 |  |  |  |  |  |  |  |  |  | Inappropriate outcome |
| Monitoring of herpes simplex virus DNA types 1 and 2 viral load in cerebrospinal fluid by real-time PCR in patients with herpes simplex encephalitis | Schloss 2009 |  |  |  |  |  |  |  |  |  | Data not analysable |
| Seizures in encephalitis: Predictors and outcome | Misra 2009 |  |  |  |  |  |  |  |  |  | Inappropriate outcome |
| Prognostic value of intrathecal antibody production and DNA viral load in cerebrospinal fluid of patients with herpes simplex encephalitis | Hjalmarsson 2009 |  |  |  |  |  |  |  |  |  | Data not analysable |
| Prognostic value of cerebrospinal fluid cytokine changes in herpes simplex virus encephalitis | Kamei 2009 |  |  |  |  |  |  |  |  |  | Overlapping patient cohort |
| Persistence of herpes simplex virus DNA in cerebrospinal fluid of neonates with herpes simplex virus encephalitis | Mejias 2009 |  |  |  |  |  |  |  |  |  | Inappropriate outcome |
| Herpes simplex encephalitis in Sweden, 1990-2001: Incidence, morbidity, and mortality | Hjalmarsson 2007 |  |  |  |  |  |  |  |  |  | Data not analysable |
| Etiology and results of treatment of adults with bacterial encephalomeningitis | Piekarska 2008 |  |  |  |  |  |  |  |  |  | Wrong patient population |
| The prognostic role of magnetic resonance imaging and single-photon emission computed tomography in viral encephalitis | Misra 2008 |  |  |  |  |  |  |  |  |  | Data not analysable |
| Outcome of herpes simplex encephalitis in children | Hsieh 2007 |  |  |  |  |  |  |  |  |  | Cannot access full text |
| Relationship between inflammatory changes revealed in cerebrospinal fluid and prognosis in acute viral encephalitis | Hlebowicz 2007 |  |  |  |  |  |  |  |  |  | Cannot access full text |
| Childhood encephalitis in Sweden: Etiology, clinical presentation and outcome | Fowler 2008 |  |  |  |  |  |  |  |  |  | Data not analysable |
| Imaging of Topographic Viral CNS Infections | Rumboldt 2008 |  |  |  |  |  |  |  |  |  | Wrong study design |
| Cognitive impairment after tick-borne encephalitis | Gustaw-Rothenberg 2008 |  |  |  |  |  |  |  |  |  | Inappropriate outcome |
| Acute encephalitis: report on 32 consecutive pediatric cases observed in one hospital | Vial 2007 |  |  |  |  |  |  |  |  |  | Data not analysable |
| Study of Japanese encephalitis and other viral encephalitis in Nepali children | Rayamajhi 2007 |  |  |  |  |  |  |  |  |  | Data not analysable |
| Toxoplasma meningoencephalitis in HIV-seronegative patients: clinical patterns, imaging features and treatment outcome | Pradhan 2007 |  |  |  |  |  |  |  |  |  | Wrong patient population |
| Neurologic, neuropsychologic, and electroencephalographic findings after European tick-borne encephalitis in children | Schmolck 2005 |  |  |  |  |  |  |  |  |  | Wrong patient population |
| The emergence of West Nile virus during a large outbreak in Illinois in 2002 | Huhn 2005 |  |  |  |  |  |  |  |  |  | Wrong patient population |
| Clinico-laboratory profile and outcome of Japanese encephalitis in Nepali children | Rayamajhi 2006 |  |  |  |  |  |  |  |  |  | Data not analysable |
| Long-term cognitive and motor deficits after enterovirus 71 brainstem encephalitis in children | Huang 2006 |  |  |  |  |  |  |  |  |  | Inappropriate outcome |
| CSF findings in 250 patients with serologically confirmed West Nile virus meningitis and encephalitis | Tyler 2006 |  |  |  |  |  |  |  |  |  | Wrong patient population |
| Childhood encephalopathy: Viruses, immune response, and outcome | Clarke 2006 |  |  |  |  |  |  |  |  |  | Wrong study design |
| Risk factors for encephalitis and death from West Nile virus infection | Murray 2006 |  |  |  |  |  |  |  |  |  | Overlapping patient cohort |
| West Nile Virus disease: A descriptive study of 228 patients hospitalized in a 4-county region of Colorado in 2003 | Bode 2006 |  |  |  |  |  |  |  |  |  | Wrong patient population |
| Clinical features and factors of unfavorable outcomes for non-polio enterovirus infection of the central nervous system in northern Taiwan, 1994-2003 | Yang 2005 |  |  |  |  |  |  |  |  |  | Wrong patient population |
| Therapy of focal viral encephalitis in children with aciclovir and recombinant beta-interferon - Results of a placebo-controlled multicenter study | Wintergerst 2005 |  |  |  |  |  |  |  |  |  | Wrong study design |
| Long-term prognosis for clinical West Nile virus infection | Klee 2004 |  |  |  |  |  |  |  |  |  | Wrong study design |
| Diagnosis, treatment, and prognosis of Mycoplasma pneumoniae childhood encephalitis: Systematic review of 58 cases | Daxboeck 2004 |  |  |  |  |  |  |  |  |  | Wrong study design |
| Acoustic brain stem evoked potentials in patients in patients with different forms of acute tick-borne encephalitis | Nadezhdina 2001 |  |  |  |  |  |  |  |  |  | Data not analysable |
| West Nile viral meningo-encephalitis in Tunisia | Triki 2001 |  |  |  |  |  |  |  |  |  | Wrong patient population |
| Nipah encephalitis outbreak in Malaysia, clinical features in patients from Seremban | Ziebold 2001 |  |  |  |  |  |  |  |  |  | Wrong patient population |
| Severe complications of varicella in previously healthy children in Germany: a 1-year survey | Dunlop 2002 |  |  |  |  |  |  |  |  |  | Inappropriate outcome |
| Early psychomotor slowing predicts the development of HIV dementia and autopsy verified HIV encephalitis | Hosoya 2002 |  |  |  |  |  |  |  |  |  | Data not analysable |
| Age of meningitis or encephalitis is independently predictive of outcome from anterior temporal lobectomy | O'Brien 2002 |  |  |  |  |  |  |  |  |  | Inappropriate outcome |
| Prognosis of Japanese encephalitis patients with dystonia compared to those with parkinsonian features only | Misra 2002 |  |  |  |  |  |  |  |  |  | Data not analysable |
| Tickborne encephalitis in an area of high endemicity in lithuania: Disease severity and long-term prognosis | Mickiene 2002 |  |  |  |  |  |  |  |  |  | Wrong patient population |
| Encephalitis and encephalopathy associated with an influenza epidemic in Japan | Morishima 2002 |  |  |  |  |  |  |  |  |  | Wrong patient population |
| Clinical characteristics of the West Nile fever outbreak, Israel, 2000 | Chowers 2001 |  |  |  |  |  |  |  |  |  | Wrong patient population |
| The presence of nipah virus in respiratory secretions and urine of patients during an outbreak of nipah virus encephalitis in Malaysia | Chua 2001 |  |  |  |  |  |  |  |  |  | Overlapping patient cohort |
| Laboratory findings in tick-borne encephalitis - Correlation with clinical outcome | Kaiser 2000 |  |  |  |  |  |  |  |  |  | Data not analysable |
| High mortality in Nipah encephalitis is associated with presence of virus in cerebrospinal fluid | Chua 2000 |  |  |  |  |  |  |  |  |  | Overlapping patient cohort |
| Tuberculous meningo-encephalitis in pediatrics. Considerations on 71 cases (1968-1993) | Romano 1996 |  |  |  |  |  |  |  |  |  | Wrong patient population |
| Modified Glasgow Coma Scale to predict mortality in children with acute infections of the central nervous system | Awasthi 1997 |  |  |  |  |  |  |  |  |  | Wrong patient population |
| Prognostic significance of Glasgow coma scale in acute viral encephalitis | Gambhir 1999 |  |  |  |  |  |  |  |  |  | Cannot access full text |
| Severe acute encephalitis in children: 14 consecutive cases | Guerguerian 1999 |  |  |  |  |  |  |  |  |  | Data not analysable |
| Prognosis of Japanese encephalitis: A multivariate analysis | Misra 1998 |  |  |  |  |  |  |  |  |  | Data not analysable |
| Australian encephalitis in the Northern Territory: Clinical and epidemiological features, 1987-1996 | Burrow 1998 |  |  |  |  |  |  |  |  |  | Data not analysable |
| EEG in Japanese encephalitis: A clinico-radiological correlation | Kalita 1998 |  |  |  |  |  |  |  |  |  | Data not analysable |
| C-Reactive protein in CNS infection in children | Jan-98 |  |  |  |  |  |  |  |  |  | Data not analysable |
| Is EEG useful in assessing patients with acute encephalitis treated with acyclovir? | Siren 1998 |  |  |  |  |  |  |  |  |  | Wrong patient population |
| Acute encephalitis in Swiss children: Aetiology and outcome | Iff 1998 |  |  |  |  |  |  |  |  |  | Wrong patient population |
| Regional cerebral blood flow scintigraphy in tick-borne encephalitis and other aseptic meningoencephalitis | Gunther 1998 |  |  |  |  |  |  |  |  |  | Data not analysable |
| Intrathecal IgM, IgA and IgG antibody response in tick-borne encephalitis. Long-term follow-up related to clinical course and outcome | Gunther 1997 |  |  |  |  |  |  |  |  |  | Data not analysable |
| Proinflammatory cytokine levels in cerebrospinal fluid from children with acute encephalitis | Ichiyama 1997 |  |  |  |  |  |  |  |  |  | Data not analysable |
| Herpes simplex encephalitis in neonates: A retrospective study of 12 cases | Bigotte 1993 |  |  |  |  |  |  |  |  |  | Data not analysable |
| Prognostic factors of early sequelae and fatal outcome of Japanese encephalitis | Luo 1995 |  |  |  |  |  |  |  |  |  | Cannot access full text |
| Acute encephalopathy: Diagnosis and outcome in patients at a regional neurological unit | Ginsberg 1994 |  |  |  |  |  |  |  |  |  | Wrong patient population |
| Epidemiology of acute childhood encephalitis: Aichi prefecture, Japan, 1984-90 | Ishikawa 1993 |  |  |  |  |  |  |  |  |  | Wrong patient population |
| A follow-up study of children with neonatal herpes simplex virus infections with particular regard to late nervous disturbances | Malm 1991 |  |  |  |  |  |  |  |  |  | Wrong patient population |
| Prognosis of herpes simplex encephalitis. A retrospective study of 19 cases | Buge 1988 |  |  |  |  |  |  |  |  |  | Data not analysable |
| Toxoplasma encephalitis in patients with the acquired immunodeficiency syndrome | Renold 1992 |  |  |  |  |  |  |  |  |  | Data not analysable |
| Prognostic factors in childhood acute encephalitis | Rautonen 1991 |  |  |  |  |  |  |  |  |  | Data not analysable |
| Predictors of morbidity and mortality in neonates with herpes simplex virus infections | Whitley 1991 |  |  |  |  |  |  |  |  |  | Data not analysable |
| Intracranial pressure and outcome in adult encehalitis | Barnett 1988 |  |  |  |  |  |  |  |  |  | Data not analysable |
| Prognostic criteria in herpes simplex encephalitis | Foucher 1985 |  |  |  |  |  |  |  |  |  | Data not analysable |
| Clinical predictors for outcome in encephalitis | Kennedy 1987 |  |  |  |  |  |  |  |  |  | Inappropriate outcome |
| Detection of viral antigens in cerebrospinal fluid of patients with herpes simplex virus encephalitis | Bos 1987 |  |  |  |  |  |  |  |  |  | Data not analysable |
| Acute viral encephalitis in children | Wong 1987 |  |  |  |  |  |  |  |  |  | Data not analysable |
| Neonatal herpes simplex encephalitis: Correlation of clinical and CT findings | Noorbehesht 1987 |  |  |  |  |  |  |  |  |  | Inappropriate outcome |
| Incidence and prognosis of central nervous system infections in a birth cohort of 12,000 children | Rantakallio 1986 |  |  |  |  |  |  |  |  |  | Wrong patient population |
| EEG in neonatal herpes simplex encephalitis | Sainio 1983 |  |  |  |  |  |  |  |  |  | Data not analysable |
| Some previously unrecognized features of herpes simplex virus encephalitis | Williams 1978 |  |  |  |  |  |  |  |  |  | Data not analysable |
| Herpes simplex encephalitis. Prognosis and long term follow up | Elian 1975 |  |  |  |  |  |  |  |  |  | Data not analysable |
| Effect of Follow-Up Cerebrospinal Fluid Cultures in Post-Neurosurgical Patients' Outcome with Gram-Negative Bacterial Meningitis/Encephalitis. | Sun 2023 |  |  |  |  |  |  |  |  |  | Wrong patient population |
| Evaluating cognitive outcomes in adult patients with acute encephalitis syndrome: a prospective study from a tertiary care center in Nepal. | Rayamajhi 2022 |  |  |  |  |  |  |  |  |  | Wrong patient population |
| Varicella-Zoster Virus Meningitis and Encephalitis: An Understated Cause of Central Nervous System Infections. | Alvarez 2020 |  |  |  |  |  |  |  |  |  | Wrong patient population |
| [Clinical-etiological and MRI parallels of encephalitis in children]. | Skripchenko 2021 |  |  |  |  |  |  |  |  |  | Data not analysable |
| Inflammatory Immune Responses in Patients with Tick-Borne Encephalitis: Dynamics and Association with the Outcome of the Disease. | Bogovic 2019 |  |  |  |  |  |  |  |  |  | Data not analysable |
| [Tick-borne meningoencephalitis in Thurgau Canton: a clinical and epidiomological analysis]. | Schwanda 2000 |  |  |  |  |  |  |  |  |  | Wrong patient population |
| [Epidemiology and progress of early summer meningoencephalitis in Baden-Wurttemberg between 1994 and 1999. A prospective study of 731 patients]. | Kaiser 2000 |  |  |  |  |  |  |  |  |  | Wrong patient population |
| Unilateral hyperfusion in brain-perfusion SPECT predicts poor prognosis in acute encephalitis. | Launes 1997 |  |  |  |  |  |  |  |  |  | Data not analysable |
| [Herpes encephalitis in newborn infants. Retrospective study of 12 cases]. | Bigotte 1993 |  |  |  |  |  |  |  |  |  | Duplicate |
| Predictors of morbidity and mortality in neonates with herpes simplex virus infections. The National Institute of Allergy and Infectious Diseases Collaborative Antiviral Study Group. | Whitley 1991 |  |  |  |  |  |  |  |  |  | Duplicate |
| Clinical features & prognostic indicators of Japanese encephalitis in children in Lucknow (India). | Kumar 1990 |  |  |  |  |  |  |  |  |  | Cannot access full text |
| [Autonomic regulation in the acute period of tick-borne encephalitis in children]. | Kravtsov 1990 |  |  |  |  |  |  |  |  |  | Data not analysable |
| [The temporal dynamics of EEG changes in the early phase of herpes simplex encephalitis]. | Besser 1990 |  |  |  |  |  |  |  |  |  | Data not analysable |
| [Follow-up and prognosis of herpes simplex encephalitis. Observations on 57 patients between 1960 and 1987]. | Huber 1989 |  |  |  |  |  |  |  |  |  | Cannot access full text |
| [Herpetic encephalitis: prognostic elements in adults and children (49 cases)]. | Foucher 1985 |  |  |  |  |  |  |  |  |  | Duplicate |
